# Genome-wide identification and analysis of long noncoding RNAs in longissimus muscle tissue from Kazakh cattle and Xinjiang brown cattle

**DOI:** 10.5713/ajas.20.0317

**Published:** 2020-10-13

**Authors:** Xiang-Min Yan, Zhe Zhang, Jian-Bo Liu, Na Li, Guang-Wei Yang, Dan Luo, Yang Zhang, Bao Yuan, Hao Jiang, Jia-Bao Zhang

**Affiliations:** 1 College of Animal Sciences, Jilin University, Changchun, Jilin 130012, China; 2 Institute of Animal Husbandry, Xinjiang Academy of Animal Husbandry, Urumqi, Xinjiang 830057, China; 3 College of Animal Science and Technology, Northwest A&F University, Yangling, Shanxi 712100, China; 4 Yili State Animal Husbandry General Station, Yili, Xinjiang 835000, China

**Keywords:** Kazakh Cattle, LncRNA, Longissimus Muscle, Xinjiang Brown Cattle

## Abstract

**Objective:**

In recent years, long noncoding RNAs (lncRNAs) have been identified in many species, and some of them have been shown to play important roles in muscle development and myogenesis. However, the differences in lncRNAs between Kazakh cattle and Xinjiang brown cattle remain undefined; therefore, we aimed to confirm whether lncRNAs are differentially expressed in the longissimus dorsi between these two types of cattle and whether differentially expressed lncRNAs regulate muscle differentiation.

**Methods:**

We used RNA-seq technology to identify lncRNAs in longissimus muscles from these cattle. The expression of lncRNAs were analyzed using StringTie (1.3.1) in terms of the fragments per kilobase of transcript per million mapped reads values of the encoding genes. The differential expression of the transcripts in the two samples were analyzed using the DESeq R software package. The resulting false discovery rate was controlled by the Benjamini and Hochberg’s approach. KOBAS software was utilized to measure the expression of different genes in Kyoto encyclopedia of genes and genomes pathways. We randomly selected eight lncRNA genes and validated them by quantitative reverse transcription polymerase chain reaction (RT-qPCR).

**Results:**

We found that 182 lncRNA transcripts, including 102 upregulated and 80 downregulated transcripts, were differentially expressed between Kazakh cattle and Xinjiang brown cattle. The results of RT-qPCR were consistent with the sequencing results. Enrichment analysis and functional annotation of the target genes revealed that the differentially expressed lncRNAs were associated with the mitogen-activated protein kinase, Ras, and phosphatidylinositol 3-kinase (PI3k)/Akt signaling pathways. We also constructed a lncRNA/mRNA coexpression network for the PI3k/Akt signaling pathway.

**Conclusion:**

Our study provides insights into cattle muscle-associated lncRNAs and will contribute to a more thorough understanding of the molecular mechanism underlying muscle growth and development in cattle.

## INTRODUCTION

Consumers have become increasingly aware of the relationship between diet and health and have thus become increasingly interested in the nutritional value of food [1]. For customers, the tenderness, flavor and juiciness of beef are important factors in the judgment of beef quality [2,3]. Improving beef quality is the top priority of the beef industry because beef quality is the primary factor in determining beef consumption. The main factors affecting the quality of beef products are genetic factors, the fat content and composition [4], the packing method and animal breeding [5]. An essential step in improving beef quality is understanding the molecular regulation of muscle tissue.

Kazakh cattle are a Chinese native yellow cattle breed that is mainly distributed in the northern Xinjiang region, particularly Tacheng, Altay, and Yili. The coats of Kazakh cattle are mainly yellow and black. A new variety was formed using these cattle as the female parent and purebred brown cattle (Switzerland Alata black cattle and a small number of Kostroma cattle) as the sires. After long-term hybridization, the hybrids were selected to become Xinjiang brown cattle. These milk and meat type cattle were selected by this hybridization to resist cold and rough handling and to exhibit flexibility and adaptability to livestock farming [6]. Li et al [7] constructed a network of differentially expressed mRNAs and microRNAs (miRNAs) based on their expression in the longissimus dorsi muscle of Xinjiang brown cattle. Our previous research have found 46 differentially expressed circular RNAs and 346 differentially expressed miRNAs in longissimus dorsi samples from Kazakh cattle and Xinjiang brown cattle [8,9]. Although some related studies have been done, there has been no report on long noncoding RNA (lncRNA) research in Xinjiang brown cattle.

The latest developments in transcriptome sequencing have allowed the discovery of a new type of noncoding RNA that is surprisingly long, and this new type is called long noncoding RNA. Long noncoding RNAs (lncRNAs) contain two to four exons and over 200 nucleotides [10]. Prior to their discovery, little was known about lncRNAs, and these were thus considered transcriptional “noise”. LncRNAs exert significant effects on various biological processes, including reproduction, disease, and transcriptional regulation. LncRNAs exhibit four essential functions by acting as signals, decoys, guides, and scaffolds [11]. Sun et al [12] obtained transcriptome spectra of the longissimus muscle of Merino mutton sheep and small-tailed Han sheep using RNA-seq. Lim et al [13] performed RNA sequencing to identify differentially expressed genes in the longissimus dorsi of pigs with high and low muscle fat contents. He et al [14] performed high-throughput sequencing to profile the transcriptome of the gluteus maximus muscle of beef cattle embryos 135 days after fertilization and 30-month-old adult cattle. Trovero et al [15] revealed stage-specific expression patterns of lncRNAs in mouse spermatogenesis. Liu et al [16] identified a lncRNA-miRNA-mRNA network in clear cell renal cell carcinoma. Yu et al [17] identified a lncRNA biomarkers in esophageal squamous cell carcinoma. However, few studies have investigated muscle development-related lncRNAs in Xinjiang brown cattle.

In this study, the Illumina HiSeq platform was used to systematically identify and characterize the lncRNAs in the longissimus muscle of Kazakh cattle and Xinjiang brown cattle. A total of 11,964 lncRNA transcripts were detected, and 182 of these transcripts were markedly differentially expressed. These results will enable a better understanding of the regulation and functions of lncRNAs in cattle, provide good resources for cattle genome annotation and contribute to a better understanding of the growth and development of the longissimus muscle in cattle.

## MATERIALS AND METHODS

### Ethics statement

The experiment was executed in strict accordance with the guidelines for the care and use of experimental animals at Jilin University. All the experiments were approved by the Institutional Animal Care and Use Committee of Jilin University (license number: 201809041).

### Animal and tissue preparation

Three Kazakh cattle and three Xinjiang brown cattle were provided by the Xinjiang Yili Yixin Cattle and Sheep Breeding Cooperative. The beef cattle were slaughtered in accordance with the procedures of the slaughterhouse, and the longissimus muscle was collected at the slaughter line. Whole specimens were immediately snap-frozen in liquid nitrogen and stored at −80°C.

### RNA sequencing and quality control

RNA was extracted from six pieces of longissimus muscle (three pieces from Kazakh cattle and three pieces from Xinjiang brown cattle) using the TRIzol reagent (Tiangen, Beijing, China) in accordance with the manufacturer’s guidelines. The RNA quantity was determined with a NanoDrop rdt-2000 spectrophotometer (NanoDrop Technologies, Wilmington, DE, USA). After testing, the total RNA samples from the longissimus muscle tissue of three Kazakh cattle and three Xinjiang brown cattle were reverse transcribed and pooled for the construction of Kazakh cattle and Xinjiang brown cattle cDNA libraries. To construct these two libraries, we first extracted rRNA from the samples using an Epicenter Ribo-Zero kit and then added fragmentation buffer to the rRNA-depleted RNA. In addition, we synthesized first-strand cDNA using random hexamers with the rRNA-depleted RNA as the template, and buffer, dATP, dUTP, dCTP, dGTP, RNase H and DNA polymerase 1 were then added to the first-strand cDNA. AMPure XP beads were used to purify the cDNA. The purified double-stranded cDNA was subjected to end repair (poly-A addition and end ligation), and individual segment sizes were selected using AMPure XP beads. The U-containing cDNA libraries were obtained via PCR enrichment. In addition, the quality of the cDNA libraries was evaluated using the Qubit2.0 and Agilent 2100 instruments and quantitative reverse transcription polymerase chain reaction (RT-qPCR). The libraries were sequenced by BioMarker Technologies (Beijing, China) using an Illumina HiSeq X Ten platform.

### LncRNA identification

The transcripts obtained from the longissimus muscle of the Kazakh cattle and Xinjiang brown cattle libraries were assembled, and several steps were used for the prediction of lncRNAs. Four computational tools, coding potential calculator (CPC), coding-non-coding index (CNCI), Pfam, and coding potential assessment tool (CPAT), were used to screen for protein-coding ability. Two-exon transcripts with a length greater than 200 nt were reserved as lncRNA candidates and subjected to further CPC/CNCI/Pfam/CPAT screening to ensure that each candidate transcript was a lncRNA. CPC2 is a tool for calculating the protein-coding potential based on sequence alignment. By aligning a transcript with known protein databases, CPC2 evaluates the coding potential of the transcript based on the biological sequence characteristics of each coding cassette of the transcript. CNCI allows distinguishing coding and noncoding transcripts based on adjacent nucleotide triplet features, and a score less than 0 indicates that a noncoding transcript. CPAT determines the coding and noncoding ability of a transcript by constructing a logistic regression model based on the ORF length and coverage and calculating the Fickett and Hexamer scores. The Pfam database is the most comprehensive classification system for protein domain annotations. Pfam divides the protein domain into different protein families and establishes an HMM statistical model of the amino acid sequence of each family through protein sequence alignment. An aligned transcript is defined as a transcript with a certain protein domain and is thus considered a coding transcript, and an unmatched transcript is a potential lncRNA. We downloaded the *Bos taurus* reference genome from the Ensembl genome browser (http://www.ensembl.org/Bos_taurus/Info/Index), and the lncRNA transcripts were classified through comparisons as lncRNAs, intronic lncRNAs, antisense lncRNAs or sense lncRNAs.

### Expression analysis

The expression of lncRNAs was analyzed using StringTie (1.3.1) in terms of the fragments per kilobase of transcript per million mapped reads values of the encoding genes. This program uses a scaling normalization factor for each sequence library. The differential expression of the transcripts in the two samples was analyzed using the DESeq R software package. The resulting false discovery rate (FDR) was controlled by the Benjamini and Hochberg’s approach, and an FDR<0.05 and a log2 (fold change)>1.5 were used as the criteria for significantly differentially expressed genes.

### Target gene prediction and functional enrichment analysis

We found 100 kb of coding genes upstream and downstream of all the detected lncRNAs and predicted their effects. The names of the adjacent genes were subjected to gene ontology (GO) analysis using R software. KOBAS software [18] was utilized to measure the expression of different genes in Kyoto encyclopedia of genes and genomes (KEGG) pathways. The GO terms and KEGG pathways with p<0.05 were deemed to be significantly enriched.

### Quantitative polymerase chain reaction

The primers used for quantitative polymerase chain (PCR) were designed with Primer3 (http://bioinfo.UT.Ee/primer3-0.4.0/) and were synthesized by Changchun Kumei Bio (Supplementary Table S5). Total RNA was transformed into cDNA using an InRcute lncRNA First-Strand cDNA Synthesis Kit (with gDNase) and a FastQuant RT kit (with gDNase) (Tiangen, China), and RT-qPCR was performed using a Mastercycler ep realplex 2 system (Eppendorf, Germany). An INRCARE qPCR Test Kit (SYBR Green) and surrealistic premix + (SYBR Green) were used in accordance with the manufacturer’s instructions. Glyceraldehyde-3-phosphate dehydrogenase was used as an internal reference in the lncRNA analysis.

### Statistical analysis

The results are shown as the means±standard deviations from three independent experiments and were analyzed using SPSS 19.0 [19,20]. The significance of the differences was examined by one-way analysis of variance [21], and p<0.05 was considered to indicate statistical significance.

## RESULTS

### Overview of RNA-sequencing

**Figure 1:**
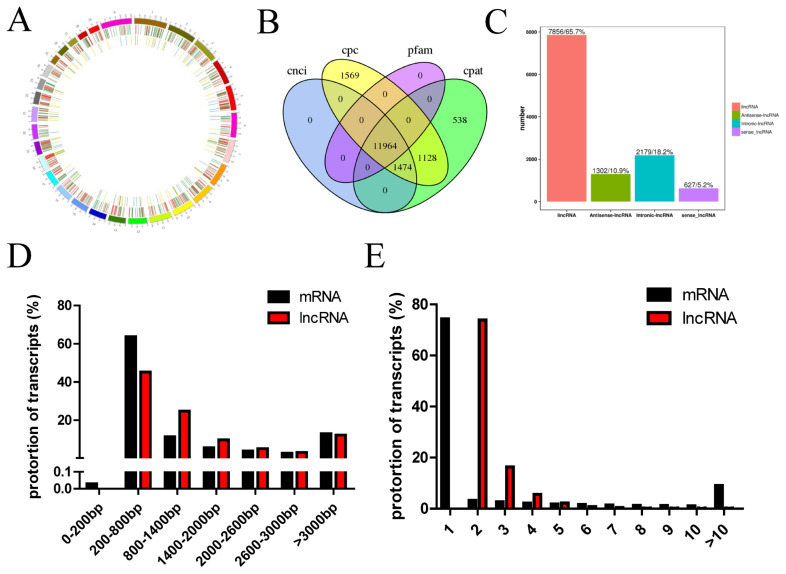
Identification of long noncoding RNAs (lncRNAs). (A) Comparison of the characteristics of the lncRNAs and protein-coding genes. (B–C) Classifications of the 11,964 lncRNAs, including lncRNAs, antisense lncRNAs, intronic lncRNAs and sense lncRNAs. Distributions of the sequence lengths (C) and number of exons (D) of the lncRNAs and protein-coding genes.

To investigate the lncRNA expression profiles of the longissimus muscle, two cDNA libraries were constructed from longissimus muscle tissues of Kazakh cattle and Xinjiang brown cattle using the Illumina HiSeq platform. We performed paired-end sequencing and the read length was 150 bp. In this study, a total of 115.43 Gb of clean lncRNA sequence data was acquired from six samples. The clean data from each sample reached 18.36 Gb, and the Q30 base percentage was greater than 92.89%. The clean reads from each sample were aligned with the designated reference genome, and the efficiency of the alignment ranged from 93.35% to 94.79%. Circos was then employed to visualize the putative locations of the lncRNAs in the cattle genome. LncRNA transcripts were distributed in almost all chromosomes (Figure 1A). The differential expression of lncRNA and mRNA sequences on the chromosomes was analyzed. The outer loop in the ring diagram shows the chromosome of the species reference genome, the middle loop shows the distribution of differentially expressed mRNAs on the chromosome, and the inner loop shows the distribution of differentially expressed lncRNAs on the chromosome. In the Figure1A, the upregulated and downregulated genes are shown in red and green, respectively, and the upregulated and downregulated lncRNAs are presented in yellow and blue, respectively. A cross-analysis using the Pfam, CNCI, CPAT, and CPC tools was performed to obtain 11,964 lncRNAs (Figure 1B), including 7,856 lncRNAs (65.7%), 1,302 antisense lncRNAs (10.9%), 2,179 intronic lncRNAs (18.2%), and 627 sense lncRNAs (5.2%) (Figure 1C). We detected 279,170 protein-coding transcripts, and the lengths of most protein-coding and lncRNA transcripts were within the range of 200 to 800 bp (Figure 1D). Most of the protein-coding transcripts comprised one exon, whereas most of the lncRNA transcripts comprised two to three exons, and the proportion of lncRNA transcripts with two exons accounted for 73.93% of all lncRNAs (Figure 1E).

### Identification of differentially expressed lncRNAs

**Figure 2:**
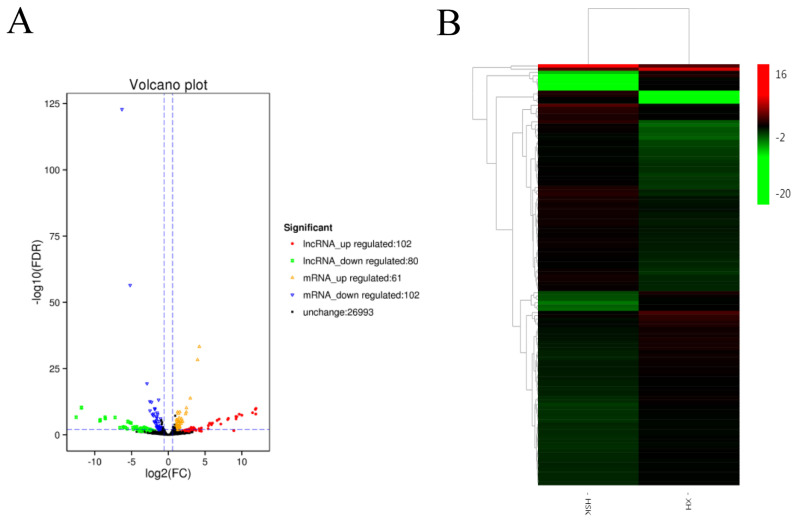
Long noncoding RNA (lncRNA) expression differed between Kazakh cattle and Xinjiang brown cattle. (A) Volcano plot showing upregulated lncRNAs (red), downregulated lncRNAs (green), upregulated genes (orange), downregulated genes (blue), and nondifferentially expressed genes (black). (B) Heatmaps showing the differential expression of lncRNAs; each column represents a sample, and each row represents a dysregulated RNA transcript. A red stripe indicates that an RNA was upregulated in a sample, whereas a green stripe indicates the opposite.

The DESeq R package was used to create volcano and MA plots of the differentially expressed transcripts. The volcano plot shows the symmetrical distribution of the main transcribed genes (Figure 2A). All abnormally expressed transcripts were screened to identify transcripts with a fold change ≥1.5. A total of 182 lncRNAs were differentially expressed in the longissimus muscle between Kazakh cattle and Xinjiang brown cattle (Supplementary Table S1), and 102 and 80 of these differentially expressed lncRNAs were upregulated and downregulated, respectively. A systematic cluster analysis was performed to elucidate the similarities and differences between the different libraries by examining the expression of differentially expressed lncRNAs. The DESeq R package was used for transcript expression analysis to detect lncRNAs that were differentially expressed between Kazakh cattle and Xinjiang brown cattle. Heatmaps were constructed to show the differentially expressed lncRNAs (Figure 2B). The results indicate the existence of particular lncRNAs that enable differentiation of longissimus dorsal tissues from Kazakh cattle and Xinjiang brown cattle.

### Enrichment analysis of the nearest neighbor genes of the lncRNAs

**Figure 3:**
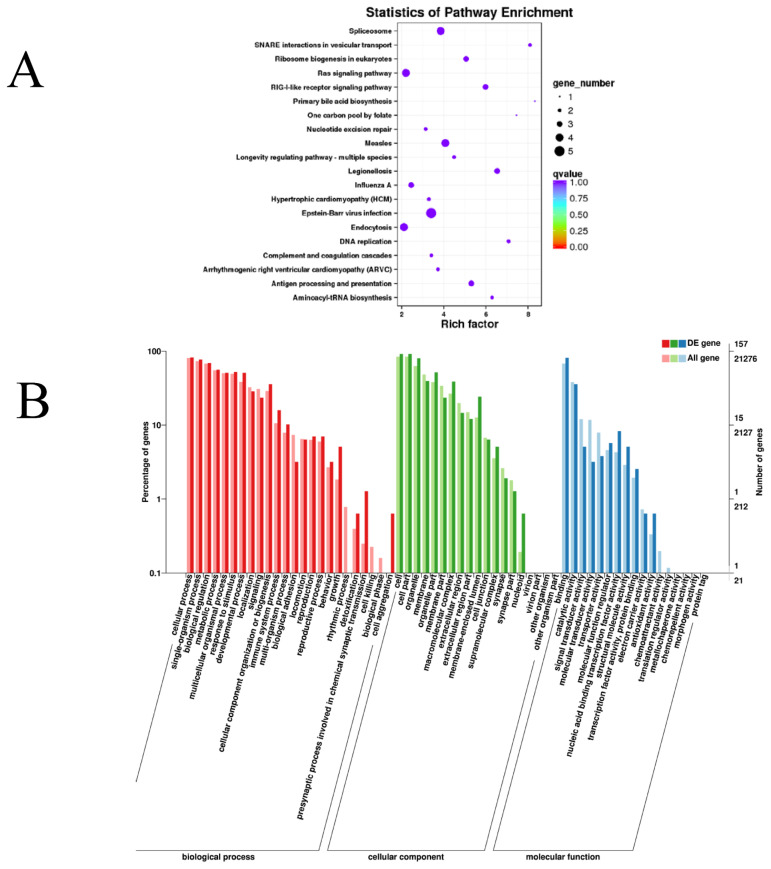
KEGG and GO enrichment analysis of target genes in muscle tissue. (A) Scatter plot of the enriched KEGG pathways of the differentially expressed lncRNA cis-target genes. (B) GO annotation classification chart of the differentially expressed lncRNA cis-target genes. The abscissa is the GO classification, the left ordinate shows the percentage of the total number of lncRNA cis-target genes, and the right ordinate is the number of lncRNA target genes. lncRNAs, long noncoding RNAs; KEGG, Kyoto encyclopedia of genes and genomes; GO, gene ontology.

We studied the potential roles of the lncRNAs by searching for protein-coding genes 100 kb upstream and downstream of all the detected lncRNAs to predict the underlying cis-regulatory targets of the lncRNAs. Overall, we found that some pathways associated with muscle metabolism and energy metabolism, such as the mitogen-activated protein kinase (MAPK) and phosphatidylinositol 3-kinase (PI3k)/Akt signaling pathways and signaling pathways related to spliceosomes, Ras, focal adhesion, and ribosome biogenesis in eukaryotes, were markedly enriched (Figure 3A and Supplementary Table S3). In addition, 60 GO terms were dramatically enriched (Supplementary Table S2), and these were mostly associated with single-organism processes (GO: 0044699), cellular processes (GO: 0009987), organelles (GO: 0043226), cell parts (GO: 0044464), biological regulation (GO:0065007), and other GO terms(Figure 3B).

### Construction of the lncRNA/mRNA coexpression network

**Figure 4:**
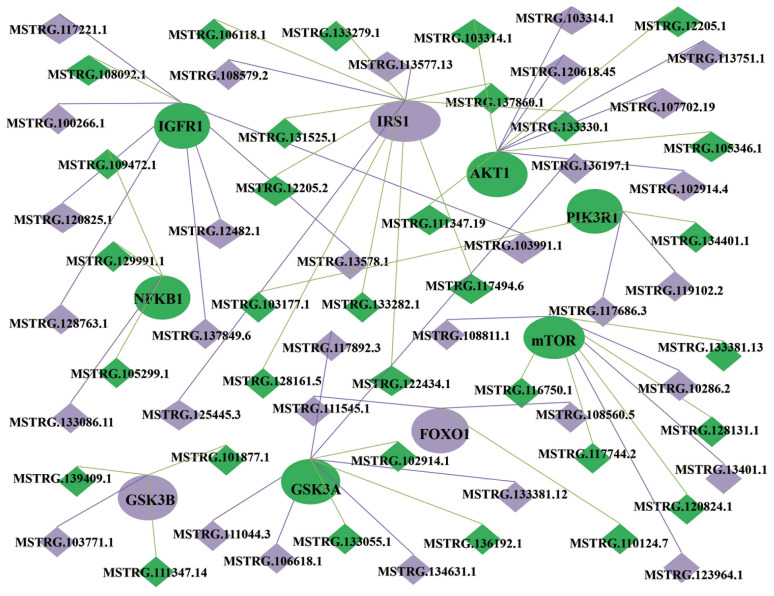
Construction of a lncRNA-mRNA coexpression network. The oval shape represents the mRNA, and rhombus represents the new lncRNA. The green color indicates the components that are positively regulated in the longissimus muscle of Kazakh cattle and Xinjiang brown cattle, the purple color indicates the components that are negatively regulated, and the gray color indicates that the components remain unchanged in either group. lncRNAs, long noncoding RNAs.

The possible interactions among mRNAs and lncRNAs can be examined through lncRNA/mRNA coexpression networks. Hence, a coexpression network was constructed using the differentially expressed lncRNAs and mRNAs between Kazakh cattle and Xinjiang brown cattle. Because the target genes of the differentially expressed lncRNAs between Kazakh cattle and Xinjiang brown cattle were enriched in the PI3k/Akt signaling pathway, we focused on the mechanism through which lncRNAs regulate genes in this pathway (Figure 4). The network suggested that insulin-like growth factor 1 receptor (IGF1R), insulin receptor substrate 1 (IRS1), AKT serine/threonine kinase 1 (AKT1), phosphoinositide-3-kinase regulatory subunit 1 (PIK3R1), mammalian target of rapamycin (mTOR), glycogen synthase kinase 3 alpha (GSK3A), glycogen synthase kinase 3b (GSK3B), forkhead box O1 (FOXO1), and nuclear factor kappa B subunit 1 (NFKB1) were correlated with 64 lncRNAs (Supplementary Table S4), which indicates that the lncRNAs and mRNAs regulate the PI3k/Akt signaling pathway during muscle development and differentiation.

### Validation of the differentially expressed lncRNAs and insulin-like growth factor 1 receptor-associated lncRNAs

**Figure 5:**
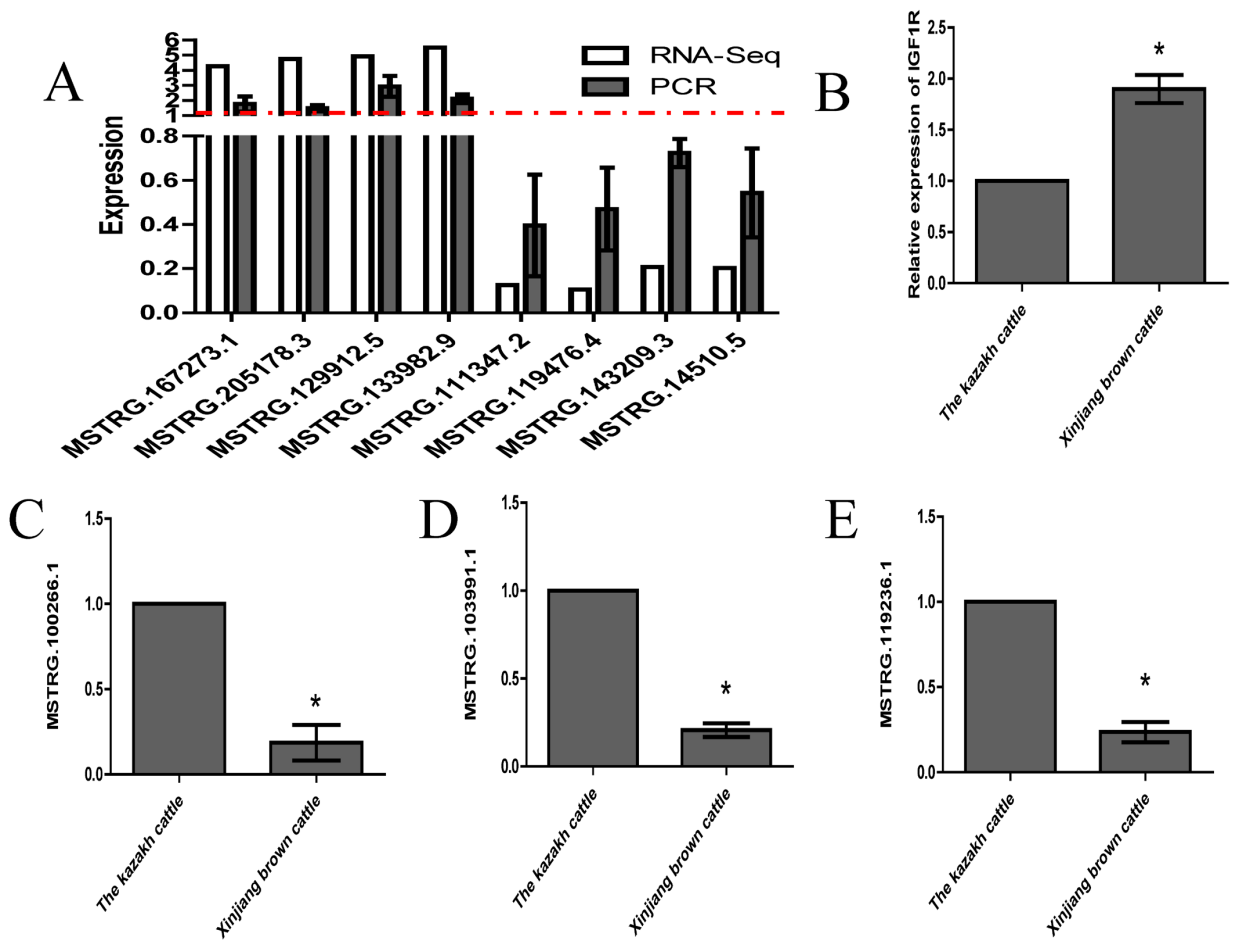
Quantitative reverse transcription polymerase chain reaction (RT-qPCR) validation of eight lncRNAs with differential expression and three lncRNAs that interact with the *IGF1R* gene. (A) Upregulated and downregulated lncRNAs in Kazakh cattle and Xinjiang brown cattle. (B–E) Expression levels of lncRNAs targeting IGF1R in Kazakh and Xinjiang brown cattle. All the experiments were performed more than three times. The data are displayed as the means and standard deviations. lncRNAs, long noncoding RNAs; *IGF1R*, insulin-like growth factor 1 receptor. One-way analysis of variance was used to analyze the statistical significance, p<0.05.

To verify the RNA-seq results, eight genes were selected for validation by quantitative RT-PCR (RT-qPCR). Eight lncRNA transcripts with differential expression were selected at random, and their relative expression in longissimus muscle tissues from Kazakh cattle and Xinjiang brown cattle was verified (Figure 5A). The RT-qPCR results were highly consistent with the RNA-seq results. Based on sequence complementarity, we used the lncRNA Targets platform to establish an interaction network between the lncRNAs and *IGF1R* genes. The expression levels of all three lncRNAs (MSTRG.100266.1, MSTRG.103991.1, and MSTRG.119236.1) and IGF1R were measured by RT-qPCR (Figure 5B–5E). The MSTRG.100266.1, MSTRG.103991.1, and MSTRG.119236.1 expression results were exactly the opposite of the IGF1R expression results, which showed that MSTRG.100266.1, MSTRG.103991.1, and MSTRG.119236.1 might regulate muscle growth and development by targeting IGF1R.

## DISCUSSION

Because long-term inbreeding limits the individual production performance of Kazakh cattle, and Swiss brown cattle were thus introduced and mixed with local Kazakh cattle to produce Xinjiang brown cattle. This success played a meaningful role in further improving the quality of beef in Xinjiang. The quality of beef depends on the marbling, texture, fat color, meat color and overall maturity of the meat [2]. The amount of fat deposition is positively correlated with the flavor and palatability of beef [22]. The deposition of intramuscular adipose tissue affects meat quality and animal productivity. Irish consumers can judge the quality of beef with great precision [23]. The European beef quality assurance system, which includes environmental and nutritional measures as well as dietary quality, needs to be profitable, simple, effective and sufficiently flexible to allow a company to develop its own brand [24]. With the improvement of the living standards of the Chinese people, they have established increasingly higher and higher requirements for beef quality.

Many studies have demonstrated that noncoding RNA plays a crucial role in the regulation of epigenetics. The common regulatory noncoding RNAs include small interfering RNAs, miRNAs, Piwi-interacting RNAs (piRNAs), and lncRNAs [25]. LncRNAs regulate gene expression through different molecular mechanisms both transcriptionally and posttranscriptionally. LncRNAs have been identified in some species of mammals, such as *Rattus norvegicus*, *Mus musculus*, *Homo sapiens*, and *Bos taurus*. Our results indicated that the quality of the libraries was sufficient for subsequent analysis. Most of the protein-coding transcripts comprised one exon, whereas most of the lncRNA transcripts comprised two to three exons, and the proportion of lncRNA transcripts with two exons accounted for 73.93% of all lncRNAs. These transcripts include novel transcripts and known transcripts, with novel transcripts accounting for the majority. Eight differentially expressed lncRNAs were randomly selected for validation of their expression levels in longissimus muscles from Kazakh cattle and Xinjiang brown cattle.

Most studies have shown that the expression of lncRNAs is associated with the expression of nearby mRNAs. Thus, to predict the functions of the identified lncRNAs, we searched for coding genes 100 kb upstream and downstream of the lncRNAs. We performed GO and KEGG analyses to reveal the functions of the genes and found that some pathways related to muscle metabolism and energy metabolism, such as the MAPK, Ras, and PI3k/Akt signaling pathways, were clearly enriched.

The signaling pathways that affect muscle development are the transforming growth factor beta (TGF), wingless type MMTV integration site family (Wnt), MAPK, and insulin-like growth factor (IGF) [26] pathways. An extremely conserved signal transduction pathway involving IGF1 and the intracellular cascade mediating its effect play primary roles in the regulation of skeletal muscle development. The core element of this cascade is the kinase Akt, which is also known as protein kinase B (PKB) and controls protein synthesis through the kinases mTOR and GSK3b and regulates the degradation of FoxO family transcription factors [27]. The PI3K and AKT/PKB are considered key nodesin signaling pathways involving insulin receptors (IRs) and their substrates (IRSs) [28]. Skeletal muscle hypertrophy is regulated by the Akt/mTOR pathway, which can stop muscle atrophy in the body [29]. Knockout of the IGF1R and IR results in a 60% reduction in muscle mass in mice [30].

Yue et al [31] constructed the lncRNA-miRNA-mRNA network in Qinchuan bovine, and hence, the relationship between lncRNAs and IGF1R is a meaningful topic of study. LncRNA-MEG3 acts as a molecular sponge by competitively binding to miR-135 to facilitate bovine myoblast differentiation [32]. The lncRNA WT1-AS suppresses migration and invasion by targeting transforming growth factor beta receptor 1 (TGFBR1) in triple-negative breast cancer cells [33]. We used the lncRNA Targets platform to establish a network of the interactions between lncRNAs and *IGF1R* genes. The inhibition of lnc-ORA by regulating the PI3K/AKT/mTOR pathway suppresses adipogenesis [34]. In our future studies, we will select lncRNAs that control muscle development and differentiation by regulating IGF1R.

In conclusion, we clarified the lncRNA profiles of the longissimus muscles of Kazakh cattle and Xinjiang brown cattle through RNA-seq analysis. Additionally, the cattle lncRNAs that were identified and characterized in this study might be associated with longissimus muscle growth in cattle. We further constructed a network of lncRNAs and target gene mRNAs. The results of this study will offer fundamental information that will enhance the understanding of the regulatory mechanisms related to cattle muscle growth.
