# Supplementary material for: Genome-wide identification and analysis of long noncoding RNAs in longissimus muscle tissue from Kazakh cattle and Xinjiang brown cattle
Source: Anim Biosci. 2020 Oct 13;34(11):1739–48. doi: 10.5713/ajas.20.0317 (PMC8563250; doi:10.5713/ajas.20.0317)
Supplement: Supplementary file 1 [file ajas-20-0317-suppl.pdf]

Table S1. Differentially expressed lncRNAs.

#ID	K_FPK	H_FPK	Pvalue	log2FC	gulate	sequence
MSTRG.200128.7	0	0.29	1.11E-10	11.93	up	CGGCTTTGGCTCGCGCCCCGCCCCGCGCGCCGTGGCCGTTTG<br>AAGTGACTAATTTCTGTCATATGACTGAGGCGCCTATGGGGG<br>TGGCGGGGCAGCTCTAGGAGCGGGGGCCTGGTGAGTTCCCTG<br>AGGAGCGACCCCGGCCTGGCCGTGGCCACCATGGCCCCTACC<br>CTGTTCCAGAAGCTCTTCAGCAAGAGGAACGGCCTGGGCGCG<br>CCCGGTCGGGATGCGAGGGACCCGGACTGCGCGTTCAGGTAG<br>GTCGCCGCAGGGCGAGGGGCGAGGGGCGCGGAGCCGATGGCC<br>TAGTGGGGTCCACTTTCCAGAGGCGCTGAGGCTTTCTCGAAG<br>TTCACGCTCGCGCAGTAGTGTCGCCCCGGTACCGGGATCAGG<br>GCCTTGGTCGGGAGGGGCAGGGCAGCTGCAGACCAGCGCTGG<br>AGGGTTCGAGCGTGGTCCTCTCCCCCCTCCCCCACGGTCGCC<br>CCTTACCCCCTTGTCCCGGGCGGTTTGTTTACGTCTTGCTCG<br>GCGGCCACGCGCGGCTCTGCCACTCCAGGCGGAGCATCGCGG<br>CCTGAGCTTAGGGGCCACACTCCTTCCTGGCGACGCGTAGAA<br>TTGGGCGCGGGCTGGATTGGACCTCAACCGTCGTCCTCCGGG<br>CCGAAAAGTTCTCCCCTCCTCCCCGCGGCTGGTCGTCCTCTG<br>GGTGCTCGGTTCCAGCTTGGTTGCTGTTGTGGAGAGAGCAGC<br>ACGTTTTGGCCAGCCTTCGGAGTGTAGAGAAGGCTGGAGGAG<br>CGTGTCGGCAAGCTGCCGTTCTTTCAGATTTCTTGTAAATCT<br>AAATTAGAGGTTTGCAGATACCGGACCGAGTGGCCTGAATAT<br>TGTCGAAGGAGAAATGTGAAACCTAAGAGGTTGTGCTTATTA<br>TTGACTAGAATGAAGCTGCGCTGAGTCAAATGAAGCATGGCC<br>CTAGAATTGACCATCCCGTTGGAACTGTTCCTAAAAACGAGT<br>CTATAAAAATTGACAATGATACCAAGATATTTTGTGGTATTA<br>CAGGCTTGGTTATAGTACCTGTTGCTGGTAAAAAAAAAAAAA
MSTRG.114761.23	0	0.28	1.49E-08	11.89	up	AACTGGAGGCGCGGAGACCGGCACGCAGAGAGAGGGAGGGAG<br>AGGCAGGGGACGCAGACAGAGGAGGGAGAGGGAGCAGCAGCC<br>TCGCGCGCCCTCCCTGCAGACGCCCTCCTCTGTCCGAGGGAA<br>GCGGCCGGGGGCGCGGAGTGGCAGAACGAGAGAGATGCGGGT<br>GCAGAAGACCGGGAGAAGGTCCCCTCGCTAGTGGGACCAAGA<br>GGCGCGGAGGGACCGCCGCGAGGGGCAGCTCGAAGAGCGGAT<br>CGCGGGACGGCGGGAGGCCCGCGGTGGGCTGCCGGACTGAGG<br>GACAGACGGCCAGACAGACAGACGTGCCCGGAACGGACTGGC<br>AGCCAGGAGGCGGGAGCTCGCTCCCTCTCCGCAGCCAGGGCC<br>GAGAAGGCGGCGGCAGCGCCCCCCGAGCGCATCTGAGCAGCG<br>GGGAGCCGCAGGGACCTCGGGGTCACCGGCCGAAGCCGCTGC<br>TGTGGCCGCAGCCCTGGGTGCCGCAGCCCTGCCGGGAGCCCC<br>GCCGACGCCGCAGAAGGCTCCTTTTATTAGCAGTTAAATAAA<br>TCGACCCAAAATAATAACAATAATAAGCCAAAGCAAACAAAT<br>ACAGCCAAGTCATCCGTGAGAAACAATAATACTAATAAAAGC<br>CACAAACCAAAATCAATCAAGGAACAGAGACGCTGGGGGGAC<br>GTCCACTTTTCGGTCAAGTCTCTAGGGGCAGAGATGGACCCA<br>CTGAGAGTTGTCTGGAAAGTGAAAGGAGAAGACTTCTCTAAA<br>ACTTTTTAATTGAGTAATTTTCACCATCCAAAAGGGGTGGCC<br>GAAGAGCCAGGTCATTATTGAATGCGAAGGAACTGAAATGGA<br>TCCAGGAAGGAGTTACCTTGCATCCTGCGCTGAGGAGTGCGA<br>CCAGGAGCAGCAACTCTGGGGTGGCAGGGGAGGTGGAAACAT<br>TTCCCAGCATCCTCTCGGAAGTTAAGGCAAGCAGAGCAGTGG<br>GCTTGCTGCTGGGCTCTCTGGCGCCCCCTCTGGCGGCTTTGG<br>TTTCCTGTCTCCAAGCCTTGAGAAAGCTGTTCGGAGTGCGCC

MSTRG.135889.2	0	0.31	2.54E-10	11.84	up	AAAAAATGTGCAGTTAGTGAAAGTAAGAGCTTGGTGGACAGATTAAACCACAGATTGGGCATATGTGAAGAGAGAACTGGTAAACTAGAAGATAAATCAAGAAGGTACCCAGTTCTAACAGAGGAAGGGATGGGAAATAGGAAAAAGCTAAGAAGCCCGGAGAACAGGATGAGAAGATCCAACATAAAGAATAGGGGAGGGAAGAGGTTTGAGGGTTTGTTTGTTTTTTTTTTCCCCAGAATTTATAAAAGAGATATAGATCTTCAAATGAAAAGACATAACAAATATTTAGTGGTGAATAAAAACAAGTATGCTTCTAGACATATAATACAATTGCAAAAGACGAGAAAAATCTAAGCAACTAAAAAGACCTTACCTGCAAAGGAATAATTGCACTGGTAGCAGGCTTTCTCAATGGCAGCAGTATGGACCAGAAGGAAACAAAATAAGTTATAAATGATGAAAAGAATGGGTGATAGCCTTGAGTTCTATACAGAGATAAGTTAGCGTTCAAAAGAGAGGCTGAAAAACACATTTTCAGATTTTGCTGTAAGAGCTCAGGGTTGTTAAGGAAAAAAGGCAGTTGAATCCAGAAAGGAAGGAATGGACTGCAAGAAGCCTGGTGAACAAAGAAATTGGTAAACAGACTGAAATTACACTTGTGTAAAACGAGAAGAACTTTATTAACTGATACGGAACAATCTTCAAGATATAATAAATGAAGAAGCAAGAAGTTGGCAACTGTGTTTGATTTGTTACTCTCATTTGTATAAACTAGGAAAAAGCACAATATACATTAGCTGCTTTATGCGTAAAATGCCTACAGGGTACACACAAACTATTGGGAGGACACCTGGGTGCCAGGGGACAGAGTTTGTTTAAAGACAGTTTTTACTGTATATTCTTTTATTCTTTATGAGTTTTCTAGCAGGACTGTAATCACTTAAAGTAGAAATAGAACTGGAAAGGTTGGGGAGGAAAGGTAAAAGAACACTCCAACAAAAATGGGGACCTTAAGTGACATACTTTTTTCTCTCCCAAAACGTATGA
MSTRG.171438.6	0	0.14	4.71E-09	11.45	up	GGCGACCACCTGCGCCGCCCCACTTACCATCTGCCCCGCGGGGAGGGCCCCCCGGGGTGGCGGAGCCCTCCCTGCTCCCGGGCGGCTCTGGGGGAACTCGGTGTTTGCCTCCAGCCCTTCGCTACCGGAGAGATCAGGTTCCTCCCCTCCTCCCCCTCCAGCTCCGGCCGCCGCCGCCGCCGCCTCCTTCTTGCATGACACAGCAGAACCGCAGCTGCCGCAGCAAGTCCCAACCATCCGCCCAGGAAGCGCCGCAACCGGGACTCGGGAGGGGCGGAGGGGGAACCAGGCAGGGGCCACAGCCGAGGGGGATGCTTGCCCCCGCTTTGCACCACACACTCTTTTCCCAGCCCAGGGGCATGGGAACAAAATCAGGCTCAAGCCGACCAGGCCAAGCCCGTGAGGAAAGCTGGGGGTTCGGCGTCAGAAGGGATGGGACCAAAGAGAAGGGTGTGAAGGAGACCCCAATTGAGGCAGGTCATTGCAGGGGAAGAAGGGGAAGAAGAGGCCGAGAAGTCGAGGGCCAGACTGTGAAGGAGAATAAAGGAGGTCAAAGAAAGGTCATATCGTCGTACCTCACTTCCTGACACAAACAAGTTTTTACTGTTGTCAGCAACAAAGTCCTAATATAGCTGCGGAAGAGAAAAACTGCATTGCATTTGCCTCCTGCAAGCATCATCAACACAGTTACCGGAGGAATGTTGATTCCAGAAAAGCTTTAAGGCTGGTACGATGGTAATTATGTATCAAATGCCTGATTCTATTTCTGTTATTATTGTTTTGTCATTTCTGAGCCACCCAAGAACTGGCTGAACTCGCAGAGGGACAAGTCCGGTTCTTCTTTTTGCCTCGGTAAAATTTAATCAGACCTGATAGAAAACTCATTGCTCTAGGGGAAAATAAAGTAGGAGCCACGAAATGTCATTTTAACAGAGCGTGGGTTTTGTGACTGTAGGAAAGGATTTAAGGACGCTCCTTCTGTTCGGTTTCCTATGTAATGAGCAAAGGCTACACGCACGCACAGACGCCACAGCTCCCGGATGCTGTGGC

MSTRG.117744.2	0	1.41	4.01E-08	10	up	CTTTTACCCCCCACCCCACCCACCAAGACTGCTGGACAGAGC<br>TGCTGCTGGGATGCAGTCTGGTACCTGGACTGAAGATGGGAG<br>GGAAGGGACACCGCCCTGGTGTTACTCCTACCTGGGTGGCTG<br>GAGGGTGACCAGAGACATCATCCTTCTCCTGTGTGAGCTGAG<br>GGGCATGAATGTAGACGTGCTGCGTGTGGGGAGGGAGGGAGG<br>CGCTAAAAGGCTCTTGAATGTGACCATTTTGGTGAGAAGGAG<br>GGAATGCTGTTTTTACATGTCTGTCCGGCAAGAGTCATGCTA<br>AAAATGCACAACCCCTGAGTAAAGACCACCACTTCCCCAAAG<br>AAGTCGCCCCATGTCATTCAGATGAGTGAGGAGAACGAGACT<br>GAGCCTCTAGGATGGAGCAGCCCCCAAGGGAATGCTTGGGGG<br>TTCACTGTGGGCCCCAAGGTGCTCCACGGGGGCAGCAGGGCA<br>CAGAGACAAAGCCCCGGAAGTATCAGAACAGACCGCGAGTAC<br>CACGGTGCCCCGGAGCCTGACAGACACCCTTGTTCCCGCCCA<br>GCGCTCCGTCCCCACTCCTCTCTGGCCATCTCCCACGGCAGG<br>GGCTGAGTCGGGGCTGCAGACTCAGCATCTCCCTGGTGCAGC<br>CTCCGCTGCAGCCTGGGGCGACCACGTGACCAGGCTGAGGTG<br>CCTGGAACCACAGCAGCCATGTTGCTACCATGAGAAAATGGC<br>CAAGAGAATCTCCAAGACACGGGCCTGACTGTCCTGTGACCA<br>AGCCAATGCCGGCAGCCACCCTCCTCCAGGATTCTTGTAAAT
MSTRG.177057.2	####	0.63	1.77E-08	9.646	up	GGGAGACCAGGTCATCCGTCCCTTCCCCCACCAGTCCTCTTA<br>GGGGTGGTGGAGATACGGGCCGCAGAGCTGCGAGTCCCGAGC<br>TGTGCGGATTCCCTAAACGCCGCACTTGCTCACAGCCCCTTA<br>CGTAACTGTCAGCGCCGGGGATTCCCTAGAGGGGGTCATTCT<br>ATTCAACCATTACACACACCCGGGGCTCCTGCCGCCGCGCCG<br>CCGCCTCACAAAATGGCGGCGCCCATAGAGGAGACAGCGGCC<br>GCCTCCTCGGCCCCATTTTGTGGGAGGCGAGAGATCTGTCAA<br>CATGGAATACCTCCGCTAAGGATGCATATGAGTTTGGAAATC<br>CTGCTTAAGGGATGGAGCCGAGAGGATCTGCTTCAACGGAAA<br>ATGTCAGCGACTTCTAATACCTCCACGCTTTTTCACTTTTTT<br>CATTTTCTCTAAGGAAGCGCCCGAAGCTGCCGGGGGACTTGT<br>AACTGAAGAGAGAAGGGAAAGCTGCAGCTGACTGCTCCGGGA<br>ACTTGCCGCGGCGTGTTTTGGCTTTGGCTGCTCTTCCCCGCT<br>CCCAGGAGCTGACCGCGGTCTCGTGGAGGGTCGCCTGGGCCG<br>CCCGGACGGCTCAGAAAGGACCGGGACCCGGCGACGCCAAGT<br>GGCAGCGGTCTCCGCCGAGTCACTTCAACAGCGAGACTTTTC<br>AGTAGTTTTTAAATGCCAGGGTCCACCGCCCAACCGGGACCC<br>CTTGCCAGAGTCGTGAGCTTCGGGAGCCGCGGACGCTCCTGC<br>TCCGTCAGTCGGCCGGGAGCCGGGTTGCCCGGAGTCGGCGGT<br>CCTCGCGGATGCCGGCAGCCACGGCGCGCCGGGTGGTCGCCG<br>TCGGGACCCGGGCCCTGGATGATGCTACTGGGGCAGGAGTGG<br>GAACGAAGGCACGTACACTGAAAATTAAGTCGTCTGGACCTA<br>GGGTTGTATGTTTTAACCGTGTTTTAGGTTAAACGTTAGAAC<br>GTTTTTTCTCGTATCCCACTGGAGTGGGCGAACCTGGCTGTC<br>TTATTTCGAATTTCAAGGGCAGGATGGAGGGGTGACATTTTC

MSTRG.79992.12	0	0.21	1.48E-07	9.315	up	GTACACAGCCGGTCCGGGCGGTGCGCAGGGGGCCGGGGCGCG<br>TCGCAGGTGAGGAGCGGGCACGAGCAGGCGCTGCCTGGTTCT<br>GGCTGGCGGGCGGGCGGGCTCACCCACTGCGGGGCGGGAGGG<br>CGGTGGGCCGCTGCCGGGGCTGGCGACTGGATGAGCTCGCGG<br>GGCGTCGCTCCCGCTCGGGGCCTCAGTGCTCAGGGCCGTCGG<br>CTCCCGCCCCTTGCCTGTCCGCTGTGGAGGACGCCGGGCTCA<br>GGGGGAGCTGGCTGGGCGGACGGAAGCCCGGAGAGGCCTCGC<br>GGCTCGTGCTTGGGACTGGGGGCGCGGCAGGGACCCCGCGGA<br>TACGGAAACCCCTTCGAGGAGTACGCGGAGGGACCTCGGCCT<br>ATGGGTCTCGGGGTCCGCGGGGACTGCCTAGGGACGCGGCCC<br>TCACGCCGCCCTGCGGCTGCCGGTCGGCTGAGCTCACCTTCC<br>CCGCCGGGGCCGGGCCGGGCCGGGCCCCAAGGAACACGATTT<br>TCCGGCCCCGCTGAGCCCGTTGGCGCAGTCGGGCCCGGGGAG<br>GTTGCGCGGGGACCCCGAGAGGGCCGGGGATGAGGATGAAGG<br>ATCCAAGCCCGAGGGGCGAGCGCAGGGTCAGGGGTGTGTCTT<br>CCTGCTCTGCTCGGTGCGAGGAAAGAAGCTTGCCTGGTTGGC<br>ATGATAAGAATTTTATTTATTCTCCTCACAAATTTTGCCAGA<br>TGGAATACTTAGCTTTGAGGGGAGAGGCCCTAATTTGAAAAT<br>AGCTTTCAAATTCCGCCCCTCACTCCCATTATAAAAGCAATT<br>CATTCTCCTTTCTTCTCGCTCAAGACTACACAAAGTGTTGTG<br>ACAGTACTTTTTAAAGTAACAGTCATCATGTAACCTACCATT<br>CAGTGATAGCCATTGCTAAGATTTTGTTGATACCAATAAATA<br>CATGTATTATCACTTTTATGGACTTCATTATACCTTTGTATA<br>TCACAATTTAACCAAGTCCTACTAATGAACATGTAAGGTGCT<br>TCCAATTCGTCATGGGATAGATGATTCTGCCACAGAATTTGT
MSTRG.41883.2	0	0.15	1.10E-06	9.213	up	GGGTGGGCTCCCCCTGAGCCAGAGTAAAGGTGCTCAGGCCTC<br>TCATTGTGGTGGCTTCTCTTTTGCAGAGCACAAGCTCTGGAG<br>CTCATGGGCTTCAGTAGCTGTGGTGTGGGCTTAGTTTCCCTG<br>CAACATGTGGAATCTTCCTGGACTAGGGTTCAAACCCATGTC<br>CCCAACATTGGCAGGAGGATTCTGAACCACTGGACCACCAGG<br>AGAGTCCCAGAGTCTTTATTTTTCAATTCCACAGGTTAAAAA<br>CAATGCCTTAGATCATTTGCCTCAATCCAAGATACAAATGCC<br>AATCCAAACACTTGGGTAAAGAATTGGATTTTGATTGATTCA<br>AATTCACAGTTCGGTTCAGGATTTTATCTTATTTTATTTTAT<br>TGTAAGTAGAAGAGACAAACAGACTCACTTGTGAAATCAGCG<br>AACTATATGTTAAATAGAACCATCCTTTTTCTTCTGGATGAA<br>TTTCTACTTTTTAAAATAGATCATTGAGTTATCATTTTGAAT<br>CCTGAACACGATTTTTGTGAAAATGTACTCTAAAAATATACT<br>AGGAATTCCAAAAATCAGTTTCATTTCTTAGGATCTCCAGCT<br>TAATGTACATGTTCCAAAAAAGCTTAGATTTCTTGGACTTCT<br>AACACTGCCAACTATTTTAGAATTTAAAAATCAAATTGTGTT<br>CATTTTTCATGTGTTGTTATGAAGTTACAACCTGTCTTCCAT<br>ACTGACCTCTCTCCCCATGTATAGAGTTTACAGAGCATTTAC<br>ATTTGTGGTCATGCTAGCTTCACTTTTAAGAGAGAGCCTAAT<br>GGAATAACTAAACTTTTCCTGATCTTTTCCTGCTACTGGTGG<br>TATTTCTTAGCTATTTTTAAATTATCTTTGGGCTTCCCGCAT<br>GGTGCTAGTGGTAAAGAATCTGCCTGCCAATGCAGGAGACAC<br>AGGTTCCATCTCTGGGTTGGGAAGATCCCCTGGAGTAGGAAA<br>TGGCAACCCACTCCAGTATTTTTACCGGAAAAATTCCATGGA<br>CAGAGAAGCCTGGTGGACTACAATCCACACGACTGAGCAGAC

MSTRG.155031.14	0	0.08	1.26E-07	9.193	up	CTCTAAGGTTGTGAGCCTGGTGCATGGTGGCAGTCCCCGGAG<br>TAATGAGGTTGTGTTGACTGTAACTTCATAAGCTCAGTGGTA<br>TGCAAATCTTGCATACAGAAGAATATTTAGAAAGAAATGTCC<br>TACCAATAATTAGAGCCAAAGGACTGAAGGGACAGTGGGAAG<br>TCTCAGCAGGAAGGCTAGTTGGAACTGTTGCTACACGTTTGG<br>GATCTGGTTCCCTGACCAGGGATCAAACCCGGCCCCCGCTAC<br>ATTGGAAACTCAGAGTCTTAGCCACTGGACCACCAGGGAAGT<br>CCCTGTTTTTTCATATAGAATGTACTGTTCTGTGTCTTGCTC<br>TGTTGCTCAACATTATGTTTCTTATTTGAAAACATATGTCTC<br>TCAGTCACCAGGAAATAGAAGTTAAAATCACAGCAGTATTCC<br>ACAATATCCAGTGTTCGTGAGGACGTGACGCGCTGGGTCCCT<br>CATACACCTCTGGTTAAGAATAACTTGATGCTCCTGAGACGT<br>GGCCTGCACAGAAGAGAGGACTGTTCCTGCAACTGAGACCAG<br>TTGACTGCCTTTGGAAACTCACCATCCGTGCCACAGTGGCGT<br>CTGTTTCCCATGTGGAACAGCAGTTATGACCAGTAAGTATTC<br>TGTTTAAGAACCCTGTGTTAAAGACTTGATATTCTTCAACAC<br>TTCAGCGGAAGCCCCACTGCAAAGGAATCATCTACTCAGGTC<br>AACTGTTGATAGAATGGCTCAGCCAAGGCACTTAGCTGTGAT<br>CCCAGAATGATATCTTCAGCACTGTCTGGTGAAGAGAACAGT<br>CTGAGAGACATTCCATTTATTTTCTCAGCTACCTGGTGATGA<br>AGATGTTATTTCCTCCACATCCTATATGAGGGAACTGAGGTC<br>TGGGGTGTTAAATGACTAAAGGGCAGAGAACACAGTCAACAA<br>GTAGAAGAACCAGGGCTCTCCTCTGAGGTTTTCTGGGTTCAG<br>ACCCTGTGTTCTTGCCATTCTACACATTAAGTGATTAGGGAC<br>TTACTGATGAGCAGCGGATGGATTGTGGTAATGCAGGAAATC
MSTRG.16644.1	22.9	9670	0.02689	8.915	up	CTGGCAGGGGAGATACCATGATCACGAAGGTGGTTTTCCCAG<br>GGCGAGGCTTATCCATTGCACTCCGGATGTGCTGACCCCTGC<br>GATTTCCCCAAATGTGGGAAACTCGACTGCATAATTTGTGTT<br>CTGGGCTGCAGTGCGCTATGCCGATCGGGTGTCCGCACTAAG<br>TTCGGCATCAATATGGTGACCTCCCGGGAGCGGGGGGCCACC<br>AGGTTGCCTAAGGAGGGGTGAACCGGCCCAGGTCGGAAACGG<br>AGCAGGTCAAAACTCCCGTGCTGATCAGTAGTGGGATCGCGC<br>CTGTGAATAGCCACTGTACTCCAGCACGGGAGTTTTGACCTG<br>CTCCGTTTCCGACCTGGGCCGGTTCACCCCTCCTTAGGCAAC<br>CTGGTGGTCCCCCGCTCCCGGGAGGTCACCATATTGATGCCG<br>AACTTAGTGCGGACACCCGATCGGCATAGCGCACTGCAGCCC<br>AGAACTCCTGAGCTCAAGCGATCCTCCAGCCTCAGCCTCCCG<br>AGTAGCTGGGACTACAGGCGCGCGCCACCGCGCCCGGC

MSTRG.1356.1	0	0.15	6.56E-07	8.17	up	CGGGTCCGGCTCCAGTATCATCAGGAGGACAATGAGAGGTAGTAATCGATAGGTAACTGACAGAAGCATGCAGGATATCTGAGCCAAAAGTCGCTCCCCTCCATCTTCCCCAACTAAGAATGCTGTTTTAGTGATCAAGCTGAAGTTCCAGTGTTTTGGTCATCTGATGTGAACAGACAACTCGTTAGAAAAGTCACTGATGCTGGGAAAGATAAAGGGCAGAAGGAGAAGAGGGCGTCAGAGGATGAGACGGCTGGACATCATCACCAATACAATGAATATGAGGGACAGGGAGGCCTGGTGTGCTGCAGTGCCTGGGGTCACAAAGAGTCGGACATGACTGGGTGACTGAACAACAACAAGCAAAGGTATTGAGACAAAAAGGTATGAGCTCCATGGAAGTAGGATGGAAGGAAGTTACTAAGAAAGCCAGAAGAAGCGACAAAGAGGAGACGGGGTCTATTTGACTGAATATGATGAAAAGCACTTTGTCATAACCAGCAAGTTCCCACAAAGGGAACCCTTTGCCCAACCCTGGCCACGCTGTCCACAAAAATTCAGATTTACCCCATGCCCTGCCCCAGGGCGGTGGGACGTGAGTGCCCAAATCTTTGGGGAGAAACTGGGTGAAGCAATAGTAGTGGTTCTCTAGCCTTTCTCGAGGATTCTTGCTTAAATGCTGGGCAGATCTGGCACCAGTGTGACTTCCCACTTGCTTGGTGACGTGATGAACGAGTGTCCTTTGGTTTCTTTCTCTTTTTTCTACCCACATGCAAACTTGACTCCTCTCATTATGGTTTCAGGTAAAAACAATGCAAAGCTTGGCGCCAGAAATTCCATTCAAGACAGAACTTTGTGGTTTTACCAGACTTGTGAGTCCTGAGACTTTATTTTAACCCACAGTTTATAACCTCAAAATAGTCCAACCCAGGCAAGTCAAGTTGTTTTTAGAAGAAGAAAGTTCTCCAGAAACCTAAAAATCCCCTATCTTTCAGTTCCCAAGTGTTCCATCATTGAAGTCACAATTCCCATATTTATTACATGAGAATC
MSTRG.6892.3	0	0.12	1.87E-06	8.087	up	AATACACAAGAGCTCTTTGGTTCTCTTTTTTGGAGGAAACCTGAGCCATATTTTTATTATCTCTTATGTCATCTTAGAGTAAATTTTAGAAATATATAAGTATTTTAAATTTCCATCTCATTTAAACCTAATATTTTTCCCAAGTTTGTGTGATACATTTACTTAAAAAAATGTATCCTCACTTTTCCTGTTATTTACCATTTTCAAAAAGATAGGAATCGAAAAATATATTCAAACATTTGGCTCATGATTCACTGGTTTTTTTTTCAGCCTGTATCCCTCTTTGATATTTTCAGCCACTGCCATTAGCAAGATTTTGACTTACCCCAAAGTTAGTGTTAAAAAAACAAATCAGTTTGAAGAGTGGTGGTTGTAGAGAAATTATTACATTCTTTACTTCAGTAGCCCTGTGTTTACTAAACAGAAACAGAGAACTGGAGGTGAGTTACAACCAACATTGGAAGCTACAGATTATCTTTATGATGTTCTTATATAACATGTTTTATAATCAGGTCCCTTGGATTTCAGGTGGAATCTGCCAGTTCATAAAGCTAAATGAGTCTTCCTAATAGAACTTTTTGGGTCCAGTTACATAATTTTCAGTTTCCCTGGGTAAGGAATTGATTCATATTTACTTTTTTTCACTTTCTGAAGAGGTGGGTTTATTTTTGGTTCTACTTTACACTCACCACTTGGAGGAGCTCATTATGACATGACATACTTTTGAGTGACTAATAATGTCTAGGGAGTAACTATTCTCATTTCCCATAGATTCATCCCTCACATATGCTTAACATCATTTATTGGTCAGTTTCTGTTTTCTTTAGGTTACATATTCTTTACTATATTAGTATACTGTGTTTATGGACAAAAGAGTAAACAATGGTTCAGAAAAACTTTCTCAAAAGGTACTCAGCTCTGCTAAACTTTAAAATTCCAACCTTACAAACCCCAACATACCTGTCACTCTAGTCCAAGGGACTGATGAGAAGAAATTGGTCAGTAGGAAAAGTTTGAGCAGTTGGGTGAATATTGATTATTTCAACCATT

MSTRG.112338.2	0	0.09	9.39E-05	7.136	up	CGCGCCGCCGCCGCCTCCTCAAGCTGCCGCCGCCATGTTTTC<br>CTTCCTTATACCTGGCCCTGCCACCGCAGCCGGACGTTGTGC<br>TCCTCTCGTCTGCTTCAAATGTCCGAAGCTGACGGGCGCCAG<br>GTCCCGCTGCAACTTCTACCACTGCGCGGAAGACTCCGAGAG<br>CCCCCAAGCGCACGAGCGCTTCTGGCTACTGGGAGTCCCTGT<br>CCCTCCGCCGCCGCCGCCGCCTCTTCCGTCTTCCTCACCGAG<br>CCAAAGCCGCCGCCATCTTGGTTCGCCCCTCAATAGTGGCGC<br>GCGCGGGGCCACCCAGGCAGCGGCTGCAGGGGCGGCCCGTTG<br>GCCCCACCATTTAAAGAGGCAGGAACGAAGCTGAATCAATCC<br>AAGTTTGCCAGAGCACGTGGCTTCCTCTTCAAATACCAACAG<br>TGAAAGATTCTGGGTTGATCTGTTATCGGACCCCACGTCCAA<br>GGTCAGGGGTGGCGGCTGAAAGGAGCAAACCCACGTCCAAGG<br>AGCGGCTGTTGCGCGGGCGCAGGAGGACCAAGAGGAGCTACT<br>CCACATTCAAGGTCAGGAGGGCTGGCTGTGAGGAGATACCCC<br>ACATCCAAGGGAAAAAGCCTCCAAATCATAAAGAAGATAATA<br>TAGACTCTGGTAGAGAATAGAGAAGAGGAATCCTTTAAAAAG<br>AAAAATTAAATCTCTACAAAATGTATAAAGCATGGTTGTGAG<br>AAAGGTGGATTCTTAACCCATTGAACCATCAAAGAAGCCTGA<br>ACACCGAAGAACTGACGGTTTCAAATTGTGGTGCTGAAGAAA<br>ACTCTTAGAGTCCCTTGGACAGCAAGGAGATCAAACCAGTCA<br>ATCCTAAAGGAAATCAACCCTGAATGTTCATTGGAAGGACCC<br>TTGCTAAAGCTGAAGCTCTAATACTTTGGCCACCTGATGAGA<br>AGAACTGACTCATTGGGAAAGACCCTGATGCTGGGAAAGATT<br>GAAGACAAAAGGAGAAGAGGGGCGACAGAGGATGAGATGGTT<br>GAATGGCATCGTCAACTCAATGGGTATGAATCTGAACAAACT
MSTRG.192050.4	0.01	1.05	1.25E-06	6.912	up	CGGGCAACTTGGATGTTCTCTGCGCACACTGGAACGTTCCAT<br>GTTTGCAGCGGGTGCAGACCAGCAGAGAGAAGAGATTCCATC<br>TTCCAGAGGTCGCCACTGTCTGCCTCCCCACTTGTCCCCATC<br>CTCGGTCATCTCTTTTAACACATTATTGACCAGAGATGTATT<br>AATATGTCTTTTGTTACCCAAATGTTATTGACTGGAGATATA<br>TCAATATCACTTGATAAGAAATTGCTGGCCACAGGTGTTAGT<br>CTCAGCAAAAATCCCCCCGGTCAATAATGAGTTAATATGTAG<br>TGACACATTAGTTGTGTAGTTATTCATAGTGAATGTGGTTTA<br>AATCCTACACTATCCTCTTGACTTGAAATATGAGCCTTAGTG<br>TTTTTCTCACTGCAGTGAATCTTTGAGTTTGATGAACTCTTT<br>CCCTAATGATATTGACTGTTTTCAATTAACAGATTTATTACC<br>AGTTTCTGGTATAGAGTCTCAAATGAAACAAAGTATTTTTGA<br>TTGTCTTTCATATCTTTGGCTAATTAGATACTTATAATGGAA<br>CATATAAATACTTTTTTAGAGAATTTCAAGGAATAGTGTTTA<br>ACAGAATTTGAAAATTACAAAATTATTATTTATTGTTTAATA<br>TTTAATCTCAGAATGGTTTCCCTTTAAAATTTGCAGTGAGTT<br>TTTCCCCCATTTACTCATGATAAAATAGATTTATTAATTGAA<br>AGAAAATATATTTAAATGTCAAGGAAATAAATGAATAAGTCC<br>CTTCTATTTGTTGCAGCTTATTATTTCTTGAAATGAATTACC<br>TAAAAAGCCAAAAAGTTACAAATAAAATATTCTTGGTGATTA<br>AATTTTCCCAATTTGGCCTTGCTGTGTGAGAAGTTTACAAAT<br>CAAAATGTGCTTGGGTTGTGAAACATCATATGAGCCTGGCAC<br>AGTGTTTTTAAGTGTATGGAGCTGGGCAGCAGTCTATCTCAT<br>CTGGAGGCAAGGTTACAGCAGACTTTTATATTAATGCCGTTC<br>TCGAGGCAGATTTTCGGGTATTCTCCAACAGAGGCTTCCCCA

MSTRG.224405.8	0	0.28	3.74E-06	6.636	up	TTTTTTTTTTTTCCTTTCGTTCCCTGTCCATTTTATCTAGTT<br>TTATATAAAAAGTTCTGGGATCCCCACAGTGGGGTTGAGCCA<br>AGATGGTCAGGCCCCTTGGCCTAACTCTTGCCCCAAATAACT<br>GTTGGTCAGATATCGCAATGTGAATTTCTCTAGCCCCTTAAA<br>TAATGGCTGCAGCCTGATGGCCACTAGATCATAGGTGTTCTC<br>CTTCCTGAGTGCCCTTAGGGCTCACCAGCTCTCGTAGGAAGG<br>CTGCAATCAACTGGTGACTATAGCATCCTCGTTTGCTGGTAT<br>GGCAGAAGCTATTCCATTTCTCACTTTCTTAAACATCTTTTA<br>TTTCTTCTAAGCCTACATCCCTTTCCTCTCTCATTTCCTTAT<br>TAATGTGATATTTTGTGTGTGTATATAAACTGCTTTTTATCA<br>TTTATTAAAATTTTTTGTATTGTATTGAGATATCTATTATAT<br>AAGTTTCAGGTATACAGCACAGTGATTCACAATGTTAAAATG<br>TTATTCTCTGCTTATAGTTATAAAATATTGACTGTATTCCTG<br>TGCTGTACAATTAAAGGTAGTATTTAAGTCTGAATTCTAAGG<br>AGATACTCATTTTTCCCTGGGTATCTCCCATATATACATGAG<br>GTATACATGTTAATAAACTTAGCCACCTGATGCAAAGAACTG<br>ACTCATTGGAAAAGACCCTGATGCTGGGAAAGATTGAAGGCA<br>GGAGAAGGGGACAACAGAGGATGAGGTGGTTGGGTGGCATCG<br>CTGACTCGATGGACATGAGTTTGAATAAACTCTGGGAGTTGG<br>TGATAGACAGGGAAGCCTGGTGTGCTGCAGTCCATGGGGTTG<br>CAAAGAGTCAGACACAAGCCACTGAACTGAACTGAAACTTTT<br>GTTTCTTTTTCTCTTGTTAATCTGTCTTTTATTAGAGCCTAG<br>AACTCAGAGGGGTAGAGGGGAAATTATTTTTCCTCCCCTACA<br>ATACGAATATTTTAGAAAATAATTTTGTGTATCTATCTATGT<br>AGTCGTGGCTGTGCTGGGTCTTTGTTGCTGTGAAGGCTTTTC
MSTRG.102013.2	0	0.25	3.86E-05	6.009	up	GGAAATAACAGCACTAACTGGAAGAGACGTGTGACCGCATGT<br>TCATCACCGCGTGGGTACAGTGACCAGGGCGTGGGCACAGCC<br>GAGGCGCTCGCTGATGGATGAGCGGATGAAGCGTGCGACGCA<br>CGTACGCGGTAGATGCCGTTCAGCCACAAACAAAGAAGACCC<br>TGCCTGTGTGACGGCATGGATGGACCCTGAAGGCCTTATGCT<br>GAGTGAAGTAAGTCAGAGAAAAGCAAACATCATACGGTCTCA<br>CTTGTTGGTGAAATCTGGGGAAAAAAAACAACGAACCAATCC<br>CAACAAAAAGAGCGTAGTTCCGGGAGTCACTATGAAGCCAGG<br>GGAAGCAGACTTGGGGCTCTGGAGAATGGAGGAGCCAGGCGT<br>TGGCGCATGGGGACACCATTTATCTGATGCAGAGGCAATGGA<br>AACAGTTTTCATCAAATACCTGTATTATAGGTTATGGTAAAA<br>GTCCCAGATGTTTATATGAAGACGAAGTGAGCCCTCACTCCC<br>TGTAACTCTGTCTAGCTGCTGAATTCCATCTGCAGCCGGTGA<br>CTCTGCCCCAGTGAAAGCTCTTCTGGGGTGTCACGTGGCCAA<br>AAGCAGACCAACTGAAATTCACAAGGCACGGAAGCTGTCTGC<br>CACCCTCCCCGGGCTCTGAGGGTGAGCATCGTGATCTGGAGG<br>GAAGACGGGCAAACGTGCTTGGGGCCCGAGGCGGATGTCACT<br>CTAGTCGGAGCTGATGTGACGACCTGTGTCACCTTCGCTCAG<br>CACCCCAGACTCTTTCAGCCGCCCGTCCTTCCATGCCTGTTT<br>CTGAGCCAATCACCAAGGCCAGGGGCCGAGGGTGGAGGATGC<br>TGCTTGGCCAGACGTGGGGCACGGTGCCCACTTTCTGAGGGA<br>GGGGAAGTGCTTTAGCCCCACCCACCCCTAAGGACCCATGGA<br>AAGGAAGAGCATGGCGATGTCTCCCGAAGGAAGAGAAATGGG<br>TGGCAGGTCCTGCCTTCAGACGAGACCAAAGCCTGAAGCCTG<br>TTCTTTGAGGAGGAGTCTGGAGTTTCCATCAAAAGTGACTCC

MSTRG.54320.15	0	0.16	0.00016	5.861	up	CGTGTTCAGTGCCGTGTGGGCGTCCGTCCCAACCTCCCACTTCACCCCCCACCCCTCACCCCCTGGTAACCGGAAGTCTGTTTTCTACACCTGTGACCCCGCTGCTGCTTTGGAGGTAAGCTCTTGTGCGCCTTTTTTAGGCCCCGCACGTGGGCGACACCGCATCCGTTTGCCTCTCCCATCTGACTTGCTTGGCCCCGTGTGACAGTCTCCGGTCCGTCCGCGTCGCGGTAACGGAAGCGCCGCGCCCTCGCCTGGGCCGCCGCGGGATTCAAGAGGTGCTTTCTCTCTGCCCTCCAGGTCTGTCAGCTCGTCGTCTCTGTGAACGGGCTTAACGTGCTGCACGTCGACTACCGGACGGTGAGCAACCTGATTCTGACCGGGCCGCGGACCATCGTGATGGAGGTCATGGAGGAGCTGGAGTGCTGAGCGGGGCCGCGGGCCTTCACTGCGCACGTACGCGTCTCATCCGAGTTTCCTGCACTGCTTCCCTGTTGAATGTGGGAGAACCAGGGAGTGTGTCTTTAAAAAAGAAAAAAGTGAAAAAGACAACGTCTGTCGGTGCAGAAAAAGTTTGGGACACAGAATTTTTTCCCCTACGTAGTTGCGTTGCCTTACTCAACTTCCGTTTGTGGTTCTCGTCCATTGTTTTTGGTCTTGCTTCACAATAAGTTGAGATGCTTCTCTCGCCGAAACAGCACCGTGCTAAATTTGCCTTCGTGGGAATCAATACAGTAGTTTTTCTAGACTTTACAGTTGTACTCTCACCAAAACGAGCATGGATCTGTTTATCAGAGGATCTCGAGTTCACTGAATTTCAAACTGAAGCAACATCCAAAGGAATAGTGCTTGTTAACGGGCGTTTTGAAGGTTCTAAGGCGTTTTTTGACTTTTTCTTAATTCCTGCCTTTAGTATCAACTTCTAACTTAATGTGTGTTTTCAATTACGGCAGTGGTCAAAAGCAAACACTTTGTGATGACGTGGGTGGGGCAGAGAGAGAGAGAATTACTTAAACTATGTGGGCTCTGTTTTCTCTTTCTGAGCCTGGA
MSTRG.80654.2	0	0.29	5.23E-05	5.759	up	CCAGAGCTGGCTGCCCTGACTGAAAAAACGGTGGTAGAAATAAATCATTGCCACGGAGTGCAGTTATTATGGTGGAAAGTTCATTCTTGCAGGTTGTGACCGCGGACTGCGGGCTTTTATTGAGGTGAGGAGATTCTGCTAGGCTCGCTGATCTGCCCGTCCGACAGAGCACGGGGGAATCCCTGGTGCGGCGCTTTCTCGGGCTGAGGGGCCTTCCCGGCCGGTCCCGTGAGCTGGGCGGCTGCGGGCGGCTCTGGCACGGTTGCAGCCTCCCTCTCGCCAGTGACTCACTCACAAGGGGCGGGGGAGGGGGGCTTCCTGGAAGCCGTGACGAGGAGGCTCGACCCGGACGCCCGGGTTCCTCTCTGTAACCAGGGTTTTGGTCGCCTGTAAACAGAGGCTTGAGGACTACGGGAAAAGGAGGGAAAAGGGAGATGCCCGGCACCCGCCGCCCAGTTGGGCTCCAAATCCCTGTGCTCGTAGTCGGACTAAGGGGCGGGAGAACGAGAGATGGGAGGCTTTCTGGGGTTCCTAGGGGCCGGCTCGGCTCTGGCCGCTGTCCTGGAGGAGGGAATACCAGTTTCGATTCCCTGGAGACGGGGAGGAGTTCCCATCCCTGACTCAGGCTGACCCCCTCCCCTCTTCCATATGCTGAGTCATCGGCTCGAGGGCCAGGGCGCTTCGGATTGGGAGATTCTCAAGAGAGACTGGAAGGGGCTTTGCGCGCCGAAGGACTGAGCACCCGTCTAAACACTAACAGGGGCGGGGGTACTAAGAGCCCCGACCTCTGCCCCTCCTTGCTTAGATCAAGGGGTTCGGGTTTCCAAGCCGCCTCCTCGCTCAGACCCAGGAGACCCGGTTCCCAGGCCGTGGCTTCACCAGACTCAGGAGTCCTGGTTCCCAGCCACCCTGTCTCTCAAACGCAGGAGTCCGAGCCCCCCCGCCCCGCCCCGCCCCCAACCCCTCTCTCCGATTGAGGAGCCAGGCTCCCATCCCCCTCTCCACCCCGTCCAGGCCTCAGGAGTCCCAGCTCAGGAGGAAGTTCAGGGC

MSTRG.9283.3	0	0.19	4.95E-05	5.681	up	GGAGCGGGCCGGGTTCCGCGAGCCGGCAGCCCTGCGTGGCTGACAGGCCCGGCTTCCCCGCTTGGCCGCCAGAGTCCGTGCGGGCGACGCGGTTGCCCTCTCCGGGCCAGAGAGTTGGGGACGCCCCTTCCCCGCCCCCGGCGCCCCTAGCGCCCCCAGCGCCCCCGGGGTCGGCGCCAGAGACCTGGCGGCCCGGAGGCTCTGACTTGAGGCGGCGAGGCGGGCTCTGGGGCCTCCCCGAGCCAGGCCGAGTTTAGTCTGAGGAAGCGACGGCGGGGGACGCGGGTCAGGGCCAAGGAATCGGGGACCCCAACCGGACAGGCGCTTTCTTCCCCTCCCCCTCCCTCCGCCCCAGTCCCAGCCCCTGCCCAAGTTGCCTGTATCCGGTAGGACTGACCGTCTGCCCGGCCTTACTCTGCTGCCAACCTTGCCCGAAATCTAGACCCCAGATCCGGATCTTCCTGATGTCCCATTGTCTTTTCTTTCTTTCCATGTACAGCTCTCCAACTGCACAATGAACTCCTAAGGGCACGGATTGAATTTCATTCTTTTTGTTCCCTGGACTGTATGAACTGAAGTAGCAAGTATATTCATTGGAATAATCAAGATACATGAAAAAATAGTGGAAATATAGTGATTTTGCAGAGAAGGCAATGGCACCCCACTCCAGTACTCTTGTCTGGAAAATCTCATGGATGGAGGAGCCTGGTAGGCTGCAGTCCGTGGGGTCACTAAGAGTCGGACACGACTGAGTGACTTCACTTTCACTCTTCACTTTCATGCATTGGAGAAGGAAATGGCAACCCACTCCAGTGTTCTTGCCTGGAGAATCCCAGGGACGGGGGAGCCTGGTGGGCTGCCGTCTATGAGGTCGCACAGAGTTGGACACGACTGAAATGACTTAGCAGCAGCAGCAGCAGCATAGTGATTTTGAAAAATCTTCATTGAATTTAAAAAATACTTTAAAAATCTTCAAAGCTACAGAAAACAGTATGGAAATAATGTAACAAACATGCATGTAGCCAGCCTACTGATGTAACAGTTGTTGAC
MSTRG.168210.6	0.01	0.43	4.48E-05	5.599	up	GAAAACTGACGTTTTTCCAGTCTTGTGGCCACTGCTGAGTTTTCCAAATTTGTTGGCATATTGAGTGCAGCACTTTCACAGCATCTTCTTTTAGGATTTGAAATAGCTCAGCTGGAATTCCATCACCTCCACTAGCTTTGTTTGTACTGATGCTTCCTAAGGCCCAGATGACTTCACACTACAGGATGTCTGACTGTTGTTGCTTAGGTCCTTTTATTACCATGCTGTGAAGAAGCCTAAAGTAGCCTACTGTAGATGAAAGTCTATCCATGATGGGAGAGTAGAGGTTACCCACTGACAACCAGAACCATGTTCCAGAGGCCATCTGGGACCTACAGATTCAGAGTACATCATGCAGAATACCAGGCTGGATAAATCACAAGCTGGAATCAAGTTTGCCGGGAGATATATCAATAACCTCGGATATTCAGATGACACCACCTTTATGGCAGAAAGTGAAGAGGAACTAAAGAGCCTCTTGATGAAAGTGAAAGAGGAGAGTGAAAAAGTTGGCTTAAAACTCAACATTCAGAAACTAAGTTCATGGCATCCGGTCCCATCACTTCATTGCAAATAGACGGGGAAATAATGGAAACTGTGAGAGACTTTGTTTTGGGGGGCTCCAAAGTCACTGCAGATGGTGATTGCAGTTATGAAATTAAAGGATGCTTGCTCCTTGGAAGAAAAGCAATAACCAACCTAGACAGCATATTAAAAAGCAGAGACATTACTTTGCCAACAAAAGTCCATCTAGTTAAAGTTATGGTTTTTCCAGTAGTCATGTATGGATGTGAGAGTTGAACCATGAAGAAATCTGAGCGCCGAAGAATTGGTGCTTTTGAACTGCGGTGTTGGAGAGGACTCCTGAGAGTCCCTTGGACTGCAAGGAGATTCAACCAGTCCATCCTAAAGGAAATAAGTCCTGAATATTCATTGGGAGGACTGATGCCAAAGCTGAAGCTCCAAATTTTTGGCCACCCGATGAAAACAACTGACTCATTGGAAAAGACCGTGATGCTGGGAAAGATTGAAGGCAGGAGGAGAAGGGGA

MSTRG.135318.19	0.01	0.22	0.00035	5.438	up	CAGTCGTGTCCAACTTGTCACAACCCCATGGACTGCAGCCCATCAGGCTCCTCCGTCCATGGGATTTTCCAGGCAGGAGCACTGGAGTGGGGTGCCATTGCCTTCTCCCCTCTGTCCATGGGATTTTCCAGGCAGGAGTACTGGAGTGGGGTGCCATTGCCTTCTCCCCTCCGTCCATGGGATTTTCCAGGCAGGAGTACTGGAGTGGGGTGCCATTGCCTTCTCCGGGTCATACCCCTAGGCCAACTGAAATCAAAGCTTTGGGTGGGTTGCCATTTCCTTCTCCAATGCATGAAAGTGAAAAGTGAAAGTGAAGTCACTCAGTCGTGTCCGACTCTTTGCGACCCCATGGACTGCAGCCTACCAGGCTCCTCCGTCCATGGGATTTTCCAGGCAAGAGTACTGGAGTGGGGTGCCATTGCTTTCTCCATCTGGCAGGTGGGTTCTTTACCACTAGCGCCACCCAGGAAGCCCAAAACTTCAACTACCATCATCCACAAGAAATTCTGATTTAGCAATTGAGAATTGGTAATGTAAATCAAACACCTTCTTTGGGAATCTGATTTAATATTTTTGTATTGAAAAATGTGTAGTTTATATGCATTGAAGTGATATGCTTAGAGATATTACTTTTACAGATTTTCTGTGATCTTTCACTGTGGAAGTGCATAGATTACATGTTATTTTCTAAAAATGGCAAGTATCTGTTCAGTGTTCTTTGTTGGCTATGCTAAGTTAAACTTTTAAGTGAAACCATCCAGCTGTTTTAATTTTTATTTTGCAGAGTGCTTGTTAATGATAACAGCTTTTATTAAGTCCTTATATATGGCGTTGCCCTGATCCCTCTCCTCTTATTAGTTCCTTCTTTCTTTTTACAAGCATAGCTACAATTTAGCTTTATCTTGGTCCACCACTGAGCTTTTATTGTTTAGGGCTAGAGTCTGCCATGTATTGATACTCTCAAGGCTCTCTTTTTTCTGTCTACCATCAATAGTGTTGATTTTGTTTCTCTCACAGGTTTAAAAAAAATTTTTTTTTAAGAAGGAAAAA
MSTRG.133391.2	0.22	10.9	0.00505	5.415	up	CGACGTCGGGGCGTTGGCCCGTGGGGCGTGCCGTGCCCCTCGCCCGCCTCACCCTTTCCCAGGTACCTAGCGCGTCCCGGCGCGGAGGTTTAAAGACCCCTGGGGGGGGGGGGTGTCGCCCGTCCGCCTTGGGGTTCGGGGCGGTCGGGCCCGCGGGGAGTAGTCGGGAGCTGCCTCCCGTCTCCCCCCAGACCCCGCCGTCCCCCGAGGGGACGGGGTGGCGCGCGGCGTGGCGCGCCACGGTCACTGCCGCCGCGGCCGTCGGGAGGGGGCTACCCGACGGCCGTCGTGTGGGCCGTGGCCGTGTGCGGCGGCGCGTGCGCGCGCTGCCCCGCGTCCCTGGGGGAGGGTTGGGGGCCCCCCGGGCGCCTGTGGGGTTGTCCGAGCCCGCCCTGGCGGCTTGGCGCCGGACGCCCCGTCGTGTGGAACCTTTCCCGACCCCTCCGTTCTGCTGTTTTCTGTCTGACTTGGCCGGCCAGAGGCAACCCCCTGCCCCAACTCCCACCTCCGGGGAGGGAGGCGGGGGTGGGTGGGATGTGCC
MSTRG.157991.6	0	0.16	0.00275	4.495	up	AATCGCTCGTGCTACGAGGGCACTGGGAACGCGCCCGTCCTACCCCACCCTGTCCAGGATGGAAGGAAACGAAGAAAGCCACGAGAAAAACTGCCCTGCCCTCCCTCGTCTCTGCTCTAGCAGGAGGGGACCAGGAGATGGCAAGGCTCGGCTGGGCGCTTACCGAGCGCCACACCTCCCTACTCCCTTTCCAAAGATGAGTTCGCCTGGGGAGCCCCCAGTCCCAAAGACAAACGATTTCTTCTTGAAAATCTTAGCCAGAAACTTTCCCCTCTGTCACCAGTGCCACAAGCGAGTCGTTTGGCTGTCGGACGTGACCACTGATTCAAGAGTCTCTCCGGCATCCCCTCTCCTGCCCTATGTTCTGCCCCCACCGCCCCCTCTCCAGGCCCTCCTCAGTTTCCCCTAGAGACCCTCTAGGGCCCTGCTGAGCTCGGCCCTCCCTTCGGCGACTTCTTGAGATGACTTCTGCAGCGCTGTGTACCCAGGTCTGCGCTCGGCGATTCGGACTGGAGACGCCTGCGGGCGGATACCTGGAGGCAAGGCAGGGAGGATGGTTCCCCCGGGAGGGTGCTTGCTCACTAGGGGATGAGAGATCAATAAAAGAAGCCGATTGAGG

MSTRG.40315.3	0.01	0.18	0.04463	4.487	up	GTTACTTTCCACAGTAGATACTATATTTAATTCAGAGCTTGTGAGAGCCTGGAAACATTGCAGTAGATTGAATGACTTTTAACTGGGAAATCGAAGGTCCGTGTTCTTTGGAAGCAAGGGTCATAGTCAAGGTGGAAATGAGGACACAGTTGGCAGGCGTCAAGGCCACCCCAGCGCACAAATGACCTGACAGTCCCTGGGGACTTCAGAGAGCCACCCTGATGTTTCTCCTAACCATAGATTTTAACCTGGTTTTCCTTCTTTTTTACCTGAGAAATTGTGATTGGCTGTCAACACCCCCATCCTGCATCCCCCAGCATCATTCTTGTTTGGTTGGTTGGTTTTGTTTTACTGAGATAGTCACCCTTCTAAAGTGTACAGTTCAGTGGTTTTTAATATATTCACAAAGTTGTGCAGCCGTCGTCACTGTGCAGCCATCACCTCCAGAAAGAAACCCCATGCCTCACCTGTGCTCGTCACCACCTAGCACTCCTCCCAGCCTGTGAGCCCCGGCAGTCACCTGTCTGTGGATGTGCCTCTTCTGTACATATGACATAAATGCATTCATGCAACATGTAAACTTCTGTGTCTGGCTTCTTTCGCTCAGTGTAGTGTTTTCTGGGGAAGATTGAGGGCAGAAGGAGAAGGCCTCGGAAGAGGATGAGTTAGGCGGTTGGCATCACCAACTCACTGGACGTGAGTTTGAGCAAACTCTGGAAGATGGTGAAGGACAGGGGAGCCTGGTGTGCTGCAGTCCATGGGGTCACAGAGAGTCTGACATGATTTAAGTGACTGTACAACGTTTTCCAAGGGTCACCATATTATCGTCTTCCTCAGTATTTCGTTCCTTTTGTGGCTGGGTAGTATTCCATTGTATAGACAGACTACAGTTTATTTGTTCATCAGTTGATGGACATTTAGATTGTTTTGACTGACATGAATAGGGCTGCGGTGAACATTCACATACAAGTGTTTGTGCGAACATGTATTTTTAGTCCTCTTGGACTAAAAGGAGTAGAATTGCTGGGTCCTACGGTAATTCTGTGTTTA
MSTRG.174668.3	0.01	0.12	0.0257	4.303	up	CATTGCTCCAAAATAGCTTGTTTAATATTTGCAGAGTGGTTCAGGTCTTTTCTCTCTCCTGCCTCTCAAAAAGATGCTATTCCTGTGATAAAACCATTACAGTCCTTCCAAGGTTATACATGACTTTCAGGCTTCAGCCTCCATAGCAGCAGTCTGCCCAGGAGACCAGTACAAGGACCAGAACTGAGGATGAGCAAGGAATCTCACCAGACAGAAGGTGTCTGAATAAATACACGGATGCTCTGTATACCTAGTCAAAACTACAGCTCACCTATAGGGAGCGGTATCTACTATATCTACGGGGTGGCTAGCAGATTATGTGGACCAAACTGGACTTCACCAATAAGTAGTCATTTTAAGACTTCTTGAAATGGGGTTTCTCAGAATTACTTATTGTATGCCACTGCCTTGGCTGTGAGAGACAGGAAGAAAAAGAAGAGACCATCGCTGAGAAACGTCTCGAGACCACCTGTGGCCAGTGCTGAATGGGGTACATCCGTGGCCACAAGGATGCTGTTTTGTAGTACCTGAGGACTCACTCACGTCCCTGTGCACTATGGTTCCTAATGCTTGACTTTCCCTCTTCTTTCATTCCTACTAACCCAGATGTGTCAACTTTACACTCCAGATAACCAGTTGAATATGCTGTCCTCATATCAATAATCTATACAAACCCAAGAAGCTGTGAGATGAATAATACACAGCAAACAATACAACAGAGTAATCTCTTTTTAGCACTGTATCTACAGAATGTCATTTCTCTAAGCCATATGAGCAGAAACTGGCTTTTTATACAATTAATGGTCCAGAACCAGATGAGAATTTCTAACTAAAAATGCTGGAACATTTTCTATCTTCTCTCAACCATAACTCTCACTGAAATCTATTGCACTGGTGATATATACTGTGGATCTGAACAACACAGAGTTCCACAGGCTCAGAGACTGATACACAGGACACACAAAGTCGTGAATTACTCAGAGAAATCCGAGGGAAAGAATTGCTCCCAAGGAAGTATAATTTAAAATCTTTCATTATAACCTATCAAAA

MSTRG.196420.13	0.02	0.3	0.03121	4.253	up	CTCGTGTCCTTCGGTTCTCGTGATCTGGCCGTTTCGTCCGTT<br>TTTCCAGCGAGAGGCGCCGGTGAAAAAATTTTTTGTGTGTGC<br>CGGTGAAATTTAATCAAGGAGTCCCTTTCTGCCGGCCTTGTA<br>CAAGAGAATTTTACTTATACAAGAGACTTTTGCTTTCGTGAG<br>CGGCATTGAGACAGAAAAGCCTGGCGGGCTACAGTCCATGGG<br>GTCACAAAGAATTGGACACGACTGAGCGACTAATACACAAGG<br>CTGAATAATATTCAATTCCTGTGGGATCTTAGTTCCCTGACC<br>TGGAGTCCAACCCACGCCCTCTGGAAGCGTGGAGTCTTAACC<br>ACCGGATTGCCAGGAAAGTCCCTCAATATGGTTCATGTTTGC<br>ATTTCCCTAATGATTAGTGATGTCAAGCATCTTTTTGTGTGC<br>TTACTGGCCATTTATGTATTTTCTTTGGAGAAGTATTCATTG<br>AAGGCTTTTTCCTCACAGTCATTTTTAACACGTACATATATT<br>TTATAAGCTTCCCAGTGGTAAAGAATACACCTGCAGTGCAGG<br>AGTTGCAGGTTTGCAATGCAGGAGACTTGGGTTTGATCCCTG<br>GATTGGGAAGGTCCCCTGGAGGAGGGAATGGCAACCCACTCC<br>AGTATTCTTGTCTGGAGAATTCCATGGACAGAGGAGCCTGGT<br>GGGCTATAGTCCTTGGGGTCCAAGGAATTGGACGTGGCTGAG<br>CCACTGAGCATATACATATATTATAGATTATGTAAACGTATT<br>GAATACTTCAAGATCTATGTCAGTTTTTTGGTTTTAAAGGCC<br>ATAATCATATCCCACATACAAATATCTGGTCATTCTTTAGCC<br>CCTTGCTATACTCTAATGGATATTTTGGTTTGTTCCAGTTTT<br>TACCCATTATAAACAGTTTGTACAATAGTAAACAAGCTTGAA<br>TACACACACACATCTATTTATCTGTCTATATCTGCACAGTTG<br>CCAAGGTTTCTCTTAGCTACAGTCCTGCAAGTTGCTAGTTGA<br>AGGCATGGGGATATTTAAAATTTTTATACACATAAATTCGAA
MSTRG.67057.2	0.15	2.42	0.00541	4.179	up	GCCATCTCGGTCTTTCGTGGCTGCGAGCCGGGTGTTCTCCGT<br>TTTTGTATGCTGCTAAGAAACGCGGCAGGCATGACTCCAGTG<br>TGGATTTTGATGGCTTCTGGTGGGCAGTTGTGTACCAGAAGG<br>ATGACAACTACTGCTGTGGTCTTAAAGCTCAAGCTTGATGCT<br>CTATCTATGAGCAGACAGCCATTAAGCAGACTTGACTGGAAG<br>TTGAAGAGGCATTTTAAAAATTTGTGGGGCGAAAATTTGTCT<br>TTGGCTATAGAAAACCACTCTTTTATTTCTTAGGTGAACTTG<br>AATCCTTGACCTGTCAAAAGCAGTTGGCCTTTTTGATGATGG<br>CTACATGTTTTTTTCTATGGATGACTTCTTTGGGGTGTGAAG<br>AGAGGTAGAGACTACGTCCATGCAAGGAACCAAGAGTTCTAA<br>CCAAAGGCAGATGTTTTGTTGTCCTGAAGATTTCAAGGGCAT<br>TTATGATACCAGTCAGATAGAACAGGCAGCCTGATGGATGCA<br>CATGGGATAGAAGAGCAAACAGCTATTAATTGACTGCTGCTC<br>TGCACAGCTTTTGGGATCCTAGCTTCCTGACAAGGGATCAAA<br>CCTGTGACTGCTGCAGTGGAAGCTCAGCGCTAACCATTGGAC<br>CCCAGGAAATTCTCCAGCTTTCTATTTTAATAAATGTTTATA

MSTRG.231934.33	0.03	0.48	0.0205	3.769	up	TGGATTTAACGCGAGGGGGAGAGGGGCGCATGCGTATTTCAA<br>GCGAAATGACGCGAGGAGGCGCGCAGCGTACGCGTAATTAAG<br>ATTGCAAAATGGCGAGGGGGGAGGTGACACCAAACTTGATCA<br>GAAACCATGCCACAGGTGAAGAGGTATATTGAGCCCTGCCTG<br>GCCAGTGAGGTTACTAGAGAATTGGAAGAGGCTCAAGTCAGC<br>ATCCTGCACATGGTTATCAAACCATTGTGAAGTCTTATCACC<br>TGGGATACTTGTTAAAAATGCAGATTCTTGGGCAGCTAGAAA<br>CCTTGTCACTGGTCAAGAGCAACAAGTAACAAGACCTACTTG<br>TCACTTATCCTGAGGCATCGTGCCCAGTGGGTTTACCAGCAA<br>ACCAGAATAAAGCATCATTGCCCTGGATGTCCTTGTGAGATG<br>GTTGGGAAGTCTTGGCTGAACTGTGGAGAATATCTTTGAAAA<br>GTGTTTTAGAGAGTCTACCAGATTGGCCATGTGAGTGCCTGT<br>GCAGCAGAAGAGTGGGTCTTCTCAGTGGTTAAGGTAACAAAG<br>TGATTAAGGTAAGTGAAGGAGGAAAGATTGACATTGTCTTGA<br>CTCCTATTAGGGAGGCTTGTAGGAATGTCATTTCTAGGGGCC<br>AGTAATTTTAAAGCCTGTAGTTTATTCTACAATATATCCTTC<br>TCAAAACAGTTGGGGTAGGTTATATGCTTACCCAGTCTGAGA<br>TCACCCCTGCAGAGGATCAGAAGAATTGAAATGGTTCCTAAG<br>TAAACTAGGTGGCATAAGCTGCATGTCATAAATAATCTCAGC<br>ATGCACTGATTACAGTCCCTTTGGTGAGAGTCTATTCTTTCA<br>AACCAGACTTATTGAGAGTTTTTCTTTATCAGCCTATGGAGC<br>TGCATACATCTTGTCAAAAGCCAGCTCATTAGCAGAAGCTTA<br>TTATTTAAGCAGCAGACAAGAATATAACACTTTGAATTAGTA<br>ATTTCAGAGGTAGCAGCATCTTTTAGTATAATCCATTTAAAC<br>TGGACTAGCATTCATTTTTGGCTTCTCAGGCATTGACCCCCT
MSTRG.216124.1	0.01	0.17	0.00535	3.602	up	TTTTGTTTCCAGGAGCCCTCAAGTAGCCAGGCCCTCCAGCTC<br>TGGAATATCCTCTACTTGCGGTTCCCTTTGTATTAGCTATAG<br>TATTTGGTATTTCAGAGAAACCCTCCTTCCCACTTTATACCA<br>GCAGGCAGAGGAACCCATTGATGGGGCTCGGAAGTTGCCTGA<br>AGGTATTTCATGATGTCCTAATAATCCGGAGCTGCCTGTTTA<br>AGAATTTGTGAACTGCGATGGTCCTTTCCAAAAGAAGATTTG<br>CTTAGGAAAATGCTAGTGTAAACAAGATCTTCAGGGGAAGAA<br>CTGTAAGCCTGAATGCCCAGGCAGCAGTGGACATGAGTTCCT<br>ACTGGCCAGCTGTCAGCAGTAAAAGGGGCAGGAACCCGGAGA<br>GATCCCAGCAGGTGATGGAAGACCGCCTGTCAAGGACAGCAA<br>GCCTTCTGTGGTCTCTGGTCAGGGATACTGGAGGCCTATTCG<br>CTCAGCCTGGAGATGTAGCCATCAGCAAAGGCAAGCATCGGT<br>GAGCTGTGTATGCCCCCAGCTTAGCACTCAGCATGCTCCACC<br>CCAGGGTACGTGTGGCCCAGAGTAGCAAGGGGTCCCATTCTC<br>TCATTCTTTTTGGTCATTTATCCATCCATCCCAGAAGTGTTT<br>ATTGAGTGCCTGATGTAGGTCGAGCACTGTACTAGATACCAG<br>AGTGAATTAGACCACAGGACCCCTGCCCTGTGGGGTTCACTA<br>GTAAACAAACAGGCAGGTTTCTAGTATATAGGATAAGTGCTC<br>TGATGGTGGAAATCCAGGGGTTGGTAGAAGGCACCTCTTGTC

MSTRG.130001.2	0.08	0.81	0.01101	3.508	up	TTCCCGGTGACGTGCTCAGGACTCAGCAGACGGATTTCCTGCGGGTCCGGCTGCATCTATTCGAGGGGCGCCAGGAGTCCCTTCTGTTCTCCTCGGGCTCGGAAGTAGAAAGGATGGTGTCCGTGTGGAGGCGCTGTGTTTCGTGGGTGTGAGACGCGGATACGTCTGTGTGAGGATCACAAAGCCGACAGATTGGTGGCCTCTGTGGCGAGGTCGACCAGATGGCCCGACAGGTTGTTGGGGATTTTTGTTACTATTTGTATTGTACTGTAGGTATTCAGTGAGAAATCTCGAAATTAATTATAATGAAAATCACCATTATCATTGTTACTCAACAATAACGTAATTATTACCTTTGTTTGCAGTTAATTTAAGAAACACTGTTTCAGGTTTATCCACAGAGTATCCAATTGAAGACGACCAGAGAAGGATGTTGAAGTGAAGAACTTTCTTAACACACTGGGAAGTTGGGGAACCTCTCCTTCTAGCCACGAGGCCCTGCCCTGCAGCAACCGACCTGGAAAACCAGACCCCAGCCCCTGCAGCAATGGGCTCCAGGATTCAGTGCGTGGCCCAGAACTGACGATTACACAACTCAGTTTAAACTCTGTGAAAGAAAGTAAAGTTGCTTGGTCATGCCTGACTCTGCAACCCCATGGACTGTAGCCTACCAGGCTCCTTTGTCCATGGGATTTTCCAGGCAAGAGTACTGGAGTGGGTTGCTGTTTCCTTCTCCAGGGGATCTTCCCAAGCCAGGGATCCAGCCCGGGTCTCCCACATTGTAGGCAGATGCTTTCCCATCTGAGCCACTTAAACTCTGTACTAGGGATCATATAACAACAATGTATCCTGCTTGAGGACAGTTTCTCCTTCCTGAAAACCTTCTGATTAATCCTGATATCTTAGAATGTATATTATGGGAATAGGTCTGGTAGGATCTTTCTGTTAAATTCTAATCCTCTTATCCTAAAATGCTAATTGTGGGAGTGGGTCTGGTAAACCTTTCACAAACTTGAGACATTCTTTTGATTTAATGTAGAAACTAATTTA
MSTRG.138781.2	0.01	0.18	0.00267	3.466	up	AACATTTGGGTGCATTTCCTTCCACCTCGCTCCACGTGAAGATAAATGTTTGAACAGTTGGGATTGCACCAGGCGCCAGTGGGAGGGGTCCGGCCACCGTGAGCCTGCGGCAAGCGGCGGCACCATCGCCTACATGGAGGCGCTGTCCTGGCTGGGTTGCTGCCCCCAGAGCCGGGGCGTGAGGACCGCCTGCACCCCAGGCTGTGGGATCTGACACAGTCATCGTGTGGGTGACCTGCCTCCAGGCCAGGGTGTGTGAAACTCGAGCCCGTGGTGGGGCGTCTGTCCAGGGCGTGGGCTGGAGGCTCAGGCAGCACCAGCCAGAGTGTGGAAGCGCACAGGCTGCCCCTCCCCGACCTTGCTCTGGGGCCCCTTGTGGACAATGGGTTTGTTTCCTTTCTAAGGTGGACTTTGCATTTTAAAATTTCTTTCAGTGTATACAAATTCACAAAAACTTTTCCTGCTACAAAAATTTTGTTGAAAAGTAAGGCTTTCTGAGAGTGAAAATCCTCACGAGCTCTCCAGGGGCCTGGGAGTCGTGTCTGCCCACGGGCGCTGCACTGGCCCTGGCCTCCACCGCGAGTAAGGCAAGAGGGTATGCGGTAATGTAAACGCTGAGGCTGCCGCCCCGATGCCCGGCCCGGCCCCACGGGCAGCGGCCTCTGTTAGTCGGCGGCTTTAGTGAGGGTGCCCCAACACGAAGCCCCCACACCCTTCAAATGCACGACCCTGCTCTTGTCCTTCCAGGCCCCCGGGCAGGCAGACAGCCTGGCACAGCCCTCCCTCCTAGCTCTGAACTTCCTCAGTTCCCCCTTACAGGCAGGAGTTCATGGGCTGCACTAGGCAGGGTCCTGGCAGAAGTGAGGTTCGGGGCTGGGAAAGGAGGCACCAGCTGGCCTGCTGCCCCCTAGGGACACCCCTCACCCCAGGGTGGTCAGAGAGAGTCTCCCTTCAAGACCAGGAGTCCTGGTGCTGGGGGAGCAGAGCCCAGAGACGGGCCTCAGGCAGCACCTCCTATAGACAGATGGGGGCTGCTCTCTTTGATGGGAA

MSTRG.141898.2	0.03	0.39	0.00185	3.427	up	GCTGCTTTAACCCCTTCCTGCCCTCCCGCCGCCTCAGCCCTC<br>AGCTGCTCTGGGAAGTCCAGCTGGGGGGATGGGGCGCGTGGG<br>GAAGAGTGGTAAAGAGGAAGCTGAAGTTTTTGAAAAACACTC<br>GCTGAAGACCCAGGTTCATGGATGGAAGCGCCAGTTATCTGT<br>GCGCCACAGGCAAAGCAGCCTCCCTTGGATAGCATGACAGGC<br>CTAAAGACCTCACCACTTGGCTCCCAAACAACCTTTTCTGGG<br>AACAGAATGCTGCTGCCTGTGTGATGCTGAATGGTGGAAAGA<br>AAGCGGGAAGCAGCCCAGATGTAGCGGGAGCTCCGTGGCACT<br>CGCTTTCTCCCCATTCCGTCCCTGGACACGCTCCAACCCCAT<br>CCGCCAGCTTTTGGGGGCCCCGGTCCGCGGCCCTGCCCCCTT<br>CCCTCTGCTGCGGTTGTAACAGGTTCATGGCTGTCGCCCAGC<br>CCTCGCCAACCCAGACAGCCAGGCGCGGAGGGAGGGAGCCCC<br>GGCAGGGCCCCACCGGCGGGCTCCCAGGCCCCTTTTAAGCGC<br>CCCCCGTCCCGTGCCCGGCGCCCCCCAGCCCCGATCCCCGGC<br>GCCTCGCCCACCCGCAGCGGCCCCGCTACCTCGGGAGGCGGC<br>GCGGGTCTCAGTCCCGGAGGCGGATCGCAGGCGCAGGCAGCC<br>CCACGGCCATGACTCCGCCGCCGCCTCGGTCTGGCTTGGCAG<br>ACCGGTTGACGGATTAGCATCTATTTTTAAGCCATGACACGC
MSTRG.133381.13	0.08	0.84	0.00699	3.398	up	GTAGCATATGCTTGTCTCAAAGATTAAGCCATGCATGTCTAA<br>GTACGCCCGGCCGGTACAGTGAAACTGCGAATGGCTCATTAA<br>ATCAGTTATGGTTCCTTTGGTCGCTCGCTCCTCTCCTACTTG<br>GATAACTGTGGTAATTCTAGAGCTAATACATGCCGACGGGCG<br>CTGACCCCCTTCGCGGGGGGGATGCGTGCATTTATCAGATCA<br>AAACCAACCCGGTCAGCCTCCTCCCGGCCCCGGCCGGGGGGC<br>GGGCGCCGGCGGCTTTGGTGACTCTAGATAACCTCGGGCCGA<br>TCGCACGCCCCCCGTGGCGGCGACGACCCATTCGAACGTCTG<br>CCCTATCAACTTTCGATGGTAGTCGCTGTGCCTACCATGGTG<br>ACCACGGGTGACGGGGAATCAGGGTTCGATTCCGGAGAGGGA<br>GCCTGAGAAACGGCTACCACATCCAAGGAAGGCAGCAGGCGC<br>GCAAATTACCCACTCCCGACCCGGGGAGGTAGTGACGAAAAA<br>TAACAATACAGGACTCTTTCGAGGCCCTGTAATTGGAATGAG<br>TCCACTTTAAATCCTTCCGCGAGGATCCATTGGAGGGCAAGT<br>CTGGTGCCAGCAGCCGCGGTAATTCCAGCTCCAATAGCGTAT<br>ATTAAAGTTGCTGCAGTTAAAAAGCTCGTAGTTGGATCTTGG<br>GAGCGGGCGGGCGGTCCGCCGCGAGGCGAGCCACCGCCCGTC<br>CCCGCCCCTTGCCTCTCGGCGCCCCCTCGATGCTCTTAGCTG<br>AGTGTCCCGCGGGGCCCGAAGCGTTTACTTTGAAAAAATTAG<br>AGTGTTCAAAGCAGGCCCGAGCCGCCTGGATACCGCAGCTAG<br>GAATAATGGAATAGGACCGCGGTTCTATTTTGTTGGTTTTCG

MSTRG.110124.3	0.01	0.14	0.01903	3.346	up	GGAGCAGTTGCGAAGTGAGGTTGGCCTGCGCGGGTGGAAGCA<br>AACGTGACGTCAGCTTGTGGCCTTTGCTGCTAAGTCACTTCA<br>GTCATGTCCGACTCTGTGTGACCCCATAGACGGCAGCCCACC<br>AGGCTCCCCCGTCCCTGGGATTCTCCAGGCAAGAATACTGGA<br>GTGGGTTGCCATTTCCTTCTCCAGCGCATGAAAGTGGAAAGT<br>GAAAGTGAAGTCGCTCAGTCGTGTCCGACCCTTAGCGACCCC<br>ATGGACTGCAGCCTACCAGGCTCCTCCGTTCATGGGATTTTC<br>CAGGCAAGAGTACTGGAGTAGGGTGCCATTGCCTTCTCCTTG<br>TGGCGTTTGGTAAAGGCCAAACTTTACCAAAAGTTTGTCACT<br>TCTGTAAAGGCAGAGCCACCTGTGTGAGGAGGACATCAGCAA<br>GCAGAGCTGAGAGTTCAGGAGAGATGGTTCCGGCTGACGTCC<br>TAAAACTCCCGAGTGCAGCCCTGGCGGACGCTTGTGGACTTA<br>GGGACCTGTCCACCTGTGGATGGCTACAGAAAGGAAGAAATT<br>AACACTTCTTGGTCTGCCAGCTCTCCAAGTAAAGTCATATTC<br>TTTGCCTCAAGACCTTGCCTTTGATTTATTGGCCTGTCGTGG<br>GGTAAGCAGAGTGAGCTTGGACTCCATGATAATCCCACATGC<br>CTTGCAGTCAAAAAACCAAAACATAAACAGAAGCAATATTGT<br>AACAAATTCAATAAAGACTTTGAAAATGGATCATATCAAAAA<br>GATCTGAAACCAAAAACTGAAAAGAACTCGGTGGTCGTCTGA<br>AATATTTAGAAAAGAAAATGGTGCCCCTCATGCCAGTACCCC<br>CTGAAGTCACACAGGGGACACAACCTAGGGGAGGTCCCTTAT<br>CTCGTCCAGGGGACTGTCATACCAGCCACCATTTCTCCCTGC<br>CCATGATAAACACACTGTAACGAGGGTCATTGGCACCTGGGC<br>CAGTAATGCTGTGAACACCTGGATTCCTTGTCCTTGTGAGAT<br>TTGATGTGAAGATAACACAGAAAGAGAACCAGCCAAGAAGGA
MSTRG.154624.1	0.02	0.26	0.00726	3.329	up	GTCTCCCGTGGCGTGGAGAGGACTTTTGCTTTGAAGTGCGTG<br>TATCCGCTGTGTGGAAGGTCCAGGAAGGCTCGGGGATGCGGG<br>GAGTGAAGTGAGCATGTCTAAACAGTGTAGGGCATATGAGGT<br>TGAGGTGGGGCGGGCAAAATGCGATGTTATGTATTTGGAAAC<br>CAATATAATTGTAAAGGGGGTGATGTCATGTTGATATTAGTA<br>GAATATACTCCCGATTTGTTGATGCTTATGCGATTTTCCCAA<br>ATGGGGAATTGAGATACTGGGTGAATATGCATCGCGTTTTCC<br>TTTGGTTTTTTCATGCATACAAAAACAGACTTCATTGATTAA<br>TGTTAGCTTTCTATTTTATTTTTATGCAATAAGGTGATTTAT<br>CAGTTATCACATAGGTGTGCAGCAGAAGTTTTGTGCTACTGA<br>GAATGTTTTGTGTATGTAAGCTCCTGAGGTTGTAACTGTAAT<br>CAGATTTGCAGAGGAGCCAAGAGCCGTAATGTGACCAGTTTT<br>TTCATCTGACGGTGAGAGACTGCAAGTAAACCATTTTTCCTA<br>CTGGTTTTCTGTCCTTTAATCTAGGACGTTATTTCTTGAATC<br>GTCAGCTGACTTGCTTTCACATATTACCAAGTTGCTTCGTCC<br>TGGCCTAAACTGATTTAGTGGTGTCTGATTTTTTGCAACCCT<br>ATGAACCATAGCCTACGAGGCTGGTTTGTCCAAGAGATTTCT<br>CAAGCAGGAATACTGGAGTGGATTGTCATTTCTTTCTTCAGA<br>GAATTTTTCTCACCCAGGGGTCAAGCTCGGATCTCTTTAAGT<br>CTTCTGCACTGTTTCCCACCCCAGACGCTGCAGACTGGCCTG<br>CAAAGGGGAAAGTGGAGTGTGTGTGTTTCCTGCTCGCCAGTC<br>ATCTACAAGGCAACAGCCAAGACTTCCACTCACCTGCAGACC<br>CACACAGCCCACCTCACTGCTCCACACAGAACCGCAAAGGGA<br>ACTTTGCCAGAACAAATGCCACTCCAGCAGCTTTGCGATTTG<br>GGGTGAATCGGGTCCTGGGACACTTCAGTAAAGGGTGGGAAC

MSTRG.221949.1	0.05	0.56	0.03399	3.19	up	GTCGGAACTTGGGAGCAGATCATAGCTGCTAGGTTAAGAAATTGATTCTCCCGCAGAAACAATGGATTTCGGTGTAGCGGAACTGTTACGTGGAGATGCTAAGGTAATTGTTGAGAGCTTAGTTTTAAGTTGAAGGAATAAACCTGTTGATTTACAGGTTGATCAACCTGTAAAGATGATGCATCATTTGAAAATTTTTTTTTTTTTAAGATGCAAAGGTACATTTCAAAACACGCCGGACAATATTGGCTGGAAAATGACTTGGTTAAAACCTGATGATCTTACCAGCATTCTGCAGGTTGATGAATACTAATGAAGCTGTGGATGTCACTGAGCAGCTTCATTTTAAATGAGGGGTTGCTGTCTGCCTGCTGTCTGCCTGGTGTGCTGTGACATTTTGAAGGTGGAAACATTTCTGGCTAGTGCTGCGAGATTTACTTGTCTGTCTTATGAAAATCTGGTGATTGGGAAAACCTCCAATGGATGTGGGAAGAAAGTTCAAGATGAATTACATTTTTACATTGGTTTGTAAATAGATTCTGAACCAGCATCGAGTCTAGATAATGCATCCATAAGAGTCAAGGCATTTCTGTTTGACCATGACACTGCTGTAGAATCATAACCTGTTAATCAGTGTATTACTTTCTAACTATTTTGAATGGAAAATTCTTTTCTAGTTATCTAGCCTTGTAATTAAATAATTTTGAATAGCCTTTGGTTCTTTGTCTGAATTGAGTTTAAAACTGTGTGATTGTAATTACACCAAGTTAAAGTGTGTACTCTTGCGGTGGAATCTTCAGTCAGGTTTCTAGGGAGAGATGATTTGGTTTGGCAAGAATAAGTAATGTGCAAAACATTAGCTTCAGAGTGTTGGGGTGAGAGCAAAGTGGGAAAGATGGGACCTGTTAACAGTGAGGGAAGGTCACTGATAAAATCTGGGTTAAAACTGGTCAAGCATTGTATGGAATGGGTTAGTGTCAATAATAGCATCTAATCTAACCAAGGTGGTTGGGCCCTGCTGCTCCTGCTAAGTCACTTCAGTCATGTCC
MSTRG.43629.35	0.06	0.59	0.00234	3.15	up	CTCTGCAAGGACGCCTGGCTCCTAGGTCCGAAGGGAAGGCGAGAGCTGAGCTGAGGCACCAGTCAGGTCTCCCACACTCCCGCAGCCTGCTCCTTCCCGGGCGCGAACGCTAGAAGCTGGGCAGGGCTTGGGGAGGCTCGCCCAGCACAGAGCAGCCTCTCGGCCGGGGCTCTGTGCTATCGCGCCTGGGCGCAGAGCTGGAGCTCCGCCGGTGTGGAAGCTCACTCGGTCCATCCCAGTACAGGCTTGCAAGCACCCGCCGGCAGCCAGCCTCTCTCTCCCGCGTTCCGGCATTGGGCTCCAAACCTGGGATCAAATAACTGCCCGGAGCTCACTGATCATGGGCTGGTTCCCCTGCGCTGCGGCTGTGCTAGGGAGCCCAAACGCCCAGGGGAAGCCCGCACAGCACATCCACCTGCCTCTAGGCGGGAGGACAGCTCCAGGGAAGGCCCGGGCTGGCAGGCGCTTTCTGGGCACAAACGGAGCAACACTCTGGCTGTGGAGCTGCCTCCCCAAGCCAGGGGCACCGGGACCCTGATTCCCTCCGTGCGGCGCTTAGTCTGGGCGGGGAAGCCCCAGCTTGGGAATGCAGCCTGGGGCGCTTCCTTTCCCTCGGAAAGAGCTCTCCCAGCGGGAGGCTTTGGCAGTGTGCACACCCCCAGCCCCTAAGCCCTCTGCAAGGACGCCTGGCTCCTAGGTCCGAAGGGAAGGCGAGAGCTGAGCTGAGGCAGCAGTCAGGCACCACTCACGGCTCCCACACTCCCGCAGCCTGCTCCTTCCCGGGCGCGAACGCTAGAAGCTGGGCAGGCCTTGGGGAGGCTCGCCCAGCACAGAGCAGCCTCTCGGCCGGGGCTCTGTGCTATCGCGCCTGGGCGCAGAGCTGGAGCTCCGCCGGTGTGGAAGCTCACTCGGTCCTTCCCAGTACAGGCTTGCAAGCACCCGCCGGCAGCCAGCCTCTTCTCTCCCGCGTTCCGGGCATTGGGCTCCAAACCTGGGACCAAAGTAACTGCCCGGAGCTCACTGATCATGGGCTGGTTCCCCTGCGCTGCG

MSTRG.202650.1	0.03	0.25	0.025	3.134	up	GCTATAAATGGCATTTACTGTCATAATATGACGAACTTTGAATACTGATCTAACTAAACATACAGTAGAGGCATTTGGGAAGGATGAGAGGATAGGAAAAGTGACTTAGAAGAGAAGGAAAGAGGACTAAATCTTGGTCTTGTGTCCACAGTGAGAAGCCAACCAGATCATGTTTAAAACTGAAAGAATCTGAAGTAACAGTACCAGAAGTTATTGAGAGACATAGAGGCTAAAAACATTCTAAAGTGGTGGGGAAAGGAAGAAGAATGCAAGCTAACTAATGTAAAAGAGAAGACAGAATTAGAAAATCACCTGTTTGTAATCTCCAGTGTAATAGTTAACCACCAATGGATGCCAAAATCATGAGACAAAGGGCTGGGGGACACAGGATATCCACCTGCCACGGTACCTGTCCAGAGTCCTCGCTGGTTGTTTACAGGAAAAGCCGCCCTACAATGGGGCAGTCCTGCGGTTGCCACCACATGATCGGACTTTTAGCATCACCGATCGCAGAGCACTGTGACATTTTGTGTTTCCTAAAGAGGTAAGATACTCAGCATCAGTTGGGAAGTATCTTTGCTAAAAATATCATTAAACTGAACCTGCCATTAGACCTAAATTCTAATTTACCAGACACAGAAGAACAAATGAAATAAAACTATGAATAAACAGAAGAATTCAGTCACCAAAGAACAACAGTGCCCAAGGAAGAGTCAATGTCATGAAAAAAGACGCCTGTGTGGGTGTGGAGAGGGTTCTGTATTTTAAAAGATTAAAGAAACATAATAACCAAATGCAATTTGTATATTCTGACAAGATTTTGGTTGGGAAAAAAGCTGTAAGAGATATTTTTGTGAAGCTGGGAAAATTTGAATATAGACTGAATTTTAGCTGATACTTGGGGATTATTATTGCTTTTCTTAGGTGCAATAATGCAATTTTAGTCTGCTAGAAAAAAATATTTTTAGAGGCTGAAGACTCTGAATGAAGTACTTGGAGGTGACATGA
MSTRG.236907.1	0.01	0.1	0.00161	3.107	up	GCAGAGTGGCCTGTTGAAAGCCAGGGTCATTCCCCTCCATGGTCTTTCCCCGAGCTCATTCACTTGCCTACTAGATACCCAGAATCCTCCGTCATCACAATTGGTTTCACGCGACGGCCTTCCGGGGGCCTGGAGAGCACACGGATGCTGGGATCTCACCATCAAAACATTCTCACGGGGAATAACCTAGAGTGTGGTGTGGGCTGTTGAATTGGGTATGCAAGTCTGAGAACAGCTGGCTGAGAACTCACCCAGACACGGTGAGGAAGTGCAGATTCTGGTATGTTGACTGCCTGGGAGGCTGTGTGCTAGCATGTTCAGTTTCTTTTCACCTGGGAAGCCAGAGAGGATGGTGACAGGTAAATAACTCCCAGTTCCTACTGACTCTGCTGGTCTGCAGAACACATTTCCGAATGGCAGGGACTTGGAGATCCTACCTTGTCCTCTCTCCTGTGGGGTCCCGTAGCGACATTCCTTTTCCCCAGCCTGGGTCTCGGTTCACGACAGTCATACCTCAGTGGAAAACCCATGCTCTCAGTACAAAGATTCCACAAGCTTCGCCTGGCAATCTCCCCACTTATCAATACCAGTCTTCAAACGTACACATCCACCCTTCCGTCTTGTAGCCAGACACCAAAGAAGTCCCCAGAGTGGACTTCTTTGCCACCCCCACAATTATCCCCCTGTCTCAATCATTATCCATAGCCATGGTGAACTAAACTCCTCCAGGCAGGAGTAGACTATATCTCCTTAAAAAGAATGAAGTAATGCCATCTGTAGCAACATGGGTGAACCTGGAGATTGTCCTACTGAGTGAAGTCAGTCAGATACAGAAAGAGAAGTATCATATGCCATGCTTTACATTTGGAATCGAAAAAGAAATGATGATACAAATGAACGAACTTACAAAACAGACATTCCCAGACTTGAGAGAATGATCTTATGGTGGTGACGGGGAAGGATGGGAGGAAGGGATAGTATTGGGAAGGAAACTCTGCTCAATACTCTGTAAATAACCTTATGAGGGAAGTTACCAGGGGGGAAGGATGG

MSTRG.44275.1	0.01	0.16	0.00939	3.089	up	TTTATTTATATATTTGGCTGCTCTGGATCCTAGTTGCGGCAT<br>GCAGGACCTTAGCTGTGGCATGTGGGATCTAGTCCCCAACCA<br>GCGATCAAACCTGGGTGGAGTGCCCTGGGAGCTCTGAGTCCC<br>GGTCACTGGATCACCAGGGAAGTCCCTCTACGTGCTGTTTAT<br>TGGATAAACTGTTGCCTGTTCGTACAATGGCACACAACACAA<br>CAACACAAAGAAAGAGGCTACCACTGAATGAGACAACCAGGA<br>TGTCTTGCAAATATAATCTGTATGAAAGAAGCCAGCCACACA<br>CACACATACTGAATGACTCCACTGAAGTAAAAGACAGGAAGC<br>TAGAAAAGAGGACACAGCCTCTCAAGAAAGTGAGCCTAGAAT<br>TGTCGTCTGACCCAGCATCTCTGCCTCTGGGTATTTGCCGGA<br>AGGAATAAAGGCAGGATCTTGAGGAGATGTTCACAGAGTTCT<br>TCATAACAGCATCACCAAAAGGCAGAAACAACCCAGGGCCAG<br>TGGCACCCTCTGCTTTCTGTTTAGGGGGACGTCGAGCCCAGG<br>CCTGGAGGAGCCGAGGCCTTGATGATTTGAGGCAAGGCTCAG<br>ATCGGCTCACTCGGCAGCTGAGTCCAGAGGACATGGTCAAGG<br>ACACCCCCTCCCAAGAGGATCCGAGGGGACACCTGAGGCCTC<br>AGGTGCCCAGATACACAGGGATGGGAGAAGCAAGCCTGCTGG<br>GGGATCCTGTAACTTCCTTTTCTGCTGTAACGAAGACGGGGA<br>CTACCAGGCATGGAAGAACCACACCTGCCTGGACACACAGCC<br>AAGAGCGAGAATCTGGCTCAGAGGCAGTGCTCCTAAAGTCAC<br>CAGCAAGGTCACCTTCCTCCATGAAGCTGATTCACTCCCCTC<br>CCTCCTGGGAAAGCTGAGTCCCTCACGCACCCAAGGTTCTCA<br>CTCAGCAGGACCGGCAGCCGGCCAGACGTCCCTCCCCTCCTG<br>CCTGCAGACACTGGCAAAGATACCCGCTGTCCTGTGGCTGCT<br>GGAGTTGGGGTAGCTGGGCAGACCCAGGGCCTGGGGGCCCAG
MSTRG.33957.1	0.01	0.13	0.01954	3.075	up	CAGATATCTGATGGTTCATCTCTTTAAATAATGGGTAAATTT<br>GTTTTCTGTTGTCCTGCAGGCAGAGTTATTAGAAGGTAGAGG<br>GAAGCAAATTTCCATTTAAGATGGAAAAGAACTTGCCTATTT<br>AGTTGACTATTGAAACTGCTGCCTTGCAAAGAAATGAACTTC<br>TGGTCCCCGGAATTGTTTTAACCGGTGTTTTTAAGATGTAAG<br>TTTTGTCTTTATTCAGGTATGGTGAGCAGGGGACTACTGCCA<br>GTGAAAAAAAGATAGTTTGTTACTGTTCCGAAGAGGAGAGGG<br>CACGCCATGCCACACAGGGACCCACAGGACAGTTTTGCTGGA<br>TATGGCTTCCCCAGTAGCTCAGATGGTGAAGAATCTGCCTGC<br>GATGCAAAAGACCCATGTTTGATCCCTGGGTCGGGAAGATCC<br>TCTGGAGAATGAAATGAAAACCCACTCTAGTATTCTTGCCTG<br>GAGAATTCCATGGACAGAGGAGCTTGGCGGGCTACAGGCCAT<br>GGGGATCCAAAGAGTCAGACATGATTGATAGACTAACACTAC<br>AACTACTACTAATCCAATATGACTGGTGTTTTTAAAAAAGAG<br>GAAATTGGGGGACAGACATGCATACTGAATGAAGACAGTCAT<br>CTACAAGCCAAAGAGAAAGGCCTGGAAAAGATCCCTCCCTCA<br>GGGCCCTCTCAGGAAGAACCAACTCCATCTTATTTCAGACTT<br>CTGTCCTCCAGAATTGTGAGATGATACATTTCTGTTGTTTAA<br>ACCACCCAGTCTGGGGTTTTTTGTTATAGGAGTCTTAACAAA<br>TGGCCTCCCTGGTGGCTCAGTGGTAAAGAATCCACCTGCCAA<br>TGCAGGAGACACGTTCGATCCCTGGGAAGATCCCCTGGAGGA<br>GGAAATGGCAACCCACTCCAGTATTCTTGTTTGGGGAATGCC<br>ATGGACAGAGAAGCCTGGTAGGCTACTGTCCATGGTTTCATA<br>AACAGTTGGACATGACTGAGTGACTCAACAACAACAACAGAT<br>TAACACAAATCTCAACTTCGGCATGTTTCAACTGTGCAAATA

MSTRG.121502.1	0.03	0.22	0.02414	3.053	up	CAGGCATACTCAGACTTGTTCAAACACAGGCTGGTGGTGTGGCAAGGATAAAAGCAGAAATGATAAGGATTGGACTCCTGGACCTCCAGGAGGAGGAGACCTAACTCCTACAGTTCATCTGCTCCACAAGCCTGGGTCTCCCGGAACCCCCCTGTGTCAGATTGCTGGGCCTCCCACCTACCTGGTGAAAACCTGGGAGAAGCAACAGTTTGTGTGATGGTGAGAAACCTCATGTGAAGATGGCATTAAAGAAATTCTCATCACTCTGTGATCTGGGACAGGCAGAGTCTAGATGGGGAGCAATCACGAAGGGAGATGATGAGAGCTGAAGTCAGAATATGCAAAATATAAGGACTTCCCTGGTCCAGTGGTCAAGAATCTGCCTTCCAATGCAGGGGATGTGGGCTCAGTCCCTGGTTGGGGAAGCAAGATCCCACATGCCTCAGAGCAACTAAGCCTATATGCTGCAACTACTGAGCCCGCCAAATAAATAAATATAAAAAT
MSTRG.43630.13	0.01	0.15	0.0061	3.043	up	AACCAGCCCATGATCAGTGAGCTCCGGGCAGTTACTTTGGTCCCAGGTTTGGAGCCCAATGCCCGGAACGCGGGAGAGAAGAGGCTGGCTGCCGGCGGGTGCTTGCAAGCCTGTACTGGGAAGGACCGAGTGAGTTCCACACCGGCGGAGCTCCAGCTCTGCGCCCAGGCGCGATAGCACAGAGCCCCGGCCGAGAGGCTGCTCTGTGCCGGGCGAGCCTCCCCAAGCCCTGCCCAGCTTCTGGCGTTCGCGCCCGGGAAGGAGCAGGCTGCGGGAGTGTGGGAGCCGTGAGTGGTGCCTGACTGCTGCCTCAGCTCAGCTCTCGCCTTCCCTTCGGACCTAGGAGCCAGGCGTCCTTGCAGAGGGCTTAGGGGCTGGGGGTGTGCACACTGCCAAAGCCTCCCGCGGGGAGAGCTCTTTCCGAGGGAAAGGAAGCGCCCCAGGCTGCATTCCCAAGCTGGTGCTTCCCCGCCCAGACTAAGCGCCGCACGGAGGGAATCAGGGTCCCGGTGACCCTGGCTTGGGGAGGCAGCTCCACAGCCAGAGTGTTGCTCCGTTTGTGCCCAGAAAGCGCCTGCCAGACCGGGCCTTCCCTGGAGCTGTCCTCCCGCCTAGAGGCAGGTGAATGTGCTGTGGGGGATTCCCCTGGGCGTTTGGGCTCCCTAGCAGAGCCGCAGCGCAGGGGAACCAGCCCATGATCAGTGAGCTCCGGGCAGTTACTTTGGTCCCAGGTTGGGAGCCCAATGCCCGGAACGCGGGAGAGAAGAGGCTGGCTGCCGGCGGGTGCTTGCAAGCCTGTACTGGGAAGGACCAAGTGAGTTCCACACCGGCGGAGCTCCAGCTCTGCGCCCAGGCGCGATAGCACAGAGCCCCGGCCGAGAGGCTGCTCTGTGCCGGGCGAGCCTCCCCAAGCCCTGCCCAGCTTCTAGCGTTCGCGCCCGGGAAGGAGCAGGCTGCGGGAGTGTGGGAGCCGTGAGTGGTGCCTGACTGCTGCCTCAGCTCAGCTCTCGCCTTCCCTTCGGACCTAGGAGCCAGGCGTCCTTGCAGAGG

MSTRG.148893.4	0.08	0.73	0.01047	3.019	up	GTACAGGCTTGCAAGCACCCGCCGGCAGCCAGCCTCTTCTCTCCCGCGTTCCGGGCATTGGGCTCCAAACCTGGGACCAAAGTAACTGCCCGGAGCTCACTGATCATGGGCTGGTTCCCCTGCGCTGCGGCTGTGCTAGGGAGCCCGAACCCCCAGGGGAAGCCCGCACAGCACATCCACCTGCCTCTAGGCGGGAGGACAGCTCCAGGGAAGGCCCGGTCTGGCAGGCGCTTTCTGGGCACAAACGGAGCAACACTCTGGCTGTGGAGCTGCCTCCCCAAGCCAGGGGCACCGGGACCCTGATTCCCTCCGTGCGGCGCTTAGTCTGGGCGGGGAAGCACCAGCTTGGGAATGCAGCCTGGGGCGCTTCCTTTCCCTCAGAAAGAGCTCTCCCCGCGGGAGGCTTTGGCAGTGTGCACACCCCCAGCCCCTAAGCCCTCGGCAAGGACGCCTGGCTCCTAGGTCCGAAGGGAAGGCGAGAGCTGAGCTGAGGCACCAGTCAGGCACCACTCACGGCTCCCACACTCCCGCAGCCTGCTCCTTCCCGGGCGCGAACGCTAGAAGCTGGGCAGGCCTTGGGGAGGCTCGCCCGGCACAGAGCAGCCTCTCGGCCGGGGCTCTGTGCTATCGCGCCTGGGCGCAGAGCTGGAGCTCCGCCGGTGTGGAAGCTCACTCGGTCCTTCCCAGTACAGCCTTGCAAGCACCCGCCGGCAGCCAGCCTCTTCTCTCCCGCGTTCCGGGCATTGGGCTCCAAACCTGGGACCAAAG
MSTRG.90496.2	0.02	0.13	0.01158	2.971	up	GGCGAACCTGCGCGCGATCTCCGCGGGTCCCCGGCGCCGAGGCCAGGACGGCCGGCGCTTTGGCCTTGGATGGAAATGGGGACTGGTCCTCCGGGCCCTCTACCGTGGATTACTTGGTGCATCTCTGCCGGGCTTGATGAACCACGACCTCGTCTCCATCCGTGGATTGGTGCGTGCTACCCAATAGTCGGGTCGTTTCCCTGGAGCCTAAGGATCTCCAGTGATTTCTTTGAGCCTAATGATGGGGAAGCTGAGAATGGAGCTATTATTTTTAAACACCTTTTGAAATACTACTCTTCCTGGTTTATGAAGTGAAATCAGAGGGCTGTGGCTGACCTGAATAGATGCATAGATGCTTTGAACCAAGAAAGCAGGACAAGTTGAAAGGCGAGCTGCACCCCGAAATCAAGGCCTGGGAGGCAGGACAGGTGGAGAACTGTTCCTCTCGCCCACAGTCCCCACCAGGAACCTCAGTGTAACCAGGAAGACCCCAAGGAGCTAAGATGGGACCTTCTCTTAAGAGGCAAGTGCTCTGGATTGGAATGAGAAGTCTTATTTTGGAGAATGGACTTCCCTGGTGGCTCAGTTGGTAAAGAATCTGCCTGCAACATAGGAGACCCAGGTTCGATCCCTGGGTCAGGAAGATCCCGTGGAGAAGGAAATGGCTACCCACTCCAGAATTCTTGTCCCATGGACAGAGGAGCTCGGCAGGTTACAGGTCCATGGGGTTGCAAAGAGTTGGACGCAACTGAGTGACTAACACACACACGCTTTCTGGAGGCTAAAGGAGCTTCTGGATTTAAGAAGATGTTTGAGGAGAAAGCAAAGAACCTCGGCCTGAGGAATCCTGTGTGTGTTGACCTGTGTGAACAGGAGGCCACCTCCAGCTGAAGGGGAAGCCATGACCGTGGACCTTATAACTTCTTCTGCTGGTGCCTGGTCTTCAGACCGTCCCCCACCAGTCCTCGGAAACCAGAGGGTTTTCTCCTGCATTCTTTCATTCTCGCCACCTCCCACGTCTGCTGAGCCTTCGGACTCAGCAGTTCTCTT

MSTRG.22144.1	0.02	0.13	0.03511	2.945	up	CTTTAGGATGCCCTTGGAATTAAAAAAAAAAAAAATCAAGAG<br>GTGACCATGCAGGGGGATTGTCTGGCTTTTGGAGGCTCTCAC<br>CAGTTGGCAATAGTTAGAGAATGACTCAGCTGTCTGGCTCTA<br>CCTTTCAGACAGATTTAGGTGACCCAGAGACTCATCAACGGC<br>AGTAAGGCTTATTCACACATAGGCATGCCTGGATTAGCCGAG<br>TCCCATGGGTGCACAGGAAGTTTGTCTTTCTTTCACTGCTTC<br>AGGTACCTTTGATATAGCTCCAGTGTCTGAAATTGAGAACAA<br>CACAAGCTGCTAATAACAAGGAAGAGGAATGGAAGCTGACTC<br>GGTTATTATGACAGTACTGTGCTTTGAGCCAATCCTGGGGAC<br>ACTGACCTCTAGCTTCATGCCCTGCAAGACAGTTACATGTCT<br>TTATTGTTTATTTAACTCAATAGCTTGGCATTCCCTTATTTG<br>TAACCCAAAGTTCCTACCTTCTAGATTAAACTAATAAAGATG<br>GCACGATGACAACTGAAAGTGAAATGGAAGTTTTAAAGAAGA<br>CTTCCAAAAATAAACCCAAGCGGCACATATTCCAGTCAGCTA<br>AGACCCAGGTGTGAGGGAGTTCTGATTTGGATTGATGGAGAA<br>GGAAATGGCAACTCACTCCAGTGTTCTTGCCTAGAGAATCCC<br>AGGGACGGGGGAGCCTGGTGGGCTGCCATCTATGGGGTGGCA<br>CAGAGTGGGACATGACTGAAGTGACTTAGCAGCAGCAGCAGC<br>AGCAGTGTGGTTTACTACCAAAAACACCCAGAAACTCATTAG<br>AGGATTGCTCAGTTAGTAACACGATGTCAAGGATAGGCACTT<br>AAAACAGTATTGTAGGGCGTCCACAGTAGAGGTCGTACAGAT<br>CGCAGATTCTGTCTATGAGGATAAATAACTCGGGCTGTGCTC<br>AGAGGGCATCACATGATGTGGCTGACTTGCATCTCAAGTTTC<br>CCAAGAAGAAAGCAGTGGTGCAAGAGCAAATTCCATTGCATT<br>TTCCCTATCAGGAACAGTTTGTAGACATTTGTTTCCAAGGAC
MSTRG.110124.7	0.02	0.19	0.03659	2.928	up	AGCAGTTGCGAAGTGAGGTTGGCCTGCGCGGGTGGAAGCAAA<br>CGTGACGTCAGCTTGTGGCCTTTGCTGCTAAGTCACTTCAGT<br>CATGTCCGACTCTGTGTGACCCCATAGACGGCAGCCCACCAG<br>GCTCCCCCGTCCCTGGGATTCTCCAGGCAAGAATACTGGAGT<br>GGGTTGCCATTTCCTTCTCCAGCGCATGAAAGTGGAAAGTGA<br>AAGTGAAGTCGCTCAGTCGTGTCCGACCCTTAGCGACCCCAT<br>GGACTGCAGCCTACCAGGCTCCTCCGTTCATGGGATTTTCCA<br>GGCAAGAGTACTGGAGTAGGGTGCCATTGCCTTCTCCTTGTG<br>GCGTTTGGTAAAGGCCAAACTTTACCAAAAGTTTGTCACTTC<br>TGTAAAGGCAGAGCCACCTGTGTGAGGAGGACATCAGCAAGC<br>AGAGCTGAGAGTTCAGGAGAGATGGTTCCGGCTGACGTCCTA<br>AAACTCCCGAGTGCAGCCCTGGCGGACGCTTGTGGACTTAGG<br>GACCTGTCCACCTGTGGATGGCTACAGAAAGGAAGAAATTAA<br>CACTTCTTGGTCTGCCAGCTCTCCAAGTAAAGTCATATTCTT<br>TGCCTCAAGACCTTGCCTTTGATTTATTGGCCTGTCGTGGGG<br>TAAGCAGAGTGAGCTTGGACTCCATGATAATCCCACATGCCT<br>TGCAGTCAAAAAACCAAAACATAAACAGAAGCAATATTGTAA<br>CAAATTCAATAAAGACTTTGAAAATGGATCATATCAAAAAGA<br>TCTGAAACCAAAAACTGAAAAGAACTCGGTGGTCGTCTGAAA<br>TATTTAGAAAAGAAAATGGTGCCCCTCATGCCAGTACCCCCT<br>GAAGTCACACAGGGGACACAACCTAGGGGAGGTCCCTTATCT<br>CGTCCAGGGGACTGTCATACCAGCCACCATTTCTCCCTGCCC<br>ATGATAAACACACTGTAACGAGGGTCATTGGCACCTGGGCCA<br>GTAATGCTGTGAACACCTGGATTCCTTGTCCTTGTGAGATTT<br>GATGTGAAGATAACACAGAAAGAGAACCAGCCAAGAAGGATC

MSTRG.224419.1	0.12	0.99	0.01518	2.903	up	TTCGCGAAACTTCTTGGGGGGTGGCGCCGCCGCCCTCTCCTC<br>TTTTCCCTCCCCTGGGTCCTTTACACCTCGGTGGCTCCCCCG<br>GGCTACTTGCGGGCTCTCCGGGCGAGAACAGGGGTGCCTTGG<br>TGAAGCAGTAATGCGGGGACTTTGGGAGAAAACGGGGCCAGA<br>ACGTACAGGAAATGCTGTTGAGGCCCGCCAGGGCTCAGCCCG<br>GCCCTGGGAAGTGGGAGTCCTGGAGCCCGGGGAAGGGACCCA<br>GAGCCGCCCTGAAAACCACAAGCCCATGATCTGCCCTTCTGC<br>GCCGTCGCAGCCGCCGCGCAGCCGCCGCTGTCCTGCGCCAGG<br>CTCCAGCAATACTGAACCTTGAATTTCCACATGTTCAAGCTG
MSTRG.221949.7	0.04	0.3	0.00869	2.879	up	CGGAACTTGGGAGCAGATCATAGCTGCTAGGTAAGTACGCGT<br>TGTTGGCGGTCGTGCGCGCGAACCTGAGTGGCGCAGCTTGGG<br>TTGGGGAATGCGATACGGTAAAAAGAGTTCAGAAGTTGAGGG<br>TTAGAGTGCGGCGTTGAGTATTTACACATGGAAAACGCGCCA<br>CCGGACCGCCACGTGTCCCCTGTCGCGGTGGCGACCGGCTTC<br>GAGTTCAGGCGTGCGTAGGCTGCTGCTGGTCATGGCGCTGCG<br>CGCGGCCGTATTGCTAAGCCGGCTGGATAATCCTGAACGGCG<br>CACGGGAAACGACCACATTCCTGAGATTTGGGGCGGGAGTTA<br>CGCTTAACGTTGACTTACAGGCCTTAGATACGTAGTCATATC<br>AACAGTTAATTTAAAAATCGGCAGCTCCTGACTGCGTGAAAC<br>TGAGTTGTATTGTGCCCAGGTGGCGCTGTGTGCAAATCGAAT<br>TGTTGGAGGGGAAGGTTTTAGTGCTGAAAACCTTAAGCTATG<br>ACCTTCTATGACAATACTTAATTTTTAAATATATTTATTCTT<br>CCGCAGGTTAAGAAATTGATTCTCCCGCAGAAACAATGGATT<br>TCGGTGTAGCGGAACTGTTACGTGGAGATGCTAAGATGCAAA<br>GGTACATTTCAAAACACGCCGGACAATATTGGCTGGAAAATG<br>ACTTGGTTAAAACCTGATGATCTTACCAGGTATGTGTTCGAC<br>TAGTACTTCGAATGTCTATATGTTTAGTCCACGTGTAGATTA<br>AATGAGAAAAATATTTTTGTTTTTTAACAGCATTCTGCAGGT<br>TGATGAATACTAATGAAGCTGTGGATGTCACTGAGCAGCTTC<br>ATTTTAAAGTAAGTTACCCTGACTTACATCTGCTGCAGTAAT<br>TCTGTCTGGTTTGTGGTAAATGTCCGTGTAATGTAGCATAAT<br>GGATTTTGGTCTGATCAATGATGAACCCTATCCCGAAGCTGA<br>TAACCTGAAGAAAAGCATGTACGGATTCGGCTTCTGAGACAA<br>GACTAATGTTCAGATATGAGAACAAGTTTTATAACAGATTTC
MSTRG.81913.2	0.04	0.29	0.04159	2.834	up	TAACGATGTGACGTCATGAGGCTTTCGGCCTGCCTCCACCAA<br>GCGGCTGGCCGCAAGGAGAACGTGACGATTGGCCCACGAGAA<br>AGGAAAGCAGGCTTTAGGCTTCAGAGATTGGCTCAAACAAAC<br>TGTCACTCATCGTCCACTCTGTGGCTCTCCCAGTTTGTAGGG<br>AATCCAAGGGGGAATAAAGGAGCTTGTGCCCATTTTCCTGCG<br>GGAAAACTGAAGCAGTGAGAGGCTCAAGGAGAGGGAGGTCTT<br>GGCCAGGATCCCGAATCAAGTCAGAAATGGGACTCAGGCATC<br>GAGGTATTTAATCTCTAGCCCTCTACTTAATTGTTACAAGTT<br>TTTTAAACCCAGTGACCCCACTCAAGCACTTGGCCTTTGCCG<br>AGTCCTACCACGCTGGGAACACCCACCCGCTTCGTCTGGCCG<br>TCATGAGTCAGTCCCGACACAACTCAGTCGCCACATCCTCCA<br>GATGGCAGCCACATGGGACAACTGAGTAATGGGCCATGACTA<br>GTCTTAGCTGAGATAAGCTATGGCTGTAAAACACCAGATTCT<br>GAAGACTTCAGTGCTGCATTAGGTCATAGACCACGAAATGGA<br>CAAGATCACTGGAGAGTTCGTGTGATTAGAAAGGTGGTCAGA<br>AGACCTGAGTCAAATTGAAGAGTTGGGTGTCTCTAATGAGGC<br>CGCAATGATTCTGCTAAAACCAGCACTGGCAAGAGGCCCCCA<br>GTCTCAGGTGAGACTGAAGCTGAGACCTTGATAGCAGCATAG<br>TCATACCCTGAGCCAAGCACCCTGCTAAGCTGCACCCGTGCC<br>CCTGACACACAGAGACTGAGATTAATAAATGCATTGTTGTAA

MSTRG.215663.1	0.03	0.21	0.00597	2.821	up	ACTTCCAGAACACAAGAAGTTTACACTGAATGGCAATTTTTA<br>GATCTTGGCATAAAGTTGTACTGATTTCAACACAGTGGCTTT<br>TTGCAGTCACAAATACAGAGGATGACATGAGAATTTAACACG<br>TGAAGTTCGAGTCGTCCAAGAGTTTACAGAAGAGATTCAGAG<br>TTTTTATTTTATTTCTCCTCTGAAAGGCTGTTATTTGGAGGT<br>TGGGTGCAGACTTCCGATCCTACAAGTTCAAGGCTTCTAAAA<br>CAGGGAACTTGCTAATCTGGAGTTAAGTTTGCGCCTGGATTT<br>ATTGGAAGGGCTCTAATGCAACAACCTTCACCACCAAAAAGG<br>CCTCGAAGTGCAATCTACGTATGGGTGTGGGCTGGGTGGCGG<br>GAGAACTGCAGAGAAACGATTCAACATTTATGCAAATAAAAG<br>GGTCACCAATTCTGCGGCGAAGAGGGCAAAAAATGCATTTTC<br>ACCACCCCTCACACTCACTCTGCGGCTGACGGAACCTGATAC<br>CATTTTATTTAAGGCTCATTATCTCCAGTTACCACCTCCAAG<br>ACCTCAGCGCCTAACCAGGTCCACGCAGGATCTTGAACGGTA<br>CGCTAGCCATTCCTCGAGAGAAACCCAAGGCGGACACTCACA<br>AGACGGCCACAGTTTTCCTACTGCCTGCTGCCAAGTGTCCTG<br>AGGGCCCAGCTATTGGGATAAGATGTTGCAGAATTTTGATGT<br>ATTTATTTATTATTATTATTTAAAATTTATATATAATATACA<br>GCTCATACATATTGTATATAAATTCTAAAATTATATGTATGT<br>ATAAAAACCTCTGAGATTTCAGATTTTCTGTAGTGTCCGAGG<br>GCTAGAAAATGTGGCAGTGGGGGAAGGTAGTGGTGGAAGGAT<br>GGGTGAGAGAGAGAGGAGAGAGAGGAAAGGAGTGGAGAGAAG<br>CCCAGAGGGCTGGGGGTGGGAGGAAAGAGAAAGGAAAGGGCA<br>ATGAGGGAAAAGGTGCTGGAAAGAGCACAGGCTTTACAATCA<br>GACAACTAGCTTGTTACTCTGTGACCTTGGGAAAGTTACGTA
MSTRG.8488.1	0.04	0.29	0.00911	2.812	up	ACTTCCAGAACACAAGAAGTTTACACTGAATGGCAATTTTTA<br>GATCTTGGCATAAAGTTGTACTGATTTCAACACAGTGGCTTT<br>TTGCAGTCACAAATACAGAGGATGACATGAGAATTTAACACG<br>TGAAGTTCGAGTCGTCCAAGAGTTTACAGAAGAGATTCAGAG<br>TTTTTATTTTATTTCTCCTCTGAAAGGCTGTTATTTGGAGGT<br>TGGGTGCAGACTTCCGATCCTACAAGTTCAAGGCTTCTAAAA<br>CAGGGAACTTGCTAATCTGGAGTTAAGTTTGCGCCTGGATTT<br>ATTGGAAGGGCTCTAATGCAACAACCTTCACCACCAAAAAGG<br>CCTCGAAGTGCAATCTACGTATGGGTGTGGGCTGGGTGGCGG<br>GAGAACTGCAGAGAAACGATTCAACATTTATGCAAATAAAAG<br>GGTCACCAATTCTGCGGCGAAGAGGGCAAAAAATGCATTTTC<br>ACCACCCCTCACACTCACTCTGCGGCTGACGGAACCTGATAC<br>CATTTTATTTAAGGCTCATTATCTCCAGTTACCACCTCCAAG<br>ACCTCAGCGCCTAACCAGGTCCACGCAGGATCTTGAACGGTA<br>CGCTAGCCATTCCTCGAGAGAAACCCAAGGCGGACACTCACA<br>AGACGGCCACAGTTTTCCTACTGCCTGCTGCCAAGTGTCCTG<br>AGGGCCCAGCTATTGGGATAAGATGTTGCAGAATTTTGATGT<br>ATTTATTTATTATTATTATTTAAAATTTATATATAATATACA<br>GCTCATACATATTGTATATAAATTCTAAAATTATATGTATGT<br>ATAAAAACCTCTGAGATTTCAGATTTTCTGTAGTGTCCGAGG<br>GCTAGAAAATGTGGCAGTGGGGGAAGGTAGTGGTGGAAGGAT<br>GGGTGAGAGAGAGAGGAGAGAGAGGAAAGGAGTGGAGAGAAG<br>CCCAGAGGGCTGGGGGTGGGAGGAAAGAGAAAGGAAAGGGCA<br>ATGAGGGAAAAGGTGCTGGAAAGAGCACAGGCTTTACAATCA<br>GACAACTAGCTTGTTACTCTGTGACCTTGGGAAAGTTACGTA

MSTRG.198921.1	0.02	0.16	0.03246	2.812	up	CCATCCCTTCATTCTTTCTGGAGTTATTTCTCCACTGATCTC<br>CAGTAGCATATTGGGCACCTACTGACCTGGGGGCATCAGAGG<br>GCAGACACACTGAAACCATAATCGCGGAAAACTAGTCAATCT<br>GATCACACTAGGACCACAGCCTTGTCTAACTCAATGAAACTA<br>AGCCATGCCCATGGGGCAACCCAAAATGGGCGGGTCATGGTG<br>GAGAGATCTGACAGAATGTGGTCCACTGGAGAAGGGAATGGC<br>AAACCACTTCAGTATTCTTGCCTTGAGAACCCATGAACAGTA<br>TGAATAGTGACTTTAGAGCCTGGTTATTCAGGTAGCAAAGGG<br>GCAAGCAACCTTAAAAGGCCAGGGAACGTGGGTATATGGTTA<br>AATATGCTTCAATATAGCATCAGCAAGGGCTTCCTGGATAGC<br>TTAGCTGGTAAAGAATCTGCCTGCAATGCAGGAGAAAATGAA<br>AAAGACTGAGTGACTAAGCACAGCGCATAGCATCAGCAAACA<br>ACGTGAAAGACTGCATGGTACGGTGACGGGAGGTACACAGGC<br>TCTGACCCCTGCAGCCAAAGCAGATGGGAGGACAGGCATCAT<br>TATGAAGACCTCACAGCAGAGAGAATTCAGAGCTGGAAGGAT<br>GAACAAGTGGAGGCAGCTGGGGGCACTAAAGGACGCCCCAGG<br>GAAGAAGCTCACAAACTGTGGTCTACTCCGGGAATGGTAGAG<br>TTGGATATGTTTGTTTGTTTTCTACCCTAATTAGTTTTCTTT<br>GTCACTGCCTGCGGTGTGTCCACAGGATTTTCCTGATAACTT<br>CATAAGGCATGTTACTTTGCCTTAATCTGTTGTATTTCCCAG<br>ACACTGGTATGCCCTCTGTTGATTCCTGTGGGTGAGGAGTCT<br>GGCAGGCTTTAATCCATGCGGTCACAAAGAGCTGGACACAAC<br>TGAGTAACTAAGCACAGCACAGACGCGCTAACGATTGCGTCC<br>CGTTGGCTGTAGCCCTTTGGCTGTCGGCCTGGGCCTGTGGTG<br>GTCACCTCCAGGTGTGAGTGGCCTCAGTGATACACACTCTGT
MSTRG.42208.1	0.02	0.18	0.02006	2.773	up	ATCTAGTCTTCTCTTGCTGCTGCTAAGTCACAGAGTCAGACA<br>CGACTGAAGCAACTTAGCAGCAGCAGCAGCTGGGTTAACGGC<br>TTCTGCCCAGCAGCCAATTCTTACATCACCCATCGTCACTTC<br>ATTGTTTCTTCTACTTCTGCCCTCAAAAATGTTGGGACACTC<br>AATTAAATCTTCAGTTCTTGAAACTGATTTATAACTATGTAT<br>GTGCACAAATAGGATTTGCAGAACTGAAATAAGGTACAGGAA<br>GTGAAATTGGCTTGTTTACTCTGATTGTTTTTGCCAGTTTCC<br>TATGGTGGAGGCTGGAGAACACATTGGACCACAGTATAGAAA<br>AGAAAAATATGGTGTAATATTGGACATTACTATGTCTTATAA<br>CTAAGTTTAAAAGTAGGTATTAACCATTTCAGACAATGCATG<br>AATAATAATTAAACATTAAAAAGTTTAGCATGTGGAATTGAT<br>GCTTTGTGTAATAATACTAAAAACATTTGGTAATCTTTTTTA

MSTRG.108770.3	0.03	0.23	0.02723	2.772	up	GTTACTTCAGTCCTGTCCAACTCTGTGCAACTGTATGGATTT<br>CAGCCTGGTGGGCTGCCGTCTATGGTGTCGCACAGAGTCGGA<br>CACGACTGAAATCACTTAGCAGCAGCAGCATACCATGTGAAT<br>TATGTCTCAATAAAAAAAAAATCAAAAAGAGAAAAATAGAAG<br>GAGAAGTTGTTGACACTCTGCTCCAGACCCTCCCACATCTCC<br>CTGCCATCCTCACCGGCCCCCTCCACGGGCCCTTGGCACTAC<br>CCTTGCCCCACAGCTGGTTGGTAGGTAGCAAGACAGCACACA<br>CACAGCCGTTGCTATGGTGACATGTGACCGGTCCAAGCCTTG<br>TTTCTGCTCCTCATCTCCAAAGCAGGTGGGCAGCTGTGCCCA<br>GGACAAGCACAGATGCATTGATACCACATGGTAACATTGATC<br>TTCATTTCTGCAGGGCATTCCCAGGGCACGGGGGTTTCCAAT<br>TGAACCATGGAGCTTCTGGAAACAGTAGCCTAAAAGGTTTCT<br>CTGCCATCTGGTCTTTTAATGACCCTTCAGCTCAAAACTCCA<br>AGGCTTCCAGAGAAGCAGTCTGGATTAAAATGCTACTTTTTC<br>TTGCTGGAGCAGCTGCAGAAAGTTCTGAAGACACAGGCAATC<br>TTTAGATTACTTCATTTCAGGAATAAGAATTCTTTGCCCCTG<br>AGTAAGAACAACTCTTTATTTTTGTATCTGTGGTTTGCAGCT<br>TGTTTAATTCTTTTTTTAGTTTTTTAAGGGAATTCCATATTG<br>TTCTGGGGCTTCCCAGGTGGCACAGCGGTAAAGAATCTGCCT<br>GCCAATGCAGGAGACACAGGAGATGCGAGTTCAATTCTTGGG<br>TTGAGAAGATCCCCTGGAGGAGGAAATGGCAACCCACACCAG<br>TATTCTTGCCTGGGAAATTCCACAGACAGAGGAGCCTGGGGA<br>GCTACAGTCCATGAGGTCACAGAATCCAAAATGACCGAGCAC<br>ACACACACACACACACACACACACACACACACACACACCCCA<br>CTACTCTCCCTAACAACACCTGCTTTTCAATTCACTACATGG
MSTRG.17023.4	0.02	0.16	0.03395	2.703	up	CTTCCTTTATGCTTCACAACAACATATGAATCTCTTGCACAT<br>ATAATCCCATCTTGGTATCTGCTTCTTGGAGGACCGTGGTCT<br>AACATCTCATCTGATGAAGGAAGGAAAGAAGAAGGTGTATCA<br>CATTGAATGATTTGTATATATTGAAGAATCTTTGCATCCCTG<br>GAGTAAACCCAACTTGATCATGGTATGTGAACTTTTTGATGT<br>GTTGCTGAATTCTGTTTGCTAAAATTTTTTGTTGAGGATTTT<br>TGCATCTATGTTCATCAGTGATATTGGCCTGTAGTTTTCTTT<br>TTTTGTGTTGTCTTTGTCTGGTTTTGGTATCAGGGTGATGGT<br>GGCCTTGTAGAATGAGTTTGGAAGTGTTCCTTCCTCTACAGT<br>TTTTTGTAGGAGTTTTAGAAGGACAGGCATGAGCTCTTCTCT<br>AAAAGTTTGATAGAATTCTCCTGTGAAGCCATGTTGTCCTGG<br>GCTTTTGTTTTTTGGGAGATTTTTGATCACAGCTTCAATTTC<br>AGTGCTTATAATTGGCTTGTTCATGATTTCTGTTTCTTCC
MSTRG.83189.1	0.02	0.17	0.04115	2.685	up	GCACCCGGGGAGACAGGCATTCATCTCGAGTGGAAGCAAAGA<br>ACCCCGCTCTGCTCTCGAGTCGCGACGGGTATCTCTTGGAGC<br>TCACTGGGTGGACTAAAGGGAGTCAAGCCTCCTGAGGCGTTT<br>GGAGAGAGGTCGCGAGATTGGCCTCTAGGCCATGAAGGAGAC<br>GAAGGTCCTCATCTCCGCGATGACGGGGGAGTCTCGTGGTGG<br>TTCTCGAGCGGTGGCCCCAGGGTGCGGTTTCTCACGAGGTAC<br>GACGGCGAGGTCAGTGAGCCTCTCGTGGGGCGCCAGGGAAGT<br>CGGGTCTCCATGCGAGTGGCGAGGGGGAGCACGTCATTGCTC<br>TCGAGTCATGGTAGGGGAATCTGGCCTCGAGACGTGTTGAAG<br>AAGGCCTCTCGAGGCCTTTCTCGGGTTGAGGCAGGAAACCCT<br>GGCTTCCCTCGACTTGTGCAGGTGACCTCAGGGGACTTCTCA<br>TGGTGGCTCTGAGAAGTCAGGGAAACTGGAGGTGGGAGGGGC<br>CTCTCGGGACTCCACGGGGTTTGGTGCATTGGAAGAGGGCCT<br>CATCTCCAGTTGAGGCAGGAACCGCAGGGTACCTCTGATTTC<br>AGACTCCGATCGCAGGGTCCCTGCAGACTGGGGACAGGAGAG<br>TCAGGCCTCGTCTTGGGTCGAGGCATGGAACTCCGC

MSTRG.227580.1	0.03	0.21	0.03223	2.657	up	TGTATATTATATTCTGGGAAGTAGAATCAAAGTCAGGAATTA<br>TCAGTTACATAGTCTGAATACAAATGTTAAAGTCTGAATTGA<br>TAGGAAGACTGTCCTCTTGGACCAGAAAAGCAGTCTCCTTAA<br>GAGAGAGATGGAGTGATAGCTACTTCCCCCATTTTCCTAACT<br>GTATCTTGACTTTTCTCTTGATTGAAGACCCCGAAGTGCAAG<br>CCTGAGTCTCATCTTGTGGCTTATCAGCATCATGGGCTGCAC<br>CTGTAGCTCCATTCGTATCCTTCCTTGCTGTGGACTGTTCAC<br>TGCAAGGTGTCTGGCTACTGCTCTCTCTCATTGAAATACGAA<br>AAACACCTAATGATGGTCTTTGCACAGTAGAGCTTGAAGTAT<br>AGTCTAAAGGAAATAGATGTAAAGTGTTGTTTTCGTCTCTCT<br>GTGTGAGAGGAGAAAAAATGCTTTTGCTGTAATCAATTATAC<br>TTTCCTCTGAAGTTTCAGGTAATATTTCAGTAGGTAGGTCTT<br>CTTCTGGAGAAATACTGGAAAGGCTCCAAGGGAAATCAATCT<br>CAGTTCCTCGCTTTGGTGTGGAGATCCTCTGTGGGGAGGTCC
MSTRG.8306.3	0.46	3.05	0.02466	2.635	up	TCTGATGCCAAGAAGCCAAGAGAAGAGAGGATTTCAAGGAGG<br>ATAAAGTAATTGGTGGTGTCAAGTGGTACCAAGAGGTCATCA<br>GGAGGTACTGGTTCATGGCCCAGAGGTTGGGGACCCCTGCAC<br>TTGAATTGACCCAAGTTCCTGCTTCTCCATCATCATAGTGCC<br>ACTTTCTGCTCTGAACTACCCTTCCCTTTTGGTAACATGAAG<br>AAGGGATCCCAGAGAAGGAGATCAGGAGATGGAGAACCATTG<br>GTGGATTTTGATGAGTGATAAGATGTGGTCGTTTGCAAGAAA<br>CATTACTCTTGGAACTCTGAAGGTGGATCAGATCAGGACTTG<br>TCTAATTGAAATGTCATCTTTCCTCTGTACACCCACTTCCCT<br>GAAGAGTTCCTTCATGTTGTCTGAGGCATTGAGTGAGTAAAA<br>AAATCTGCATATCTCTGAGTTAGAATTATTCAGCTTAACTTT<br>AGATCCTATAGTCCCTTGTTTCCTTCATAATAGGATAAAGAA<br>AAAGACAGAGGAGTTCTGCTTTTCAGTGCCTTACATCACCCA<br>GACAGTTGGCAGCCAGGAGTCAGGCAGCTGCCACACTCTTCA<br>CGTATCTATTTAAAGTTCAAAGAGACACTGGATAACAATAAC<br>AAAGAGGTAAGAGTGGCCCTGACCAGCCCACCCAACAACAGG<br>AAGAATGGCTGAAAAAGAGGCTACATTATCTCGCTATGTGGT<br>CCCTATTCTTCTCTTAATTGGCTGGATTGTGGGATGCATCGT<br>AATGGTTTATGTTGTCTTCTCTTAGAAAGGCCAGAAGACTTC<br>AGACTGACATCATTTAGAAGAATTAAGAAAAGCATGAAATGA<br>CAGATTATTAAATGTTTCTCATGTAAATGACTGCAATGGTTG<br>ACATCAGTTTATAAACTTGCATTTGTAAACTGTTGCTTTGAA<br>CCTAAGAATCTCATCAGAAACATATTTTAAAATTCTGGGTAC
MSTRG.140824.1	0.06	0.42	0.03619	2.615	up	TGGGAATCTCATTTCCAAATATGAAAACAGAAGAACAGCACC<br>ATTCTGAGGAGGTCCTTGGCAAAGAAAAAAAAAAATCCAGGA<br>AAAGTTGTGAAAATGTCAATAATCCTAATAGGGAATAAAACG<br>CAGCTTCAGCCGATTCAGTGCAGCTGTCAGGACATCGTGAAA<br>GTGTTGACGCTGAAAGTGTTTGACTGAAGCCGTTCCTACAAA<br>CAAGGCTGTACGTTTCTCGCTGGCAATGAAATGGTGAAACGA<br>GTTCAAGGACAAGGAGCCGAGCCAAGAAAAGAGTCTCCAAAG<br>AAGGCTTCCCAGGTGGCTGGGTGATAAAGAATCTGCTTAACA<br>ATTCAGGAGCCACAGGAGATACAGCTTCCTCCCAAAAGTATC<br>TTTCAAGACAGCTCTGTTGTATAGAATGAAGTCACCGCAGGT<br>ATGGCAATGTTTTTCAAACACATGGAGGATAACTAAAAAACA<br>GCAGGTATGCCAATCCTAATCTCCCCAATCATTGGTCTCTCC<br>TTCTCCACGCCTCCCATGTATGTGTTCTCTACTCTGCTTCTC<br>TATGCCTGCCCTGGAAATGGGTTTATCTGTACCATTTTTCTA<br>AACTCCACATATACTGGTTAATATACTCTATTTGTTTTTCTC<br>TTTCTGACTTAACTCTGAGTGACAAGACTCTAAGACCATCCA<br>CATAGTTAGTGGGAAGCTGCTGTACAGCACAGGAAGTTCAGC

MSTRG.43630.19	0.04	0.23	0.01453	2.564	up	GGGAATCAGGGTCCCGGTGCCCCTGGCTTGGGGAGGCAGCTC<br>CACAGCCAGAGTGTTGCTCCGTTTGTGCCCAGAAAGCGCCTG<br>CCAGACCGGGCCTTCCCTGGAGCTGTCCTCCCGCCTAGAGGC<br>AGGTGGATGTGCTGTGCGGGCTTCCCCTGGGCGTTTGGGCTC<br>CCTAGCACAGCCGCAGCGCAGGGGAACCAGCCCATGATCAGT<br>GAGCTCCGGGCAGTTACTTTGGTCCCAGGTTTGGAGCCCAAT<br>GCCCGGAACGCGGGAGAGAAGAGGCTGGCTGCCGGCGGGTGC<br>TTGCAAGCCTGTACTGGGAAGGACCGAGTGAGCTTCCACACC<br>GGCGGAGCTCCAGCTCTGCGCCCAGGCGCGATAGCACAGAGC<br>CCCGGCCGAGAGGCTGCTCTGTGCTGGGCGAGCCTCCCCAAG<br>CCCTGCCCAGCTTCTAGCGTTCGCGCCCAGGAAGGAGCAGGC<br>TGCGGGAGTGTGGGAGCCGTGAGTGGTGCCTGACTGCTGCCT<br>CAGCTCAGCTCTCGCCTTCCCTTCGGACCTAGGAGCCAGGCG<br>TCCTTGCAGAGGGCTTAGGGGCTGGGGGTGTGCACACTGCCA<br>AAGCCTCCCGCGGGGAGAGCTCTTTCCGAGGGAAAGGAAGCG<br>CCCCAGGCTGCATTCCCAAGCTGGTGCTTCCCCGCCCAGACT<br>AAGCGCCGCACGGAGGGAATCAGGGTCCCGGTGCCCCTGGCT<br>TGGGGAGGCAGCTCCACAGCCAGAGTGTTGCTCCGTTTGTGC<br>CCAGAAAGCGCCTGCCAGACCGGGCCTTCCCTGGAGCTGTCC<br>TCCCGCCTAGAGGCAGGTGGATGTGCTGTGCGGGCTTCCCCT<br>GGGCGTTTGGGCTCCCTAGCACAGCCGCAGCGCAGGGAAACC<br>AGCCCATGATCAGTGAGCTCCGGGCAGTTACTTTGGTCCCAG<br>GTTTGGAGCCCAATGCCCGGAACGCGGGAGAAAAGAGGCTGG<br>CTGCCGGCGGGTGCTTGCAAGCCTGTACTGGGAAGGACCGAG<br>TGAGTTCCACACCGGCGGAGCTCCAGCTCTGCGCCCAGGCGC
MSTRG.122434.4	0.03	0.19	0.02685	2.56	up	ATTTTGGAAGGACACAGTTCTGCCCACAGCCAGCTCCCAAAG<br>GATACATTTCAGAAAGAAAGAAATCGATTATGAAAGTAAGGA<br>ATGGAGTGCAAAAACATTGAAATGATATGTTGTTGTCATAAG<br>TAAACTTTACTAAAGATGTCGGAGCAGATACACGCTGAGTGA<br>TGGATCATGGTGACCCCTTAAGAATGAACAAAGGAATGTTAT<br>CTTCTGAGGAATTATGTGGGCTGGTGTTCAAAGAAGAGCAAC<br>TGATGTTTATGTCTGAAGAGGTATTTCTGTCATTGGGAACTC<br>TGATTAATGCAAAATGTAGATGCATGATGTATAGGAGAGGGG<br>AGGGAAGAGACTAAAGAACAGAGAAAATTTTATGTTTAAAGT<br>TTTTCTTGTCTTGCCTGAAAATGTGAATTATTGCCTCATGTA<br>TATACATACAAACACCGTTTTTCACATTTCGGTGAGTTCAGG<br>ACTGCCGAATAAACCCAGTTAATAACCTCTTAATTACTTCTT<br>AGCCGAACTCTCCGTTCCTAAAATTCTGAAGTCCGCAAATTA<br>TAACTAAACTAAAATACCTTAGATGCCTACCACAAGATACAA<br>GATCTGTTGTTTTTGGATATTTCAAATGAAGTCCTTACAAGT<br>TACCTAAAGCATATATGAACTGTGCACCAGAGAAATCAACTT<br>TTTTATTCCTTTCTTCCTTCCTCTGTTAGTCACTCATTCGTT<br>TAACAAACATTTGTTTGCTAAGCACTTTCTATTTTGGAATAA<br>TACCTTTACGTAAAATCAACATCCAGTTTCAAGAATCAAAGC<br>AGTTAATGGATAGAATCAATATTTAAAAGAGACAATTACAAC<br>TGGGTGCTGTGACAAGGATCATGGAGGAGCAGGAGAGGAAGG<br>GGTGTGGGGAGGGGTCAGTGGGGAGGGCACCAAATACAGTCT<br>GGGAATTGGGTACGGCTGAGAGTGAAGAAGGCTGAGCGCCAA<br>AGAATTGATGCTTTTGAACTGTGGTGTTGGAGAAGACTCTTG<br>AGAGTCCCTTGGACTGCAAGGAGATCCAACAAGTCCATTCTG

MSTRG.5694.1	0.02	0.14	0.03815	2.551	up	ATCATTTGTCAGTCATTTGACAGATAAATTAAAAAGATTGCA<br>CCTTTGTACAGTTTGGAGTGAAAAAAAATGTGATCTGGCTGC<br>TGATGACTACAGAGACATAACTCTGGTTGTAAGCAATTAGGC<br>TGTTGGCTCGAGCAAAGTGATGGCTAGCTAGAACTTTCTTAA<br>TGGGTCACTAGGGATAGGCAGCATTCATCTCCATTTATCATA<br>ATGAAAACCAGGCAGAAGTTAAGCATGCGTGAACAGTTAGAA<br>CAACAAACTTTGTTTTTATACCATCGAGTTCATTAAGGGCCA<br>CTCAGTCTGTGCCAGCACTATTTCCCCGTGAAGCTATAGCTC<br>ATTGTTTTATTATAATGTGTTCTTGTAAATAATAGTCAACCA<br>CTTTTGTACTGGACCTAAGGTGAGGTAGGGGTGCATCTTCCA<br>CATTTTTCAAGAATTTCACAAGAGGAATTGATTATACACTTG<br>ATAAATGATCATTTTTATTATTTTAATTTTTATCAGTAGTAT<br>TGTAAATTGTGTTGCAAACTATACAATAGTATACAAGGTTAA<br>ATTCTTATTAACAATCTCACAAACAAAATGGTGCTCACATTA<br>TATGGGAGTCACAAAGGAGAGTACAGTTCTGAGCTCACAAAG<br>AGAAATATTAGATTTTTATTTATTTATTTATTTTTCAAAATC<br>AGACATGAGTAAGATTCTTTACATGGCAGTAATGACTACTAC<br>AATAGACATGTGACATTGGCAAAACTGGAGTTGAACATGAGT<br>GACAGCCACCTTCCAAAGACATTTTCAAAGAGGATTAAAGCT<br>GTGTCTCCAAAAACAGTAGCAGGAAGCAAATGTCAGAGAGGT<br>CTTGGCCCAAACTTACTTTTCGGTAACCATTAGTCATTCTCT<br>GACACCTCAAGGGAGACTTCTGGACAATACCACAGAGGTTGA<br>GACGCTTTCTCAGTAGTGCAGTCTTACCCTGTCCTAATGTTT<br>GAAACCAAAAATAACAGTGGCATACCAGTAAACCACACATCA<br>ATTTCATCTGATACGTGGTAAGTAGGGCTTGTCCACATTGCA
MSTRG.20952.14	0.13	0.73	0.02468	2.55	up	GTTGGTTCAAGGCCTTGTACTGATAGGAAAAGGAAAGCGCTG<br>CCTGTTGCTGAATTCAGGACGATCAGTCTCTCCTAACGATCT<br>AGGTTTTTTAAGCTATATTTTAGCCGAAAGTTTTCTTAAAGA<br>AAAAAAAACAAAAAACAGTAGGTTAGGGTGGGGCATTAGGGA<br>GACTAAAGGGGAAGAGAGGTGATACTTGAGCTTCAGTTAAAA<br>TCTTAAAGTAACCAGAAGGGAGGAAAGGCTGGTAACTTTTTA<br>TTTTAGGAGAATGTCTTTGAAAACATTTCTGCCAAGTTTGTG<br>GTTCTTCATGAAGCTGTAGTAAGGGCGCTGGATTAGAAAATC<br>TGGGCGTATGACTACACCAAGTGTTAAAGGCCCTTCCTTCTT<br>CCATGATTATAGTAGCAGTTTATTGTCCTGTGATAGCTGCTG<br>TTTTCATTGTTCTGAAGATGGTCAACTATCGACTCCACAGAG<br>CCCTGGATGCTGGAGAGATTGTAGATAGGAACGCAAATGAGT<br>TCACAGATCAGCGATCCAAAACTGAGCAAGGCAACTGTTCAA<br>CACGGAGAAAAGATAGCAATGGCCCGAG

MSTRG.87428.3	0.03	0.19	0.01164	2.534	up	AGGCTTTATAGGAGAGGACAGGGTCCCTGATCTCCTAGAGCTGGTGGAACCTGATGAAGCTGGAATGTGTGGGGGGCTGTGCTGTCAGAGAGAATCACAGGAGACTTCTGGGGAAGTGAAATACTGAGCTCAGCTGAAGGCTGAGCTAGTGGGAAATGATGAGGTAGTGGGAGGGTAGATGGGAGCATTCCAGGCACAGGGAACAGCGAGGCGAAGCCCTGCAGCAAGAGGGAACGTAGGTCCAAGGATGTGCAGGAAGGCCTGTGTAGCTGGGACACAGAGGGCAAGGGGTGTTTGCAGCCAGCAAAGTTGGAGGGGCAGTCAGGGGCTTGAAGCTCAGCACTGGGGTGGCAGTGAGATGATGGCACCTTTGGACGGAGGTGGGCTAGTCAGAAAAGGAGGAATAAGTTTGTGTAGGTGGGGCCAAGTTTGGGGCAGGGTGCCTCTGAGGAACCCGAAGTAAAGCTGGGCTGGAGATTTGGGGCTCAGTGCTCAGAGGTGGTGAAGAAAGTGAGTACAGAGGGAACAGAGGCCCTATGATTGGGCTGTGGAGCTAGGAGGTCTGCACTGGGGTGGCATGGGCACTAGGCGAGCAGCCAGGACTGTCTAGGAAGCCGCAGAAGCCAGGGCTACAGAGAGGGAACAGAGGGCAGTGGTGCCCTGTTCTCACCCATCTACCATCACACTGTGTAGCCTTTGTGAGCTTGGCAAGGCAGGAATCAGTCCCACTTTACAGATGGGAGGAATGCAGCTTAAGACAAAAGTGGCCTTACCCCAAGTCACGGAGATAGTCAGTTATGAACGCGGGCGGCCTGCCTTCTAGGAGTGCAGCACGTATCAGGGCTCCTGGCTGGGACAGATGGGGTCCTCCCTCTCCGGGTTTCAGTTCCCAGACTCAGCCAAGGAGAGTCAGTAGTTGGGGGGCAGTGGGCTCCACATCTGCAGGAATCATCCAGAGACTCTCGTCCCCACCAACCCAGGGCTGTTCCTTCCTCTCAGCTGCAGAGCCTGGAGCAGGAGAAGGGGCGGTGGCGGGTGGAGAAGGCCCAG
MSTRG.24164.1	0.02	0.12	0.01857	2.522	up	AGAAACCTACATATTGCATTTTTCTGAGTCTTGCAGTTTGTCCTTTTGCTAGTTGTCTATCTATACCTGTGTTGTCCAGTTTGGTAGCGACTAGCCACATATTTAACTTTTAAATATAAGTTAATTAAAATGAAATAAAATAAAAATTCTGTTTCAGTTGTACCAAATTTCAAGTGGTCAATAACCACACGTGGGTAGTGGCTACTATAATGGACAACAGATCTAGAGCATTTTTATCACTACAGAAATTTCTGTTAGACAGTGCTGATCAAAGCTGTTAACATTTAGAAGCTGTTCTTATTTCTTCCCCTTATGCTTTTCTACTCTCACGTTCTTTGCTTACATCATGATAGCTGAGCACATAAACATATATTATTGGTTCATAAAACATTTCATCATCAGATGATTATGGGACAGGCTTTCCTAGTGCACACACCCATGTGAAATTATGTAACTCACCACCTGGCTGGGACATGAACCTAGCGCAAAATCCATGGTCTTTTAACTGAGATCACATACCTGGTTTCAGGACTTAATGAGGCGCAGGTTCTTGATGTCTTATCACAGAAAGAATTCAGTGAGAGACAAAGTGATAGGTAAGAAATAGATTGATTTAGACAACACACTCCACAGAGTGTGGGTCATCTCAGAAGGCAAGAGGCCTCAGGATATGGGGTTGTTGGTTTTTATAGGGGTGGGTAATTTCATAGGCTGAGTGAGGTGTCTTCTAGGTATTTTGGGGAGGGGGTGGGGATTTCCAGGAATTTGGCCAGTGCTCACTTTTTGACTTTTGATGGCCAGCCTTGGTACTGTCATGGCACCTAAGGCTGTGTATGTGTCATTTATCTTGCTGATGTGTTACAATGAGCATATACTGAAGCTCAAGGTTTAGTGGGAGTTGACTTGTCCGCCATGTTGAACCTTTTTGGTTCTAAATGTCATGTCTGGGCTATGTCATTCTTTTAAATGTTGTGCCCTTACTCTTTCCCCTCTGTTACAGTTGTAACATCTTTTACTTTTTGTTTGTTTTTGGCTGTGCTGGGCCTTCAC

MSTRG.43629.9	0.03	0.2	0.03564	2.513	up	GCGAACGCTAGAAGCTGTGCAGGCCTTGGGGAGGCTCGCCCGGCACAGAGCAGCCTCTCGGCCGAGGCTCTGTGCTATCGCGCCTGGGCGCAGAGCTGGAGCTCCGCCGGTGTGGAACTCACTCGGTCCTTCCCAGTACAGGCTTGCAAGCACCCGCCGGCAGCCAGCCTCTTTTCTCCCGCGTTCCGGGCATTGGGCTCCAAACCTGGGACCAAAGTAACTGCCCGGAGCTCACTGATCATGGGCTGGTTTCCCTGCGCTGCGGCTGTGCTAGGGAGCCCAAACGCCCAGGGGAAGCCCGCACAGCACATCCACCTGCCTCTAGGCGGGAGGACAGCTCCAGGGAAGGCCCGGTCTGGCAGGCGCTTTCTGGGCACAAACGGAGCAACACTCTGGCTGTGGAGCTGCCTCCCCAAGCCAGGGGCACCGGGACCCTGATTCCCTCCGTGCGGCGCTTAGTCTGGGCGGGGAAGCACCAGCTTGGGAATGCAGCCTGGGGCGCTTCCTTTCCCTCGGAAAGAGCTCTCCCCGCGGGAGGCTTTGGCAGTGTGCACACCCCCAGCCCCTAAGCCCTCTGCAAGGACGCCTGGCTCCTAGGTCCGAAGGGAAGGCGAGAGCTGAGCTGAGGCAGCAGTCAGGCACCACTCACGGCTCCCACACTCCCGCAGCCTGCTCCTTCCTGGGCGCGAACGCTAGAAGCTGGGCAGGGCTTGGGGAGGCTCGCCCAGCACAGAGCAGCCTCTCGGCCGGGGCTCTGTGCTATCGCGCCTGGGCGCAGAGCTGGAGCTCCGCCGGTGTGGAAGCTCACTCGGTCCTTCCCAGTACAGGCTTGCAAGCACCCGCCGGCAGCCAGCCTCTTCTCTCCCGCGTTCCGGGCATTGGGCTCCAAACCTGGGACCAAAGTAACTGCCCGGAGCTCACTGATCATGGGCTGGTTCCCCTGCGCTGCGGCTCTGCTAGGGAGCCCAAACGCCCAGGGGAAGCCCGCACAGCACATCCACCTGCCTCTAGGCGGGAGGACAGCTCCAGGGAAGGCCCGGTCTGGCAGGC
MSTRG.174668.8	0.08	0.49	0.02622	2.495	up	CCGCCAGGCTCCTCTGTCCATGGGATTCTCCAGGCAAGAATACTGGAGTGGGTTTCCATTTCCTTCTCCAGGGGATCTTTCCCAACCCAAGAATTGAACCTGAGTTTTGTGCACTGCAGGCAGATTCTTTACTGTCTGAGCCACCAGGGAAGCCCAGTTAGTTTATGAATTGGGTATTATTCTACTTCTGGAAATGAGACTTGCAGAGGTTAATTTGTTAAGTGACTAATTCAAAGTCACAAGTTCCATATGCCAAAATGGCCTGAAACATGCTTGTCTGGCTGCAGAGTTTACGAGCTTCACTATCACATCACTACTGCCTGTGAACATCATAAAACTTCCCCCCGTTCCTTTTGTCACAAAAAAATATATAGTGACTCCTTTTTCTTCCCAAGTGAGAATACTGGAACTTTTCATCGTTAGTACAGTAATTAAATGAGGAATGTTATGACAAAAAACAAACAAACAAAAAAACCAATCACAGAGTGCCTTTGCCATGGAGTTTGTTTCCTCTTGTTCAGCTTTAATGAAGCCCTGTGAGGTACATAAAGGAGTGTCTGCTGTTCAAATCACAGGACTTCTCTATAGTGAAACCTTGTAATCAAAACATTGCCTGCAAGCAGTTTCCATAAACTTATATACCTAAGGAGGAGTTTTTACAAATGAATTTAAGAATTCTTGTGAATTTGGTTAATAAAAATTCTAAGGCTCAGTCCTGAATGCAAATAAAAGCAAGTTGTAAGTATATCTTTAGGATTAAAATGATGGCATGTGTCATTTTGCATTATTCCTACAGGTCATCAAGACTTCCCCAGGTGTGATTCCTCTTCCACCTTATTCGTCTTTATTTAGCAAAGCCCTGTCTGGCCCCTTCTCGCTTGTCCCGCTGCTAGCCTTTTCACTAATCTTAAGCCTTTTAGTTCATCAGTGATTCATGCCCCTAATTTAACATCCCAATAATAATATCTCTACAGTCGCAGCCACTATCAAAATGGCCAGTGTTATAAGTGCCTGTCCTATACTAACTTCAAAACACAGCCTTCAAAAGGT

MSTRG.90991.1	0.05	0.35	0.01945	2.493	up	GTCGTCCCGCTGCCTTCGCGGCGCTGAGGCTGCTCCCAGGCG<br>GCCACGGTTCCCGCTGACGTGCTCAGGACTCAGCAGACGGAT<br>TTCCTGCGGGTCCGGCTGCCTCTATTCGAGGGGCGCCAGGAG<br>TCCCTTCTGTTCTCCTCGGGCTCGGAAATAGAAAGGATGGTG<br>TCCGTGTGGAGGCGCTGTGTTTCAGGGGTGTGAGACACGGAT<br>ACATCTGTGTGAGGATCACAAAGCTGACAGATTGGTGCCCGC<br>TGTGGCGAGGTCGACCAGATGGCCCGACAGGTTGGGGATCTT<br>GGTTAACATTTATACTGTAATTTTATCCACAGAGTATCAAAT<br>TGAAGACGACCAGAGAAGGTGTTGAAATGAAGAACTTTCTTA<br>ACACACTGAGAAGTTGGGGAACCTCTCCTTCCAGCCACGAGG<br>CCCTGCCCTGCAGCAACCGACCTGGAAAACCAGACCCCAGCC<br>CCTGCAGCAATGGGCTCCAGGATTCAGTGCGTGGCCCATAAC<br>TGATGACTACACAACTCAGTTTAAACTCTGTGAAAGAAAGTA<br>AAGTTGCTCGGTCATGCCTGACTCTGCAACCCCATGGACTGT<br>AGCCTACCAGGCTCCTTTGTCCATGGGATTTTCCAGGCAAGA<br>GTACTGGAGTGGGTTGCTGTTTCCTTCTCCAGGGGATCTTCC<br>CAAGCCAGGGATCCAGCCCGGGTCTCCAACATTGTAGGCAGA<br>TGCTTTACCATCTGAGCCACTTAAACTCTGTACTAGGGATCA<br>TATAACAACAATGTATCCTGCTTGAGGACAGTTTCTCCTTCC<br>TGAAAACCTTCTGATTAATCCTGATATCTTAGAATGTATATT<br>ATGGGAATGGGTCTGGTAGGATCTTTCTGTTAAATTCTAATC<br>CTGTTATCCTAAAATGTTAATTGTGGGAGTGGGTCTGGTAAA<br>ACTTTCACAAACTTGAGACATTCTTTTGATTTAATGTAGTAA<br>CTAATTTAAAAAGTATATACATCCCTTGCTAACACCATCAAG<br>GGGGGGCACTCTCCACCCCCTTCTGCTGTCCATGACAGAAGC
MSTRG.30239.1	0.2	1.13	0.01448	2.471	up	CTTTTTAAGTAGCTGAACATCATGTCCAGTACTTTGCAAAAT<br>TCTTTCAGATCCTCTACTGACAGCTTCAGTAGAAACGTCACC<br>ACAAGCCTACTCGGTTGCAGTGAGACAGGTTCTCATTTTGTA<br>TTTCACCTCGGAAATATATCTACAATCCTGGCAAAGAATCAT<br>TCTTCTCCATCACACTGCAGGACTTAATTTGGTAAAAAAGGA<br>AAATTGAACTGCAGGACACTCGCTTTTGGAGCCGAGTCTGCA<br>CATAAGAAGTTTGCACGCTGTGAGGAAGCCCAAGTTACATGG<br>AAAGGCCACATGTGGATTCTCCAATCAACAGCCCCAGCTGAC<br>ATCCCACCCACCCAACAGCCAGATGGACCACCAGACATGTCA<br>GCATCCAGCCCACAGCTGCTGAGTCACTTTCCCAGGCGTCAT<br>GAGGCTCCAGAGCCAACCCACTGCCTTGTTGGAACGTCTGAC<br>CCCAGAATGCATAAGAGTAGTTATTTTAAATTTTTTTTTTGG<br>GGTGGGGGGATCATTTGTTACACAGCAGTAGTAACTGCAACA<br>AACTGTAAAGCTCTTCTGGATCCCTGCCAATCTTATCCTACA<br>ACTCACATGCACTGAAATGCATGAACTTATATATAAGTCTTC<br>CCTCTAATAATAATACTGTTTTAGTACTCTTGCCTGGAAAAT<br>CCCATGGACAGAGGAGCCTGGAAGGCTGCAGTCCACAGGGTC<br>CCTGAGGGCCGGATACGACTGAGTGACTTCACTTTCACTTTT<br>CACTTTCATGCATTGGAGAAGGAAATGGCAACGCACTCCAGT<br>GTTGTTGCCTGGAGAATCCCAGGGACGGGGGAGCCTGGTGGG<br>CTGCCGTCTATGGGGTCACACAGAGTCAGACACGACTGAAGT<br>GACTTAGCAGCAGCAGCAGTGATCTTCTTTCCCTTCTGCTGA<br>GTATCATTATGGAAAAGAAAGCCCAAAACGTCTAAACTTGCT<br>ATATTGACTAATGGTTTTATTTGAAGTGCTTAAAAAAAAAAA<br>AAAAACTATTTCAGAATTGTGTCACAAGTCTTGTTACTTTAT

MSTRG.25404.1	0.06	0.34	0.01259	2.464	up	GCTGGGTCTTTGCTGCTCGCTGGAAGTTGCTAGGACCTCTGGACTGCAGTCTCCCATCTCTTGCTGACCTCTTGTTGACCTCTGCAGAATCCTGGTTGCTGGGAGCTTGTTAGTTCCTTGTTCCTTACCCGGACCTCCTGTGGTAAATGTTTTCTCTCCTCTGTGGCCTGGTAAGGCTGGGTGGTCATTGGCATCCCAAAGGTGGCTAGAAGTCAATGATTTAGGCAGGCTTGTGAATAGATATGGGAGAAGGAAATGGCACCCCACTCCAGTACTCTTGCCTGGAAAATCTCATGGACGGAAGAGCCTGGTAGGCTGCAGTCCATGGGGTCGCTCAGAGTCGGGCACGACTGAGCGACTTCACTTTCACATTTCACTTTCATGCATTGGAGGAGGACATGGCAACCCACTCCAGTGTTCTTGCCTAGAGAACCCCAGGGACTGCGGGGCCTGGTGGGCTGCCGTCCATGGGGTCACACAGACTCGGACACGACTGAAACGACTTCGCAGCAGCAGCAGCAGCAGCAGCAGCAGCAGCAGTGCATAGATATCAAAAGGGTTTAGAACAGTCAGGTAGGATCACACAAGTCACCGTGAAGCAATACTTCTCCACTTAGCCAAAGCTAACAA
MSTRG.133982.9	0.03	0.18	0.01699	2.458	up	TGAGGAAATAATGTTCTTAACTTAGTGTGTACTGGGACCGCCTAGTCTCTGGATCTTTTCCTTCTCCTGTGATTTCAGAACGCGGGAGAACAGGCTCCCCTCCTGTTCCTCAAGCCTGTTGTGCACACCAGGATCAGTGTCCTCGCCCCTGGACTCCAGTAGGGGGCAGCCTTTTAATGGCAATAATTCAGATGCCCCACGGAGGGAGGGTATAGTGAGGGTTGAGGAGAAGGGGGGGGGTTGTGGATTAATGAGGTGCACAGATGCTGAGAGGTGGGGGCTAAGCAGGACACCCTTAGATCAGATAGCCAGGGAGGGAGGCTGTCTCCAGTCCCCTGCCTGCCGCCTCCTCCTCTTTACTGGAAAGGGATCCAAATGCAGGGCTTTGAAAGTTTGTGAATGACTTAGGACAATTTTGTTTAATGTGTTCTTTTCTGGAGGATTTCCCTCCTGGAGGGATTTCAGATTTCTCTCATGGGACAGTGTAATTAGGGGATGATTCCCTCACTCTTCAGAGAGGGGAATGAACTGGAGTAAATTTGGTTTCCTTGTCTTCCATTCTTCCCCCTAAACAGACAGTGGGTGTGGTTATCCTCTGCCCGAGGTGTACAGAAGGGGTTCTTTGCCACTATTGTTAATCTGTGTGTGTGTGTGTGTGTGTGTGTGTGTGAGAGTGTGAGTGAGTCGCTCAGTCGTGTCTGACTCTGCGAACCCCTGGACTGTAGCCCGCCAGGCTCCTCTCTCCATGGGATTCTCCAGGCAAGAATACTGGAGTGGGTTGCCATTCCTTTCTCCAGGGGACCTTCCTGACCCAGGGATCGAACCTGGGTCTCCTGCATTGCTGGCAGATTCTTTACTGCCTGAGCCACCAGGGAAGCCCCTGGGTATTGATAATCTGGGTCCAGATGATTCTTTGTTGTGGGGGGCGTCCTGTGCATCGTAGGATTTTTAGTAGTGTTGCTGACCTCTGCTCGTGAGATGCTGGTCGCACTTCCCAGTCATGCCTACCAGAATTGTCCCCCAAGATGTGCCCTGGGGAGCAGCATTGCC

MSTRG.72224.16	0.04	0.21	0.03374	2.441	up	GCTTGGGTTTGTCATCAGAATAGCTTGAACGCCATCATTCTC<br>TAAACTGACCAGACCTTTACTCAAACTGAAACTCCTGTACTT<br>ATCTGATGTTCAGAGAACCTGAAAGAGCTTAGTGTGTGTTCC<br>CTCTAGTTGGACATTTTCAACAAGAAAATGTCTAGTGCTGTC<br>AACACGTGGCCCTGTCCCTGGCTGTCTCTCTAGATCAGCCAT<br>GAAGAGGTTGGGTTCACTCTCCACCCATCACTGAAAAGACCA<br>CACTGTTTACACTTCTCCTTCCAGAGAAATGTTTGCTTATCC<br>CTACTTCTGTTTTCTGGTCCTAATCCAGATCTCTGTCCAAGA<br>TAAATATTCTATTGGCTTCACACTGTTTTATCCTCGTTAAAA<br>ACGAATGACCAGATACCTGCAGTTCATAGGGTGAAAATGTTA<br>ATGGTGATTCAGACTCGGATGAATAAATGAATGCTTGTTTTG<br>CTTTCTCATGTCATAGTTTTAAAAAAGAAGCCACCTAAGTTT<br>ACTTGTGCATGAATGACATATACTTACAACTTCAGAGGGCTC<br>TCAGATGTCTGTTCCTCTCATTTGAATTTTGTGGATGTCAGT<br>TTTGAGAGAAGTGGGCTGACTAGGACTTGAGAGTTATGCATT<br>TCACCCATAAATTGTGGCTGCTTTCATATGGTGTTGAGAGTT<br>GACAATGTTTTTTTAAGTTTTTTAAAATAATTTTTAAATTTA<br>TAATTGAAGGATAATTGCTTTGTAATATTGTGTTGGCTTCTG<br>CCATCCATCAACATTAATCAGCCACAGGTATACATATGTCCC<br>CTCCCTCTTGAAGCTTGCTCCCACCTCCCACCCATCCCACCC<br>CTCTTGGTTGTCCCAGAGCACCAGATTTGAGCTTTCTGAGAC<br>ACACAGCAAATTCCCTCTGGTTATTTATTTTAAATATGGTAA<br>TGTGGCTTTAATTTCCCAAAGCACATAGCTTAGTTTGGGAGG<br>AAGCTCCAATAGTCATTCTCTGCAGCCTTTATTCCATGAGAG<br>TATTGCCCCGTCTCCTAGTAACAAGTTACCCAGCATCAGAGG
MSTRG.215929.3	0.12	0.66	0.02301	2.427	up	AGTTACTTGATAAACGTGTTTATTAGGCGGGTGAGCAGAAAC<br>TTAAGCTTAAAGTCCTCAGCCGGGAGAGACTGCTGAGTAGGG<br>ATGAGAAGCAAGTGGTGCGGGAACCCGGCGCTGCGGGGCGCG<br>AGGACCCCCAGCGGCCCAAGTCCACCCGGACACGGCTTGGGG<br>AGGACGTCGAGCGCCCCCCGCCTGCCTTCTTGTGCCCCCACC<br>CAGTCCCAGCTCTGGAAGACATCGCTGGGCCCGCGGAACCCC<br>TAACGTCTGGCCTCTCGCGTAGTGCAGTGTCCAAACTTTCCA<br>GCAGGCACAGCTCTCCCAGCGCAGCTTTTAACTCGAAGCTGC<br>GAAGAACGCGGAGTCAATTTTGCTGCCCGAAAGGTGCACACG<br>TTTCCATTTCTGAGTTTGCGACTGACTGGGTCTGCAGACCAA<br>AGCAAATGTATGACTGTGCTCCTCTAGTTCTCCATATCCATG<br>ATTATACATTTCTCTTCCGCTTTCCCAGTTTGCACTTGCACT<br>TCGCCAAGCCAAGTACAAACTATCAATTTTAGAAGAGTAATT<br>AAGGGCGGGGAATACACACCTGAAGATGTGGGCGGGAGCGGT<br>GCGGGACTGAGGCCTGGGGATCCTTCTCCGGAAAGAGGCAGC<br>CGGAGAAACTCGCCCGGGTTCTGAACACCTCCCAGTCCACGG<br>CGGGGGGCGGCCACAGGGAGGACGCGAGTGCTCTCCTGCCGC<br>GCCACCCCTACCATCTACCACCCTTGGCTTCCAGGAGTGTTG<br>GCAGTTCTCTGCCTCTGGCTTCACTGCTAAAGCCGCAGCACA<br>ACAGAAGTAGCTGCTTACTAGTAAACACTGCAGACTCTGGCC<br>TGAAACGGCACGTGGGGGCCACGCCAAGGAACTTTGGCAGGA<br>CTGATCCAACTACCTGTAAGAAAGAAAGGCAGAACCGCGAGG<br>GAGGGCTGCGTTGGTGCCGGTCACTCGGTAACCTAGGGCTTT<br>GCAGAAAAGAGAGGTCATTGGAAACCACAGCGAGTGGGGAGA<br>GGAAGACTTCTTGGAAGGCATGAGAACTTCTCAAGCTTCCAG

MSTRG.168241.2	0.21	1.26	0.03299	2.403	up	TACTGGAGTGGTGTGCCATTGCCTTCTCCGGAACATATGTATACCTATGGCCAATTCTTGTTCATGTATGGCAGAAACCAACACAACATTGTAAAGCAATTATCCTCCAACTGAAGATTTTAAAAAATCATACATTTTTTCCTTTAAAAAAATAGCTTTGATGTATAATTGACATAAATTTGTATATATCTAAGGTCTATAGTGATGTTTTGAGCTATGTATACATTGTTAAATGGTCACATAATTAAGCTAATTGACATATTCATCACCTCTACATAGTTGCTATTTGTATGAATGTGTGCTGTGTATGGAAAGATCTAAGATCTATACTCCTAGAAAATTTCAAGTATACAACACAGTACTGCTAAGTTGTTGTCACTATAACGGACATTAGGTCTTGAGAACTTGTTCATCTTGCAAAACAACCTTCATACCTTCTGGTTACAAGTAGTCTCTTGGCAGTCAGATTTGGTGGTTGAGACACGGGCTGGGGAAGCCAACTGTCTGATTTGTCCTGGTGCTACCATTTACTGTGGCTTTGGGGAAACTAACCTCTTTCCTCTTTTATCAATAAAGTTGGATAATTATAAAATCTGTTACTATTGTGAGTCTGAAATGATAAATGGCTTACAACAGTGTCAAAAACAAAGTACTCAATAAATGCTAGTTTTTATTGTCATTATTATTGTTAATGTTAAAAGTAGTAGATGTATAAGAGAAATAACTAAATTTAACATCTCCTGATGGGGACTTAGGGCTAAATGTTTTTCTGCTGCGCATCCTGATCTAATCTGACCAGGGGTAGTGGAGTAAGAGCTTTTTGGCAAAACAACTTAAGCTAATTTACAAGTTCTTGTTCAGTGTTAAGTCGTGTCCAGCTTTTTGTGATCCAGTGGACTGCAGCATGCCAGCCTCCCCTATCCTTCACTGTGTCCAGGAGTTTACTCAGATTCGTGTCTGTTCAGTCAGTGATGCCAGCCAACCATCTCATCCCCTTCTCCTCCTGCCCTCAATCTCGCCCAGCATCAGGGTCTTTGCCAATGAGTAGGC
MSTRG.65420.2	0.05	0.3	0.02933	2.392	up	CAATAAGGGCTTGAAGGAAGTTTTCCAAAGAAGAAAGATTTCCACTGTGCTGCCTTCCCCATTCCCTTCCCAGTACCATTCCACAAACACAGGTTTAAAAAGAAGAAGAAGAAGAATGAAGACTTTGTGAAATAGCCTGTGGACTATTAAGGGAGAGGGGGTAGTGCAGGAAGGAGAAGGGGCAGGTCCACCTATGACATGAGCTGGAAAAGGATTGGCAGAACCTGTCCTGACTTCCCATGTTAAAGACCTGCTGGGGAGTGAAGAAACAGCGGTGCCGGGGCAGTGCTCTGGGCGGCCACACAGCAACGCTCTCAGTAGGAGACGAAGCTCTTCAGTGGATTGGCTACCTTCCACCATGGTGGTGGCTCTGAACTGGTTTAGCAACCTGAGGACCCTGAGAATATCTGGACTGGAGACTCCCTCCTAAAGAATGGCTTTGAGCAATCACAGGACAGGAAGGACTCAGCAGGCCAAGGGACAGAGCGGCATCGCCAAAGAAAGAAGCCCCATACCAGCAAGTCTCACGTGGTGTCTGACATGTAGCAAGTAAATAGCAAATACCATTGAAGGAGCTGCTACAAGAAGCTGTCTCCACCTAGACACACCCCAAGTACCGTCTCAGAAAATGCTCTGTGCGAGAAGCAAAGGCCTGAAGATCCAAGGACCCCCTCATCAGAAATCTTGCGATCTTTTCCCCATAAAAGAATAGAAGGCTACTTGATGAGCTCCACTCTTAATGGTAACAAGGGGTTAATGCTTCCAGTTTCCATCTCAAAAGGGAAAATAAAAATGCC

MSTRG.108023.1	0.09	0.46	0.02613	2.391	up	CAGGGATGGAACTCGCATCTCTTATGTCTCCTGCATTGGCATGTGGGTTCTTTACCACTAGCGCCACCAGGGAAGTGCCACCATTATGGTAATATATGACTATGCTATACAATTATATTAAACTGGATTCTATGATGAAAAATAGTGGGGAGAATCTAGTGAAGACTGAGGGAGAGGAATCAGACAAGCTGGTGAGTCCTGAAGGATGGGTCTGCATTAGCCAGGAGATCAATGAGGAAGAACCTTCCAAGAGCAGGAAACAAGCGTGGATTAGAAAAGGCCACTGTGGAACTCACATCAGGGGAGGAGGCAAAAATGAGACTGGAAATGCCAGCAGAATTCAGATCTTGCCACGTATTGTGGGCAATGCGGGATTCCAGCTTCTCTTAAGGGCACTGAGAAGCCAGTGGAGTATTTTACAAATAGAGGTCCATTGATCAGATCTGCTTTTTTAAAAAGATCACCCTGGGAGCAGGCAGGAATCACACAGCTCAGCAGGCAAAAATCAAGGAGCTGAGCACGCACGAATCTCGCAGCTCAGCTGGCAAAAATCAAGGAGCTGAGCAGAAAAAAAAAAATAAAATAAAAAATAAAAAGATCACCCTGGTTTACTTATTCAGAATAGAGTAGAAAGGGGCAAGAGTGGAAGGAGCAGCTGGGGGCCCAGGGTTTGCTAGAGTGGTCCAGGCAGAGATGAGATGGCAACCAATGGATGGAGTCGAGGGGCCGTCTGGAGGCGAAGATGACCAACGGGCCAGAAGGGGTGAGGCATGATGGGGGCACACTTCCTGTCAGACTTCTGCCCTGAGCACTCTCCACAGAGCTTCCAGCGATGTTCTAGAACCTGAGGCCCCGGGAAGGCCCCTTTCTACTTGGCTGAGTGATCCAGAAAAATATGAAAACTCCATTTATTATGGTTGCTGTAGCCCTATTTGGCCTGAGGACCATGGGTACATCTAGATTAAAAACCTATCCTTAAGCTCACATCAATTGAGAGAAGGGTGAAGCATTAAGAGTTAGGAGAGGGCTGTTGGGCAGGAGGTCTACAGAG
MSTRG.81336.3	0.08	0.43	0.02618	2.361	up	CTCAGCTCCGCTAGCCTCTATTTATAGAGCCCGGAACCCGAAATAGCGCGGAGCCGAGCGGCCGGGATGACGCGGGAGCCGCCGGGGGCTGACTCACCCGGCCCGGAGCGGCGGCCCCCGGATGGGGGACAGTGGAGGCGCTGGACTCGACCCCACGCCATTCCGGGCACCTCTTCTGCTGGGACGTCCCAGGTTTCCACGAGGCACTGTGGTTGGATACGCAGCAAAAAGGAGGATCTGCAGCACGAGAGACTGAGGAGATCTTCTCAACCCAGGGATCGAACCTGCGGCTCCTGTATTAGCAGGCAGATGCTTTACCACTGAGCCATCTGGGAAGCCCAACACCATGTTAGAGATGAAGAAACTGAGATTCAAGGAATACCTTACATGTCCTCAGAACTGTCTCCTGAACCAGGTACCCTGGCCCCGGAGATGTTCAGTTCCCAGGACACTGTGTTTAGTTCCTATTGGACCGAAAGTCCAACCTGCAAAATATCTCAACACACTGAGCACAGAAGCAGAGGTGAAGTCCTAGCTATCTTCTGTCTAGCCAGACATTAAAGTAATTTGTAAAACATTGCCCCTCTT

MSTRG.133252.10	0.05	0.24	0.02217	2.345	up	CCCCCATCCTCGGCCCTGCTCTGTTCTTGCCTCCCTGCCGGG<br>TCGGGATGCGGAGCCGGCAGAAAGTGCACGCGCTCTGCCTGG<br>AGGCGGTCAGGCCGCGCCGGCGCGCCGGGCTCGCTGCAGCCA<br>ATCGGCGCGCGCACCGCGGGTGACCTCCCCCGGGCCCAGCAT<br>AAATCGTCAGGTTGCCCCGCCGCGGGTCTCAGTCTTCGGGGC<br>TAATTAGCAGAGCCGGTCATGAGTGGCGCGGACGGCCTTCCG<br>CGGGCCGGGGAGCGGCGCGGGCGGGCGGCGCGCTTCTCGGGG<br>GCCGAGACCTCCCTGCACCGCGGGTAGACGGCTGGCGCGGCC<br>GCTGCCCGGAGCCGCTGTCGGGCCCGCTTCCCCCGACCTCGG<br>CCGCTCGGTGCCCGCACCCGGAGAGGAGGGTTAGGCTGGCCC<br>GGGCGAGGACGCCGCGCGGCCGCCACCCTCTCCCCTCGGACC<br>CTCGGCTCCGCTTTCCCGCGGCCGAGGCGCAGCACATCCCGC<br>CTGCCTGCGGACCTCAGAGGCGCCTTTCCCGGAGACTCTGAA<br>CTTCTGGAAATCCCGAGACCGCGTCGTGGGTTTTGTTTTTGT<br>CTTCAACTTGGCTTTGGGATTCTCTGGCCACCTGGATACTCC<br>GTTCCTCTGTGGGCCTCAGATGCCAGCTCTGAAAAACTCTTC<br>AATACTGTTATTGTAAACAAAGGTAAGAACCCTTAAATCTGA<br>GTGGCAGCTTTTATTTCCCTATCTGTTCTGTTGTCTTTGCCT<br>TGAATGAAAGCAGAAAGAACAATGGTCACCCCTTACCATTCT<br>GGAGCTCCAGTTCATAATTTGTATTTTTGCTTGAGACAGAAT<br>CTGTGCTCTCATGATGTATCCCAAAACAGTTTATTGTGAAAG<br>AACACATTGAATCCTTATTTAAATTGATTTGCCTAAGAAATG<br>ACTACGTCTCTGTGACTTTCATTTAATGTAGGAAGTGATCAT<br>GGAGAATAATCATTCTCAAGATGCCTTAACTTTATTGTTAGT<br>TATCAACTACCCAGCAGTTGGAGAACGATAAATAGCTGGTTT
MSTRG.145823.1	0.04	0.2	0.02361	2.331	up	CGGAAGAGAGAACAGGGCAGCAAGAGCCAACAGCACGAGGAC<br>GGAGGTGGGGGAGGACCCGGGGGCACAGGCAGGCAGAGCACG<br>GACGGGTGTGTGCCTACTTCCTGGGACAGTGAGGGGCGCACA<br>GGTGGGAAGGTGAGGCCCCCGGGAGACTGGAGACGTTCTGCT<br>GGGAAACGAAAGGTTTCCAGGACATGCACAGTTACCATCACA<br>GGCCAAGGACACCTTCCACCTCGGACACGGGGGCAGACCCCA<br>GACCTCAGACCTCAGCGCAGTGGGGAGAGGTGAAGGGAAGCA<br>GACCGCAGCTCAAGGTGAAAACTGGAATTGGCTTCCCTGGTG<br>GCTCAGACTGGTAAAGAATCTGCCTACAAAGCAGGAGACCCA<br>GGTTCAATCCCTGGGCCAGGAAGATCCCTTGGAGAAGAGAAT<br>AGCTACCCAATCCAGTATTCTTGCCTGGAGAATCCCATAGGC<br>AGAAGAAACCCGCAGCGAGGTCCCAAAGAGTTGGACACAACT<br>GAGCGACTAACACTTTCACTTTCTGACAGGTGGAGAAAAAGG<br>ACAAGCAACAGGATGATTGGAAAAGTAACTCAAAAGCAAACG<br>TCTCTACCACCAGGACATCACTGAAACACAAGGCAGAAAGCC<br>TCCGCAGATCCAGAATGACACGCTCTCAGGAAGCAAATGATG<br>GCTGCCATCACCAGGCACCTCCATTAACAAGCTCAACATCAA<br>CCCTAAACATTTTTTATCTTTTCAAAAACCAACACTGAGGCC<br>AGAACTGTCAAAGCAAGCAGGCATTCACACATTGTGTTCAGA<br>GTATGAATCACTCAAGTTGCTCAGTCGTGTCCGACTCTTTGT<br>GACCGCATGGACTATAGCCTACCAGGCTCCTCCATCCATGGA<br>ATTTTCCAGGCAAGACTACTGGAGTGGGTTGCCATTTCCTTC<br>TCCAGTTCAGAGCATGACCTTGCCGAAATCCTGAAGGCTGGG<br>CAGCAAGGTTAGAGCACATTATTACACACTCCTATCTCCCAA<br>GTGCTCTGGGAAGAAGGTGTCTAGGTTAACTGTCTGGTTAGC

MSTRG.114102.2	0.03	0.25	0.03996	2.328	up	GCGAAGGAAAGCTGGGGATTGCTCTCGAGTGACTGCAGGGCC<br>AATAGACCTCATCTAGGCTTGTGTCCAGAAGCCAATGTTCCT<br>CTCCAGGGGCGACAGGGATCTCGGGGCTGCATTCCAGACGCA<br>CCCGGGGAGACAGGCATTCATCTCGAGTGGAAGCAAAGAACC<br>CCGCTCTGCTCTCGAATCGCGACGGGTATCTCTTGGAGCTCA<br>CTGGGTGGACTCAAGGGAGTCAAGCCTCCTGAGGCGTTTGGA<br>GAGAGGTCGCGAGATTGGTCGCTAGGCCATGCAGGAGACGAA<br>GGCCATCATCTCTCGATGACGGGGGAATCTCGGGGTTGTTCT<br>CGAGCGGCGGCCCCAGTGTGCGGTTTCTCACGAGGTACGACG<br>GCGAGGTCAGTGAGCCTCTCGTGGGGCGCCAGGGAAGTCGGG<br>TCTCCACGCGAGTGGCGAGGGGGAGCGCGTCATTGCTCCCGA<br>GCCATGGTAGGGGAATCTGGCCTCGAGACGTGTTGAAGAAGG<br>TCTCTCGAGGTCTTTCTCGGGTTGAGGCAGGAAACCCTGGGT<br>TCCCTCGACTTGTGCAGGTGACCTCAGGGGGCTTCTCACGGT<br>GGCTCTGAGAAGTCAGGGAAACTGGAGGTGGGAGGGGC
MSTRG.68681.6	0.03	0.15	0.03948	2.313	up	GCTCGCCGACGGGCCGGGCAACCCCAACCGAGCGCTAATAAA<br>ATCGCCGCAGAGGCGCGGCCGCCTCGCGGGCTTCCTTCCTAG<br>GGGGCGCTGCGGAGCGCGGGCGGAGGCGCGCGCGCAGGCGCG<br>CGCGCCAGGCCTCGGGACTCTTTTGCCCTCTCAGGGCGGAAG<br>GAACGCTGTCGGCCAGCGGGAAGGGTCCTTGCCTATCCGAGG<br>CCTCAGCGGCCCGGGAGCGGCCCCCGCCCCTTTAATCTTTTC<br>TGAGCGGGGCGTCGCGGCAGCGCAGCCCTTTTGAGATGCCGC<br>GGAGGGTCGGCCGCCGGGTTTCAGGGCTTCCCCGCCTGCGTC<br>GAGGGGAGTGGGCGTTGTCCGTGGTGAAATAAAAACTGGGAA<br>GGGATCTTCGCCCAGTTGGCGCTTTCTCAGCGCGCTGACCTG<br>TGGTCGAGGCTTGGAGCCGACCTCTGGAGAGGGCAGAGGCCC<br>CAAGCGGAGATCAATACTGAATGGATCAAATCGGAAAGAAGC<br>CTCTCTGGCCAGAAAAAGAAGAAATAACATCCTTAAAGGAGG<br>AACGTGTAAATCAGGAATTGAGCCAAGAAAAGCATGGGAGGA<br>GAAGAAACCATTAATGAGAAAGGAGCAGAAGAAACAAAAGCA<br>GAGAGGGATTAACTGATGGAGCAAGACTTTGGAGATGGAGCA<br>ACAAGGACAAGATGGCTGGATTGGTCTTGACCAAGAGGTGGT<br>ACTCCTCATCCTCTGAGGCTAGAGAAAGCTAAAATGGAGATA<br>CTTATACAGCAAGTTTGCAGGAGTGGTGGTTGGACATTTGGG<br>GTATTTCATAAAGGTATTCTTACCTGTGAAGTAAGGGTCAAG<br>GTCATCTGCTGAGATGGCATCTTGTTACTGCTGTTTTGGAAT<br>TGAGGTATTTGAATTAAACTCAGTTTATTTAAAACCCTAGTA<br>AGAATTTTGTTCTACTTTATTCTATTTACTTTATAAACATTC<br>TACTTTCCACAACCTATGCATTTTAAATTATTTTCATCAATG<br>TTTTGTTATGAAAATTATCAAGCATACAGAGAAGTGGAAAGA

MSTRG.185825.1	0.08	0.42	0.04045	2.313	up	CGCATGCGCATACAAAGTTGTCGCCTGACCAGGAAATCCTTC<br>CGATCTCGCGCGTCCCCTGTGTGAGGAAGGTGAAAGGGTTGT<br>GTTGTTTCTTGTATACTTTAGGACAGTTACTTTAGAAGTCGC<br>TGTTGTTACCAAAACGCCAGGTTCGATAGTTCTCTTCTTCAT<br>AACCTTTTGTTTTCACCTGGTGTGAAACAAAAAGGTGGGTCT<br>AGGTGAAGCTTTTACTTAGAAGCGGCCAAGTTGGAGCACTAT<br>GTCGCAGCAGTTGGCAGGCATACTGCTATCAAGAGGTCTGAA<br>CTTTGCAGGTGGCTCAGTGGTAAAGAATCATCCTGCCAGTGT<br>AAGAGATGCAGGTTCAATCCCTTGATTGAGAAGATCCCTTGG<br>AGAAGGAAGTGGCAACCTACGCCGGTATTCTTGCCTGGGAAA<br>TCCCATGGCCAGGAACCTGGTGGTCTACAGCCCATGGAGTCA<br>CAAAGAGTCCTTATTTAGGACTAAGTGACTAAATAACAACAA<br>TACCTAGCGAGTAAAGTGCTAAGAGATCCCTGAAGCTTCTTG<br>AAATTGCTAGAGTAAAATTTCTTGTTATGGTTAGGGATGGGA<br>ATGGACAAGTTTGTAAGGGCATAATAAATTTTAATTTCAAAG
MSTRG.129912.5	0.05	0.27	0.01814	2.296	up	AATTTATTATCTGTTTATTTTTGGCTGTCTCAGGCCTTCTTG<br>CTGTGAGCGGGGGCCGCTGTGTCATGACAGCGCTTGGCCCCT<br>CACCGCGGTGGCGTCTCACAGGGAGAGGGGCTCTGGGGTGTC<br>AGCGTCCGCGGTCGTGGCTCTCAGGGTGTGGAACGCAGGCTC<br>AGTAGCTGTGGCCCACGGACTCAGTTGCTCCAGGGCAAGTGG<br>GATCTCCCTGGATTAGGGATCGAAGTCATGCCCCCTGTGGTG<br>GCAGGCGGATTCTTAACCACTGGACCACCAGGGAAGCCCCTG<br>GTCCCGTCATTTTAGAACCACATATTTGATGTCTGGCCACCG<br>TACTAGTATGGAAGTATAAGTTGAAAAAATGTAGGGCACTTT<br>TTTGCCAGTGTACATAATAAAAAATATCTAGTAATGTTATTT<br>CACTGGACACAGTGGAGATGTTTGAAGTTATTAAGTCCATGT<br>TCTGTCTATGTCAGTATATTTACGCTTGTGAATGGCGCTGAT<br>CACTGAAGTACCCTCTCCTCTCACAAGAAGCATCTTCCAGAA<br>ATCACCTCACCCCAGATTAGCTGGCCGATGACCACAGTGACA<br>TGTGCTTCTCAGATAACACATGACATTCTATTTGGCTCAGAA<br>CTTAGTGGCTGGCTCTGCTAAAAATAAGTCTTTAATGGTTGA<br>TTAGGACATTCATCACGGGGTGCTAATATTCGGGAGTTTTCT<br>TCGTGTCATGAAACATATTTATGGACGGCCACAGAGTGGATG<br>CACCTCGTTTCTTGGATGAAATGTGATAAGGATTTCAGAGAC<br>TCTCTTCCCGGGAAGGTCATTGCAGAGTACGGAGCAGTTCCC<br>CGCGCTCTTTCGGGCCTTGTTGGCCATCTGCTCAGCATGCGG<br>TGGTGCCTGTTCCCAGTTCCCAGCTTACCCTCCCCCGCTCTC<br>CCTCTGGGAACCACAGTTGTTTCCTGTATCTGTAACTCCATT<br>TCTGTCTTGTAAATATGTTCCTTGATACCGTTTTTTTTTTTA<br>GATTTCACATATAAGCAATATCATATTTGTCCTTCTCGGCCT

MSTRG.199116.1	0.03	0.17	0.04375	2.293	up	GATAAATACATGGGTTGTTAAGAATGCGCTGTTTCTTGAAGT<br>ATCTTTTACTGATTATATCCTAGCTTTGTATGTGTTCTGCTG<br>GCTGTATGCTCTAAGCCCTTTGTTCCTTGTTCCTATAAAAAG<br>GCTTGACTGCAGAAATAAACTTGTCAGTCCTTGCAGAGACTG<br>TCCAAGTGTTCTTTCAGGTTCACCGTTTCCAAGTCCCGGAGA<br>CATATCTCCAAAAAGTGGCGGCCGAACAGGGACTTGAAGGAC<br>GACTGAACAAAGCGGCGAGCCCTGCAGAAAGAGATGGAGAAG<br>AAATCTGGGTTCCACTTCGCCATGTCCGTTACCGAGAAGGAC<br>CCCAAGACCAGATTCCCCCGACAGGTGATGCAGCTGACGCGA<br>AGGGTCTCATCAATGACCATAGAGAAGCCGAAGAGAAAACGT<br>CAATGTCTGAACCCCTACCATAGGACCTGAGGAATGGCATGT<br>GTGGAAGATTCCTGCTGCACTGGAGCACTTTGCCAATGTGTA<br>TGAGACTGCGAAAGCCTTAAAAACCTCAAAACTATACCTTTT<br>TATACGATAGTACAGGTTATACCCAATCTTGTGTGCAGCTCC<br>CGTACATGTTTATTGTAGGTAACTTCGATATAAATCTCTTGG<br>CTAAGATTGTTAACTGTACAGCATGTGCCTTGTATACGTGTT<br>TAAACCATACTATTTCATATCATTATGTCAACATTGCAATAG<br>TCAGGCAAAGATCTGATTTGTGGCTTCCTGTTAACCTAACAG<br>AACCATGGACTGACTGTATTTCTCTCAGTTTTGCTCAAATAT<br>GGCTTAAGGAGGTCAAGGCGCATAATAGGATGGATTATTGCT<br>GGCATTCTTAGTCTAATATCTATTGTTACAGTTGGAACCCTA<br>TCTGGTATGGCACTACACAATTCTATACAGATGTCTCTACTC<br>AAGACTCCGTGCCTCCTACGACAGAGTCCAACTAACAGCTGC<br>CATGCTTAAACTAAAAACAAAAGGAGGATATGTTGGGAGGCA<br>CAGTGCAAAATAGCCTTGAAGGAGGAGGTCCGTGGGAAAATG
MSTRG.225378.1	0.05	0.25	0.01685	2.283	up	GAGCCACCAGGAGGAGAGGGAGGGAGGGAGGCAATGGGCCCC<br>CCAGATGTGAGGGGAAGGAAGGAGGGGGGAGCCCACCTCCCT<br>CGCCGCCTGAACTGAGTAATTGGACGGAGACCCACTTGGCGG<br>CAGGAAATGGAACAGATCCAGGTGACCCATGAATAATCCCGA<br>GTCGACTGTAACTTTCATTCTCGGGGTTTCCTGATGGGAATA<br>TAACAAAGTTCTGGACTCTGGAGGACAAGAAAACAAGAAAAA<br>GCCCTTTTTATTAAAAAAAAAAAAAAAAAAAAGACTTCTTTA<br>TTCACAACCTCACTGCCCATGGAGTGAAATTGATCACTTGTG<br>TAAAACATTTCATTTTCTGGCAGGGAAGTTCAAACCTGGCTG<br>AGGGTTATAGCCCTTTCTTTGTGTCCAGGGATTTCCATGTGC<br>GATTTCTCATGATGTTAATTATCTTTCTATCAAAATTTGAGT<br>GTGTCACAAAACATTCTCGAAAGCACAGGATTCTTTTCTATC<br>CAGTGGCTGTAAGAGGAGTCACTCTGACACATGCCTTCTTTT<br>AGATGATAACTGGCACTCTGTGGACAAAATAACCACGATGAG<br>GGAAAAGCCACTCCCCTCCCAGGCTTGTCGCCTGGGGGAGAG<br>ATTCTGAGTGATGGAGAGGTAACAACACCACAGCACCCAATG<br>CAGGGGAGGCCAACGGTCCCTTGGCCGCCGGCCACCCTGGCT<br>TGCAGGGCTGTGGGGCCACCATCGCCATGGACACTCCAGCGC<br>TCTCAGCGTGGTTCAAAGGCCGTGTGGTGTTGAATGGACACC<br>CATCTCAGGGCCCAGAACTGAGGAAGCCACAGAAACCTGTAG<br>GAAGAGTAATAGCAGCACTCCTTTGGGCACGGGGCCTTGGGG<br>TTGATCGTCTCTCGTGCTTATAAGAGGGTCTACACGAAGCTG<br>GTTCTCAATGAAAATTTGTTACAGGAAGAGTACCTGTTTCCT<br>GCCTCTAGGACAGCTCTGTGGCATAGAACTTTCTGTTGGGTA<br>GAAATATTCTTTATCTGTATTGTCCTGGTGGCGACCACCTGT

MSTRG.205178.3	0.04	0.23	0.02504	2.245	up	TGCCCTTTCGACCTCCAATTCCTAACGTGGGACTTCTCCTGAGGCGCTGTAGCGGGAAAGGGCTTCATCTTGCGATGGCGGGGGAGCCACGTGGTTTTTCTCGAGTTACGGCGGGATTCTCGAGTTACGACTGGGAATTCAGGCTGCCTCTTGTGTTGGCCCAGGCAAGTCCAATCTTCCATTCGAGTTGTGAAGGAAAGCTGGGGATTGCTCTCGAGTGACTGCAGGGCCAATAGACCTCATCTAGGCTTGTGTCCAGAAGCCAATGTTCCTCTCCAGGGGCGACAGGGATCTCGGGGTTGCATTCCAGACGCACCCGGGGAGACAGGCATTCATCTCGAGTGGAAGCAAAGAAGCCCGCTCTGCTCTCGAGTCGCGACGGGTATCTCTGGGAGCTCACTGGGTGGACTCAAGGGAGTCAAGCCTCCTGAGGCGTTTGGAGAGAGGTCGCGAGATTGGTCGCTAGGCCATGCAGGAGGCGAAGGCCCTCCTCTCTGGATGACGGGGGAATCTCGGGGTTGTTCTCGAGCGGCGGCCCCAGTGTGCGGTTTCTCACGAGGTACGACGGCGAGGTCAGTGAGCCTCTCGTGGGGCGCCAGGGAAGTCGGGTCTCCATGCGAGTGGCGAGGGGGAGCGCGTCATTGCTCCCGAGCCATGGTAGGGGCATCTGGCCTCGAGACGTGTTGAAGGAGGTCTCTCGAGGGCTTTCCCGGGTTGAGGCAGGAAACCCTGGGTTCCCTCGACTTGTGCAGGTGACCTCAGGGGGCTTCTCATGGTGGCTCGGAGAAGCCAGGGAAACTGGAGGTGGGAGGGGCCTCTCGGGACTCCACTGGGCTTGG
MSTRG.163094.1	0.05	0.24	0.03481	2.221	up	GCTGCTGATAAGGCTCCCTGGAGCCTCAGCGTGGGCTGAAGCATTTTCCTGTGCTTTCAGACCAGCAATGTAAGTAACAGATAATGGCAGCATCCTGACCTGCGAGAGGCAAGGGAGCCTCACCTCACATCCTTCTGGGGGAGATCTGGGTAATCTGAGCAGGCAGTCAACTGAGACCAGCCAAAGCGAGTGGTAGCCAAAGGCGCTAGGTCCTCTGGGGTCCTGTGGTCAGCAGCTGTTGCTTGATCGTCTACTTTGGGGCTTGCCTTTGGGGCACCACCTCTTCCATATAGGACTCCATTCATCCCATTGTGCACACCCAGCTGAGAAGGTGGCTCAGTGGTAAAAGAATCTGCCTGCCAATGCAGAAGATGCAGGAGACTTGGGTTTGATCCCTGGGCTGGAAAGATCCCCTGGAGGAGAAAATGGCAACCCACTCCTGTACTCCTGTTGGTGAATCCCATGGATAGAGGCGCTACAGTCCATGGGGTCTCAAAGACTCAGACATGACTGAGCAACTGAACACACACACACATTTCCTTACTAAAAATAAACATTAAAGGTCACTGCTTTTATGGACTCGACTCTTGAGAGATTTCCAAAATGGAAACTCACTGAAAATCTGGTCCTACTTTGTCCTAATCGCGGGCTCTCCCAGCTGATGACTCTCCACTGCAAAAATGCACGTAGGCACGGAAGCTATTTTCCAAAGAATCTAAGCTGAAAGTTCAGTTGAACTGACTCTGTACCACCATGGTGTTTAAGCATGACTCAACCAACGGATAAAGTTAATGGAGTCAAAAAACGTATAAGACTCTTGCCAAGGGTGCCAGTGAAAAGGAGTCCCTTCTGGAAGGACACTCAGAGAAAGCTTGGGACCTGTCCCCGAGGCTGCTGAGTGCTGTGTGTGACCCCGGACAGCTCAGTTGAGTGCCTCTCTCCCACCAGTGGCAGCAACACAGCGGGCTGCCCTAATCACGGTGGCTGCAGCAGAAAGGCCCTAGAGTGGGCAGCGTTCTGGAGCAGCCAAGGTGAGGTTTGGGCAGAGAC

MSTRG.31739.1	0.04	0.2	0.03132	2.184	up	GCCAGCTGTGGTTCCAGCTGCCCCAGCTGAGCCAGGCTCCCAGGCAGCCGCTGAGCTGACCACAGCCCACTGCCATTCTCAGCTTCTCCTGTCAATTCTGGGAGTTTCTGCAGTCATGCCAGCCCCATCTTCTCCTGTATGAGATGCCAAAGTTCATTTAGGCTGTTGCCACCAAGCAAAAAATGTACAGTGTCTGCCAGCACCCCTGGAAGACATTGGGTGCACCTGCCCGGAATCCTCTCAGTCAGTGGCCTGAGGTGGTGAGTTCCCTGTCGAGAGAGGCGGACACGTGCCAGCTGGAGGGCTCCTTGACAGGAATGATGCCTAGATTTTGTGCGGATTCAACAAACAGCTTAGATAAAAGCCACTGCAGAAATTAACACAGCACTGTGTGGATGTGTTGCTTTCTTAAGGAAGAAAAAGTGAGAGAAGTAGGAAGTGAAGTCCAGATCTGTTTTAGCCCCCAGGTCATTTAGTTGTCATAAAAGCTGGAGTTTTTCATGGGTGTATTTTTTGTGTGTACTGTGAATGTGTTTATCCTTCCAAGGCCAACGTTCATAACAGAACCACCTTCTATTCCTCGGCTGATTCCTATGGGGAGGGGATGGCAAGGGGCAGAGCAGCGACATGACCTCCATGAGGTCACAGGTCGAGGCTGTGCAGGAAGTGAGACACAGAGATGCAGCGGCCTGACAACTTCCAGGGGGCGTCATCAGCGGGAGCATCACCCATGGGAGACTAGCAGTGGGCGGTGCGCATCCCAGAGCAGGACCTGCCCTTCCTCCCGAAGCTTGGCTCCAGCCTGGGTCGGGGCAGGATGCCAGGCTCGACTGTCTGCCAGCTCCCTCTTCCCTCGCTAGGGGACCCTTCCCCTGCCTACAATCCCACTGCCCACCTGCAGATAAGGGACTTGCCCAGGCCACA
MSTRG.50900.10	0.09	0.42	0.02955	2.167	up	CCCTCCCCAGGCGCCGAGGCCCGGCCCCGCGGCGCTCCCCTCCCTTGCCAGCTCCCCCCCGCCCCCGGCCCTGCCCCCAGCCCGGGAGCCACCGCTTCCCCGGCGGGCGGGCAGGGGGACGTGGGGACCGCCCGGCGCGGCCGTGCGGCGTAGCGGGATGCGGGTGGGCGGCGAGTGTGCCCAGGGGCGCGCGAGTGCCCGCCTGCCTGGAGACCGAGGCGCGGGCGTGGAGGGCGGCGGAGGCTCCGGGATTTAGGTATTCGCGTCCACATCAGAGCCTGCTCCCTCTGAAGTCTTCTGTATTCTTCTAACAACCCTAGAAATTCTTCAACCCGGAGCTGTGTACTCAACAGGCAGACGGCTCAGCCTGTGTATGTTGTCTAAGTGTCTGGAATGTACACCTGGTTCCTCTCCACTGGCACCCACAATTTAAGAAAGTAAATAGACGCTTTCAAGGAGGAACATGGGCACACCAGGATCAGGAGCTACTGTAGACATTTCCGTCTATTTTGGGGTTCTGTTTGATATGAGAAACTGTTATTGCTCAGATGTCTTCCTTGGAAAGACCCTTCATTAAAAGAAAGACTCCAGCACCAGATGTCTGCTATTTAATTTCAACTAATCAGTCCTAACTCGACTCTGCCACTGACTCTCTTCATGGGTGAAAATTAGTATCTAATTTTTGGTAGCTCACTGAGCAGTTTTTGGCAAAGTAACCATTCAAGCCATGCTTCCATTTGTCTGCTATTGTCCTGGTGGGATGTTTCTCCAGTCTCACTGATGTGTTCATTAATGTCAGTGTTCACGTTCAATGGTGGTGATTGGCGAAGACAGGGAACAAGAACCAGGAAGAAGAAGAGGCGCTGACAGTGATGGCCCCGCTCATAGGAGCAGTAGAATAATGCTGCAGTTTACTAGCTGGGGTTAGTAATGTCCTCATTTTCCCTCTCACTCTGAATCTTTGCCCTGGATAAGGGGGAGTTGAATTTAGCTTTAAGAAGGGATTAGTAAGAAGAGAATGTGAATGTTGGTTTGACAGGTGGGGTGGGG

MSTRG.43630.39	0.13	0.59	0.0418	2.138	up	ATCAGTGAGCTCCGGGCAGTACTTTGGTCCCAGGTTTGGAGCCCAATGCCCGGAACGCGGGAGAGAAGAGGCTGGCTGCCAGCGGGTGCTTGCAAGGCTGTACTGGGAAGGACCGAGTGAGCTTCCACACCGGCGGAGCTCCAGCTCTGCGCCCAGGCGCGATAGCACAGAGCCCCGGCCGAGAGGCTGCTCTGTGGCGGGCGAGCCTCCCCAAGGCCTGCCCAGCTTCTAGCGTTCGCGCCCGGGAAGGAGCAGGCTGCGGGAGTGTGGGAGCCGTGAGTGGTGCCTGACTGCTGCCTCAGCTCAGCTCTCGCCTTCCCTTCGGACCTAGGAGCCAGGCGTCCTTGCAGAGGGCTTAGGGGCTGGGGGTGTGCACACTGCCAAAGCCTCCCGCTGGGAGAGCTCTTTCCGAGGGAAAGGAAGCGCCCCAGGCTGCATTCCCAAGCTGGGGCTTCCCCGCCCAGACTAAGCGCCGCACGGAGGGAATCAGGGTCCCGGTGCCCCTGGCTTGGGGAGGCAGCTCCACAGCCAGAGTGTTGCTCCGTTTGTGCCCAGAAAGCGCCTGCCAGCCCGGGCCTTCCCTGGAGCTGTCCTCCCGCCTAGAGGCAGGTGGATGTGCTGTGCGGGCTTCCCCTGGGCGTTTGGGCTCCCTAGCACAGCCGCAGCGCAGGGGAACCAGCCCATCATCAGTGAGCTCCGGGCAGTTACTTTGGTCCCAGGTTTGGAGCCCAATGCCCGGAACGCGGGAGAGAAGAGGCTGGCTGCCGGCGGGTGCTTGCAAGCCTGTACTGGGAAGGACCGAGTGAGCTTCCACACCGGCGGAGCTCCAGCTCTGCGCCCAGGCGCGATAGCACAGAGCCCCGGCCGAGAGGCTGCTCTGTGCTGGGCGAGCCTCCCCAAGCCCTGCCCAGCTTCTAGCGTTCA
MSTRG.25405.1	0.08	0.35	0.042	2.097	up	GAAATCTTTTGTTAGCTTTGGCTAAGTGGAGAAGTATTGCTTCACGGTGACTTGTGTGATCCTACCTGACTGTTCTAAACCCTTTTGATATCTATGCACTGCTGCTGCTGCTGCTGCTGCTGCTGCTGCTGCGAAGTCGTTTCAGTCGTGTCCGAGTCTGTGTGACCCCATGGACGGCAGCCCACCAGGCCCCGCAGTCCCTGGGGTTCTCTAGGCAAGAACACTGGAGTGGGTTGCCATGTCCTCCTCCAATGCATGAAAGTGAAATGTGAAAGTGAAGTCGCTCAGTCGTGCCCGACTCTGAGCGACCCCATGGACTGCAGCCTACCAGGCTCTTCCGTCCATGAGATTTTCCAGGCAAGAGTACTGGAGTGGGGTGCCATTTCCTTCTCCCATATCTATTCACAAGCCTGCCTAAATCATTGACTTCTAGCCACCTTTGGGATGCCAATGACCACCCAGCCTTACCAGGCCACAGAGGAGAGAAAACATTTACCACAGGAGGTCCGGGTAAGGAACAAGGAACTAACAAGCTCCCAGCAACCAGGATTCTGCAGAGGTCAACAAGAGGTCAGCAAGAGATGGGAG
MSTRG.91649.1	0.05	0.23	0.04533	2.095	up	TTTTGACTTAATCAGCTCTTTCAAGCCTTATTTCCAATTAAGTTGGAAACACAATATAGTTGTGTTTTGAGGTATGGGAAGTAAGCCTTCAACATATAAATTTGGGTAGGAGGACAAAAGTCAGCACGTAACACATGGTAACACTATATATGTGAAGTGCTTGTATATCTCCCCTACAGATCCAATGGGAAAAAAAATAAAAATATAATCTTTTCCAGATTACAAAGTAGGAAAAACCACTAGACCATTTAGGTATGACCTAAATCAAATCCCTTACAATGAAACAATGGAGGTGACAAGTAGATTCAAGCAATTAGATCCAATAGCAGAATGCCTGAAGAACTATGGACAGAGGTTCATGTACAGGAGGCAGTGATCAAGACCATCCCCAAGAAAAAAAAAGGCAAAATGGTTGTCTGTTCAGTTCAGTTCAGTCACTCAGTCATGTCCAACTCTTTGCAACCCCATGGACTGTAGCATTCCAGGACTCCCTGTCCATCACCAACTCCTGGAGTTTACTCAAACTCATGTGCATTGAGACAGTGATGCCATCCAACCATCTCATCCACTGTTGTCCCCTTCTCCTCCCTCTTTCAATCTTCCCCAGCATCAGGGACTTTTCCAGTGAGTCAGATCTTTGCATCAGGTGGCCAAAGTATTGGAGTTTCAGCT

MSTRG.167273.1	0.06	0.28	0.04869	2.094	up	TTATTTATTTCTACTTATTTGGTTGCAGTGGGCCTTAGTTGT<br>GGCCTATGAGATCTTCAGTTGCGCGTATCAGATCCAGCTCCC<br>TGACCAGGGATTGAACCCAGGTCTCCTTCACTGGAGCACAGA<br>GTTCTAGCCACTGGACCACCCAGGAAGTCCCCCATGAAAAGT<br>ATTTTAAGCTAAAATTATTAAATTTAGAAAATTCAAATCAAA<br>GACTTTTATTTCCAAGCAGAGACTTTCTCTTAATCCACCATT<br>CAACTTTCCTAGCAATTTATTATTTTCAGATAATGCAGAAAA<br>GGAAAAAAAAAAAGACCACTTACTTTATCTACTCATGAATGA<br>ATCCCTAATTTTCATCATAGCCATTCAAAAAGTTTAAAAAAT<br>TAAAACTGTTAACAAACTAAGGATATACTATACATCGCAGGG
MSTRG.46945.1	0.05	0.22	0.04988	2.083	up	GGGCGGTGTGGGAAGGACAGAGCAAGGCGGAGATGGAGGGCA<br>GCAGGGGCCAGAGCGTGCAGAGCCTCGCAGGCCTCAGGGAGG<br>ACTCGGGGCTTGATCGCAATGGTAACAGGAAGATTTGCTAAG<br>TCGCTTTAGTCGTGTCCGACCCTGTGCGACCCCATAGACGGC<br>AGCCAACCAGGCTCCCCCGTCCCTGGGATTCTCCAGGCAAGA<br>ACACTGGAGTGGGTTGCCATTTCCTTCTCCAGTGCATGAAAG<br>TGAAAAGTGAAAGGGAAGTCACTCAGTCGTGCCCGACTCTTA<br>GCGACCCCATGGACTGCAGCCCACCAGGCTGCTCCATCCATG<br>GGATTTTCCAGGCAAGGGTACTGGAGTGGGGTGCCATTGCCT<br>TGCTTTGAGCTGGATTCAAATGGACTAAGACATAAGATTCAC<br>AACCTCCAGAGAGTATTACTTTGCTACACAGAAGAAACCAGC<br>ACAACATTGTAAAGCAAACCGTACTCCAATAACGATTAATAA<br>TAATAATAACCCCTCCAATAGCTAGCAGTTAAACCACTTGGG<br>TATTCATTCCTTCCCCCAGTAGTTGAGCGCCTACTCTGTTCC<br>AGACACTCTTCCAGGCACTGGAGAGAAACAAAGGGGGTTGGA<br>TACAACAAACTCAGACTCTGCCTTCCTGGGGCCAGAGGGGTC<br>ACAGACCCTACAGCAAATAATCATACGGTCAAACACAGTGAT<br>CAGAGCCACCAGGGAGGGCTTCTCCGAGGTGATGCAAAGGCA<br>GCTGGTTAGGTGCTTCATTTGGCCGGTGTACATGGAGCAGCA<br>AGTGTGTCAGAGACTGTGCTGGGTGCAAGGGGTGTCGCACGA<br>GCCAGGCAGACCCAGTTCGTGCCCTCGAGGCGTGGACAGCCA<br>GTGGAGGAGACACACCAGAAATCCCGTGGACAGAGGAGCCTG<br>GCGGGCTGCAGCCCAGGGAGCCGCACAGAGTTGGACATGACT<br>GAGTGACTCACACTTTCACATTCATCTCTGGAGGGGCGTCAC<br>TCTGCTTGCCACAAACCTGGAATCCAGGCCCAACCCAGCCTC

MSTRG.169420.1	0.05	0.22	0.03115	2.077	up	CAGCGCTGAGAGCGGCCGGTTGGAGCCCTGCTTACCTTAACT<br>TCTGACGAGCTCGGGCTCGGGCGCAGGCTCGGGCTCCGGCCC<br>CCGGCCCGGCTCGGCGGCCCGGCCGGGTCCCCTCGGCGCGGG<br>ATGGGCACCGACCCTCAGCATCGGCCGAGACGGCGGGCGGGA<br>GGGAGACCCTGAGGCCCGGACGTCGCGGTGCAGAGCTTGGCC<br>GCCTCGCTGCTCCGGCACGGCTAGCCCAGGGCGGACTGGGGC<br>GGCTCGGACCCCCCCTCCCACCCACCCCACCCACCCTCCCCT<br>CCCCCACACCCCCGCCCCCTGCCCTTCCCGAGGGCAGCAGCC<br>GCGGCCCGGAGATAAGCGCTAGCGCGGCGCGGGGCTCTGCCT<br>GGGCTAGTGGCACCGACTTGGGAAAAGAAAACGACGAGACAC<br>AGAAAACCCGTGCTTTTCCCACAGGAAGATCAGTGGATCAGT<br>CCGGCTAAAGAAACCGAGAAGCCCAGCTGCCAACGATGGTCT<br>ACACATTTGAAATACAGCAACTTCCCTCTTGCCAAGGAAAGG<br>CAAGACGATAGGAGAAACTACAAGGCGTCGAGGCACGTTTTC<br>TGCGCGCAGACCTGAGGCTGACCACAACGGCTGAACTCCCAG<br>GCTCCGCAGGCGCCGTGAACAGTCCACAAGGAGAGCGGCCCA<br>CGCGGCTACCGCCGCGCCCGTGGCCTCTACTAGTAGGCTTTG<br>GCCCTGCTGTTCCGTGGAGCGGTTCGGTTCGTCTGAGGCATG<br>CCCGAAGAAACACACACGCCCTTCTGAGCCCCCCACTCGCCC<br>CCGCCCCCGACGAACACACATCTTTGGTTTCTGCAACCGCCC<br>CTCCTGCACGAAGCCGGTCTGGGGTAGAGCTTTGTCGCCGAA<br>GCCCTCGCTGGCAGGGCGCGCAGCTGAGGGGAACTCCTCAGA<br>AGTTATATATTGACCAGCACCTCTTTCAATACTGACCAGCTA<br>GCTGCAAGGGGTGGCAGCTGCCAGGGAAAGTAAACTTCTTCA<br>CCGGTGCGAGGGCCGGCGCGGTGAAGAGTTGGGACGCAGCGC
MSTRG.140737.2	0.04	0.2	0.03455	2.057	up	TAGTTTTCCTTCCAGTTAAACTGATTTCCTCATCTATTTATT<br>TCTGGACTGAAGCCAGAATCAACATTGTCCGTTTTTCTAATC<br>AAATAGTGGAGAAGCAATTACAAAGTCTGAGTGCAGATAGAG<br>GAGCCTGGAAGGCAGAGCAACTGGACCTCGCCTAAGCTGTGT<br>CTCTGACTCATTTCTGACTTTAGTTCTTCCAATGGGAAAAAT<br>GGCGAAGATGTTTAGTTTCATCCTTGTCACCACTGCTCTGGT<br>AATGGGCAGGGGAAGCTCGGTAAGCTTATTACAAAAACTATT<br>ATATTTTTAATTCCTGCTTTGATCTTAATTTTCTCTTGCAAA<br>AAAACTGTAGTAACTTGTCACTGAAAAAACTAAGGAGGAAAA<br>CTAAAAAAGATTTGTGGAAGTACCTGCCACATAATAGGCATT<br>ATTAAGTGCTTTAAAATTTTTTTAGAGTAAAAAATCAGTATT<br>TACACTGTCTTTTTGATTTACTGAGGTTATTTGGGTGGTTAC<br>ATGGTATTAAGAATTTAAAAGCTCTTTCAGATTTACCTTGAG<br>CTTTGTAATATTGGACACTGTCCATGTAAACTAAACAGATTA<br>TTTGAAAGTAATTGCAATGCACTTGTGCCTCCCTCCATGAAT<br>AAAAGTGTGAAAACACTCATTTACTCAAAGTTGTAGGAGAAA<br>GAAAATCCCTAATTTTTTTAAAGATCTTTTTTTTCTTTGATG<br>TGGACCATTTTTAAAGTCTTTATTGAATTTGTTACAATATCA<br>CTTCTGTTTTATGTTTTGGCTTTTTTGACCACCAGGCACGTA<br>GGATCTTAGCTTCCCGACCAGGGATCAAACCTGCAACCCCTG<br>GTTTAGAAGGTGAGATCCCAGCCACTGTACTCCAGGGAAGCT<br>CCCCCACTAAGTTTTAAATAAGTTTACTAACACGGTATATAT<br>AGTCACATAAAAATTGAACATTCTTGCCCTGTAATCCTGTAG<br>TCACTTTAAATTGTATAGTTCACTGTATTTAACAGTATCACA<br>ACCAATCTTCCACTGTTAGAAAGCAAGGCAGAATTCCAGATG

MSTRG.81595.3	0.08	0.31	0.04347	2.051	up	CCCTGTCGGTTGAGTAGAGCAGTCAAAATTTGGCAATCGTTT<br>TGGAAGGAGCTTGGCCGGCCATGGTCCATGGGGTCGCAAAAA<br>GAGTCGGACACGACTGAGCGACTTAAACATCAACAAACAACT<br>TCGGTAGGATAAGAGGAAGCACTTCCAGAATAACACGGGGAG<br>TGGGGTGGTAGCTGGCTAGACAGGTGAGCCCAGGAAGGCCCC<br>CCCAGCACTTTTGTTCTCCCCCGGGAAGTAGGAGCTAAAACA<br>TCCGCCCGGTGGACGTCGGGGACCCAGGGGGGCAGGACCCTC<br>CCAACTCCTGATGCCCTGGCCAGCGCGGGGGACTCTTTCTGC<br>ACCATGTAGCATACTGGACTACAGCCCTGCCCGACCCAGGGG<br>CGGGCCAGGGCAGCCACTCCCGACCCAGCCCTGGCGTAGCCC<br>CGGGGAGCACTGATGGAGACGTGGACTCCTGGGCCCCTGGCT<br>GAGGGGCCAGGGGAGAGAGGGCTGACGGACTTGGCGTCTGAA<br>GGCGGCGGGGACCAAGGGGGCAGGGCCTGAGCTGCCTCCCTC<br>TGCTGCCCACCACCATCTCAGGAAAGGCTGCTGGTGCTGGCT<br>GCCCATTCCAGCTGCAGCGGTGACACTGGGGAGCGGGGGTCT<br>CTCCCCCCGGCGCTCCTTCACTTTGGGCCTGGCCTCAGGCCC<br>CTGGTGCTTCCCCCCCTCCTCCTGGGGAGGGGGCCCATGAAG<br>AGCAAATGAGCCAAACGTGACCACTAGCCTCCTGGAGCCAGA<br>GAGTGGGGTGCGTCCTGACGGGTGCCCCGGCCCAGCGCCCAG<br>CCATCTTCCCTGAGCCAGCCGGTAGGTGGTGGGCATGCCTGC<br>CTCACCTTCATCTGGGGGTGGGTGGCCAGGAGGGGCCCAGAC<br>TGTGAATCCTGTGCTCTGCCCGTGACCACCCCCCGCTCCCCC<br>CCATCAATCCCATTGCATAGGTTTAGAGAGAGCACGCGTGAC<br>CACTGGCATTCATTTGGGGGGTGGGATATTTTGGCGGAAGCC<br>ACCCCAGCCTTAGTCCCCAGGGCGAAGCGCTGGGGGAAGACG
MSTRG.221949.8	0.04	0.16	0.03974	1.964	up	CGGAACTTGGGAGCAGATCATAGCTGCTAGGTAAGTACGCGT<br>TGTTGGCGGTCGTGCGCGCGAACCTGAGTGGCGCAGCTTGGG<br>TTGGGGAATGCGATACGGTAAAAAGAGTTCAGAAGTTGAGGG<br>TTAGAGTGCGGCGTTGAGTATTTACACATGGAAAACGCGCCA<br>CCGGACCGCCACGTGTCCCCTGTCGCGGTGGCGACCGGCTTC<br>GAGTTCAGGCGTGCGTAGGCTGCTGCTGGTCATGGCGCTGCG<br>CGCGGCCGTATTGCTAAGCCGGCTGGATAATCCTGAACGGCG<br>CACGGGAAACGACCACATTCCTGAGATTTGGGGCGGGAGTTA<br>CGCTTAACGTTGACTTACAGGCCTTAGATACGTAGTCATATC<br>AACAGTTAATTTAAAAATCGGCAGCTCCTGACTGCGTGAAAC<br>TGAGTTGTATTGTGCCCAGGTGGCGCTGTGTGCAAATCGAAT<br>TGTTGGAGGGGAAGGTTTTAGTGCTGAAAACCTTAAGCTATG<br>ACCTTCTATGACAATACTTAATTTTTAAATATATTTATTCTT<br>CCGCAGGTTAAGAAATTGATTCTCCCGCAGAAACAATGGATT<br>TCGGTGTAGCGGAACTGTTACGTGGAGATGCTAAGGTAATTG<br>TTGAGAGCTTAGTTTTAAGTTGAAGGAATAAACCTGTTGATT<br>TACAGGTTGATCAACCTGTAAAGATGATGCATCATTTGAAAA<br>TTTTTTTTTTTTTAAGATGCAAAGGTACATTTCAAAACACG<br>CCGGACAATATTGGCTGGAAAATGACTTGGTTAAAACCTGAT<br>GATCTTACCAGCATTCTGCAGGTTGATGAATACTAATGAAGC<br>TGTGGATGTCACTGAGCAGCTTCATTTTAAAGTAAGTTACCC<br>TGACTTACATCTGCTGCAGTAATTCTGTCTGGTTTGTGGTAA<br>ATGTCCGTGTAATGTAGCATAATGGATTTTGGTCTGATCAAT<br>GATGAACCCTATCCCGAAGCTGATAACCTGAAGAAAAGCATG<br>TACGGATTCGGCTTCTGAGACAAGACTAATGTTCAGATATGA

MSTRG.46782.1	0.05	0.23	0.04481	1.959	up	CTTTCACAATTAATGTATGGGCATTTAAACGAGCTGCAGCCAGCCACGAAACTCCATCAAGAATCCATGCAGGACAACCGGCTTGACCGCGTGGCCATGCTTACCTCCTCTCAGGCCAGGGGTAGATGGCACAACTGATCACGTTGACTTGTGAGCCACAGTCAGAAGATACGTGGTTTGGACAGAATGAAGAACTGCCTCAGGCCTCGCAAAGCCCAGAGAGCTGGAGCCTTCCTCCATCTATTACCAAGACTGTTGTTTTTTTGTTTTTTTTCCCCCAAACTTTTATGTCGTCTTCAAGAGAGTGGGCTCTCTCTCTCTCTCTCTCTGGGAAATGATTGAGTCACAAACAGTGTCAGATGCTGCCAGGGAGCTCTTTCCCCCCACCCCACCCCCACCCATCTTCCTCAGTGCTCAGGGATGAGATCATTTCATTCGGAGCCAATTATAGAAGGAGAACGCTGGGGATGTACAAAGAAACCTCCTCCCACTAACACAGGCTGTACCCTGCCGGCATGAGCAGGAACAGGAGGCTGGATGTCCGGTTCCAAGTTCAACCTCAGGGTGCGCGAAGGCCAAGCATTCCTAAGCCGCAGCTTGGATCTGTCAAGCTAGGATGCTGGGCTTCTGGCTAAATGAGATCTTTTGGTCAGAAGTTCAGAGCTGGCAATACTAAACTGGGAGGAAGAAGATCCAGAGATGCTGGTGTCCATGTCTGCCACCACGAGGTGCCTGCCTAAGGAAAGAGCCGAACGGAGGAGCAGAGGACCAAAGGGTAGCGATCAGACAGCTCTAACCCCACAAGCCCTGGATCTAGCCTCACCTGAAGGGAGATCTGTCCTCAATATTTTAATTACATGTGTTGATAAATTTCCATTTT
MSTRG.68246.1	0.06	0.25	0.04715	1.934	up	TGCCATGCATTGGTGCATATGTGTACGTAGTCTTCCTACATGAAGGGACGTGCTTGTCGCATGACCATCTATCCATATGTAGAAGCTTTGTTTTCTCCCATTAGTAATTAACATTTGATTGCATCAGTTGGGATTATGCAACTCTCTGACAGCTGACACTAGTTCATACCTGTTTCCATTCTTTCTCCCCTCTGATTCAGCATCTTGGCTCCCGAGAGGTGGTTGTATTATAATACTTTAGAACCCACTTGGCTGTGTTCCTGACTCACTGGGCGTCCTGTCATGGTTCTCCATCAGCGGGACCAGTAATTGCTTGTTTTTCATTCATGTTTTGCCTCCTTATGATGAATAGCCTTAACTTTAAGCATTCCAAAACCATTGATTTGTTGGCAAACTGTTTTTGTTTTTTAATTCTTTGGGGACTTGGCGATAGTCTGGCTTTTCTTGGGCTTCCTGCAATATTCAGTGAAATAACAGTGACAAGGGCAGCGAGAGAGAACAGCCGCAGGGCGGACCACACGCCAGGCGCTGGGCAGCCCCCAAAGGGCACTAAACGCCTATCATTCCTCCAACGGCTCTCTGAGGGCTGGGACTCTTATCACCCCCCCATGTTACACATGGAGAAAGTGAGGTGGCTTTGAAGTAGCTTGTGCATGGAAACGAGTCCACATCGGGGCGAGGCTCACTGACGGGGCTGGAGAGACTCGGGACAAGGCGACGCGCCCAGGGAAGCCAGCGCTGGATGGCGCAGACCCGGAGGGAGAGAGTGGGCAGAGGTGCTTGGCTGCTGCCTCTGCAGAGGAAGTGACACAGATGAGTCCTCCCATCCGAAAGCCCTGGACAGGGAACTCAGGGCAGGAAGGAAGCGGAGGAAAGGGAGGTGTGGAAACTGGGCTGAGGGAGTTCACTTGCTGCAGCCTCTCACGAAGAGCCCCTGCTCTTCATCAGCTTTGTCCCTTCAGGGTGTTCATCCCAAGGGCAGGGGGGTGAAGCAAGTGAAAGGTATGTTTACCTTCAGCCAGTTTTCTTTTTTTTTTTTTAATTTTCACA

MSTRG.91984.1	0.03	0.14	0.04408	1.906	up	CTCTCACATCCATACATGACTGTTGGAAAAACCATAGCTTTG<br>ACTAGGCGGACCTTTGTTGGCAAAGTAATGTCTTTGCTTTTT<br>AATATGCTGTCTAGGTTGGTCATAACTTTTCTTCCAAGGAAC<br>AAGCATCTTTTACTTTCATGCCTGCAGTCACCTTCTGCAATG<br>ATTTTGGAGCCGCCCCCCCCCCGCCCCCAAATAAAGTCTATT<br>TCCACTGTCTCCCCATCATTTGCCATGAATTGATGGGACCAG<br>ATGCCATGATCTTAGTGTTTTGAATGTTGCACCATTTCTTGG<br>TACCAAAAGTAAGCAAAAATAATACAAGATAACTGCAGACCA<br>AACTTTCTCTTAAAGAAGGTTATAAATATTTTTAAGAAACAT<br>TTGTTGTTTAATAAACTAGTAAATAAAAAGGATAGTACATCA<br>TAACCGGTGACTAAATGGAGTTTATCTTGGGAATGTGGGGTA<br>GATTTAAAAATTATTTAATTTGCTAGAATTTAATAATAATAT<br>TTGGGCAACTTCTTGTGTACTTGTAATTATTAAATATAATAA<br>TTTATTAGAAAAAGGGAGGAAATTATGATAATTATTTTAGAT<br>GTAGAAAAAAGTTTGGACAAAGTTAATATTTATGTGTGATAA<br>TGGCTCTAAACAAATTAAAATGAAGTTCCCTGTGCTGAAAAG<br>GACATCTGTAAACAAGTCACAATTAAGCCATCAATACAGATG<br>AAAGTCTGACTGTTTCCTTCTAAGTTTGAGAATAAGACAAGA<br>ATGTCCACTGACTTTGATTCAGTGTTGTACTACTTGGTAACA<br>TAAATTATAAACAATAGATACAAGGAACAGGAGGCTTACATC<br>TCAAAATACTATGCTGAGAAAAGCATCTAGACAAAGAAGTAT<br>ATCTGTAGTGACAGAAAGCAGATCGGGTTTTGCCTGAGGCTA<br>GGGATAGAACTGACTAGGGACAGAAACAAAGGACCTTTTTAG<br>AGTTATGGAAGTGTTTTGTATTTTGATGTAGTCACCATCAAG<br>TTGATATATACATTTGTCAAATTCATAAACACGTATGCTTAC
MSTRG.133381.7	1.06	0.3	0.04493	-1.87	down	GGGCCCAGCGGGACCCTCCCCCAACTTGAAGCGGGGAGGCGC<br>GGGCCGCGGTAGGCAACGAGCGGCACACGGCCCCACCGCGGG<br>GCCGGCGCACCCGCCCCCCACCGCCCCCAGAGGGGAGGTGGG<br>GGGCGGGGTGTTCTGCCCACGCGCTCTTGGCAGCCGCCACGG<br>CGGCCACTGCGCACGCAGGAGGGGCAGCAGCTGGGGGGTCCG<br>ATACCCCAAGGCTCCCTCTCGGATCGCTAGAGAAGGCTTTCT<br>CACCGAGGGCTCGCCGCCCCTCCCGCCACTCCCCCCTCCCCC<br>CTGGCTCCCTCGCCTCGCGGGTCTTCCGGCGCGCGTGCCGCG<br>TCCGTGGCCTCTCCGCCCCGCCCCGCTTTCCCGGCACGCCCG<br>GCTCCTCGTGCTCGCGCCCCTACCTGGTTGATCCTGCCAGTA<br>GCATATGCTTGTCTCAAAGATTAAGCCATGCATGTCTAAGTA<br>CGCACGGCCGGTACAGTGAAACTGCGAATGGCTCATTAAATC<br>AGTTATGGTTCCTTTGGTCACTCGTATGCTTGTCTCAAAGAT<br>TAAGCCATGCATGTCTAAGTACGCACGGCCGGTACAGTGAAA<br>CTGCGAATGGCTCATTAAATCAGTTATGGTTCCTTTGGTCGC<br>TCGCTCCTCTCCTACTTGGATAACTGTGGTAATTCTAGAGCT<br>AATACATGCCGACGGGCGCTGACCCCCTTCGCGGGGGGGATG<br>CGTGCATTTATCAGATCAAAACCAACCCGGTCAGCCTCCTCC<br>CGGCCCCGGCCGGGGGGCGGGCGCCGGCGGCTTTGGTGACTC<br>TAGATAACCTCGGGCCGATCGCACGCCCCCCGTGGCGGCGAC<br>GACCCATTCGAACGTCTGCCCTATCAACTTTCGATGGTAGTC<br>GCTGTGCCTACCATGGTGACCACGGGTGACGGGGAATCAGGG<br>TTCGATTCCGGAGAGGGAGCCTGAGAAACGGCTACCACATCC<br>AAGGAAGGCAGCAGGCGCGCAAATTACCCACTCCCGACCCGG<br>GGAGGTAGTGACGAAAAATAACAATACAGGACTCTTTCGAGG

MSTRG.199472.1	0.38	0.09	0.04847	-1.89	down	GGGAAAGACAGCAGCAGGTGTGGAAAGCAGACCTCATCCTCCAGGAGCCAACAGAGCAGAAAGGAGCGGCCGGCTGAAGTGCACCGCAGCAGGGGTGAGGGGAGGGCTCCCCCCACGGCTATGCCTCCAGCCACAGAGTCAGGGAGATGTGAACACCTGGATGGGCTGACTTCCTGTCGGGAAATCTCCCTGGCTGCCTTCACCAGTCACAAGTCTGCAGCCCAGATTTCCTCTCACTCTGTTGTACGACCCTCACCTGAGCTTCTTCCTGTGCTTTGTCTGAATCCAAACACTAACTGGATGCTGTTGGGACTGACCATGGGAGCTGATGGAGAAGAGAGACATTAGCTAGGCCAGTATGATTCCCCTTCTCTCAGGAATATAAACACAAAATCTGGAGAGAGGCTGCCCACTGGTCGGGGAAGGCGAGAGAACATGCATGAACAGGAGCTGGTGAACCACGCAGGCTGAAGATGACTTCTGACTCCGCTGCTGGAAGCCTTTGAGCCACTGGGGACACAGGAACGCCTCCCAGACCCAGTCGCCCAGAGCAATATCCACACTGGAGTTTGGGTTCTGAGGTTTCTGAACAATTACACAAGGATCCAGAAAATAAAACCCCTTGTTTGAGCTGATGTTGCTATGTATCTACCTCTCACAATGGCAACAAAAGATACATCCAGGATAGTATAGATATAATACAAAACAAATATG
MSTRG.198307.9	0.43	0.11	0.0497	-1.98	down	CTGAAGAGTTCATGCAAAATAATTTGGAGTGCTGGAGGTAATTGGAGAGACCCCTAATATCATTAAAGGTCTGAAAGATGCCAGGTAGGCAACTATTTTGCTTTTCAAATTTTCCTTGTGGTCATTTTAAATGATTTTGAGAGACTAACTACAAATTGCTGCTGATGTAATAAAACTGTTCCTGCCATTGTTAATACAGGACTTGAGATTGTTTCAATTCTGGAAAAGCTATCCAATATGCCCTGTAATATTAGCAACAGATTTGGCCAGTGCATTTATTTCTACATCTGTACATAAAAAAATCACCACTAGCAATTTGCATCTACCTGCGCAGGACAGTAATATATTTTCACTGTCTTGTCTCAAGGGTGTGTAAATTTTCTTGCTCTCTGTCACACTGTTGAAAGAAGGGATTTTAAATCACATGGAAACTTGAAAGCTTTTCTTTCACCTATTACAGAGATGATGTATTAATTAGGGGAAATAGGAGAGCTGTTTTCTGTATTTTCTCCTCCTTTATTGAGTTTATGACCTTCAGGGGATGGCTGAGTAAATACAAGAAGGCTCATGAGCCCGTAGTCCCCTCTGTGGGTATTCTACTCTTTCAAGCCTAGAGAGAAGCACCCATTTAGACTAAAACCAAACTCCTCTGGCTTAAACCTCCCTCTTATGATTATAAATTATTTTACAAGACTTTTATTGTTAGAGCTGGGCTTGGAAAGGGGCACACATTAGGATTTTCCTGTTTCCTTATTAGGAATAGGTTTTATCCCCAGCCATTTCTGCAGCAAAGTGATTTGAATACTGTATAGCATTACCTCTTTATTGATTTGTAGCTGTTTCTGCTTGTTTTATATTCTGTTGATAGTGCCTGAAAATGTCTGTTATTGTGTTAGGGGTGCTGGAGTGGGATCAAGAAAGAGGTGTCAGTTCCCGAGCTCCCAGCAGTGACTGTCAGCAAGGGACAGAAGGTGAATGCAGCTTGTTAGGCGTATGGCAACAGCAAAGAAGCAGGCTTCTATGTAACCTGTTAGGGATGGGTAAGAAATG

MSTRG.53403.3	1.09	0.28	0.04362	-1.99	down	GGGCTTCACTGAAGGGCCCGGAGGGAGGAAGCGCCGGGGTCG<br>GCACGAGCGTCCCCGAGCGGGCGGGGCCCGTGAACGGCCGCT<br>TCCCGCAGGCAGGCTTCCACGACGCACCGGCACCTCGGCGAG<br>CCTCCTTCCCTCCTTACGGGTGGGCTGGGGCTTCGTAGTTTT<br>GGGCTGCGAGGGGAGGCTCCCTGCCCAACGGGAAACTAAATT<br>CCCAGAGACGGCCCTACCTGGAATAACAAGGCATGAGAGGAC<br>CCCAGGGTATCTGAGGCCTGGTTCATGGGATTCTTTTCGGAG<br>CGTTGACTTTGGAGCATGGTTTCTGTCATTTTCTGGACATGT<br>CTCAGGGCCCAGCAAGAGAAAGGCGGTCTGGACAGCAGCCAG<br>AGGAAGTTGTGATGAAGGAATTATTGACTCGGTCGAGTAGGT<br>GTGTGTGCTCACTTTTTCTGAAAGAAAGAAAGTGAAGTCGCT<br>CAGTCGTGTCCGACTCTGCGACCCCATGGACTGTAGCCTGCC<br>AGGCTCCTCCATCCATGGAATTTTCCAGGCAAGAGTACTGGA<br>GTGGGCTGCCATTTCCTTCTCCAGGAGATCTTCCCGACCCAG<br>GGATTGAACCCGTGTCTCCTGCATTGTAGGCACACCACGTTT<br>TCTGAAGGGCTTTGAAATCAGTCTTTATTTGCCCTGAATAAG<br>AAAAGGGTAGAACGCACAATGCGAAAATGGGAAGATCCCTGA<br>ACTTTCTGTGTCAGGACCTTCATCCAAGTCACTTCTGCTCCG<br>TGAGCCTCAGTTTCCACTTCTGTTAAGCTGGATTGGGGTGGC<br>GTTGGCAGTCTTGGTTAAAGGGTAGACATGGCAGGGGCTGCT<br>TCACTTGTTTCTGTCCATACGTAATCTCCTCTTTATGGGAAC<br>TGAGTCACTGATAGTGGCTTCTGAAAACTTCCTTGGAGATGG<br>TATCAGGCAGATCATTGCACTTATCCTGCTGCTGCTGCTAAG<br>TCGCTTCAGTCGTGTTCGGCTCTGTGCGACCCCATAGACGGC<br>AGCCCACCAGGCTCCCCCGTCTCTGGGATTCTCCAGGCAAGA
MSTRG.186142.1	0.42	0.11	0.02735	-2.02	down	AGTATAAATAATATATGAAAATAAAGTTGTTTGTACTTTAAG<br>GTGCATAGAATACATTTGTTCTAGAGAGAGCTTAAAATAGAT<br>TCTCAATTAATTATCATTTGGACAATGAAATCAGATGCTTTC<br>TGGTGCTCTAATTTATCACAGCCTTGGAATAAATTCAATTTA<br>GTCTGTCATCTTATAGTTGTAGTTCATTGAATTCTTGATTAA<br>ATGCACACTTAACAAGTACTTTTAAAAGAAAACTGCCGCTAA<br>GTCACTTCAGTCGTGTCCGACTCTGTGCGACCCCATAGACGG<br>CAGCCCACTAGGCTCCCCCGTCCCTGGGATTCTCCAGGCAAG<br>AACACTGGAGTGGGTTGCCATTTCCTTCTCCAATGCATGAAA<br>GTGAAAAGTGAAAGTGAAGTCACTCAGTGGTGTCTGACTCTT<br>CACGACCCCATGGACTGCAGCCCACCAGGCTCCTCCATCCAT<br>GGGATTTTCCAGGCAAGAGTACTGGAGTGGGGTGCCTAGTGA<br>TACCATATTTTACAGTCAAACTTGGATGAAACTAGTTGTTTC<br>ATTGTTTTAATATATTATAAGAATATTGGGTACCATACTCAT<br>AATAATTCAGTTTTATTTGTAATTTTGATAAATACTCATATC<br>TGCTCCAGTTTGGTTAGATACTTAGTTTGAAGTTGAATTTGT<br>TCTTAATTGATCAATACTTGGCTATAGCCTGACAGTCTGTTT<br>CAAATGGTGTGTGTGTGTGTGTATACATTTGCCCTTATTTTG<br>TTTGGAAAATATTATTTTAAAATATTTGAGACCAACAAATTA<br>AAATATCTGAGTATGTGATTGCATTGATCTGGACTCTGTTTT<br>ACAGCTGAAGAATTCTTGCAGTAATGACAAATCGATTAAAAC<br>ATTTAAACCATCATATTGGATGGCATAACCCCCAAAGTTTAT<br>GAAAAGTTGCTATTTCTCTGGACCCTCCAAGGTGAATGTACA<br>ACCTGAGACTATAAATGCATGTAGCTTAATGCCTGTGAACCA<br>TCAAAGGAAAACATTGTCCAGATTCTGAGTGAAGTGAGAATT

MSTRG.186150.6	0.3	0.07	0.04201	-2.05	down	GTTCAGATTGGAGGTTGGCTTTGGGACCTGCCTTTGTTAGAA<br>GGTGTCCTGTCTTGGGGAAGGGAATGCCTGACTAAATGTGGC<br>AGGACACAGGAATGACCTTCATTTACTGAATGATAAATGAAT<br>TGAATGATAATAATATCATTTCGGATTCCAGGAAGTGACAAT<br>ATTAGTGTGTAACAGAATAGGAAATAGAAGAGGGAAGGTGAC<br>TCAGATACCAGAGCAGAACTAGCATAAGAAAGAAGGAATGGG<br>CTCCAACTTAGAATATCATTTATGCTCTGGTGCTTCCTCCGT<br>GCCCAAGTGAAAAGAAATCAGTCCTTTGCACTGCAGAGTAAT<br>AATAGCTATACATATGTGACTGCTATGTGAGAAAAGAACCTT<br>TTAGAAGTTGCTAAGATAGCATACTTGAAGCAGGTTAATTTG<br>TTCACTAGACATTTGTTGGGCACCTCTATGTGCCAGGGTGTT<br>CCCTGGTGGTGCAGCCGGTAAAGAGTCTGCATGCAATGCGGG<br>AGACCTGGGTTTGTTTCCTGGGTTGGGAAGATCCTGGAGAAT<br>TCCATGAACAGAGGAGCCTAGCAGACTACAGTCCTTGAGTTT<br>GCAAAGAGTTGGACACAACTAAACGACTTTCATTTCACTTCT<br>GTGTGCCAGGCTCTGTTCCCACAACTGGGTGCGGCATTAAAA<br>CAGAGAAAACCCTTTGTTCTCTGGCCATTTACATTGTGGTAG<br>GAAGCAAGTGTCCTCAACAGAGCTTGTCCCTGTTATTCTGGT<br>GACCACATTAGGAGCATTGCTTACAGGATGATGGCTGAAGTA
MSTRG.43306.1	0.63	0.15	0.04299	-2.12	down	CAGCGCCAAAGATACAGGACGCTCAGAGACAATACCGCCCAG<br>CCTGGGTGCAGCACTCTGCCCTCACGTCTGGAGGTTCTCAAC<br>GCTTTGTGAACAAAGGAGCCTCCATTTCCACTTTGCTCTGAG<br>CCCCACAACTCTGGGGAGTTTTTCACGGCAAGAAAGAGAAGG<br>TGCCAGCTTTGCACCCAGCCGTCTGGCCTCGAAGCCCAGCCT<br>TCTCATCTCAGCGGCTCATGAGGACCTCTGCAAGATGGAGGA<br>AACTGACCCCGGAGCAGACCCGGCCCCAGCCAGCTCAGTATC<br>AGTCCCGGTCTCTGCTGGACTGGCCTTCACGATCCAGAACGA<br>GTTCAGGGCTAGATCAGAGCTTCCTGGCGTCGTGGCTGCAGC<br>TGGTGTGGTGAAAGGGCTGGTGCTTGGAAAGGAGACGAGTGC<br>TTGAGCAGAGGATTCTACCTCTCGTGTCCCATATCTTCTGAA<br>TCAGAATCTGGGGGTGGTGCATGCTGTGGGGACCTGGGAATA<br>TGTGTTTCTTAAGAGAAAGGAATGGAATGAAAGAAAAAGCAC<br>TTCTCCAGAGATTCTGATGGAAATCCAGCCTCAGAAACTATC<br>CGTTCAGATGACAAGACTACTGTCAAGCCTGCTATCCATGTC<br>CTCGCTGAAGACAGAGCTGGAGGGCACACATCTCTGAGCTGC<br>ACACCTGGGCAGAGCGGTGCACACAGGAGAAGAGCAGTGTTC<br>TCGCTGTGGCTCACGTTTCAGCAAGTGGCCTCTTTCTCCACT<br>TACACTGGGGGAAGGATCATTCAGATGGTGGGACAGGTCAGT<br>CTGAATTTTACCATGCTACAATTTCCCAGGGTGGTGAACAGA<br>AACATCTGGAAAGAAAACAGCTGTTTTGGGCAATTTTCCCCC<br>CAAATCAAATCTTCACAGACGGAGAACATGACCACCTTGTGC<br>TGCCATATTCCTGAACTTTAAGGACTGTCTGTTCTGTTACTT<br>TTGTTTGAAACTGTAAGGAAATAAAGTTAAGAACAGTGATGC<br>TATACATACTGACAGTCTGTCTCCCTGACTGAACCTGAGTTG

MSTRG.210622.4	0.11	0.02	0.03043	-2.14	down	CCTGTGGAACTGGCTGAGTGGCAGTGTGTCCCAGGAATACTC<br>CTCAGGGTTCAGGTTTGGTGACTGTCACTGGAGGACTCAGGT<br>AGGGTCATTTGTACCTCGTGATGGACAGCATTACCCTTTCTG<br>CTGAAAGCACAAGGACATCCTATACTTGCAACTGGATATAGG<br>GTAATAACCACATGAACAGAAGATGGGAGACAGATACTCTGT<br>CTCGAGTTTCTGCATACTTCATTAGATGCATTTGTGAAGAAT<br>TTCTGGCCTTTGGGTCTGTTGCCATTTGTAAGCACCCATGAG<br>TAGATTTCACTTACTGGAACCTATATGCTTTCTTGGTTTTCA<br>AATTTGATGGCAGAGGGAAGAAGAGGCAGGACAGCTGCCTTC<br>TTATCTGATTTCCCCCTTGCAGACAAGGAAGCAGCCACACTC<br>CAAATCTACTGCATCCTAGACCTCCCCAGAAGCCAGGAAATG<br>GAATTGGAGCCAGCCTCACCCTCTTTGGGCCAAAGAGATTGT<br>GGTAGAGCGTCCGGAAGCGCTTGCGCGTGTAGTTGCAGCGAT<br>AAGCCCCTCCCATCAGGTACTCGATCACCAGGCCAATATCGA<br>TCAGGCTGATTCTGTAGTCTGGGGGCAGGTTCCCCTACCAGG<br>GAAGAATGGAACACACACACATCCAAGAGACAAGGGGGTTTA<br>CTAGGGATTCTACTCCGTGTGTTCTGTAACTATTTTAGACTA<br>AAATAATAATAATAATTAAAAAGAAACATATTAATGTATTAT<br>TATGCTGGTTAATCAATTCACCTTAAAATAGATCCAACCGAA<br>ACCTGGATATTCTCGCTTGGAAAGAAGACATAAGTTAAATAA<br>GAAGATGCAGTAATCGTCGGCAATAACAGAAAATCAAAATAT<br>TTATTCAAGTCTTCAGCTGGGGAGAAAAAAACACCAGTGTAG<br>CAGAAAGAAACACAAATGGCAAATTCTACCACTCCCAGCCAC<br>TAAGGAACTAGGTTCCCAGATGCTCCCCCAGACCCCTTCACT<br>GCTCTGCCTCGGACCCGGGCCTGTTACTGGCCTCTTGCTGAT
MSTRG.26889.3	0.55	0.13	0.03102	-2.14	down	TGCAAACGGGGACTGAATTAAGCAGCCTCCAGGGAACGTGGG<br>CTCCCACGTACAACAGCTCCTGCCATGCCCCGCTGGGAGGGG<br>GCAGGCAGGAACGTGCCTGGGCCTCGGAATAAGCAGCAGACA<br>CTTGTCTGCAGGCCAGGTCGCCTGGATGCAGACAGAGTGGGC<br>AAGCCCTCTGTCTCCGCTGCTCCCTGATCACCCACCCGGGGC<br>TCGGAGGACACCAGGTACCTGCCCAGTCGTCAGCCAGAGGCA<br>GGGAGGAAGTTCTCATGTGAGAAAAAGGACGGTTTGGACTCC<br>AGAATGGACGGTGCAGACTGGTCTTTCTGTGCAATCATCACA<br>AGCAAACGCAGAGGTGGCAACATCCCCAGGGTCCATCAGCAG<br>GGATGGTAAACTCAACGGGGTACTATCTACACAGCAGAATGA<br>TACTCGGCCTCGAAAAGGACTGGAATGCTGGTTCCTGCTACC<br>ATGGGTGAGCCTGGAAAACCTTATCTGAGTAAAGAAGTCTGT<br>CTTTGAAGGTCACTAACTGTACGGTCCCTGCACAGGAAAACC<br>CAGAAACGGGAAACCTAGACACAGGAAGCAGAACAGTGGGGG<br>CTCCGGAGGTGGGGGGAAGGAAGTGGAAGACTGTTTCAGGGT<br>GACGGAAACTTCTCAATGATGACTATGTGCTGCTCTCACACG<br>CCTGCATGAACTAGAAGCCCCTGTGTGCCCTCTGGGATCCCA<br>AACCCAACTGTTAGAACTATTAAGACATCTCTCTCCACAATT<br>TCTCTGCTCGCTCACCTCTGAACCAGGCTAAGTGATTTCTCT<br>CCAGGTTGACCCCTGGGTCATTAGCAGACCTGCATTGCTGCT<br>GCCAGGAGGCTGGGTACCAGGTCATAGGAGCTCACCTCGCTC<br>CCTCTGTCTGAACTGCCCTTAATTTAAATAACAAGGCTGCTG<br>CAGCTGAGGTGGCATCTCTCAGAGGAAGGTCATAAATTAGCA<br>ATTCATAGAAGCAGGACACATGCGCACATGACCTGGAGTGAG<br>TCCAGCACCTCTGTCAAGGATGAAGCTCCTGGGTCCCTGCTC

MSTRG.46959.6	0.39	0.08	0.03738	-2.27	down	TCAAGACGCAGAGACTCGGAGGCGGAGAAGAGGAACACAGAA<br>CCAGAAGGAAGGGACCCCGAGGGCGGGGGAGGAAGACCCAGG<br>ACATGAAAGGGGAAATTGAGGTGGAAATTGAGAGACCCAGAG<br>ATGCAGAAAAACAGCAATAGAAAGTGGTTTAGAGAAAGCTGC<br>GGGCCAGGCAGATTCTTTACTGATTGAGCCACCAGGGAAGAT<br>CCAGCCCCCAAGGTCAGTAGCATGGAGGCTGAGAACCCTGCT<br>GTAAGTGGAACAGACCTTTGAACGTCACTTTGTATGGCATAA<br>GGAATTATGCAGCTATGATTTAGCTAAAGATCTTGAGCTGGG<br>AAGATGATCCTGGATGATCTGAGTGGGCCCAATGGAATCACG<br>GGAGTCCTTATAAGATGGAGGCTGAAGGAGGTTTGACCACAG<br>AAGAGGAGAAGGCAGAGAGAATGACAGAACCAGATTGAAAGG<br>AGGCACATTGAAGAGGGAAGAAGGGGCCACAAGCCAAGGGAT<br>GCAAGTCACCACTGGAAGTTGGAGAAGACAAGGAAACAGCCT<br>CTCCTCAGAGCCTCCGGCAGGAGCCAGCCCTGCCAGCATCTT<br>GACTTCAGTCCAGAGACTGATTTCAGACTTCTGACCTCCAGA<br>ACTCTAAGTAAATAAATTTGTGCTCTTAAGC
MSTRG.34606.1	0.19	0.04	0.04543	-2.27	down	GCTTTAAAGGCATGAGCTCTGATTTAATATCACTTAGGCAGC<br>CACCAAAGAACATAAAGAATGAGGCTGCTGACCTTGTTCCTG<br>ACAGACATTTTTCGGTTTTCATGTTCCAAAAATTATTAAGAA<br>GGGGCTTCTATCAACATGACTTTTCCTGAGTCTCTTGTGTTT<br>ATGAGGAGAGCTTTGCTGATGTACTCACGATTTCAAACTGGA<br>AGAGCTCTCTGAGAATTCAGCTGCCAGAGATAGAGAGAGAGA<br>GAGAGAAGACTTACCCTCAGCACCTAGAGAATGTGAGAATTC<br>TGCTGCCACAAATCCCCCTCCAGGGACCTTCCAGCCAGGCAG<br>GATAATGACCATTCGCACCAGCACCTGGATGAGAATTGTGAA<br>AATGTGTCACAAACCTTATAAGAACCTCCCACACTTTCCCAC<br>AGAAGCTCTGACCACCAACATGCCCTTATTATGCTGATAGTA<br>TATAAACCTTTACCCATGGCTGTTCAAAGAGATTCTCTTTAT<br>AGAATGCTGCTGCATGAGTGTATGATAACATTTTCTCTTGTT<br>ACTCTGAAGAAAACTCCAGATTGTCAATTAATTCGCAGGTCC<br>CAACTACCACCATTCAGACCTCACTTGGTCAAGGAAAAGTTT<br>TCCTGCCAGGTCTCCAAAATCAACAAGCCACTGAAATGACCC<br>TACTCGCCCTCACCTCCAGGATTCCTGCCATGAGAGAATAAA<br>CCACAGTTTGTTTAAGCCACTATTGGTTGGATTTTCTGCAAC<br>TTGAAGCAGAAAGAAGAGCCAGTAGTTTACCCTGTTCATTCT<br>TTTTGAGCCAAGACACAAGTGCCCACTCTCCATTCCCCAAAA<br>TGATGGGAAAAGTCATAGTTCAGACTTGGATGCCAGAACAAT<br>CACAAAAGAAAGTGGAACCTTGTCTGGTCAGATCTATGTTCC<br>AACTCCTATACATCACTAATGTAGATCAGCTTCTCACACTTT<br>TTCTTTGCACAACATATATCAACTCTGGATTAATCTGGTAAA<br>ATGAATAAGACATTTTTAAAACACTGCCATGGCTTTCAAAAA

MSTRG.143209.3	0.47	0.09	0.01943	-2.28	down	TGATCTTGGGTGTGTGTGTGTGTGTGTGTGTGTGTGTGTGCG<br>TGCGTGCGGGGGAGGGGAGGGGCGCTACTATCACTGATAGGT<br>ATGTTCACCAATCTGTCGAATGCCAATATAAAAGTCAGTTCT<br>TGGTTGCTGAAGGGAACTGTATGACTTGGTATTAAAAGAAGG<br>TATGTGGCATGAAATCGCATTTTCTGAAGGAAAACGACGTCG<br>TTCTTTCCTCCAGGTGGCAGTTTCAGGATGGGAGAAGCAAAG<br>GAATGGGTACAGAATGGGATCAGGAGGTTGTAGGGAACGTGG<br>ACCCCCAGAAAAGAGTGTTGTGCCTAGCACAAGGTTTCTTGA<br>GAGGTTGAACTGCCTTTGCTAATGGATTTTAAGTTTCTTTAC<br>CTCTTCCGTGATCTCTTCTAGGTTGACCTAGAACAGAAATCT<br>TTTGTTAGCTTTGGCTAAGTGGAGAAGTATTGCTTCACGGTG<br>ACTTGTGTGATCCTACCTGACTGTTCTAAACCCTTTTGATAT<br>CTATGCACTGCTGCTGCTGCTGCTGCTGCTGCGAAGTCGTTT<br>CAGTCGTGTCCGAGTCTGTGTGACCCCATGGACGGCAGCCCA<br>CCAGGCCCCGCAGTCCCTGGGGTTCTCCAGGCAAGAACACTG
MSTRG.14510.5	0.33	0.06	0.01719	-2.3	down	CCCGGCTCTCCTGATCGCAAACTCCGAGCCAGAGCTCTCCGC<br>GCGCGCCCCAGCCGCTGGCCGCCCGCGCAGCCGGACGCGGAA<br>CCCCGGCGCAGCGGGAGCCTTGCAGGACGGGCGCGGGGCGGC<br>GGCGGCAGCCGTCCATGCGGCGGGCTCGGGGCTGCCCGGCGC<br>CGGGAACCACGCGGGGGCCAGGCAAGGCGGCCACTGGCCGCA<br>CCGGGACTCGGACCGCCAGGAGTTGAACATCACCCCCATCTG<br>CAGAAAATTGGCAACATCTCTGAGGAGCCCTTGCTTTTTCCT<br>GGGACACCCAGGACCATGGTTTCCTGTGTCATGGAGTCTCTC<br>AGTGTCCTGGGTTCCCTCCTGCTGCTTGGACTCCAGCTCGTC<br>TGCCCGCAGCCCTCCACTGAACACAGAAAGTTGCCAGGATCA<br>TTGTCATTGTCATTCACCTCAGAGAAGGAAGATTCAGTGCTT<br>TTTTGGGTCGCAAATTTCTTCCTGACGTTTTGTTGGAGTTCT<br>GTGAAGCCGTGGCTGTCATCCTTCCTGACTGTCCCCAGAGCC<br>CTGGAGCAGCGACAGTACTGGCAGTAATAGCATCCGTTTATT<br>TCTGCAACATGAGAAATAAGGAGATACCTATTAAAACAGAGG<br>CTGGAAATGTTTTCCTATAAATGGTCAGTTGTGATTGTTCTA<br>GGCTTTCTGGGCTACAAAATCTCTGTCACAACTAGTCAGCTC<br>TGCTTTTGTAGCACAAATTCAGTCATAAACAACTTGTCAATA<br>AATGTACATGTCTGTGTTCCAATAATACTTTATTTGCAAAAC<br>ACTGGATTTGGCCCATAACCCACCGTTTGCCAGCCACTGCTC<br>TAAAGTCAAGATAGAGAAGAGTTAACTGTAATTACTGTATAT<br>GTATTTCGAAGTTGGAAATTGTCTCCTCCTGGGACCTTAAGA<br>AACACAAAGGATTTGAGGGGAAAAAAAAGTATATTTTTTAGG<br>GGAAAAATATATTTTTTCATGAAAACATAAGAATTATGATTG<br>CTATCAAATTGTTTTCGTTGCTTGGATAAATCATGATTGAAT

MSTRG.68876.1	0.58	0.1	0.01727	-2.32	down	CCGGAATAGATTCTCCTGCCAGTGTGTTAAATTTGCTCGCAGGTTCACCTTTGTGTGTCCACCAGCATGAACTGCATAAGGGAATGCAAATGAGCCCCTACCTGGGGAAATCTGAGGCCAGAAGGAAGACAACCTGCAGGAGAGGAAGGTGACGGCAATTAAAGAAGGGATCGCCTCACTCCAGAATGTCCGAGGCCCTTTGGAGAAACTGCTGTTTCCCCCAAACCCTCTACATGGCCGCTTGCTAGCAGGTCTCCCCAGCGATCACCTCCATTGGTTTTGTCCTTTTGGCAAAACTGTTTGTTGAATCAATGTCAGCACTGCGAGTGAGGCCAGAAACCCCTCTCACAACCTTGGCCGAAGCAGGAATTCATGGGAGCTGCAAAGGGTTACGAATAAAATGCAGTCACAAAGGGTCAAACGTGGATTCTGCCTTCTCTTCTGAGACCCACCCCTCCTTCACAGAGTTTGCCTGAACCTCGGCCACATTTCAGTCCGGTATGATTATCAGAACCGCTGAGTCTCAAGTTTGCAGCTTGCTCCGTTCATTTCTGTCATCCCATCGGTGAGAAGAAGTGTCTGCAGTCATCCTTCATATTGGGAGGGATGAGCTGAAGGAGGCAGGGAGATGTCTCCTAAGGAGGAGTCTTTCCCAGCTATCATGGCTTCCAAACCTGCTTATCTGGTTCCTCTGGCAACACGTTCTACAGAAGCTGTAATGTATAAATATGGGGACTCCCCCTTGCTCTCTCACAA
MSTRG.13394.1	0.13	0.02	0.03567	-2.37	down	GGAAAACCCAGCAAAGAGAAGGAAACTATTTTAACCAACTTGTCCACTGATAAGAAGAGCTTTATGGAGCACTTCTGAAAGATTTTACAGAAGGACTAGACGTGCAGATTGGTACATTATAAAGATAAATTTGGGGTGGAAGCAAGCACAGGTTGCAGAAGGCTAACAGAGCCAGAGAAACAACTAAGAATAATCCAGAAGAGTGGAGGAGAGCCTGAAGCAGAACACTGGCATCAGGAATGGCGAGGAGGGGGAGAATAAGAAAGAAACCCAAGGACACAATAGGACTTGGAAGCCAATGGGCTCTGAGCGGGCGGCTCTTGGATGCTGGTATGACCCACTAAGACAGGCACTGGTTGGGCAAAAGCAGGAACTCTGGTGAGCGGGTCTGAGTCGGGGGTCAGAGGTGCCTGTGGAACACAGGAAAGACATCTACCAGATACCTACTTTGCGCCCTCCTGACCTCGGGGAGGAAGAAGGAATGAACTCTCCACTTGAGTTTACTGGTTGGCAGATGGCAGTTTGGTGTGAATGCATCTGAGACCAAGTGTTACCTTAGCCAGTAATGTCGGTTTCAAGACTTTTGCTTACTTATCTACTTACTGGAACTTACTTTCTAATTTCCTTATTGGGTGGTTGGGGGAGAAGGCATGGGTAGATGCCAGCAATAAAGTAAACAGGTGGGACAATCCTTCAGGACTCAACCTGCCTCCTGGGAGACCTCCAGCCCATGTAGGCTGCCACCCGTGGCTTCCACAGCGGCCTGCCTTCTCTGTAGCTGACATCAACACTGCTGTTTCCTGCGTCACGTCTGCAGCTTCTACCAGACTAAGTTCCTTGAGAGCTAAGACTGGATCTGTCTTAATTTTGTATTCCCAGACAAAAAAACGAGCACTCGATTCATGTTCACTGAGTGAATAAACGAAAGAATGAACTGAGGTTGGGATACAGGAGTTATAATGTTGTCCTTTTAAGCTGCCGGAGAAGATTGAAAGACTATCCTTTTTCTTTCAAACACCATCAAATGGCCTTTGTTCCCTGGGAGGTGCT

MSTRG.122651.2	0.73	0.14	0.03192	-2.4	down	CACGGCGGGGAAGGAGGGAGGGCACACAGGAAATACAGGGTA<br>AGGGGGCGGGGGAGTCCAGAAGATCAGAATCACCCCAGAGGA<br>TCTTCCACCTTTTTACCCGTCCAGACGTCCCCAGGAGAGCCA<br>GGGACTAGATTCGGGAGATGGGACGGCGGCAGAGAGAAGACA<br>GCAAGCTCCCAGCTGTAGCCAATCCCTGCCCAGGGCTGCGGC<br>TCACCCGCCTCTGGCGGTGGGGACCTTCTAGCTTCTGGCAAC<br>CCCAATCCATCCGACTTACTTGTGTCAGTTACAAACCTGTCC<br>GGTGTTTTCACCCAACATATTAGCGAGTTTGAGGGAAACTCT<br>AAAGGTCTCTCCTTTACTGACTCCTTTAATCCCATTTTGAAA<br>AAGACAACCGAAGAACGCCGGCACCGGCCAGGCAACTCCGCG<br>GCCAGCCCCGCCGTCAGGCCCCGCCCCGCTCCATCGGGGTCT<br>TACTCGCTCTGGCTCCTTGCCCCCGTTTCGGGCTGTGTCAGG<br>AACTTTCTGGAGCTCTCTGGGCTCAGAGGCGGGGACTGGCTC<br>GTAGGAACACTCTTCAACAAACAAACTGCCCCACCCAAGTCT<br>CCCTCCCCCGCCCTTCGCCCCCTGCCCCGCCGCCACCCTAAG<br>ACTTCAAGCTTAGTCACGGCTCTACATTTAGCTTCCAGAAGA<br>TTCTTGCTAGCCTAGCCCTAGTAACGGGGCTCCCCCTCCCTT<br>CCTCTGTTAACAACCGACCAGTCTGTGATAACGGGAAGGGGA<br>GACGGTCCTGGGAGAACCTGGAAGGGCTGAAAAGGTGGAAGT<br>GTGGGTGTTGGCGGGGAAAGCGGCGGAGCTGGGGTGCGTAGA<br>TAGGCGTGAGTCAGAAGCAACAGCCTGGAGAGCGTGGAACGA<br>TAGGGTTTATGTGCCAGCATTGAGAGGAGTCCAAAGTAGAAA<br>GTAGGCCGACATGTTAGTTCAATCACCGGTTCCGTAATTACC<br>TGTCTGGGTGATCTGGCCAAGCCACGAAACCTCTGAACCTTT<br>GTGCTCATCTTTGAAAACAGAAAGGTTTGGCTGAAGGACTCT
MSTRG.70112.1	0.37	0.06	0.01603	-2.44	down	GACAAATCTGGCCTCGGGGAGCTTTCCCCGTGCTCAAGACCA<br>CCAACAACACCATCCAGGGCGGGGGCTGGGAGCTCTGCAGAC<br>ATGAGCACACTGGGCATTGTGTGCTCACGGATGTTTATTCTG<br>AGTTGCTGGAAAACCTTGTCGGGTAGGTCTAGGAGTCAGTCG<br>TGCTGGGCAGGGGTCATGGAAGGCTTCTGGAAGTGGAGTAAG<br>GGTGTCTCAGAGAATCTTTATTTCTTGGAAGATGAGGCAGAG<br>GTCCAGAGAGTGACTTCTCAGGGTCCCTAGGGAGCCAGTGGC<br>AGCTGCAACCCTCTGCTGGTGCTCCCCATACCCGCCACGCGA<br>TGCAGGAGGGCCTCAGGCTGCTGGCTGCCGTTGCGTCCCAGG<br>CTGGGCTGCGTCTCCAGAGCTCAGAGAACGAGCAGGAGAAGT<br>CCTACAAACGGAAGCTGCATTGCTCTCTGACTCTTCAGACGC<br>GTTTGGCGCTTCCCTGGCAGCTTCTGGAATGCATCATTTTGT<br>GAGGCGCGGGGTTGCATCTGAAGGCAGTGGACCTGGTGTTAT<br>ACTAAAACATCACATATGTTTAGCAGTTCCCAAAACCACATG<br>TAGGGTCTGCCTCACCGTAGGCTGAGCTGGTTTCAGGGCGGA<br>GGGGAGGTTTTCACCTCTGCTAATGATCATTAAACAGTAAAC<br>CGGGCTAAGAACCTACCCATTGCTGGGGTGGGCACCAGCGAG<br>TCCCTGGGTGGGACCAGAAGGTCTTCTCTCTCTGTGATTGCT<br>TTCTGTGGTGGCAGAAGGAAGGCTGCACAGCCCAGGTGGCTC<br>TCAGAGCTAAGCTGCTCTTCTGCACGGGGGAGGCGGGCACGA<br>TGGGAGCAGACACAGCGATACAGGACCCACCCCACGTGCACA<br>CCGGGAGACTCCCTTCTTTATGTGGAGAGGGTGTGAATTTCA<br>GTTTCATGATCATCAACAGAAGAGATCGCTGCAAGCAGCCAT<br>GTGTGTTAAATTATTCTGAGACCCCCGTTTCTCCTTTAATGA<br>GCGGGTGCTTTCGTGTCAGGCAGCAATAGTGGCGATTTCCAC

MSTRG.9772.9	0.28	0.05	0.02219	-2.44	down	CCCGGCCGGGTGGCGTCCCCAACCCCGCGCCGGGGGCGGGCCGGGCCGGCCGAGCCGAGCCGCCGGCGTCCATTTTCTGGGCGGTGCTGCGAGACGCCGCGGCCTGACTCGCGGGTCCGGAAGCGCCATGGACCCCCGGGGAACGCGCTCGCGGGGCAGCGGCCGAGCCCTATCTAGATGTGGAGGAAATGGAGGACTCAGAACCAAGGATTTCCAAGTGATTTCTTCCAAAGCTCAAGACGCTAACTCTTTTAAAGCTGGTCTGTTCTAACTGAGACGACCAAAGAATATCCGTTGATAGAAAGTTCTTAAAAACTACATTGCTGTTGATGTAAAGGTTTCTAACAGTAATCAGACTAAAAATTATGGATCGAAGGAACACAACTGCATTTCACAAGAATGAGTGAATTTTTGAAAGAAAGAAAGATAACACTAAGTCATATCCAACTCTTGTGACCCCATGAACTGTAGCCCGCCAGGCTCCTCTGTCCAGGAGATTCTCCACGCAAGGATACTGGAATGGGTTGCCATTTCCTTCTTCAGGGGATCTTCCTGACCCAGGAGTGGAACTCAGGTCTCCTGCATTGTAGATAGATTCTTTACCAGCTGAGCTACATGGGAAGCCTACATGAATTTTTGAGAATCAGTTTAAAAATTCAGCCTTGGAAGTTGGGAAGCCACGGAAGAAGAAGACTACAAAGGCAGAAAGGAGAACTCCATGCCAAAAAAAGAGTCTGAATATTATAAATAAAATCAATTATCACACACTTACTATTTGCTATGTACTTGCCCTGGCTAGGTGCCCTTCTGTGTGTGTGTGTGTGTGTGTGTGTGTGTGTGTGTGTGTGTGAAGTTGCTTCAGTCACATCTGATTCTTTGCAACCCCATGGACTCTAGCCCACCAGGCTTCTCTGTCCATGGGGTTCTCTAGGCAAGAATACTGGAGTGGGTTGCCATTTCCTTCTCCTGGGGATCTTCCCAACTCAGGGATCGAACTCGCATCTCTTACGTCTCCTGCGTTGGCAGGTGGGCTCTTTACCACTAGCGCCA
MSTRG.80295.2	0.21	0.04	0.01717	-2.48	down	AAACCACTCCAGTATTCTTGCCACGAGAACCCCATGCACAGCATAAAAAGGCAAAAAACATATATATTATTGGAGTTATAAACTACATGAAGCAAAATCAGACAAAAATGAAAAATCTCTAATTATAGCTGGAAATTTCAACACTCCTTTCATTTACTTATAGGACAAGTAGACACAAAAATAAAGACTTGACCTGCTATTTATATCAACTTTACATTTATAGAACACTTCATTCAACCACAGCAGGAAATGTTTTCTTTTCAACTGCATGTAAGATATTTATCACCATAGACCATAAAATAAGTCCCTGTAAATTTAAAGGGCTTAAAATATATTATTTAACCACAATAGAATTCAGCTAGAAATCAAAAATAAAAATACTTGGAAAATTTTGCAAAATAATATTTAAATACCATATGTCTAAAGAATACATAAGTCAAGAAGAAATCATGAAGAAAATGTAAAACTTTAAACATGATGATAAAGAAAATACAGCATACTGAAATTTCTGAGTGACAGCTGAATAGAACTACACAGAAGGGAGGGAAATTCTACCTTCAAAATCAAGGTTCTAAGTATTAAAAAGTATCAGTTAAACCCAAAGCAAACAAAATTAAGTAGAGTGGAAATTAAGGGAAGAGAAAATTAGAGATATAGATAAAATCAATTAAAGTTTAATTGGTTCTATTAAAAAAATCAATAAAATTGACAAACCTCTATCAGAGGCAATCAACAGAAAAGAGAGAACACACAAATTCCCAATATCAGAAATTAAAATGTGGACATCAAAATTTCAAACAACCTAACTTTACATCTAAAGGAACTAGAAAAAGAACAAACAAAATGAAAAGGTAGGTGAAGGAAAGTTATCACAAAGATCAGCTCAGAAATAAAATACACACAAAACAATACAAAAGTTGAATGAAACTAAGAGCTGATTCTTTGAAAAGATAAAATGAATAGACCTTTAGCCAGACTCATCAAGAGAAAAAGGGAGCCCAAATCAATAAAATCAGGAATAAAAGTTACTACTGACACCACAAAAAAATA

MSTRG.172143.1	0.45	0.07	0.00871	-2.49	down	GCTAAGGGTTTTGTTTTGGTTCAATGCTTTTTGTCTCTGAATTGAACCATGTCTGATATTCTCTTTGTTTGGATTTGCCTAATGTAACTGTCCTAAAGTAATATAATTTCTGTTTTCAACCTTTCTGAATCACTTTGAGAAGTGAGTGTTGCCCATGCTTTGGAACCGTGGTGCTGGAGAAGACTCTTGAGAGTCCCTTGGACTGCAAGGAGATAAACTAGCCAATCCTCAAGGAAATCAACCCTGAGTATTCATTGGAAGGACTGGTGCTGAAGCTGAAGCTCCAATACTTTGGCCACCTGATGTGAAGAACTGGTGCATTGGAAAAGACCCTGATGCTGGGAAAGACTGAGGGCAAGAGGAGAAGGAGGCAATAGAGAATGAGATGGTTGGATGGCATCACCAACTCAGTGGAGAAGAGTTTGAGCAAATTCAGGGAAATAGTGATGGACAGGGAAACCTGGTGTGCTGCAGTTCGTGGAGCTGCAGAGTTGGACACGACTTAGCAACTGAATGACAGCAACAGCATACGTCATGGACTGCAAG
MSTRG.221654.5	2	0.37	0.01829	-2.5	down	ACAGAATTTAAAATTCAGCCCTCAGTTGCACTGGCCATAATTCAAGTACTCAGTAGCAACAAGTACTCAGTAGCAACATATGGCTAGTGGGTACCATATTGAATGGAAGAGGCATGGAGCCTTGCCATCATGGCAAAAAGTTCCCCTGGACACAGGACTGCTCTGAAAGATCTTTTCTTTGTGATTGCAGTGCAGCTGTCTGCTTCAGGAAGTGAGGTCAACAGGAGCAAGGAGAATCTTTAAGAGCTGCCTCGCATTGTGACATGTCTGATTCCTTGCTGCAGTCCCTACAGCATGAACAAGGTAAGAGGCACTTTGGGGACTGATCCTTTGTGTCTTCTTTATGCTACCATGGATTTCCCTGGGTACGTCATGTCGTTCTGTGATGTGAGGAGGTTGTGTCTCCTGCCAGAAGTATTATGGAGGTTGGCAAGCAGCGTTGACAGGCAGGGTCACAGGCTGCTGCTGGTGTACTGTTCTAGAGGTGTCAACTGTGTTTCTTGGAGGCCTCTGAGCAGCTGGACTGTCAGTTGTTAGTATAATTGTCTTGGCATGGATATTGGAAGACTGAGGCTCTAGGGCCAAGCTGTCTGGGTTTAAATATCAGCTAGCTTTGAGGACCTTGGGAAAGTGGCTTAATGGCTCTGTGCCTCAATTTCCCCATCTGTAAAATGCTGGTGATAATGGTATGTAACTCGCAGGGTTGTTTTAAGGATGAAATGAGTTGATATGTGTCCCAGTGTTTAATACAGCCCCTGTCTACACTATAAGCATTTGATTAGTTTTTTGCTGTTGTTGTTTTTGGTGTTGAGGACACTGTTGTCCCTCAGATATTGTTGCCAGCAAGCCTTAGGTGCAGGTGGCGCCAGTGGTAAAGAACCCACCTGCCAGTGCAGGAGACTTAAGAGACATGAGTTCGATCCCTGGGTTCGGAAGACCCCCGGGAGGAGGGCATGGTATCCCACTCCAGTATTCTTGCCTGGAGAATCCTATGGACAGAGGAGCCTGGCAGGCTACAGTCCATGGGGTCGCACAGAGTCAGACATGACT

MSTRG.178915.1	0.25	0.04	0.03206	-2.53	down	GTCTTATCTTTTTAAAGAGGGGAAAGAATTATTTAGGATTCA<br>AGTACAGTCAGTAATCCATGTAAGGGGAAGCCTCTATTATAA<br>ACCATTGTTGGAGCTAAGTATGTTTGACTTCAACAGTTGTTG<br>AGAACATGAATCTTTGTATAGAGCATCAGCTTCTGTTTCTGA<br>ATATGTGCTGAGATTACTATGTTGACCTTATTACAGGGTTTC<br>TTGTAGCTATAGCAAGTGACAAGAGCTAGCAACCCCTGCAGT<br>ATAAATCTCTGTGTTTTGGGGACCCAGTAAAGTGACTGAACT<br>CAAATAGTGAATAGCAAAGCAAAGATTCTCCCTTCCTCCAGG<br>GAGAGACCTGGTCTTTCTGAAGAGGAGATGACTACAAACTCT<br>TCTTACCTTTTCAGTTTGCTTAAGATAAAAGAAAGAATTTAA<br>GATTCTGTAAACATTGTCTGATTTTGCTGCTTTTTTGATGGA<br>GGAAACACTTGAAGTTGTTAACAGTGTTGTGGTTTGTGGTAG<br>CTGTAGGGGTTCTCCCTTGGGAAAGAAGAGTGGTCTTCTCTA<br>GTATTGAATAGAGCTTTATATTTTCCTCCGTTCTGAGGAGAG<br>TCTGGTGAGTTAACTCTGTGAAGTGCTCAAGCTGAAAATGTG<br>AGCAAATTTCTGGAGGGAACAAGGGAGGGAAAATAAAATGCC<br>AAGGGGACTTTATACTGCATTGCTTTAGAACTGAATCCTGGT<br>GAATCTCAAAATAATTATGCTGAGTAAAAGAATCCATATAGT<br>GTATGATGTAAAATTCTAGAAAATGGAGACAGAACTGTAGTG<br>ACAGTGGTTGGCTGGGAATATGGAGAGGGGAGGATGGGCTTA<br>TAAAGGAGCAGGAGGAAGCTTTTATAATATGCCCACTGTTTT<br>GATTGGGATGATGTTTCACAAATGTATGCATGTTTCATCATT<br>CATCAAATTGTACATTTCTTTTGGCTGTACCACGTGGCTTGC<br>AGGATCTTAATTCCCCACCAGGGATTGAACCTGGGCCCTCAG<br>CAGTGAAAATGTGGAGTCCTAATCACTGGACTGCCAGAGAAT
MSTRG.40310.2	0.61	0.08	0.01644	-2.59	down	GTGCCGGCTCGGGCGGCCGCCGCGGGGAGGCCGCGCGCACCT<br>GTCCCTGCCCGTCTGCGCCGCCCGCCGCCGCTCGGCCGCGCG<br>CACCGCCGCAAACCCGCGCCCGAGCGCAGAGCGCGCGCCGCG<br>AACATGACTTCTGCCTTCAAGCTGGATTTCCTCCCGGACATG<br>ATGGTCGAGGGCCGCCTGCTAGTTCCCGACAGAATGTGAGTG<br>TGGCCGCCCGGCCGCCTCCGGGACGCTGGTCCGGAGCTCCGC<br>GGAGGGAGAGGGCGGGCGGCGGCGCTCCTGCGCCCTGGGGAC<br>GCGGGGGGCGGCTCCGGTTCGGCCGCGCCCTGGGGTTCGGAG<br>CCGGCGCTGGATGCGGGGAGAGTAACGGCACAGCCAACAAGA<br>TGAACGGCGCTTTGGATCAC

MSTRG.222581.1	0.49	0.08	0.01237	-2.64	down	CCCGTGTGCACAGCCCAGAGGCGAGTTTTTACACCATTTCTC<br>GCCAACAAATGGTATCTTTGCTCTCTGAGTGTGATCACAGGA<br>CTGGGAGCCGGGGTGACGACCGGAATTAAAATGAGGAGAGGA<br>TAATCCAATTTATTGAGCATTTATTATGTGCCAGGTACTGTG<br>AGAGAATTCAAACAAGCTCCTCTCCCTTCTGAGAAATGAAAG<br>AGTAACTCCTAACCCTAAGGCAGAGAAGAAAATGAGACGTTT<br>GACGAGAAGTATGTAACAACCACTGCCACCATACATAATTGG<br>ATGTTCTTTGTAGCAGGAAATAAGACTTGAAAATCTTAAAAT<br>GGAAACATCTTGAGCTTCTTGTGGTCTGATTCATTCCTCATA<br>TAGACGAGTACAATGAGAGCCAGAGAGATGGAGTAAACTCCT<br>TGAGATCATGAAACTGGAGTCAAGACCTCACAGCTATGTTTG<br>ATTGCTGGGAGAAATGTCAATAACCTCAGATACGCAGATGAT<br>ACCAGCCTTATGGCAGAAAGTGAAGAGCCTCTTGATGAAGAT<br>GAAAGAGGAGAGTGACAAAGCTGGCTTAAAACTCAACATTCA<br>AAAAAATAAGATCATGGCATGCGGTCCCATTACTTCATGGCA<br>AAAAGATGGAGAAACAATGGAAATGGTAACAGACTTTATTTT<br>CTTGGGCTCCAAAATCACTGCAGATGATGACTTCAGCCATGA<br>AATTAAAAGATGCTTACTCTTTGGAAGAAAATCTATGACCAA<br>CCTAGACAGCATATTAAAAAGCAGAGACATTACTTTATGATA<br>AATGTCCATATAGTTAAAGCTATGGTTTTTCCAGTAGTCATT<br>TATGAATGTGAGAGTTGGACCATAAAGAAAGCTGAGCACCAA<br>AGAACGGATGCTTTTGAACTGTGGTGTTGGAGAAGACTCTTG<br>AGAGTCACTTGGACTACAAGAAGATCAAACCAATAAATCCTA<br>AATGAAATGAATCCTGAATATTCATTGGAAGGACTGATGCTG<br>AAGCTGAAACTCCAGTACTTTGGCCACCTGGTGTGAAGAGTC
MSTRG.1284.17	0.19	0.03	0.0345	-2.65	down	CTTAAAGCCTGAGCTCAGAGAGTTACCCGAAAAGTGATTTGA<br>GTATTTATTGAACTGAAAGAACAAATTACCTACTTCAGGCGG<br>TGGGAAGCGTATGGAAATTATATTAGAACTTCAGCAAAGTGC<br>ACAGCCGCATAATACAGAAGCCGAGACTCAGATTGACAGGCA<br>AATAAGCCAGCTCGTGGAAGGGGCCTCTGGGTGGCAGCTCTG<br>GGCTATTGGGTGTTGGACAACCATAGGCAAACTGAAATGGAA<br>CAGGGGCAGAAAATAGCACACTTTAACGCTGCGCAACCGTTG<br>CTTTCTCATGGTCATTAGGAATTTTGTTGGGATAATCCTCCA<br>AATGAATTAGAAGCATTCTATTCAAATGGTTATTTATCATTA<br>AAATGGCATTGTAAAGTGATGACTCAGAAGTTAACAGAGTTT<br>GTGGCCGCTTTTGAGAAAGAAAACTCTTGATGTCAAGTTCTG<br>AAAAGTGAAGAGAATGAAATACAGAATATGGAACACTATCAT<br>CTATCATTCTGGAATTCCTTCTCCCTACAGTCTATGTGGTGT<br>TTCTAGCATGCAGCTCCTTCTCTCCAGGCTTTTGCTTATGCT<br>GACCTCTTTGTCTGGAGGCTTACTCTTCACCCCCAACTTTTC<br>GTCAATTCCAATTCCAGTTCATTCTTACCTGTCCTTGTAGAG<br>CCAACCTAGTCCACACCCTCAACCTGACTCCAATGGGAGATG<br>ATGACTTTAATACATGTCCTGTAATATCCTGTGCATTTTTCT<br>ATTAAGCCTGCCAGACTCTTCTGTCCATGGGATTTCCCAGGC<br>AAGGATACCGGAGTGGGTTGGCTTTCCCTTCTTCAGGAGATC<br>CTCCTGACCCACGGATTGTACTTGCATCTCCTGCATCGACAG<br>GATATCCTTGAAGATTAGTCAGAGAGCCTCTTATGTGCCTCT<br>AAAGGGAAAAACTTAAAGTGGATATGAGAAGTGAAGAAAGTG<br>ATCATAGGAGAAAATCATCTCAGGTAAAACTAAAAATCAAAA<br>GAGCAATTAATATTTAATATTTTTAAAGAAAAAATAATTTTG

MSTRG.157095.1	0.63	0.1	0.00675	-2.7	down	CTTCCGGGTGTGTCAGCCGCCCTTCCCCCACCGTCGCCGGCG<br>GGAACCGCCCGTCTTCTCCGCCAGCAGCTTCCGCCTGCCGCA<br>GTACCGGCACGCGCTCTGGGGTCCCGCCCCTTCCCAGGGATA<br>GTGACCGGGCGAGCGCGCGCCTCGCGGTACCGAAGACATCTG<br>AGCACGGGCCAGGGCTTCGGCACCAGAAGCGGGTTGTGATGG<br>TGCTGGAGAAGACCCTTGAGAGTCCCTTGGACAGCAAGATCA<br>AACCAGTCAATCCTAAAGGAAATCAACCGTGAATATTAACTG<br>GAAGGACTATGCTGAAGCTCCAATATTTTGGCCACCTGATGT<br>GAAGAGCTTACTCATTGGAAAAGACCTTGATGCTAGGAAAGA<br>TTAAGGGCAGGAGGGAAAGGGGGTAACAGAAGATGAGATAGT<br>TGGATGGCATCACCAACTCAATGGACATGAGTTTGAGAAACT<br>CCAGGAGATGGTGAAGGACAGGGAAGCCTGGCATACTGCAGT<br>TCATAGAATTGCAAGTAGTCAGATACAGCTTAGCGACTGAAA<br>AACAATGGTGACCTCCAAGAGGGCTCACACTAAGGGGCACTT<br>CCTGGGACTGCTGCTGCTAGTGCCCCCATCCCTGCAGCGAGC<br>CACTGCTGACCCACGACTCCGCAGGAGTTCATCGAACACTAG<br>ATGGTTGGTCTGGTTCAGTCTCCTGTGGGGTCACTGCTCCTT<br>TCCTCTCAGTCCTGGCGCACACAAGATTTTGTTTGTGCCCTC<br>CAAGTGTGAAGTCTCTATTTCTCTCAGTCCTATGGAAGTCCT<br>GCAATCAAATCCTGTTGGCCTTCACAGTCAGAATCCCTGGGG<br>ATTCCTAGTCCCTTTGCTGGATCCCCAGGCTAGGACACTCAG<br>AACCATCACAAGAGTGGGAGAACTTCTTTGATATTAGTGTTC<br>TCCAGTTTGTGGGTTGCCTGCTAGGCGGGTATGGGATTTAAC<br>TTTATCGTGATTGTGCCCTCCCCCCACCATCTCGTTGTAGCT<br>TCTCCTTTGTCTTTAGATGTGGGATATCTTTTTTTGGTGGGT
MSTRG.90432.3	0.13	0.02	0.02659	-2.72	down	CTGCAGCTCAGTCTTTTTGAGTCAAACCAATTCCTACCCTCT<br>CTGCAGAAGTTGATGATGCACAAGATTATCACAGGCAGTCTG<br>TCTTCCAGCGACAACCAGCCATGGGCAGCCTTCTACCTGGTG<br>GGACAGATTTCAAGGCCTGCTGTTCTGCTAAATGCTGAAACA<br>GCGTCTGGGGTGCCCACCCGTTCAGCGCACTCTGTGACATGG<br>TGACAGCGTCTCCTCGTCTGCAGAACTTTCCTCAAGAACGGA<br>CCAAGGACAAGGGCGGACAGAGCTTGAAAGACGTGAACGCTG<br>TACCCCTCCAGGAGGAATCTCCCATAATTCTCCAGTTCTAGG<br>GAACAACAGAGTCACCTACTGACACAGTGGGTGTTTTGGTCA<br>ATTTTTGTCCCTCCAGCGAAAAATTCATGTGTCAGAAGTCCA<br>GTGGTTAAGATTCCCCACTTCTAATGCAGGAGGCATGGGTCC<br>GATCCCTGATTGGGGTGTGCAGGTGAGTGGGTGGGTTGGTTG<br>GTGGGTAGATGGGTCAGATGGGGGGTGGGGGATTGGATTAGT<br>GGGTAAATAGGGTGGGTGGTGGGTAAATACATGGATGGATGG<br>CTAGAGACAGAGAGATACAGATAGATACCCAAAACTCAGGTT<br>ACAAAGGAAGGGAAAGGGGATTAAAAAAGGAAAGACCCCCTA<br>AGTCAAGCTTGACAGTCATCACAGAGAACAAAATAAAGGCAG<br>AAGGAGTGTCCCCTCTACCAAGATCAGTGAAAACCCTGCTTT<br>ATTTCTAGGTCCCAACAGAATTGCTATAACCCCACGAAGCCC<br>AAATCAGTTCTAAGTAGGAGGGGGAAGTGGGAGGCTGTGGGG<br>ATTGGGTCATGTGCCTTTTTCCTGGAAGAAGGGAGTGTACCC<br>TGGAGCCTAGCACCCTCCGGAGAGGTCCTGCTGACCAGCCGA<br>GGGCAGCCGCCTTGCTGGGGGACGTCGTCTGGGAACCACCAC<br>TGGCCGGTTGCCGTGGGTTCAGGAGTCCGAGTGCACAGAGGT<br>TGCCTTGGGGATGCTGGAAGAGGCAGCATTTCTGGCCAGCCC

MSTRG.92495.1	0.3	0.04	0.01559	-2.8	down	CAGCTATATGAGGCAAGAGGACAATTCTAGTGTACAGTATTTCATTGTGGCCAGAAGCAGAAGTCCCAGGTATCTTTTTCACAAAGGTCCCAGAAGCTACAGCCTGGGTTTTTATCAAACAAACCAGTCAAAAGTGGGCCAAACACCTAAATGGACATGGATCCCAAAGAAGACATATAGAACACCAAAAGCACATGAAAAGATATTCAACATCACTAATTACTAGAGAAATGCAAATCAAAACTACAATAATGGTTGCCTCACGCCAGTGAGAATGGCCATCATCAAAAATTGTACAAGCAGTAAATGCTAGAGAGGGTGTGGAGAAAAGTGAACGCTCTTATGCTGTTGATGGTAATGTAAATTGGTACAATCATAGTAGAGAATATCATGGAGTTTCCTTACAAAACTAAAAATAGAACTACCCTATGATCCAGCAGTCCTACTCTTGGGCATATATTCAGAGAAAACTATGATTCAAAAATATACATGTACCCCAATGTTTATTGCAGCATTATTTACAATAGCCAAGACATGGATGCAACCTAAACGTCCATTGACAGAGGAATGGGTAAGGAAGATGTGGCACATACATACAATGGGATATTACCCGGCCATAAAAGGAATGACATAATGCCATCTTCAGCAACATGCCTGGACCTAGAGATACCATATTAAGTGAAGTAAGTCATATGGAGAAAGACAAATATCATGTGATTGGTGGAATCTAAAGAGAACGATATGAATGAACTTATTTACAAAACAGAAAAAGACTGACAGGCTTAGGAAACAATTTATGTTTACCAAAGGAGGAAAGTGAGGAAGAGGGATAATTTAGGAATTTGGGTTAACACAGACACACTACTATATATAAAATAGATAATTACCAAGGACCTACTGTATAGAATAGTGAACTCCATTCAGTACTCTGTATTGACCTACAAGGGAAAAGACTGAAAAAGAATGGGTGTGTGTGTATATATATATAACTGAAATTACTTTGCTGTACACGTGAAACTAACACAGCACCGTGAATCAACTATGCACGTG
MSTRG.163926.5	0.14	0.02	0.01598	-2.81	down	AGAGGATCAAAAATGCTATAAAGTTATCCTCTGAAAATATCAATAAAATGTTTATCTCACTAATCAATGAAATGAATCAAGAAAAATCCTACCAACAACTAATAACAGCAAAATAATAGTGATGTCTCTTCAGATCATAAAATCAGAGGGTGTTTTGACAATAAATTTGAAATTTTAGATGAAAATAGGCAAACTCCTATAAAAATATAACTTATTAAAACTGATGTAAGAAAAAGTTTTAGTCTGAATAAATCTATAATATTAGAAAAAAAATTGTTTTAATGCCTCTGAGCTGCTGATGCTTTGTGGAGGGTGTTTGCATCCCAGGGGACAGACCGCCCAGGGCGTGATCACCGGTAATTGTTACAGAAAATCCTGTGCTCCACGGAATGCTAATTCTCTGCACTCCTGGCTTAAATCCTGCCGTTGACAATAATTCTTTTTGAATTGCTATTCAGGGAAAGCAATTTAAGCCAGAAAACAAGAGTAACTTTTCTTCCAACTCCCTTGCCAAGCAGGCTGTCCATTCTCGTGGTTAATTTTCCTTTTACAGTGTGTTTTAAACAACTGTCATTTAAAGTGAAAAAAAGTAAAAAATTGGAAGGAAGGAAGAACTGGGGGAAGAAAAGCAGTGCTGGGGGTGGTGAGTGTGACAAGTGTGAAGTGAGGGTGGTGGGTGTGGCAGGTGTGGAGTGCTGCTTCTGACCAGAGCCTTTCCATACCCATCTCTGCAGGATAAGGTGGAGCAGACACCTCCTCAGCGTCCTGGGGAGCTGGCAGGACCTGGCTTCCCAGCGCCGCCCGACCGGCTGGCGAGCCACCAAGGTAAGGATGAGCCGAGCGCTCCCCTGACCCAGGCTGTGATTCCCGTTATATTGTGCAGCGGTGCTCAGCATCGCAGTCTTAGGAATCCATTCCTGGCACTGACGTAAGAAGTTGCTGGTCTCAGCTCCACTTTACCCCTTTAAGGAAAACACATTTTGTAAAAGAGAGGTACTTGGTTATATGTTCATAGAGTTTTCTTTCAATGAGCCGACTCCAGTGATTGAAA

MSTRG.84611.2	1.7	0.21	0.04659	-2.84	down	TGAGTACACGGAGAGTTGGTGGGAAGAGGATCTTAAAAGCCA<br>AAGTCATACAGCTGCACAGAAGGGGGAGAGGCAGAAAAGTGA<br>GTTACAGAGGAGCTTCTAATTTCCTCAAAGGCTGACATCCTA<br>ACACAATAATCCATGCTGATCAACCAAACATTTCCTAAAGTA<br>TGAAGGATTTTTTTTTGGCTGCTGTCTGATCCCTGAACAGA<br>AATCGTACTGGGGCCTCCTGCAGTGGAAGCTCGGAGTCTTAA<br>CCATAGGACCACCAGGGAAGACCAGGTCATTACAGAGTACTG<br>AGAAGAGTTTCCTGTGCTATACAATTCCACATGTAACTGCTG<br>GATTTCTTACACTGTTTACAATACAGCCAGGTTGCAGTTTTC<br>TTGATGGTGGATTCTTTACCACTGTGCCAAGTGGGAAGCCCT<br>GGCTGCATTAGGGGAAAAGTGAAAGTGAAAGTGAAGTCGCTC<br>AGTCATGTCCAACTCTTTGCAACCCCATGGATTGTAGCCCAC<br>CAGGCTCCTCCATCCATGGGCTTCTCCAGGCAAGAGTACTGG<br>AGTGGGTTGCCATTTCCTTCTCCAGGGGATCTTCCCGACCCA<br>GGGATCGAACCCAGGTCTCCTGCATTGTAGGTACACACTTTA<br>CCATCTGAGCCACCAGGGAAGTCTTTAGCGGGGAGTTTCTAA<br>AAAGACAACATGACAAGAAGACATCTTTGGGACCAGTTCTTA<br>GAATTTGGAAACATGGTTGATATTCGTGGTCTAATGCAAAAC<br>CTGTTTCACATAGCTCTAAGAGGTATAAAGAAGCTCTCCAAG<br>GCATGGAGGATCAAGGATTCATCAACTCCTGTTGCTGTCTAC<br>ATCAATCTCAGATTCAACAAGAATTCACTGGCTTTTGATCAA<br>GGCCCCATTACTCATGCTCTGAGTTTTGAGAGCTTTTGGGTC<br>CTCCTAAAAACCCAGTGGGGGGCTTCTATGCTTGGCACTCCT<br>TAAGCTCTTCAACTGTTACAGTAAAGGACTCAATAACCTCAA<br>GGCTACTCAACTGTTAACTGAGTGAAAGTGCCAACTCCTTAG
MSTRG.90432.6	0.14	0.02	0.03721	-2.86	down	TTTTTGAGTCAAACCAATTCCTACCCTCTCTGCAGAAGTTGA<br>TGATGCACAAGATTATCACAGGCAGTCTGTCTTCCAGCGACA<br>ACCAGCCATGGGCAGCCTTCTACCTGGTGGGACAGATTTCAA<br>GGCCTGCTGTTCTGCTAAATGCTGAAACAGCGTCTGGGGTGC<br>CCACCCGTTCAGCGCACTCTGTGACATGGTGACAGCGTCTCC<br>TCGTCTGCAGAACTTTCCTCAAGAACGGACCAAGGACAAGGG<br>CGGACAGAGCTTGAAAGACGTGAACGCTGTACCCCTCCAGGA<br>GGAATCTCCCATAATTCTCCAGTTCTAGGGAACAACAGAGTC<br>ACCTACTGACACAGTGGGTGTTTTGGTCAATTTTTGTCCCTC<br>CAGCGAAAAATTCATGTGTCAGAAGTCCAGTGGTTAAGATTC<br>CCCACTTCTAATGCAGGAGGCATGGGTCCGATCCCTGATTGG<br>GGTGTGCAGGTCCCAACAGAATTGCTATAACCCCACGAAGCC<br>CAAATCAGTTCTAAGTAGGAGGGGGAAGTGGGAGGCTGTGGG<br>GATTGGGTCATGTGCCTTTTTCCTGGAAGAAGGGAGTGTACC<br>CTGGAGCCTAGCACCCTCCGGAGAGGTCCTGCTGACCAGCCG<br>AGGGCAGCCGCCTTGCTGGGGGACGTCGTCTGGGAACCACCA<br>CTGGCCGGTTGCCGTGGGTTCAGGAGTCCGAGTGCACAGAGG<br>TTGCCTTGGGGATGCTGGAAGAGGCAGCATTTCTGGCCAGCC<br>CAGGTGAGCAGAGAATACTAGCGGGAATAAGAGCAGACTCGC<br>CAGGCCACGGGGAGAGGGCTGCAAAATTACTCACACAGCTGC<br>TCTATTTGTCTGTAAGAGGAGCAGTCGGTCTCCACCAGCTCC<br>CACCCGCGGGATGGCTGGGATAAAGGGAGTGGGGTGGGAAAG<br>TCCTTCACTGCAGCCTCATCAGATCCAGTAACTCATGCTTGA<br>GTAACTCAGAGCTTACTGGGATGACGTGGGTAAGTGCAGACG<br>TGGAGGTTCATGTTATGATGAGCCTGGGAGTGAGTGGAAGAG

MSTRG.156627.7	0.91	0.13	0.02554	-2.9	down	GTCATCACATGGTGGAGGCAGAGAGCTCTGGGGTCTCTTCCTCTTCTTATAAGAGCACCAGCCCTATCAGATTAGAGCTCTACCCTTATGCCCTCATTTAACCTTTATCACTCCTCACAGGGTCTGTCTCTAAATACAGTCACACTGGGTGCTGAGCCTTCAACATATGAATTTTTGGGAGAACACCAACATCCAATTCATAACACCATTAAAAAGGAGGAGAAAATATTTTTATTGGGTCATTTTCCCTGGGGCTGAATCTATTAGATCTACACAGGAGAGCATTGAGAGAGCTAATTGGAAAGATTGCTAAGCTTGCTGCAAGAAGCCAGAGAGGGGCATTTTATTTCTCACTTGGATGCTTGCTGAGCCGCAGAACACAGTACCCTGACTGCCTCTGGACTTGAGTGCATGAAGCAAGCATCCGTGTCTGGGAGACCCAGTGAAAATGAACTTTAGGGCAACTACTGGCTGTCAGCAGGCAAGAAACGGAGGCCCCCAGTCCAGCAGGACAGAGATCGCTGACTCCTGCTAGCAAGCATATGAAGCTGGGAGCAGATCCCTCTCCAGTTGAGCTTTCAGATGAGATGCTGGAAACCTCGATTGAAGCTTCATGAGAGACCCCGAAGCAGAGGACCTGGTGAGCTATACCCAGGTTGTGCGAGATGGTACATGTGTGTTGTTGTTAGCCACTAAATGTGTGCTAATTTGTTATACACAATAGGTAACGAATATGCAGTTTTGTGGTTTTGCTTACATCACTCCCCTCTTCACAGGACTGCAGTATAAGTCCCTGGAGATTGTTCTCAGAAATAAATATAACTGACAATCACCACTCTGTGCTCAGAATTGTCCTTCTCCTTCCACCAAAGGTCTGTTACCTGATCAGCTTTGTTGTGCTTGATTTCTCTGGGAAAAGTTCTCTCTCAGGTGACCTTAGCTCCTCACACAAAGGGAGCTTTGTGGGGCCTGCCTATGTGGTTCCCCAGATAAGCCTGAATACTCTTAAATCCAGCAATGGTGCAGCTTGGCCTAAAGCTAAGAGCTCAC
MSTRG.168210.5	0.39	0.04	0.01912	-2.9	down	TGGCACTATAAAACGGCTCTACAAAACCAAAACACTTCTTGTAATTTTATTGCAGATCACTATCTTGTCCTCCACCTTCATCTTTTTGTGACCTCACTTTCCCTCTTGTACTTTGAGGAACTAGCAGCTAGCAGATTGCTGTTCTACTTCTGATACCTTCAGGGGTCCACTATTCATCTTGCCTCCCATCACCTCCTGTGCATAATTCATGGAAACTATTTGTTCTGCTGTGTTCCGGCCACACTACACTGGAGAAACACTCACTGTCCTTTTATTACCATGCTGTGAAGAAGCCTAAAGTAGCCTACTGTAGATGAAAGTCTATCCATGATGGGAGAGTAGAGGTTACCCACTGACAACCAGAACCATGTTCCAGAGGCCATCTGGGACCTACAGATTCAGAGTACATCATGCAGAATACCAGGCTGGATAAATCACAAGCTGGAATCAAGTTTGCCGGGAGATATATCAATAACCTCGGATATTCAGATGACACCACCTTTATGGCAGAAAGTGAAGAGGAACTAAAGAGCCTCTTGATGAAAGTGAAAGAGGAGAGTGAAAAAGTTGGCTTAAAACTCAACATTCAGAAACTAAGTTCATGGCATCCGGTCCCATCACTTCATTGCAAATAGACGGGGAAATAATGGAAACTGTGAGAGACTTTGTTTTGGGGGGCTCCAAAGTCACTGCAGATGGTGATTGCAGTTATGAAATTAAAGGATGCTTGCTCCTTGGAAGAAAAGCAATAACCAACCTAGACAGCATATTAAAAAGCAGAGACATTACTTTGCCAACAAAAGTCCATCTAGTTAAAGTTATGGTTTTTCCAGTAGTCATGTATGGATGTGAGAGTTGAACCATGAAGAAATCTGAGCGCCGAAGAATTGGTGCTTTTGAACTGCGGTGTTGGAGAGGACTCCTGAGAGTCCCTTGGACTGCAAGGAGATTCAACCAGTCCATCCTAAAGGAAATAAGTCCTGAATATTCATTGGGAGGACTGATGCCAAAGCTGAAGCTCCAAATTTTTGGCCACCCGATGAAAACAAC

MSTRG.215732.3	0.21	0.02	0.00678	-2.9	down	CAGGAAGAGAAGGGGACGACAGAGGATGAGATGGCTGGATGG<br>CATCACCGACTCAACGGACGTGAGTTTGAGTGAACTCCAGGA<br>GTTGGTGAAGGACAAGGAAGACTGGCGTGCTGCAGTCCATGG<br>GGTTGCAGAGATTCAGACATGACTGAGTGACTGAACCGAACT<br>GAATTAGTGGATATTTCCTCGGTAGAATATTATGCATCCAGT<br>AAAAGCCACAAAGACTACTTCAAGGCATGGCAGAATGTGTCT<br>GCATAAATCCTGATTTTTTTGAGGGTCCACGTGACTTGAGAG<br>CTTCAGGGTAATGGAGCAACAGCTGTCAGACAATTTTGCATG<br>TTTTATACAGCTCATAGCATGTGGCACGGGAGACTGAGAAAG<br>GGGTCAAGGGGGTTCTTTGAGAAGGGAAAGCTTTTTTTTTTT<br>TTTGGCCTGGATTTTCACTGGTTTGTATTAAAGACTGAGGGC<br>AGGAGGAGAAGAGCGACGGAAGATGAGATGGTTAGATGGCAC<br>CAATGACTCAATGGACGTGAGTTTGAGCAACTCCAGGAGGTA<br>ATGAAGGGCAGGGAAGCCTGGCTGCTGCAGTCTGTGGGGCTG<br>CAGAGAGTTAGATGTAAGTTAGCAACTGAACAAGAGTTCATT<br>AGAAGTGCTTGTAGCCTGAGAGTTACATGAAGTCTACAGAGA<br>TTCAAAAGTACCTAGGTTTGAATCCAGCTCCACCTCTTATTT<br>AGGGGCTTCCCACGTGACTCAGTGGTAAAAGACTCCTCCTGC<br>CAATGCAGAAAATGAAGGAGAAGCATGTTCGATCCCTGGGTC<br>CGGGAGGTCCCCTGGAGGTGGAAATGGCAATCACTCCAATTT<br>TCTTGCCTGGAAAATCCCATGGACAGAGGAGCCTGGCTAGCT<br>GTGGTCCAGCGGGTGGCAAAGAGTCAGACACAACTGGGCACA<br>CACACACACACACACACACACACACACATCACCTTTTATTAG<br>CCATGTGATTTCTGACTCTCAGTTACTACAGCTGTGAAATGG<br>GAATGGTACCCTCTGTCAGAGGGCGATTGAGAGAATGCCGTT
MSTRG.98647.1	0.12	0.02	0.01163	-2.93	down	TGGTCATGTATGGATGTGAGAGCTGGACTGTGAAGAAAGCTG<br>AGCACCGAAGAATTGATGCTTTTGAACTGAGGTTTTGGAGAA<br>GACTCTTGAGAGTCCCTTGGACTGCATGGAGATCCAACCAGT<br>CCATTCTGAAGGAGATCAGCCCTGGGATTTCTTTGGAGGGAA<br>TGATGCTGAAGCTGAAACTCCAGTACTTTGGCCACCCTTGGC<br>AACTTTTCTATTAGAACTGTAAACACATATTTGTTCCAAGTT<br>CATTCCTTTCCCTTGCTACAATCTGTATCACAGACAATTGAT<br>CTCTTCAAGGCTGTACCCTTTGCTCTGACATGGTTTGGAATT<br>GTGTCACATCAGTAATCAGACTCCAATCATCCCAGCTCTTCG<br>ATGCTCTTGCTATTAGTGCATGTGCTCTTTGATCAAAACATT<br>AATCAGTTCTCTCAATTCATCAGCCAACTCCTGACCTTCCTT<br>CCTTCTTCTCTAGAGTCAGGATTCTGTGATGCATTATCTAGT<br>CCCGTGGTACTCAAATGCTCTGTGGCCAGCAGCATGGGCATT<br>GTCTGGGAGCTTGTTAGACATGCAGACTCTGAGGGCTCACCA<br>GACTGGCATTTTAGAATCATCTTAACAAGATTCCTAATGATT<br>CGTATGCAGATAAGGTGGGATTCCCTGGTGATTTAGACGGTA<br>AAGAATCTGCCTGCAATGTAGGAGACCCAGGTTGGATCCCTG<br>GATCTGGAAGGTCCCCTGGAGAAGAAAATGGCTACCTACTCC<br>AGTATGCTTCCCTGGAGAATCCCATAGAGGAGCCTGGCAGGC<br>TGCAGTCCATGGGGTCACAAAGATTTAGTGACTAAAAATAAC<br>AACTCTCTGAATTCAGTATTCCTAGTACTGAACTCAGTTATC<br>TACAAATACAGATAGAAACCATAGGTCATAGGAGAAACTTGG<br>ACACGCTTGAAATTTGTGGAATAAGGGAAAGACTAGTTGGAA<br>GGTGAACAGGAATATTTAAAAATATTTCCTTGTAATCAGTTG<br>TCTCTTCCCCCATCTTCTTCACCAGACTGAGTTGTCAGGGTG

MSTRG.34779.2	0.16	0.02	0.01222	-2.94	down	CAGAAATCAGCCCCCCACTAGCGCCGCCCGGGGCTTCCGCCC<br>CGAGGCCTGACGGTCGCCGTTCGGTTCCGGTTCTAACCCCGC<br>CCTCACGCGGTAACCCTCCAGAAGTCTTAATATCCGGGACCA<br>GAGGCTGGCGCCAGCGCGCACTTCCGGCGACGGACGCAGAGG<br>AAAGAATCTGGTGACGCTTCTTCCCTTCTACCGTGCCGCCCA<br>GGCTCCGGCTCTCGGTGCGGCAGCGCCATCTGGTGCTGAGTC<br>TCTCGCTGCCCGCAGAGGGGGCGCGCCCTTCTCCCGGAAT<br>CTTCCAGAGAAAATGTGGCCTGGGCTTTCGTCACTCGCCCTC<br>GGAAAGGGAGAGACCCGAGCTTTCGCCCTGCCGAGTTCTCAC<br>GCAGGAGGTACTTTGTAGGACTTCGCAGCGGAAGCGACTTGG<br>ACACAGGAAGAAGACAACCTTTTATGAACGTGGAAGAGAGCC<br>CTCACCAAGAACCAAAGCTGCTGGTGCCTTGATCTTGGACTT<br>CCAGCGTTCAGAACTATGCTGCCAGTGAGACTGATCCTCAAA<br>TCATAGCTTGAGAAGCAAGATTCTAAAGCTGTGCTTCCCAGT<br>ATGGAAACCTTTAACTACATATGGCTGTCAAGCATTTGCAGT<br>GTGCTAGTCTGAAGTGATCACTTTCGCGTGTGTGTGCTCAGT<br>CATGTCCGACTGTTTGCGACCGTATGTACTAAAGACCCCCAG<br>GCTCCTCTGTCCATGGAATTTTCCAGGCAGATTCTTTACCAC<br>TGAGCCATCAGGGAAACCCAGTCTGAACTGAGATATGCAGCA<br>AATGTACACACTGGATTTTGAAGACTTAGTCTAAAACAATGG<br>TTAACTGCTGGCAGGCCTCCTCCAGCTGAGCTCCTCATCCTC<br>CACTGTGCAGGGGTTACTGAAGCAAGAGCAAGCTTTCCCTCA<br>GTCCCCGGAACAGGAGCCAAGTGTGACACAAATTGTGAAACT<br>TGTTTCTGGTTACCAGGAAAACTTAAAGAAATCCTAATCAAG<br>CAAAAAATTGACAAAAATCTTTCCAACATTTGAACAAAAAAG
MSTRG.546.1	0.9	0.12	0.00847	-2.95	down	AGCTCAGAGAAGAGGCAGAGAGGGTTCTGACAGGTCAAATCT<br>GGAGAATGTGAAGCCTGCAAGGCACATCATAATTGTAACACA<br>ACTGCTTATTTTATACTCTGAAGGTTTTCAGTTCCAAGTGAA<br>ATCAATGGATAAATATTTTTTGAAACCAAATATTTTTCAATG<br>ACCATTTTAAAGAAATAGAATCATACTCATTTTGGGGGGTGT<br>GGTCATAAAAAAGTTAAAGAACAACAGTGAAGAAAGGCGTTA<br>ATCCTGTGGCAGAAAAGAACCCGATCTTCATTGTATCATGGG<br>GGAGCTAACTGTGGTGCGTGGACTCTTTAATTGTGGCTCATG<br>GGCTTAGCTGCTCCACAGCATGTGGGATCCTAGTTCCCAGAG<br>CCGGGATGGAAGCTGAGTGCTCCGCATTGCAAGACAGCTTCT<br>TAACCACTGAACACCTGAGAAGTCTCTGGATGGCAGTTTTTA<br>AACAGCCACTCTGAAGATGGGGATTTTATTGTACCACTTTAC<br>TTTTCCCTCCCTTCTGTCATAGAGTTAGGGAACTGCAACCAA<br>ATCTGGCCTTCTGCTTGCTTTTGTTAATAAAATTTCACTATA<br>ACTTGTCCAATTGTTATGCTCAGTTTTTTGGGGTTTTGTGTA<br>GTCTTGCTTTTGAGCTATAGCTGTGAAATTAAATAGTCTTGA<br>CAGAGGCCATGTAGCCCAATAAATAGTAAAATATTTACTACT<br>TGGCCTTCTTTCAGGAAATTTGTTGACTCCTGTTATTGTTAC<br>TTTTATACTATTTATAATCTGCTTTGTAACCATAATTCACGT<br>GCTTTCTGGTATCATTACATTTAAACGGATACAGAGCTCACC

MSTRG.43210.11	0.48	0.05	0.00344	-3	down	GGTTGTCAAGCACTGAGCTCCTGTGCAACTCTGGGAGGATCA<br>CATCATTCCACTTACGGGGCCAGAACCATGCCCTAGGAAAGA<br>GGAGAGAGCATTCAGTCTTCACTCTGCATCAGGGTATGAAAC<br>TGTGCTCCCTGTAGATTACTTTGGGTCTGGGTTCCCCTCCAG<br>TTTCTGTCTCTTTTCCAGCTTGGCAGAAGAGGTGAGACTCGG<br>CTGTCAAGATCACTGACTGTCAGCTGCTTTCCTTTGTGGGTG<br>ACTGCAGTTGCAAGTTTTCTCTGTACATTTTATTAGTTTAAG<br>AGCTCTTTCCTGCTTCCAATAATACAGGACCCAGAGAAGTAT<br>TTGAAGTGGAAGGTCCTGAAGATGAGATGACTGAAAATTAAT<br>GGAAGAGAATTGCTTTTCTACTGTGGGATACCTGCTTGGTGC<br>CAGGTTGCTAAGAGGGATGCTCTGGAATTCACCCCATTCGCT<br>ATAGAGGTTGGGTTCCACCATATTGACTGTGCTCATGCCTAC<br>CAAAATGAATAGGAGATTGGCCAGGTCATTCAAAGCAAGCTT<br>GAAGATGGCACTGTGAAGAGAGAAGACATATTCTGCACTTCA
MSTRG.111347.2	0.39	0.05	0.00366	-3	down	TCTCAGCCCTGCTCATTCCCCATGTACTCTCTCCTGGACCCT<br>TTGCTGCAGCATATCTTACTGTGAAGGCCACATGGGTTATGA<br>CAGACTCTGAGCTTTGATGATTCATAAACTCAGTTTTTAGAA<br>AATCTGGTCTGTTCTAACCACCTCAGAAAAAAAAAAAAAGAT<br>CAACAATAATGTCAGCTTATTCATATACAGTTTTGTCCATTT<br>CCTTGTCATTTCTCTTTGTTCCAGGACACCCCATCTCTGAAT<br>TAGAACTCAAAGGAAGTTGGAGCAGTCATCAGAAGGTTGCTT<br>GACAAGCCCATCAATACAGCAAAGAAAACTGAATTGTCATAA<br>ATGACATTTTATTCAGACCCATTTCTAACTTCCTCTTCTTTT<br>CAGTGAACAGAATTCTCCCCCTCTACCAGTGAATCTGAAGAT<br>CTGGGTTCAAGTGCAGACTCTGTTATTTATCATCACTGTGGT<br>CATTGACAAGGCATTTAGCCCCTTCAGCCTCTTGTTTAATGG<br>TCATCACTGTTGGTTTTCTATCAAAACTGCTTAAGGAGTAGC<br>TGTCTCTATGGATAGGAGAGAAGACTGTGCAAGAAGTAAAGG<br>GGAGCAAGAAGCTGTCACTATGCACTTCTGCACATGCCAGGA<br>GGGCCTTCAGAAGAGCTCTGGAACAGAAAGAAAGAGGGAGAA<br>GTAGAAATTTGAACAGCTCCTGCTGCAGAACTTACGATCTTT<br>TTTGCCCGGAGGCCTTCTACTGAAGAGATCCCAGATAAGGGT<br>GACCCATCAGAGAGGATCATCCATCTAGTTTACAGAAGCAAT<br>TTCAGAGTGGGCCCTGGGTCCTTGGACCCCGATGGAGGAAAT<br>CCAGGTGTTGTCATGTCTGGAGAGGATACTTGGAATCGCTGA<br>AGACATTTTTCCATCAGCCTGAGAATGGAGCCAACGACTGAG<br>GATGGAGGAACATAAAATGGGAATCCTCACCAGACTGGGAAG<br>GCAAGGAGAAGCACACCCTGACTGTGGCCCACACCTATGGGG<br>GCTATTGTTCATGACAGCGCTTTGGCTCTTTCCGCCATCCGT

MSTRG.166870.1	0.29	0.03	0.0033	-3.06	down	AGCTTCCACACCGGCGGAGCTCCAGCTCTGCGCTCAGGCGCG<br>ATAGCAGAGCCCCGGCCGAGAGGCTGCTCTGTGCTGGGCGAG<br>CCTCCCCAAGCCCTGCCCAGCTTCTAGCGTTCGCGCCCGTGA<br>AGGAGCAGGCTGCGGGAGTGTGGGAGACGTGAGTGGTGCCTG<br>ACTGGTGCCTCAGCTCAGCTCTCGCCTTCCCTTCGGACCTAG<br>GAGCCAGGCGTCCTTGCAGAGGGCTTAGGGGCTGGGGGTGTG<br>CACACTGCCAAAGCCTCCCGCGGGGAGAGCTCTTTCCGAGGG<br>AAAGGAAGCGCCCCAGGCTGCATTCCCAAGCTGGGGCTTCCC<br>CGCCCAGACTAAGCGCCGCACGGAGGGAATCAGGGTCCCGGG<br>GCCCCTGGCTTGGGGAGGCAGCTCCACAGCCAGAGTGTTGCT<br>CCGTTTGTGCCCAGAAAGCGCCTGCCAGACCGGGCCTTCCCT<br>GGAGCTGTCCTGCCGCCTAGAGGCAGGTGGATGTGCTGTGCG<br>GGCTTCCCCTGTGCGTTTGGGCTCCCTAGCACAGCCGCAGCG<br>CAGGGGAACCAGCCCATGATCAGTGAGCTCCGGGCAGTACTT<br>TGGTCCCAGGTTTGGAGCCCTATGCCCCGAACGCGGGAGAGA<br>AGAGGCTGGCTGCCGGCGGGTGCTTGCAAGCCTGTACTGGGA<br>AGGACCGAGTGAGCTTCCACACCGGCGGAGCTCCAGCTCTGC<br>GCCCAGGCGCGATAGCACAGAGCCCCGGCCGAGAGGCTGCTC<br>TGTGCTGGGCGAGCCTCCCCAAGCCCTGCCCAGCTTCTAGCG<br>TTCGCGCCCGGGAAGGAGCAGGCTGCGGGAGTGTGGGAGCCG<br>TGAGTGGTGCCTGACTGGTGCCTCAGCTCAGCTCTCGCCTTC<br>CCTTCGGACCTAGGAGCCAGGCGTCCTTGCAGAGGGCTTAGG
MSTRG.194482.7	0.18	0.01	0.00526	-3.12	down	CAGCTCTTTAGCCTGGCTGTCAAAGGTTTGATACCAGCTTCA<br>CGTCCTTCTCCGTGCAGCTCTGTGTGGTCACGGCAAACTGAT<br>GAGGGCCAGAGCCACTGACAAGTTTACAAGCTGATCGACTGC<br>AGGGTTAGTGGCGCAATCGCGAGACAGGGACGCAGGTGATGC<br>TCCTGCTGTGTTAGGTAGGTAGATAGGTAGCGGTGACGTCAC<br>GTGTTACGTGTGAGATGAGTCTTGTTTCTTGCCTGTGTGTGC<br>GTGTGACCGCTGCAGCTGCCGCGCTGGCCGTTCCTGTAAAAG<br>CACGCACCGTCTTCAGGCTCTCAGCCGCACCGTGGTTTACGC<br>AGAACGGCGGCCTTCTCTGACTGCACTCCGCATCCCTGTGAT<br>GTGATGCTCGTCCCCATTCAATCCTGGCACCAAGCTGTCCCC<br>ACGCTGGAGCGGGCCTCTGGACCTGCCCCCCCGTGTAGTCCT<br>CTGAGAGGAGCTCAAAACCGCAGGCGGGTCCAGTCATGTCAG<br>AACCTCGTACGCCGGCTGGTTTGGTGGCACTGTGAAGCGGCG<br>TGAGCCGTGGCTCACTCCTCCAGGGGACCCTGCTCTGGAATG<br>GGCTTCGTGGTCGTCAGGTCCAAGCTGAAACTGCCTTTGCTA<br>CACTCTTCATTCGCACATGCAGACAGCATGGGATTCTCTGGT<br>ACTCGTTGTTCTCTGTGACATTTCGCATTCGCTCCGAGTGCA<br>GCTGGTCCCAGGGCCGTAGAGAGGATGGTGCTGTGACTCGGC<br>GATGGCCACTGGACGTCCCCAGTGGGCGTGTCTCACACGTGA<br>TGTCAGCCTGAGCTGCTGTGGACAAGGGCATGTGCTGCTGTG<br>GTTAAGTGGGCCTGGACCCCCTTTCCCCCTTTCGAGAACATA<br>AAACCGAAAGTTGAACTGTGCCAGTGATTGTTTCTTGAATCC<br>TCAAGTTTACTTGGTTTTGTTTTCTCTCCGTAGAGTTAGAGC<br>TTTTCGTATCGTTTGGTTTTGTCTTTAATAATTATATTTCTG<br>TTTCATTTTTATTGAGCTAGTTAAAACTGAATCAAAGCTAGT

MSTRG.171301.2	0.27	0.03	0.00379	-3.21	down	GAAAGATTAAAGGTAAAAGGAGAAGAGGGTGGCAGAGGAGGAGATGATTAGATAGCATCACCAACTCATTAGACATGAATTTGAGCAAATTCTGGGAGACAGTGAAGGACAGGGAAGCCTGGCATGCTGCAGTCCATGGGGCACAAAGAGTCGGACACTTCTTAGCAACTGAACAACAACAGCAACCTATATTTCACATAACCTGAAAGGTAAAATAAAAATGTCAGACATGACTTAGTGTCTAAACAACAGCAACTTACACTGTCTCAAAATATCCTATACCTTTTTATGATTCTTTAGTAGTTTCTCGATGTTATTATGTGATTATATGTTTCTCTCCTATCTGGTTGTAAACTCATGTAGTATGATGTTGTGCTAACACATTCTAAGTACTAGTTTTTATCTGCTTAGTAAATGAATTAAAATTGAGAAAATAAGGCTGTCTGTGACTAGATTAAAGTTATGCTCTGAAATAGTGTTCTAGAAGCCATGTTGATCATACAGAGGACATTTTATTAAATTTGACTCTCTTTGATGGTTGGTTTATTTGTTGCATTTTTGAACTTTGTTTTCTCTGAGTCAGGTCCCTTATATGTTGGATAAAGATGCTAGTATCTACCTGACAGAGTTATGGTAACCATTAAATGGGATAAAATACCGGAATCTTATAATACAGCACTTTCCACATAATTGATGCTCAGTAAGTGACAAGAAGATCCAACCAGTCCATCCTAAAGGAGATCAGTCCTGAATATTCACTGGAAGGACTGGTGTTGAAGCTGAAACTCCAATCCTTTGGCCACCTGATGCGAAGAGCTGACTCATTTGAAAAGACCCTGATGCTGGGAAAGATTGAAGGCAGGAGGAGAAGGGGACGACAGAGGATGAGATGGTTGGATGGCATCACCGACTCAATGGACATGGGTTTGGGTAAACTCCGGGAGTTGGTGATGGACAGGGAGGTCTGGCATGCTGCGTTTCATGGGGTTGCAAAGAGTCAGACACAACTGAGCAACTAAACTGAACTGAAGTGTTAAGCTGATAATAAGC
MSTRG.226977.2	0.65	0.07	0.01421	-3.25	down	AGAGTATGTTTGTTTCACTTTAGATACATGGGGAAAAGAATCATAGCAAGGGTGCATGATGCCTGGCTGTCAAAACACTTTGGAGTAGACCGAAAATCACAAACCATGCCAGCTCTCCGAAACAGATCAGGAATAATGCAGGCCCGGCTTCAGCATCTTAGTAGCCTGGAAAGTTCATTTACACTTAATCACAGTTCTACAACAACTGAAGCAAACATTTTCCATCAGGCACTTCTTGAAGGAAATACTGCTATGGAAGTTTCCCTAACAGTATAGATACCATATCATTTTTCACTCAGTGTTTCAAGGAGTCCATATGGGATGAAGAAGCACTGTGGGAAGAGTCCATTGGTAGCCAGCACGTTTTGGGTCTGCTTGATAATCGAGTCCAAGCTCACTCTGCTTGCCACAAGACAGGTCAGTGAATCTGAAAAATGATGTGTTGAGGCAAGGAAAATACTT

MSTRG.119476.4	0.27	0.03	0.00161	-3.26	down	TTTTGCTTGCCACAAAGCTCATCCTTCATTTGTGCACTGCCAGCGCAAGCTTGGATCGCGCAAACAATCACAACCTCTTTTGGCTATAAGCTCTTTCCATTTGTCCCAGCTTCCAAACTGCAGACGAGCTGTGGCAGAGTCTGTCACGGCTGCACATACTTGGTTTCCGAGAATAACGCGGTAAATGAGGACAGCTACCACTGGGAATGCCTTCCCCCACCAACAGCCAGCCGAAATGAAGCTTCTAGTTGATCCCTGGGCTTCTCACCTTCACAGCCTCAGAGAAGTGCCTTTCACAGAACAGGCAGGTTTACGAAGTCATTTAAATGAAAACTGAGCAAAGACAGACTTCTTGTGCAGAGCAGAAGACCCTGAAGTCTTTTTCACTATGGACAACAGGAAAAAAAAAGCTTCTGAATGAAAAGCAATAAGCTCTCTGAAGTGTAAGTAACAGATTAAAACCCCACAATCGTAAACCTGAGCTCAGCTCCTGTACCATCACATACTATTTATGGAATACTCACGATACTGCATGAAAGAAGAAAATGTCTTCAAGAGACTGTTCCTGCCCTGAAAAAGTCACTTTGGGATTTACCCTAAAACAACCCATCAAGGATGTCTGCATGATTAATTAGCACTCTTACCGCAATAATCAGGTCAAATCTAAAGAAACCAACTATTTTGTGATCAAGAAAAATCTTTGTAAATTATTTATGATCACAAGAGGGATGGGAGGTTATCAGCAGAAATTGCGAAACAGTAAAGTGGTAAAAAAAAAAAAAAAAGAAAAAATTTAAGTGTCCTTTCAAGGGACTACCCGGTGTTGTCTATCAGATATTTTGGAGTTATCCGATAGTATAAATTCTGCGTCTTTGTCTCCGGCTAGGTTATTCTAAAACTGTAGAGATGGAAATTACCTTGTGGATAGTGCTGATGATGAGAAACAGGTCAATTCTGTCTGGTAGAGAATATAAACTTCCTTAAGTCCAATTCTCATTTTAATATCTTACTGCTTCCTAATAATTGGTGAATTAGTGAGACAAATAAAATC
MSTRG.224424.1	0.24	0.02	0.00311	-3.48	down	CAGGCCTGGAGGGAGGGTGAGAAGTGGTGGCAGCCTGCACCTGAGAGCAAGAGGAGAGGGGGAACCTAAGGTGGAGGTTGGGGAACCGGGTCTGCTATGGTGGGATTAGAGGCGATTGGAGGTGCACTGTGGTCAGAAGGGTCAAAAGAAGAGGAGGAAAAATCCGAACAGGGATCAGGAGATATAGCACTGGGGGGAAGTGGCCCAGGATGGGCTAAAAGTATTTGAGAAGTAGAACAGGAAGTACAGAGAGAGGGCCAAGGGTGGAAAGCGAAGAAAGCCTGAACATAAGGGATCTCTGACCACTTGCCATTGCGGCGGCTGAAGTTATAGAGGTCCCATAAAGTACTGGAATGGAGAGTTCCATTTTCTGGCCATTGGAACCTAATGTCCAGTCGAGATTGTGGCCAGACAGTGTTGGAGTGATAAGTGAGTCGCGTTGGCCTTATCTCTTCTTGAAAACCCAAGGTTTTTAGGTTTTGGAGAAGGCAGCCTAAAGGGATAGATTTTGTAGGTTGGGACTGAGAATTTCCGATTATAGACAAATAGGGAGGGAGGGCAAACTAGGGCGAGAGAAAAACGAGGAGAAAGGTCTCAGAGAAAAGGCACACCCACTCTCATGGGTGCCGGGGTCCAGCCCCGGCTGATCCAGGGTATTTGAAGGACAGACGGCGTAGGCGACATATTTATTTAAATACTTATCAAAGATATAAAGAGTAATAGAATGAGGATAGCTCAGTAGGAAAATTCAGTGGAGAAAAGAGGCTGAGTAGCTTGGTTTATGCAGGAGACCAATAAAACTTCAAGACAAGAAGTTTGCACCACTTACGTGGGCCATAGGCATCCTTCCGTTCTCCTGAAGGAGAGGAGACACTGAGGCCTCCCCAGTCGGATCTTAGAGGCCCAGGCATAATTAGTAAGCATGGTGGGTTCCGTGCTCCAGATGGAGACTCAGCCAGAATTTGAGAGAGAGAGCGACATGGGGAGACCAAGTTTCGGTGAACAAGGCCCACACTTTATTTTCCAAAGTAGTTTTTATACCTTAAGTTG

MSTRG.67346.1	0.33	0.02	0.00328	-3.51	down	TATGACCAAATTTATTTCAGGACAGTATAAATTCAGTTCCTCTTTCCCTCCAGAGTCTTCTAGCACTTTCAGATTCTAACACACATTTGCCATCTTTGTGGGCAGTATAGAGAGCACCTTCAGAATTTGGGGACATGGTCAGAAGGAGTCAGGAATTGAAGAATGCTACTAGAACCCTCATAATACATCTGAGATTAACCACTTGCAGATGTGACCTGTCTCAGTTTGGATGGCAGGTACTTGAGCTTGAACTGCCAACAGAAAACGTCAAGTTATCATCAGACGTCTGATGGCCCTCTCTGAGAGTCCCTTTAGAGCAAAAGGCCCTCCTGCTGGAAGTAAGTGCAAAGAACAAAAGGCATCCACGGATGATTTTAACCAAAGAGGAAACACGAATACAAGCCTCTTCTTAAAGGAGAAACATGGAACCAGAAATGAGTCTTCCTTTAAGAGTCTGGGAATTTTAATTTAGTAAAACTAAGGAATCACAGAAAATTTGTCACTCAAGTAGCTGACTCTAATTATGTGAGCTTCAGACTATGCTATATTCCTTCGCTGAACTCTTGTTTTGTGTCAGGCACAGTTCTCTCAGCACTGGAATGTAATGGAGACTCAAGCAGACAGGGTGTCTCTCCCCTTCAGAAGCACAGTCTAGAAGGTGGAAGACAATGAAGTAAGCAAATTAGTAAAGTTTCCTAACACAGAACCAAAACAGAAAGGAACAGAAAGGAGGCTACGGGAGAATCACGCTGCTATTAACAATGGCTGATAACCGACAGCAATATAAAAAGTGGAAGCGTCTTAAACTGCACTTAAAGTGCAGTCAGTGTCAGTTGCCCACATCATCATCAAAACACTATATGTAACAGTGATGCGGATTCACTGAGAAAAGATACCACGCTGCTGCTGCTGCTAAGTCGCTTCAGTCCTGTCCGACTCTGTGCGACCCCACAGACAGCAGCCCACCAGGCTCCTCTGTCCTTGGGATTCTCCAGGCAATAATACTGGAGTGGATTGCCATTTCCTTCTCCAGAAGATATTATGCTCAGT
MSTRG.37138.3	0.21	0.02	0.04164	-3.54	down	GTGAAGTCAGACCGCTCAGCTGAGACACTCCCAAGTTTCTGACCCACCAAAACTGTGAGTGAAGTCTCAGCGTGGATGCAAATGAATGTGCTCAGAGGCTTTCTTCAGAGAAGAAAATCAGAAGATCAGATCTTCATAGGGAGTTATAACCTTCACAAACTTTAAAGAAGTGAGGTGCTATTGTGAAAGAGCCTGCAGATTTTCCCTCCCTCCAGCTGTAAGTTAGTTAGTTGCTCAGTCGTGTCTGACTCTTTGTGACCCCGTGGAATGTGGCCCACCAGGCTTCTCTGTTCATGGAATTCTCCAGGCAAGAATACTGGAGTGGGTAGCCATTCCCTTCTTCAGGAGCTCTTCCAGACCCAGGGACTGAACCTGAATCTTCTGCACTGCAGACAGATTGTTTACCATCTGAGTCACCAAGAAGGCCCTTCAGCTGCGAGCACAGGAGCAAAGCCACAGAGAAGTTCATCAAATGGGATTGATTTTCTTCATGGGATTGAAATAATCTGGTCTAAGTGAAAAAATAGACTTTAAAAAATTGGATTTGAATCTAAAAGGGCAGAATCTGTGATGATCAAAATAACTGGCTTTATACTTGGATGTTAGTGCTATGTAGCAATTATTCTTTACTACTCTGATCCTTCTTGCTCTCGCTTTTATCTGCCATGCATTACACTTTTGTAATTAGGAGCTGATTAAGCCATGGTAGAATGTTTTTACTTTTTTCACTTCAAAAATTATTCTAGAATGTTTTTTCCCCATGCATATGTTTCACAAATAGCCTGATGACTTCTTTGGTATTGGTGTTCCAGTGATCACACTGAACTCAACTAATGTGTACTCCGTGGTGGAAGAAGCAAAATTCCTATTGGTTTAATTCAATCCATATTATATTGTGATATTTGTGCCTTAAAAGGGTAAATT

MSTRG.69346.1	0.22	0.02	0.00564	-3.58	down	TGAGCAAGTGGACACAGAAGTTCGAGCCTTAGGAAGGATGTC<br>TGGGATGGAAATCTGTATGGATTGGGAGTCACAGAGTTCGTG<br>GGAGAAGGACACCAACACCCAGGTCAGCACGTTAGGGTGAGA<br>GTAGAGACAGGGGCCGTGGACAAGACTCTGGGGGGCGTGATT<br>TCTGCGGCAGAGATGACAGAAAGAAAAGAACCAGTGATGATC<br>AACAAGGTCCTCCTGTACAGCACAGGGAACTATATTCTATAT<br>CCTGTGATAAACCACAAAGGCAAAGAACATGAAAAAGAGTAT<br>ATAGTGTAACAGCGAGTTTGCTGTACAGCAGAAATTAAACAC<br>AACATTGTAAATCAGCCATACTTCAGTACATTTTCTTTAAAA<br>GGAAAGAAAGGAGCCAGTGAAGGAGGAATCAGGTTAAAAGAG<br>GGTGTTTTCAGGGAAGACATGGGAAGAGCGTTTCAAGGAGGA<br>AGAAATAATTAGTGGCAAACGCAGGTTAAAGGGATCTAGTTC<br>TGGCGCTTGCCAGCTCTGTGAGGTTGGGCAAAGTACTCGACA<br>TCTCTGTCGCTCACTTTCATCTTGCATAAAAGGCGCACACGC<br>ATTGTAACTGTCTCACAGGATTGTTACAGGAATTAAATGGTG<br>TAAAGTGTCTATAAAGTGCTTAGAACAGACCGTGGCACGAGT<br>AATTGCCACTGAATGATGACTTCACAGTAAGGTCAAAATGAT<br>GACTTCACAGTAAGGTCAAAATGATGACTTCACAGTAAGGTC<br>ACATAAAGGCCCTGAAGCCCTAGTGGACTTGGGAGGGGTTTA<br>TTCATCCCTCTTAGGTCTGTGTCCCTACTGTGTTTTAGGCAT<br>GTTAGATAGTTACTCTTCAGTCAGTAAGTCGTGTTTGACTCT<br>GCGACCCCATAAGCTGCAGCACGCCAGGCTTCCCTGTCCTTC<br>ACTATCTCCCAGAGTTTGCTCACACTGATGCCCATCGAGTCG<br>GTGATGCCAGCCAACCATCCCATCCTCTGTTGCCCCCTTCTC<br>CTCCTGCCTTCAGTCTTTCCCAGCATCAGGGTCTTTTCCAGT
MSTRG.221229.2	0.28	0.02	0.04475	-3.58	down	CTCTTGCAAGCTATATTTGACACATGGATTGCTTTCATCTTG<br>TTATTTTGTTTCTGTACAAATAATATTTATCCCTCAGGACAA<br>GAAATCATCCATGTGGAATAAAGAACCCCTTCTCAAGGTATT<br>TAATCAGAAGATCTAAGAGAGACTTCAGGTGAGAAGATGGGA<br>CTCAGACAAGGGATTCTTCAGTTGACTGAGCACTAGGTGGAG<br>GGACCATGTGATAAGAAATGAGGCTTTCCTCTGGGAGTTGAA<br>ACTGACCCCTGGCAGATAAGAAGAAAGGGGACAGGGACCTCT<br>ATCCTAAGACCACAAGGAATCAAATTCTGCCACAACTACATC<br>AGTCTGGAAGATAGTACATGCTGGATGATTATGGCTGACACC<br>TTGAGTTTAGCCTTGGACTGCAAGGAGATCCAACCAGTCCAT<br>TCTGAAGGAGATCAGCCCTGGGATTTCTTTGGAAGGAATGAT<br>GCTAAAGCTGAAACTCCAGTACTTTGGCCACCTCATGTGAAG<br>AGTTGACTCATTGGAAAAGACTCTGATGCTGGGAGGGATTGG<br>GGGCAGGAGGAGAAGGGGACGACAGAGGATGAGATGGCTGGA<br>TGGCATCACTGACTTGATGGACGTGAGTCTGGGTGAACTCTG<br>GGAGTTTGTGATGGACAGGGAAGCCTGGCGTGCTGTGATTCA<br>TGTGGTCGCAAAGAGTCGGACACGACTGAGCTACTAATCTGA<br>TCTGAGACTTGGAGCTGAGAACACAGTCGCATCACTCCCAGA<br>CTTTGACAAACAGAAACAATGAGTAAATAAATGAGCGTTATT<br>TTAAGCTGCTTAGTTTGTAGTCATTTGTTATGCAGTATTAGA<br>AAATTAACACAGTGGCAGGGCTTCCCTGGTGGCCCAGACTGT<br>AAAGAATCTGCCTGCAATGCAGGAGACCCAGGTTTGATCCTT<br>GGGTCAGAAAGATCTCCTGGAGAAGGGAATGGGTATCCATTC<br>CAGTGTTCTGCCTATGGACAGAGGAGCCTGGTGGGCTATAGT

MSTRG.170006.2	0.73	0.05	0.02381	-3.68	down	TTCAGAGCCGCTGAGAACGAGCCTGCCCTCCTCGCCCCTTCAGGACCGCGGGAGCGCGTCTGTCGCGCCCTTACGCACCACCACCCACGCCCGTCGGGGAGGGCCAGAGCGCGCCTGCCCCGCCTTCAGCCCGTCCTCCTGCCCCTCAGAGCTACAGGAGCGCCTCCAGCTTTTCAAGTCTGCCGCCATCTTTACGGCAGCCAACTTCCCAGCCTGCCTCGCGCTCAGGGAGAGGGAGCTCGGGCCGACAGGATACGGAGGGCGGGGCCGGCGGGACGGGTGGGACCTGTGGGGAAGGCACTGGGAAGACTTCCTATGTTCCAGACTTCCTATTCAAACCTGTCAGGGAAGTTTCAATGCAACAGAACACGTCCGGACCCTACAGAATAAAACTTCGAACTTTAACCAGTCACCTAGAAGTAACAGCAGTCTTACAATGAGATCCGGACGGATTGGTTTGCCCAAAAGTACACTGCAAATTGGAGTTAGCCGCAGCAGGCCCTGAAGAAGGCGATGGCAGCCCACTCCAGTACTCTTGCCTGGAAAATCCCATGGGCGGAGGAGCCTGGTGGGCTGCGGTCCATGGGGTCGCTAAGAATCGGACACGACTGAGCGACTTCACTTTCACTTTTCACTTTCATGCCTTGGAGAAGGAAATGGCAACTCACTCCAGTACTCTTGCCGGGAGAATCCCAGGGTCGACGGAGCCTGGTGGGCTGCTGTCGAACACGACTGAAGCGACTTAGCAGCAGCAGCAGCAGCAAGCCCACTTTTTTGCCACGACTACGCTGCCAGTAAGAACCTTGCAGCACAGGTAGCATGTAAACTTACAGCAAACAAACCGCTGTTTGCCTCAACTGATCTTTTTAAAGAGTATTGCCTCCTGTAGCAGCGGTCAGTAGATTATCTCTCAGCTTCACATTCACTCTCAGTTCTCATCGCGGGATGGACCTGGGCCTTTTGAGCATTAGTCCTTTGCCAAGTAACCCAGTGTGAAGCTTTGCCAGGAGAAACTGCTTGGAGGCAGTGTGGGAGGGAAGGGGCTTTGTT
MSTRG.110319.4	0.21	0.01	0.0031	-3.76	down	CTAAGAATTATCTGCCCACAGAGCCACAGGCTCAGACCAAAGAACCTGAGACCCTGCTGCCTTGGCTAAAGCCAAGCTTTTTAATTTTCTTTTTTAAATTTTTGGCTATGTAGGATATGAGTTCCCCAACAAGGGTTTGAACCCACACCCCCTGCATTGGAAACATGATGTCTTAACCACTGAATTGTTGGGGAAGTCTTTGAAGCCAAGCTTTGTCCCCTATCTACAGCAGTTCCAGTGTTGGGCAGGAAGTGAGCTGATGCTGGCTTCTCTTTGTCTTATTCCAAGTTGGGGCATGAGATTTTCTCTGCATTAGAAGGTTGTTGAGACCTGAAGCCTGGGAAGATGCATTGAAAATTATACAGGCGCTCGTTGTGAAGAGGTTTTTCTCCCAAGCTCCAGTATCCAAACTAAAAGTGACCTGTTCACAGCTTTCGTGGCATTGACTGTTCTTCTGGGAACTCTTATCCTTGGAGCCCTCTACTTCCTCTGCAGGAAGGGCCATCTTCAGAGGGCCAGTTCAGTCCAGTATGATATCAACTTGGTAGAGACAAGCGGTACCAGTGTCCCCCACAGTCATGGAGAACATTGAAGAAATAACACAGTGAAGCCAAATCATTGCTTCTTTGTTACAAAGGCAGAAAGTGAAACACTAAATGGTTTTTCATAAAGCAAGACTCAAATTGTTTCTACAAGTGAAAACCAAGTCAGTCATCTAAAATCTCAATTTCAAATCAAGAGGATTTAGAGTCTTCTGTTTTTATTAAAATGGAATTTATGGTGGTGTTTAAATGGGTTAGGCTGTGTGGGCACAAAATGACACAAGTAGATTTAATTATTTTCTCAAAAATTCCTGTAGAATGCATAGGAAATTATCAGATTTGTAGAAGTTTATTTTACATTCTTTCATGTTCACTGTGTCAAATTTGCAGTATAAATCAGTGGCAAAACCTTTGCTGTTCGGCTTCTCTTACTAGATTGAAAAGTGGTTTTATTTACCAGTGGTCCAGGTTTAAGCAAAGTTGAGAATTACAGCAATGAACTGGCCTT

MSTRG.231934.38	0.11	0.01	0.01707	-3.84	down	GAATCCTTTGGGGACTTTGGCTTATCCTGTGTGGGTCTCAGG<br>TCTCAGAGCCGCATTCCTCCTCGTGGTTGGGTCTTGGAGGTA<br>GAGGACTAGCCATGGGCCTACCTAGCTCCTCGTGATCCTTTT<br>GGTAATGCACACAATTGATATGGGTAAGGTCAGTCCTGGTGG<br>GAGTTGAGATTGTAAATGACAGGCCTTGGTCCAGAGCCCACG<br>GTTTTGTTACCTTTCCTTATCAGTGTGAACCAAGTGCTATAT<br>GATGATTAGATGGAGGTTTAGTTCACTTTTAGTCAGGAATAT<br>GGAATAAAATGGCAACCCACTCTAGTGTTCTTGCCTGGAGAA<br>TCCCAGGGACAGAGGAGCCTGGTGGGCTGCTGTCTATGGGGT<br>TGCACAGAGTCGGACATGACTGAAGCGACTTAGCAGCAGCAG<br>CAGCATGGAATAAAAAGTCATAATTGCCATAAGGGCCCCTAA<br>GATACCAAGTTTACCTCCATTCAAAGATAGGATATTGATTGA<br>GCTCTAAAGAGGGTAGGCAGCTAGGAGGATCAGCATTAGTTC<br>AGATCTGAGTTCTAATGCTTTGGTATATTTACTTGGTGCCTC<br>TGTACCCAAGATGTCTCATTTTAAAAAAAATAAATAAATATT<br>TAACCCATAAAAAAAATAGAGGCAAGGATCCTTATTAAATGT<br>GTGTGTTGTAAGAGTCTTTGCCAGATAAAGAAGATAATTGTG<br>ATGCTAATAATAGTGCTTGACAAGAGTGAATTCTGAGGTCCA<br>TGGTGTGGACCTAGATGACTTGTCTTAAGGTGGTAAAAGTAA<br>AATTAAAGCTAGATGTCATACTTTGACTGTTCTTACCTTTGT<br>TTCTTCTTCCTTCTCTCTATCCTGCAGGTGACACCAAACTTG<br>ATCAGAAACCATGCCACAGGTGAAGAGGTATATTGAGCCCTG<br>CCTGGCCAGTGAGGTTACTAGAGAATTGGAAGAGGCTCAAGT<br>CAGCATCCTGCACATGGTTATCAAACCATTGTGAAGTCTTAA<br>TTCTTGGGCAGCTAGAAACCTTGTCACTGGTCAAGAGCAACA
MSTRG.148892.2	0.65	0.05	0.00629	-3.89	down	CACAGTTACTTTGGTCCCAGGTTTGGAGCCCAATGCCCGGAA<br>CGCGGGAGAGAAGAGGCTGGCTGCCGGCGGGTGCTTGCAAGG<br>CTGTACTGGGAAGGACCGAGTGAGCTTCCACACCGGCGGAGC<br>TCCAGCTCTGCGCCCAGGCGCGATAGCACAGAGCCCCGGCCG<br>AGAGGCTGCTCTGTGCCGGGCGAGCCTCCCCAAGGCCTGCCC<br>AGCTTCTAGCGTTCGCGCCCGGGAAGGAGCAGGCTGCGGGAG<br>TGTGGGAGCCGTGAGTGGTGCCTGACTGGTGCCTCAGCTCAG<br>CTCTCGCCTTCCCTTCGGACCTAGGAGCCAGGCGTCCTTGCA<br>GAGGGCTTCGGGGCTGGGGGTGTGCACACTGCCAAAGCCTCC<br>CGCGGGGAGAGCTCTTTCCGAGGGAAAGGAAGCGCCCCAGGC<br>TGCATTCCCAAGCTGGGGCTTCCCCGCCCAGACTAAGCGCCG<br>CACGGAGGGAATCAGGGTCCCGGTGCCCCTGGCTTGGGGAGG<br>CAGCTCCACAGCCAGAGGGTTGCTCCGTTTGTGCCCAGAAAG<br>CGCCTGCCAGACCGGGCCTTCCCTGGAGCTGTCCTGCCGCCT<br>AGAGGCAGGTGGATGTGCTGTGCGGGCTTCCCCTGGGCGTTT<br>GGGCTCCCTAGCACAGCCGCAGCGCAGGGGAACCAGCCCATG<br>ATCAGTGAGCTCCGGGCAGTTACTCTGGTCCCAGGTTTGGAG<br>CCCAATGCCCGGAACGCGGGAGAGAAGAGGCTGGCTGCCGGC<br>GGGTGCTTGCAAGCCTGTACTGGGAAGGACCGAGTGAGCTTC<br>CACACCGGCGGAGCTCCAGCTCTGCGCCCAGGCGCGATAGCA

MSTRG.83783.3	0.32	0.02	0.02356	-3.89	down	TCCTTGGGCGCTCCCGGAGACCCCCAGAACGGGGGGCGGGGG<br>GCTTTCAGGATGGTGCGGACACCCTCCGCACAAACCCCAGCC<br>ACAAGTTTTATTTTCCTTTCGCGCTGGAAGTTCTGTCTGGCT<br>CTCGATCCACACCGGCACGTCTTGCGCGGCTCCCCGCCCTTC<br>CCCGGCAGAGGAACTTTTGTATCCACGTCCATAAGTCAATCA<br>GGCTGCAGTTTTGCCTTCTCATGTTGCCTTTCTCTGGTTATA<br>CTGGCCTCAAAAAATGAACTGGTAAGTTTCCTATAAACAGCA<br>TACAATTGGACCTTGTTCCTTTATTTATTGGGTTGACTGGTA<br>TATCCATGGCCTTATTTCATTTTTTCCTATGATGAACACTTA<br>TAAACCTGCTATTCAACCAGAGACTAAAACAGCAACGGTGAC<br>TGACAACTCCTTGTGCGTTCCTCCCCCGTTTGCTCACCTAAA<br>GAAACTGCCAGTCTCAATCTTGTCGCTGAGCTGATTAAGAAG<br>AAAGCTGACTCCTACCTTCCAATCTTGTGTCCATTTCATCTG<br>CTTCCAAACATAAAGCCGCCCGCTGCTTTTGCAAGATCTTGG<br>CTGGTTTAAAAAGGCAGATTCGGGATTTGTGTGCAGGATTTC<br>AGGAATGTGGGGTCTTCTGGTTTGTTTTGTGTGTGGATGGCA<br>CAGCAGAAGGGAAACAAAGCATTCTGCTGCCTCGCATGGAGG<br>TTCTTCTAGTAGCCAGGCGACCTTGAGCGGAAAAGCCACCCT<br>CAGTCTCCAGTACCCCATCTGTAAACTGTGGGTCACTATGGT<br>TTTAAAGTATCTTGGTCCTCCTGCAAGAGGTGGAGCCTGATC<br>TTCCCACCCCCACCCCGGGTCCCCCAGCGTGGGCTGTACTTA<br>GTCACTGGGTTCTGATGAATAGAATGTGGAGGAAATGATGGT<br>GGATGAACTCCAAGGCTGGATCAGTAAAGGCACCTGGCTCCC<br>GCCTGGCTCCATCTCTCAGGTCACTCTAGGGAAAGCCAGCTT<br>TCATGCCGTCAAGATGCTCAAAGAGCCTGATAGACAGCCCCA
MSTRG.103983.4	0.17	0.01	0.0282	-3.91	down	CTGAAGTCCTCAGGGAACTTGTAGACCGGGATCTTGGCTTCA<br>TGATAATGCTCATTTTGGAGGAAAAAGAAAAAAAAAAAAAAA<br>ACTCTTCAAGAATTCTGATTAATACATTTCAGCTGTGTTTCT<br>ACAAAGATGGCTTGAAATATATTCCATTTTCAAAGGAGGTTG<br>TTTGAAGAAATCCACATTTCCCGGAATCCAATTTCTGCCCCC<br>AGTCCAGAACAGAGAGAGGTTTCTAAAACACAGAAGCTTTTT<br>CCTCTTTCATACTGAATTTACTCCATCACCAGGCTGTTCGGA<br>AGTGACAGTTTGTTTTCTGCTTTGCACTGCCAAGGGAGGAAC<br>TGGATTTTGAATCTATTCTTCATCTGGTCACAAGGGTAACCA<br>ACAGAGTGAAAGAGCCACTTGATCGCATCTCTTTTGCATTTC<br>CACAATACCAGGATTAAAGGTTTCCGACCGTCTAAAAGTGCT<br>CAGGTTAGAGAAAAGGAAACAGAAATGACCGCGAGGTACAGT<br>AACGACACCGGAGAGCTGTCAGGGAAGTTCCATTTCAGGACG<br>ACCACAAAAAAACACTCCTCTCGGGGGAGCCAAGAAGTCAGA<br>GAGGGAACAAAGCTCTCCACACTTGAGCTGGACTCACTGTTG<br>TACCTGCGAACGTAATTTCCGCTGTGTCCCCTGATTAGAATC<br>TGACTCCATGCCTTTGTGAATGAGAACTATCATTCAATCTCA<br>CCAGGCAGAAACTATTCCTCAAGGGCTGTATGACGTCTGTAG<br>GAAGGCAGTTCAAAACATCGCAGGGAAGTGAGAGAGAGGGAA<br>AAAAAAAAAAAGACCCAGCGCCCAGCCCAGCGGGTCTAGTCA<br>ATTGCTGAAATTTACAAAGCTTCTTTGCAGATCAGCGCCAGA<br>GAGGAGGAGTGTAAACCAGCCAAACAGAAGACATCTCGAAAC<br>TTATTTCCTCCTCTGAGAAATGAGGTGTCTCTTGTTATTCAA<br>GGTCTTATCATATTTGAATTTGTAGTGTTCTTCACTGTGGAT<br>TTCACCCACTGGAACAGAACTTCCATGAGCACAGGGATTGCC

MSTRG.90432.1	0.45	0.03	0.0425	-3.94	down	TTTTTGAGTCAAACCAATTCCTACCCTCTCTGCAGAAGTTGATGATGCACAAGATTATCACAGGCAGTCTGTCTTCCAGCGACAACCAGCCATGGGCAGCCTTCTACCTGGTGGGACAGATTTCAAGGCCTGCTGTTCTGCTAAATGCTGAAACAGCGTCTGGGGTGCCCACCCGTTCAGCGCACTCTGTGACATGGTGACAGCGTCTCCTCGTCTGCAGAACTTTCCTCAAGAACGGACCAAGGACAAGGGCGGACAGAGCTTGAAAGACGTGAACGCTGTACCCCTCCAGGAGGAATCTCCCATAATTCTCCAGTTCTAGGGAACAACAGAGTCACCTACTGACACAGTGGGTGTTTTGGTCAATTTTTGTCCCTCCAGCGAAAAATTCATGTGTCAGAAGTCCAGTGGTTAAGATTCCCCACTTCTAATGCAGGAGGCATGGGTCCGATCCCTGATTGGGGTGTGCAGGGAGTGTACCCTGGAGCCTAGCACCCTCCGGAGAGGTCCTGCTGACCAGCCGAGGGCAGCCGCCTTGCTGGGGGACGTCGTCTGGGAACCACCACTGGCCGGTTGCCGTGGGTTCAGGAGTCCGAGTGCACAGAGGTTGCCTTGGGGATGCTGGAAGAGGCAGCATTTCTGGCCAGCCCAGGTGAGCAGAGAATACTAGCGGGAATAAGAGCAGACTCGCCAGGCCACGGGGAGAGGGCTGCAAAATTACTCACACAGCTGCTCTATTTGTCTGTAAGAGGAGCAGTCGGTCTCCACCAGCTCCCACCCGCGGGATGGCTGGGATAAAGGGAGTGGGGTGGGAAAGTCCTTCACTGCAGCCTCATCAGATCCAGTAACTCATGCTTGAGTAACTCAGAGCTTACTGGGATGACGTGGGTAAGTGCAGACGTGGAGGTTCATGTTATGATGAGCCTGGGAGTGAGTGGAAGAGGGGTGTGGCTAGCTGTGTCCCCAAGCCAGCGGCGACAGCCGCCCCTGCCTCTAGGGATTCTCCTTGATCACCTGTCTTGGCTGAAAATGTCCGCGGTTGGTTTGCCT
MSTRG.187574.3	0.17	0.01	0.01195	-4.07	down	GGAGCGTGAAATAAATGCTTCTCAGTAGAGTGAGCGCAAATAAAAATTAGCCCCGCTTCCTCTCCACACCTCCAGGAGATTGCGCAGGACGCAAGTATCCCCAGACTCCAAGGTTCCGAGACGCGCATCCCCTGCAGGCAGCGGCCACTCACCTCGGGTGCGGCGCCGCGATGCCTCCGAATCAGACGAGCGCTGACAAGTGACTCGCGGTGCTTGCGCCTTCTCCACGGCCCGCGGGAACTTCCGCCTCCTGGCTCCCGCCCCGCCCCCCCCCCTCCAGCCCCAACACACACACACACCGGCCCCGCCCCCGAACCAGGACACACCCCCTCCGCTCCTGGCCACGCCCCGGACCCGCAGAGCTGCGGGGAGCTGAGAGAAGGCTGCGGGTGAACCTAGCTGAGGGCTAACTGCAGCGAGTCGGAGCTGCTGCGGAAAGACAATTGCAGTACGGAGTTTCGGTGGGACCAGTCTTAGGGGGTTTTGAGCAGTACCTGACAATGGTCCGCTCTGCCTGTTTGTTCTGTCGTGGATGCATTTTTCTCTCCAGAAACTGAATTGGCAAGCAGTTGCTTTGGCTAAAAATAGGAGAGGGCTTTGGATAAATCCGTATCTATGCAAGTTCAGCGGCCACTGCCTGGTTTTTTGAGCAAGCTTTATGTGCCCGGAGAAGTGAAGACTTTAAAGTTGACCTGTTAGGAAAGGGAAAATCAGCAACTTTAATATCTAGAAGTTAAATCCTCTAGTCAAACCAGAAGACCGTGTTTAAGAGCAGCGGCCAAAGGTGGTTCAACCATTATAACCTCAGCTTATTTTTCTAAGTCCAGATCAATAGTCCACTTTCCAGTTTCTATAAAGGTGCTTGATTTAGCCCAAGGGATTTATTCATGAGCAAATTTGAATAACGTTCAGTGAACATAAGAGAAAGTAACTTTTAGGCTTGTTTTGAACTGTAAATTGGCTTATAACATGTGAAAAGGACAGTTCTTAACCATGGAACTTGTAAAGCAAGGCTGATGGAGTCCGTGGGTCCCCAAGAAGTGCAAACAA

MSTRG.67346.3	0.37	0.02	0.00953	-4.16	down	GGACAGTATAAATTCAGTTCCTCTTTCCCTCCAGAGTCTTCT<br>AGCACTTTCAGATTCTAACACACATTTGCCATCTTTGTGGGC<br>AGTATAGAGAGCACCTTCAGAATTTGGGGACATGGTCAGAAG<br>GAGTCAGGAATTGAAGAATGCTACTAGAACCCTCATAATACA<br>TCTGAGATTAACCACTTGCAGATGTGACCTGTCTCAGTTTGG<br>ATGGCAGGTACTTGAGCTTGAACTGCCAACAGAAAACGTCAA<br>GTTATCATCAGACGTCTGATGGCCCTCTCTGAGAGTCCCTTT<br>AGAGCAAAAGGCCCTCCTGCTGGAAGTAAGTGCAAAGAACAA<br>AAGGCATCCACGGATGATTTTAACCAAAGAGGAAACACGAAT<br>ACAAGCCTCTTCTTAAAGGAGAAACATGGAACCAGAAATGAG<br>TCTTCCTTTAAGAGTCTGGGAATTTTAATTTAGTAAAACTAA<br>GGAATCACAGAAAATTTGTCACTCAAGTAGCTGACTCTAATT<br>ATGTGAGCTTCAGACTATGCTATATTCCTTCGCTGAACTCTT<br>GTTTTGTGTCAGGCACAGTTCTCTCAGCACTGGAATGTAATG<br>GAGACTCAAGCAGACAGGGTGTCTCTCCCCTTCAGAAGCACA<br>GTCTAGAAAAGTTTCCTAACACAGAACCAAAACAGAAAGGAA<br>CAGAAAGGAGGCTACGGGAGAATCACGCTGCTATTAACAATG<br>GCTGATAACCGACAGCAATATAAAAAGTGGAAGCGTCTTAAA<br>CTGCACTTAAAGTGCAGTCAGTGTCAGTTGCCCACATCATCA<br>TCAAAACACTATATGTAACAGTGATGCGGATTCACTGAGAAA<br>AGATACCACGCTGCTGCTGCTGCTAAGTCGCTTCAGTCCTGT<br>CCGACTCTGTGCGACCCCACAGACAGCAGCCCACCAGGCTCC<br>TCTGTCCTTGGGATTCTCCAGGCAATAATACTGGAGTGGATT<br>GCCATTTCCTTCTCCAGAAGATATTATGCTCAGTATTAAAAT<br>GAAATAACTTTGAAAGATTTTCACCCTGACTAAAACACATGC
MSTRG.184700.12	0.23	0.01	0.00112	-4.3	down	CCCGGACAAAGGTCCTTCGCGGCGCAGGTCGCGGACGTGGAG<br>ACCGTGTCCTGCGGGTCACTCTGCGGGTTGGTCCGATCGTTC<br>AACCCGGGGAAGTCGGAGCGAGAAGAGGACGAGCGGCGCGGT<br>CGGAGCGCGGTGCGGGACCCAGGGAGTGGGGCCTGGCCGTGC<br>GCTTCGCCCGCGCCGTCCCGGAGCTGTCCCGCCCTGGGAAGA<br>GTTGTGGGACATGGCATGGCTTTGCTCTTTGCCCCTTTCGCC<br>GCGTGAAGGGGAGAATCATTTTGGAAGCCAGAACTCGGACCG<br>ATGGGCCAGGGCCTGTGCAGCCGTGGGCGCGATAGCGCAGCG<br>CCGGCTGCGTCCGAGCGCGATCCTCCGGCAAGGGCAGAGCTG<br>CAGACCAGCGCGCCCCCGGCTCGATCGGGTTCAAGGCGACTC<br>TTGAGGACGGTACCATCAAGGTGCGAGCCTGGGAGAGGCTGG<br>CCTCCCCTGCCAGACCAACTCGGTGACGAGTCTGCTGTTGCC<br>CGCAGCCTTCGGTGTGGATCCACCGTGTGCCTGCAAGGCTCT<br>CGGGAACTCCACATTCGTTTGCTCAGACAGACAACAGCTTTG<br>GACGGTATGTGTAAATGGCTGCTTCTGAACATACATCATGGA<br>CGCCTCAAGAGTCTAGGCTTCACAGCCCAGAAGGTTCTCTGC<br>GGCCCTGATTGGCTCTAGAGCCTCGGCTTCTCCCCCGGACTC<br>GCACCTCTGCCCCCAGATGCTGTGTCTTAGAAGCCTGCGCTC<br>ACCGGCACCCTGCCCACACTGCCTGCCCTGGGAGCCGTCAGC<br>CCCCTGAGGCCCTCCTGGAGCTGTCTTGGCCACAGTGGGGCG<br>GCAGAGTGGGGCACACACCCAGGGTCTGTGCATCTGGGAGCC<br>CCCCTGGTGGGGGTGGTGCTGGAGGACATGGCCTGCAGGCTC<br>GCCTGCCAACTTCTGACCCCTCCCGCCGTGTCTCAGGCCCCT<br>CCGAGGGCTTGAGTCCTGGCACAGCGCCACCCAGGGTGCCCG<br>CGGCACCTCCGCCCCGCGGTTCGATAGCACTCAGCGTTTCCA

MSTRG.53914.1	1.27	0.06	0.00298	-4.38	down	AGGCAATGGCACCCCACTCCAGTACTCTTGCCTGGAGAACCC<br>CATGGATGGAGGAGCCTGGTGGGCTGCAGTCTATGGGGTCAC<br>TGAGAGTCGGACACGACTGAGCGACTTCACTTTCACTTTTCA<br>CTTCCCTGCATTGGAGAAGGAAATGGCAACCCACTCCAGTGT<br>CCTTGCCTGGAGAATCCCAGGGACGGCGGAGCCTGGTGGGCT<br>GCTGTCTATGGGGTCACACAGAGTCAGACACGACTGAAGTAA<br>CTTAGCAGCAGCAGCAGCAGCAATCATTGACCTTGGGAAAAG<br>CTAGCCGCCATGTCCTGGGGTGTCTTCAGAATCTCTCCCGAG<br>ACGCCCAGATGGTGAGGAACTGGGCCCTCAACAACATGAGTG<br>ACTTTGGAAGCACACCTCCTCCAGCCCCAGCTGAGCCTTCCG<br>ATGACTGCAGCCCTGCTGACGTTGTTACGGAACAAAGGTTTG<br>TGCGCCCGATGCACAGTGGGGCCAGAAAAACTGAGACACTGG<br>AGTCTGGACCAGGGAAAGGCTGATTGCAGCGCCAAGCAAGGA<br>AAACAGGCGGCCTGTGCTCAAAACCCCTGAACTCCCTGATGG<br>TTTGGGGGGAGAAGGTTTTATAGGCAAAATTTGGACTCAGAG<br>TTGCAGGGTATGTGACTTTCTTCTGATTGGTTGATGGTGGGT<br>GGATAGGGCTGTACTCCTGGCATCTTGGGCTCAGCCTGAAGT<br>TGCCATCTTCTGTCTGGGGAGGGGCCTTCGTTCTGCCGAAGG<br>TACTGTTACCTAAATTCATTGAGGAGGAATCAGGATCCTGAC<br>CCAAGGCTAGTTGGGGTATAGTTGATTTACAATGCTGTGTTA<br>ATTTCAGCTGTGCAGCAGAGTGATTCAGTTATACATACATAT<br>ACATTCTTTTTTAAATATTCTTTTCCATTGTGGTTTATCAT<br>AGGATATTGAATATAGTTTTCTGTGCTATACAATAGAATCTT<br>GTTTATCCAGTTTATATATGAAACCTTACATCTTTTAACCCC<br>AACTTCCCACTGCATCCATCTCCTACCCCCATTCCCCTTGGC
MSTRG.153391.3	0.51	0.02	0.00127	-4.57	down	CCAGCTTGCAGCGAGGAATCTTGGAAGCCAGTGGGTCCAAGC<br>TGGCCAGGGAAGCGCCTTGCACGGGGGCATTAGGTGTGAAGT<br>AGCTGCAGGGGTGTGTCTTCGTTCTGCTCTTTCTCTTTCGGG<br>TGAGGCCTCACTCTGTCTCGCCTTCAGGGGCCTTCACTTCAT<br>CCCATACTTGAGGCTACTTTATAAGCCCCAGTCACCTACAGA<br>TTATCCCTTACGATTCCAAGCTGGTCTTGAGTTCCTCTTCTC<br>GTCTATCTCCCGCACATCCAGGCCCTGGAATTCTTCTCTGTC<br>CTTTCTCCCAAGAGGAAGTGACACCAGAGAGGGACACAGAGA<br>AAAGGCTACTTAAGGACACAGCAAGAAGGCAGCCATCTGCAA<br>GCCAGGATGAGAGGTCTCTCTAGAAACCAATCCTGCCAGCAC<br>CTTGATCTTGCTTGACTTCTAGACTCCAGAATAGTGAAACAG<br>TTCCTCAGAACTTCTGAGCATCTGTCTTCCAGGCTATGATCC<br>TCAGTTTGGCTCGAATAAGATTCCCTCTTTTCTTCTGAACCT<br>GATGGGGGATGAGGATTCCAGGCAGTGAGAAACACTGTGCAA<br>AACATCCTGAAGTGAGAATTTGATTGGTGGTTTTGTGGAACA<br>ATGAAGAGGCCCAAGTGTGACTGTTGCCAAATGTGACTCCCA<br>TACCCCAACAAATGGAAGATGTTAACCACTTGATGACCAGGA<br>GCACACAGGCCCAGGCCTCCTAGTACTTCAGACTGATAATGT<br>TAGCCCCTGTGACACCATCCTGTTACCTCACCTTCAGCCAGT<br>CAGAGAATTGTGGTTGAGCTGATCACATACCCTGCAACCTCC<br>CTCCGTCACCTGGGTTTTAAATATGCTTTACCAAAATGCTTA<br>GGGGAGCTTGTGGCTTTTTAGTGGTTGAGCCACCCGTCTCCT<br>TGCACGGGCCTGAAATAAACCTTTCTCTTCTCTAAACCCCAA<br>AGTCTAGGTTTTTTTGGACTTACTGTACATTGGGCACATGAA<br>CTTGTGCTAATAAGACAAGGGTTTCCAGTGTGGTCTTCTAGG

MSTRG.65715.8	0.26	0.01	0.00811	-4.75	down	GACCGCGCGCGGTCGGGTCCGCTGGGCTGAGCATCGCCGGCG<br>GCGGCGGGGTGGATTTCCGGTGAGCGGAGACATCCAAAATTG<br>CAGGGAAATACTGGGGAGAAAAGTGCCCCCGGAGGAGCAGGG<br>TCCGTAATTGCCCGCTAATGATGACTGACATTTCTAAGGCGA<br>TGGCATGGACCACAATGAGAAATACGGTTCGGTCGCCGATTG<br>CTAAGTAGCAAAGAGCAGGCGACTGACTGAGGGCTGCTGGGC<br>GAAAGATGGATCCCTGTGAAGATGTAGAAAGGTCCAGTGGTA<br>AATCTGAGAACCCCAGGGGGAATCAGCGTCATGAACTTGAAG<br>GACCACAGGCCAGTGGTCCTGAGGCCCCCGGGAATGATGCTG<br>CTGAGCTATGGAGCCAGAAATGCGAGCTGCGGAGCAAATTGG<br>TGCAGACCAGCAACAGTGAACAGCAAGATTGAAAGAGTTTCT<br>GAAGAACTTCGAACGGTTGTCCAAGAAATGAAAAAATACTTC<br>CCATCGGAAAGACATAGTAAACCAAGCACACTGGATGCCCTT<br>AACTATGCCCTTCTCTGTGTACACAGGGTACAAGCAAACAGT<br>GAGTCTTTCCAGATTCTCAGACAGAACAGAGCACCTCAAGCT<br>GATGTGACCTTGTGCAGTCTGGAGGAGCTGGCCACTATTGCT<br>TCAGAGCACACTTGGAAAAACACATTCCAGTACCTACTACCC<br>AGGTTGTAACAACTGGATTTTATAGAGCAGGATACCTTTGTG<br>GCAGTGTTTTCATTTCTCTCTGGAAGGTTAGTGCATGTTTCT<br>GAACAGGCTCCTTCGATCCTGAATTGTAAGAATTGGAGTCTT<br>GTCATTTTGTTGAACTGCTTGCCCCCCAAGACATGAGGGTGT<br>TCTACACGCACACTGCCCATGCTCAGCTTCCATTCTGGAACA<br>ACTGGACACAAAGAGGTAATGGGACCAATATTCAGATGTTTA<br>TCTCACCTGGTTAAGCTCACTTTCATTCTACCAGAAATAGTA<br>CACAAGTGAATGCATTATTCAGCAATGCAAATTATATGGTTT
MSTRG.103945.18	0.19	0.01	0.00195	-4.82	down	AGAAATTGTTGATGTAATAATAAACATTCTGCAATGATGCTG<br>CATGGGAAATAGGCCTTACCTTGCTACTCAGGGTGTGGTCCA<br>TGAATCAATAGCATGAACATCACTTGAGAACTTTGTAGAATG<br>CACATTCTTGGATGCTACTCCAGCCCTACTGTATAACAATCT<br>GCAGTTTAACAGGATTCTAGGTAATTCATAGGAATATTATGT<br>GTAAAAAAAAATGGATTGGGGCAGTATCTTGAACCTCATTAC<br>AAATCATGCCACAGAGTATTTAATGTTCTTCAGATTCTAATA<br>GGTCCATATAAACTCATATTATTCTGGATTTAATGCCTTAGC<br>ATATCATGTTTGACAAGCTATTGAGAAAAAAGTATCAGATCT<br>CATGCCTCAGGAGAGACCTGCTGGGAAATCAGTAGTGGATGT<br>GATGCTTTTTGAGAATTTAGCAAACATTTGGGTCATACAGTG<br>TTAATAATTTAAAAGCTTTAAAGTGGTCAACATATTTTTAAA<br>GGTAAAATAATTTAAGTTGAGGGGTGAAAGGTACAGCAGTTT<br>AATTAGTTCAAAGGAAGGTAACTTGAAAATATAGAGGAAGAC<br>AAAAACAAAGTACAAACTTACTGTTGAATCAGAATTTTCAAG<br>TTGTAAGAAGACACTGATGCCCTCTGGTTCAGAGCTAATGTA<br>AGCTATGACTTGAATTTTCCAAGCCAGTGCTGACCGTCTTGT<br>TGAGGCTGCCACAGAGAACTCACCGTCCTCCCAGCAGCTTTT<br>ATATGTTGTTGGCCAGCTCTGTGTTGTCAGCTTGGTTTTGCT<br>TTCACTGAACCAACTTCTGCCTCGATGACTCTTCCAGGTAGC<br>ATTGCTGCTACTTCTAAAGCTTGGGGACACACAAGGCAAGTG<br>AAATTGCACTTTCGCTTCAGACTCTCTTCTATCTTTAATCGT<br>TAGATCCGTCCGCTATGTGTTCTATTTTTTTATGCCAACTAA<br>CTGTACACTAGGGCATCTATCTTTTCATATCTTGGTCAGTCT<br>CCTTTAGACTAACTCCTGTCTCACAACATTCTTTTTAAAATA

MSTRG.81336.1	0.35	0	4.47E-05	-5.06	down	GGGGGGCCCGATCGGAGGGGGAGCGGCTCAGCTCCGCTAGCC<br>TCTATTTATAGAGCCCGGAACCCGAAATAGCGCGGAGCCGAG<br>CGGCCGGGATGACGCGGGAGCCGCCGGGGGCTGACTCACCCG<br>GCCCGGAGCGGCGGCCCCCGGATGGGGGACAGTGGAGGCGCT<br>GGACTCGACCCCACGCCATTCCGGGCACCTCTTCTGCTGGGA<br>CGTCCCAGGTTTCCACGAGGCACTGTGGTTGGATACGCAGCA<br>AAAAGGAGGATCTGCAGCACGAGAGACTGAGGTTAGATCAGA<br>GGAGATCTTCTCAACCCAGGGATCGAACCTGCGGCTCCTGTA<br>TTAGCAGGCAGATGCTTTACCACTGAGCCATCTGGGAAGCCC<br>AACACCATGTTAGAGATGAAGAAACTGAGATTCAAGGAATAC<br>CTTACATGTCCTCAGAACTGTCTCCTGAACCAGGTACCCTGG<br>CCCCGGAGATGTTCAGTTCCCAGGACACTGTGTTTAGTTCCT<br>ATTGGACCGAAAGGGGAGCTACTAGGGCATTCAAGCCCACGT<br>CCTATTTCTTGTGGACAGCGCTGGTCCATGGGATTTGCTGTC<br>TCTCCATCTGGGAGGGCATCAGATGATGGGATGCCACTTGTT<br>CCGGGGGTCACTGAGCCTCAGGATTTCTTGTGATTGTTCCAG<br>AACAATCCATGTCAGAAGCACATTCAAACAGAGCGACTAATC<br>CTCACATCAAAAACATGGCTTTAAAGGACACTCTCAAATTCC<br>TCTCTGCACCAGGTGAAGAAGCTACTTAGCCGAGAGAAACCA<br>CAGTGCCAGGCAGTATTTTGCAGCCACTCTGTGTCTGGAGTA<br>TACTTTTTTTTTCATAAATTATATACACGCATGCCTTTATTG<br>GGCTTTCCAGGTGGCGCTAGTAATAAAGAATCCGCCTGCCGA<br>GGCAGGAGATCTAAGAGACCCAAGTTCAATCCCTGGGTCGGG<br>AAGATCCCTTGGAGGAGGGCATAGCAGCCCCCTCGAGTATTC<br>TTGCCTGGAGAATTCCATGGACAGAGAAACCTGGTGGGCTTC
MSTRG.39163.1	0.2	0	2.96E-05	-5.16	down	TTGTTGAGCCTGCGTCTAACGACATGTCAACCACTTGCAAGA<br>AACCAGGAACTACTTTGAGCAATGATTCCATCAGCACCTAAT<br>TTCAGTCAAGTTTAATTCCAGATGGAGCACCACCAACTCAGA<br>TTTGTTACAGGGATTTATAGCAATTGTAACTTAGACAATTGT<br>GGGGGCTACTAAACAGTGTCTGTTGGTCTGTCCAGCACATCT<br>GAAGCTGGAACTTGAAACCTACAAGGAAGAGAAGACAAGTGT<br>AACATGGGCAAGAACAGTACAGGCTGGAACTCATGACCAGGA<br>ATGGGACAGATGCCACCAAAACAGACTGAACCTGTGACAATT<br>CTCACTTCCTCTGACCTTGACAGGCAGGTATCCAGGAGAAGC<br>CCCTTCATGGAGCTAGACACATGACTGACCAGGAGGCATAGA<br>ATGTAAGAGCAGAAAAAGGCAGAGTCATTGCGTGTCCATTAA<br>GTGATCCATCAGATAAGGCAATGGACTTGAACTAGAAACATG<br>GTGTATGTGAGCTTCTGAGTGTGAGGAACAGTATGGTTGTCG<br>CTCACTTCTTCCAGCTTCTACACAGCATCTCTTGGGACCCAC<br>CCTCATCAGAGATATGCAGGGAAGGAAACGGGGACTTTTGTT<br>CAGCTCAGCTAAGCTGACGCATGGCAGAGTGGCCACTAGTCA<br>CCTTGTCTGACAACAGGAAGAGAACCAGCTTCAGCTTAGGAG<br>ATCTGTCCGTGTGACCTTAGGTACAACCACATAACCTCTCTG<br>TCTCGGTTTTAGGTCTTAATACTCTCATGTGTAATAGGGAAT<br>GCAGGGGTGACTTTTTCAACCCTCGAGGCCCCTTCCAGTTCT<br>AAGTCTATACTTTTGTCATCCTGTCTTTGTTCCGTTTCTTGT<br>GTCTGTCAGTCTTGCTTCCACATTTACTGCAATTCTGTGAGC<br>TCAGCCAGAGGGAAAGCCAGATAAGAACAATGGAAGGAGCCT<br>GTGTGTTAGTCTAAGCTCTCAGAGCCGCTGAGGTAAATGGTG<br>GGGTCAGAGGGCAATTGTCAGTGCTGGAATTAAATATTTACT

MSTRG.82259.81	0.4	0	1.37E-05	-5.49	down	GCCCTTTCTTAGTCCTGACCATTCCTCACATAGTACCTTGAT<br>ATGGATGGAAACACTGTTCTCACCTACCACATCCCTCCACCT<br>TTTACATACTCTCACTGTAAAGATCTCTGGCCATTTATTGAA<br>CAAGTTTCTCAGGACATGACCTAAATCACTCTTTGCATCCAT<br>GATGACTCACAACCCATTTCGAGATTGGATGGTTGAATAATA<br>GTTAAATGGACTACTTCTGTCTTGTATCTTCCTTGATATTTT<br>CTTTCTAAGTTCCTTGTATTTTTTTTTAAATGTTTTCTAATC<br>TATTCATTCGGTGGGTGGTCATGTTTACAAATAAGTTTTCTC<br>AGTCTGTAGGTTCAGATTCATAACCTGCATCTCTTACCACTT<br>TGTGCGCCATTTGGTGTGTCTCCTCCGCTGATTAGAGAAAAA<br>CATGTTAATTTACCTCAAGCCTTTTAAAGCAGATTAGTGCTG<br>TCAGTTGGCTTGCAGTAGTGCATGTTTTATAGTAAATACTCA<br>AAATTTTCTTCATGTTCTTTGTCATATGACTTTATCTACATA<br>TCTTTTTAAAATTTTATTAATTTTTTAATTGAAGGATTATTG<br>CTTTACAGAATTTTGTTTTCTGTCAGACCTCAACTACATTAT<br>ATCTTGTATGCTATTTACTTGCAACATTTTTCAGCAATATTG<br>TGTCTGACCCTAGTCAATAAATACACAAAGCACCTGCTTTTT<br>ATTTATTATATTTTGGTGCCATTAAACTAGGTAATTAAGAAT<br>AGTGCATGGCTAGAGAGAAGCCTTCCTTGGTGGCTCAGTGGT<br>AAAGAATTCACCTACCAATGCAGAAGATGCAGGTTTGATCCC<br>TGAGTTAGGAAGATCCCGTGGAGAATGAAATGGCAACCCACT<br>CCATTATTCTTGCCTGGGAATTCCCATGGACAGAGGAGTCCT<br>GTGGGCTACAGTCCACGGGGTCGCAGAGTCAAATACGACTGA<br>GTGACTGAACAACTGAACAACAACATAGGTAGGGATTGCTTT<br>GGTTATTGACTGTTAGAAAACTGGTGAATATTTTATGTCTTA
MSTRG.225063.2	0.13	0	9.47E-06	-5.51	down	CGCGTCTTCCTGGTCAACGAGCCTGCGCATGAAGGAGGCGTG<br>CCCGGCGGCCCCAGCAGAGGCCTGAGGAGGGCGGCGATGGTC<br>CCCGGCCCCACTCTCGAGGGGCTCTCGGCGGGGCCTGGGTTG<br>GCGCCGTTCACGCAGAGCCCGCTCCTCGGGGACCGCCCTTGG<br>CCGTTTCTCCCGCGCCTGCCGCCGCGCCTCTGATCGGGCCAT<br>CCCCCTACGATCGCACACGCATGGCGCCCAGCCTCCTATCTC<br>TTGTTACGTGCCAGGGCCGGGCCGGGGGGCTTCCCCGTTGTC<br>TCCGAGCACCGCCACCTCTCCCGCCCCAGGGTCCGCCTGCGT<br>AGCTGGTCTGCCTTTTTCTGCTTTCCTTCCCTGCTTACCGCT<br>CTGCTGCTAGTCCTTCAGGGGGCGGTCCTTGGGCGTGGAGGG<br>CCTCCCCACAGACATCTCCTTTTTGGTAAAGGGCCACTCCAG<br>TACTCTTGCCTGGAAAATCCCATGGACAGAGGAGCCTGGTAG<br>GCTGCAGTCCATGGGGTCGCTAAGAGTCGGGCACGACTGAGC<br>GACTTCCCTTTCACTTTTCACCTTCATGCATTGGAGAAGGAA<br>ATGGCAACCCACTCCAGTGTTGTTGCCTGGAGAATCCCAGGG<br>ACAGAGGAGCCTAGTGGGCTGCTGTCTATGGGGTCACACAGA<br>GTCGGACAAGACTGAAGCGACTTAGGAGCAGCAGCAGGGCGT<br>CTTCTTGAGTCTTTTAGGCCCTCCTCTTGCCCTAAAAATGAA<br>GCGTCAAGGGACGGGTCTGTCCTTAGAAACAGCAAATGCACT<br>GGTTTTAGTGTTGGCGATGTGAAAGTACTGGGTTTGTGTAAG<br>TGAAGCTTATGCTTCCCTGAGTGAACTGTGCTGTTGGAGAAG<br>ACTCTTGAGAGTCCCTTGGATTGCAGGGAGATCCAGCCAGTC<br>CATCCTAAAGGAAATCAGTCCTGAATATTCATTGGAAGGACT<br>GATGCTGAAGCTGAAACTCCAATACTTTGGCTACCTGATGCA<br>AAGAACTGACTTATTTGAAAAGACCCTGATGCTGGGAAAGAT

MSTRG.198735.7	1.92	0.04	0.00573	-5.59	down	CCCGCTTTGGGCTGCATTCCCAAGCAACCCGTCTCCGGGAAGACCCGGGCCCGGCGCGCCGGGGGCCGCCATCGGCCTCACACCGTCCACGGGCTGGGCCTCGATCAGAAGGACTTGGGCCCCCGGTATTTAGCCTTAGATGGAGTTTACCACCCGCTTTGGGCTGCATTCCCAAGCAACCCGACTCCGGGAAGACCCGGGCCCGGCGCGCCGGGGGCCGCTACCGGCCTCACACCGTCCACGGGCTGGGCCTCGATCAGAAGGACTTGGGCCCCCCACGAGCGGCGCCGGGGAGTGGGTCTTCCAAGCAACCCGACTCCGGGAAGACCCGGGCCCGGCGCGCCGGGGGCCGCTACCGGCCTCACACCGTCCACGGGCTGGGCCTCGATCAGAAGGACTTGGGCCCCCCACGAGCGGCGCCGGGGAGTGGGTCTTCCGTACGCCACATG
MSTRG.138309.1	1.14	0.02	0.0019	-5.84	down	ACATCTCCAGACTTTGCCATGTGTCTCTTGAGGGCAAAATCACCCCTGGTTGAAAACTGATCTATAAAACAGGTATAGGGGCTTCCCTAGTGGTCTAGTGGTTAAGAATTTGCCTGTCAATGCAGTGGGGAAGGGAGAAGGCAATGGCACCCCACTCCAGTACTCTTGCCTGGAAAATCCCATGGACGGAGGAGCCTGGTAGGCTGCAGTCCATGGGGTCGAGAAGAGTCGGACACGACTAAGTGACTTCACTTTCGCTTTTCACTTTCATGCATTGGAGAAGGAAATGGCAACCCACTCCAGTGTTCTTGCCTGGAGAATCCCAGGGACGGGGGAGCCTGGTGGGCTGCCGTCTCTGGGGTCGCACAGAGTCGGACACGACTGAAGCGACTTAGCAGCAGCAGCAACAGCACCAGCAGTGGGGAAGATTCCACATTCCATGCGGCAACTAAGCCCGTGCGCCACGACTACTGAAGCCCATGTGCCTAGAGCCTGTGCTCCACAACAGGAGAAGCGACCACAGTGAGAAACCTGTGCACATCAGCAAATACCCAGTGCAGCCGCTCAGCTCAAATGGCCCCTCTTGCGTAAAGCTTACCCCTCCCACTGAGATGACCTGGTCACACTCTTCTCTAAGTGCCCAGAAGCTCTGTACCTGCTACATCCTGCTCACAAGGAAGGAAGGACTAACTGCTGCTACCTGTCTGCTCTCCTTACCCGATGGGGGCTTCTGGAGGCAAGGACCCCTGGTGCGCATCCCAGTGCCTGGCACGTCATGGGACTTTGCACATGGCTGCTGGGTGAGGGAAGCTCCATGGCAGCAGGATGAGGTGCTGGGAACAAGCAGGTGAGTGGAAACAAGGGCTCTATGGAGAAGGAGCTGGGACATCGTGAGGTTAGGAGCCATGGGCTCTCCTCAGGATGACTTCTTCATCATCATCACAGTGGTCCCTTCAGGCTGGAACAAAATACTCAGTGCATCAGAGACTTGAGTTCGACTCAGACAAAATTTTGTCTAATTTCTAAAAAAGAATACTAAAAGAAGATGGC

MSTRG.231934.10	0.19	0	0.00125	-6.05	down	TCTGGATATCAGCGTTGCGGCTGGAGGCAGGGCTGGTCCTCA<br>GCCTGCTGTCCACTGCCACCTTGGCCTGCTCTTGCGCCGCGA<br>ACTCTGTGCCGTCGTTCCCTTTCACCATATTTTGGGACGGCC<br>GGCGCTAAGAATTTATGCTATGACCTTCGATTCAGCCAGGAG<br>GTGACACCAAACTTGATCAGAAACCATGCCACAGGTGAAGAG<br>GTATATTGAGCCCTGCCTGGCCAGTGAGGTTACTAGAGAATT<br>GGAAGAGGCTCAAGTCAGCATCCTGCACATGGTTATCAAACC<br>ATTGTGAAGTCTTAGTAAGTGTCACCTGGGATACTTGTTAAA<br>AATGCAGATTCTTGGGCAGCTAGAAACCTTGTCACTGGTCAA<br>GAGCAACAAGTAACAAGACCTACTTGTCACTTATCCTGAGGC<br>ATCGTGCCCAGTGGGTTTACCAGCAAACCAGAATAAAGCATC<br>ATTGCCCTGGATGTCCTTGTGAGATGGTTGGGAAGTCTTGGC<br>TGAACTGTGGAGAATATCTTTGAAAAGTGTTTTAGAGAGTCT<br>ACCAGATTGGCCATGTGAGTGCCTGTGCAGCAGAAGAGTGGG<br>TCTTCTCAGTGGTTAAGGTAACAAAGTGATTAAGGTAAGTGA<br>AGGAGGAAAGATTGACATTGTCTTGACTCCTATTAGGGAGGC<br>TTGTAGGAATGTCATTTCTAGGGGCCAGTAATTTTAAAGCCT<br>GTAGTTTATTCTACAATATATCCTTCTCAAAACAGTTGGGGT<br>AGGTTATATGCTTACCCAGTCTGAGATCACCCCTGCAGAGGA<br>TCAGAAGAATTGAAATGGTTCCTAAGTAAACTAGGTGGCATA<br>AGCTGCATGTCATAAATAATCTCAGCATGCACTGATTACAGT<br>CCCTTTGGTGAGAGTCTATTCTTTCAAACCAGACTTATTGAG<br>AGTTTTTCTTTATCAGCCTATGGAGCTGCATACATCTTGTCA<br>AAAGCCAGCTCATTAGCAGAAGCTTATTATTTAAGCAGCAGA<br>CAAGAATATAACACTTTGAATTAGTAATTTCAGAGGTAGCAG
MSTRG.142554.5	0.14	0	0.0014	-6.15	down	GAGTCCACCTGCGTCCGGCCGCGGTGCGCCCGAGCGCGCGCG<br>GCGAAGCGACGGGGGCTCCCGGGAGCCGTGGCGCCGGCCCGG<br>CTGCGGGGAGGGCGTGTGCCTTGGCCGCCCCCGCCCCGGGTC<br>GAGATTCAGTTTGGAAACGCGAGGATTTCCCGCCCCCCCCCC<br>CCCCAATTCGTGATGTGGGCTTAGGGTTACTTTTCGTATTGA<br>CTTAGGAAAGCACGTGCTCTCCAAACTGGCTTTTGCGCTTTC<br>CCTGTCATCGTCAGTGATTCCCTGTCATCGTAAATGATGCGT<br>GGCCGAAGCGACTGAAATGTTTTAAAAGCCGGATGCGCCCAG<br>GATGATGTAGTCATGTCAGTTTAAGTGGCTTTGGGGTATAAT<br>TTTGTGTGTGTGTAAGGACTTGCAAAAAAAATTCCGGCAAGA<br>GACATGTAATGAATTTATGTAGGATTTCAGCGTCAATATTGA<br>AGTAATCAGATTTCAGTGGTTGGCGCAGCCTCGCAAGTAAAT<br>GGCCCCTTATCTACGAGGTTGAGGTCTTAACTGTTCATTTTG<br>AGCGAGTTCTCTAGGATCAGCTGACTGCTGATTATAATTCAC<br>ACTAGACGTAGAGGTTGTGTCCTTAGTTGATAAGCAGAGATT<br>AGAGCTAACATTTAACATTCTACCCCGTAGCAAGTTTTCTCT<br>GCTTCTTTTCGGATCATTCTGTTCAAATTAAACTCAGGTCTG<br>CCTAGATGAATAGCATTGCTTGGTGCATAACATAATAGCTTT<br>ATTTCTAGTAGTAACACGTATTAGCTTATATACATTATGTAT<br>ATATAATCGTGCTCTACAACTGTTTAGTATGATTCCAAAGTT<br>GGAAAATTAGGAACAGTAATACTGGGCAATGTTGAGTAGTGA<br>TTGTTTGCCAGACTTGTTTCAAATCATCTACACGCTTTATCT<br>CATTTAATCTTTGGGATAACTTTTTGCATAAACATAGGTTAC<br>TCTTAAATACTTGAGTACACGAGTTGGATTTGAATGTGTCCA<br>AATGGTGAATATGTACATTAATTGTTTCTGTTTCTGTAAAAC

MSTRG.133391.3	15.5	0.16	0.00217	-6.59	down	CGACGTCGGGGCGTTGGCCCGTGGGGCGTGCCGTGCCCCTCG<br>CCCGCCTCACCCTTTCCCAGGTACCTAGCGCGTCCCGGCGCG<br>GAGGTTTAAAGACCCCTGGGGGGGGGTGTCGCCCGTCCGCC<br>TTGGGGTTCGGGGCGGTCGGGCCCGCGGGGAGTAGTCGGGAG<br>CTGCCTCCCGTCTCCCCCCAGACCCCGCCGTCCCCCGAGGGG<br>ACGGGGTGGCGCGCGGCGTGGCGCGCCACGGTCACTGCCGCC<br>GCGGCCGTCGGGAGGGGGCTACCCGACGGCCGTCGTGTGGGC<br>CGTGGCCGTGTGCGGCGGCGCGTGCGCGCGCTGCCCCGCGTC<br>CCTGGGGGAGGGTTGGGGGCCCCCCGGGCGCCTGTGGGGTTG<br>TCCGAGCCCGCCCTGGCGGCTTGGCGCCGGACGCCCCGTCGT<br>GTGGAACCTTTCCCGACCCCTCCGTTCTGCTGTTTTCTGTCT<br>GACTTGGCCGGCCAGTAGTCGGGAGCTGCCTCCCGTCTCCCC<br>CCAGACCCCGCCGTCCCCCGAGGGGACGGGGTGGCGCGCGGC<br>GTGGCGCGCCACGGTCACTGCCGCCGCGGCCGTCGGGAGGGG<br>GCTACCCGACGGCCGTCGTGTGGGCCGTGGCCGTGTGCGGCG<br>GCGCGTGCGCGCGCTGCCCCGCGTCCCTGGGGGAGGGTTGGG<br>GGCCCCCCGGGCGCCTGTGGGGTTGTCCGAGCCCGCCCTGGC<br>GGCTTGGCGCCGGACGCCCCGTCGTGTGGAACCTTTCCCGAC<br>CCCTCCGTTCTGCTGTTTTCTGTCTGACTTGGCCGGCCAGAG<br>GCAACCCCCTGCCCCAACTCCCACCTCCGGGGAGGGAGGCGG
MSTRG.180512.2	0.39	0	3.04E-07	-7.24	down	TGCTGTTCCTGACCTTTTGCTGAGTTTATTACTGAGCAAACT<br>GTGGCTTCATGGTGGAGGGGGATCCTCGTTGCTGCACTGGCT<br>CCTCTTTAGTTGCGGTGCAGGGGCTTCTCATTGTGATGGAGC<br>AGAGGCTCTAGGGCATGCAGGCTTCAGTAATTGTGGCCCTTG<br>CGCTCTAGAACACAGGCTCAGTCTTCGTGGCCCAGGGGCTTT<br>GCTTCTCCTCAGCATGTGGGGTCTTCCCAGACCAGGGATCAA<br>ACCCTTGTCTCCTGCATTGGTGGGTGGATTCTTATCCACCCA<br>GCCACCAGGGAAGCGCCGATGCCAACTGTCTTGAAAGCATTG<br>ATCCTGGATCCCAGACTGAAGCAGGTGATGCTGAAACTCCTG<br>AAGGAATCGTGTCATCTTGAGCTGCTCAAGTTTATTTCCACT<br>AAAAATACACCCTGAAACTGGGGCACAATGAAGGATTTCTGA<br>CAAATGGGGAAGAGGAATAAATAAAAACTCTGGGGCCTTCTG<br>ATTCTTCACTGAGTCACCTCCAAATACTCACCAGTGAGCTGA<br>GCTTGGACCCTCTCCACTCGTCACTGTCATCAGTAAAAGCTC<br>AGGCAGTCCGTGTGGTCTGGGAGCTTAAAAGTCTCGAAGCTT<br>GTGAGTCATTGTTAGGAGAGGCCCCTGATGGAACTGAACCCT<br>GCATCTACCAGGGAGGAAACAGAAGAACTAGAGGGGAAGTGA<br>CTTGGTCCTGCACCATTCGGATGAGTGTGCTGGAGTGGGACT<br>TAGAGCCTGCACCGGCCTCCTCATTTCACCACCCCCACTCCC<br>CAGTCTTCCCAAGCCCCCCGCCTGCACCCAGCACCTGTGTCT<br>GCCGTTCCTGTGGCACATCTTAAGGCAGCCTAGGCATGCCAG<br>GGGGCGGTCAATGAGAGAATCCTCAGTTAGCAATAAAGAATG<br>CTTTCAGCTTCAGGTTACAGAAAACCAGATCAATGGCAGCTC<br>AACTGTAAGGACATTAGTGTTGACTCATTGAGACTAGGGAGG<br>GAGGTAGCCCCAAGCTGAGACCACTGACTCAGCCATGTCAAG

MSTRG.183083.10	0.23	0	8.02E-07	-8.57	down	CTGCGGGAGAGTAGTAAACTCGGGGCCGGAGGCCCTGGTGAGAAATGAGGACACCCGGGAGCCAGGGACGTTAAAGCTGCCGCAAGGATATAACAGGAGGACCTGGGGAGTCGGCTGGATGGGGTACTGGAGCCGGAGGCAGGAACAGCTTGCGGCTGCAGGTTCCCCCGCTTCCCCTGCTTTCAAAGGCTGAGAAGCCGGAAGGCTGAGGAACTTCTCGAGGCTCTTCGCAGCTCCAGCTCACAGATGCCGCGGATGAGTTCTGCCTCAGTATCATCAGCTGTGGCCCCACCCAGCCCCATCTCCTCCTCTAAGGTGGACTCAGACTTTGAATTCTGATGGGAAAGCAGCCAGGGATCATGGGTGAGCAGACAGTTTTGATTCCAGCCTACCCTTCATTTCACATGAATGGATCTGGGGAAGGGCAAATTCTGCAAAGCTCTTCACAGGCTTGTCACGTGTATCTGAAGAGCAAAGCCTCATGAATTAAATAGGAAAAGTACTAAATCCCCTCCATTTGTGTCTAAAGTGGCCAAAGCATCCCACATCTCTTCCGATTCATCCTCATAGCAGCATATAACTTCCTGAACAAAATAAATCGATGGAACCTGCCAGACAACGGCTCAAGGTCACAGAGAAGGACTCGGCGCCGACAGAGCTCTCCGCTCACAGCCTGCTCCAGGTGCACCAGCTGCTGCAGAGCCACGTCCCCGGCCAGGGACAGCAGGTTCATCAACAGGAAGGGCCTGCTTCGCACAGTCCTGCAGTATCCGGGCGCAGATCACCTCGGGGCCCTCAGCCAGCTGGTAGATGAGGGCCACAGCCGCCTCCTTGAACGGGACCCAGAGCGGGTCTGGGTGGATGCTGCCTGCACAGGAAGAGGGGGAAGCTGCCACCACAGACAGAGGAGCCCTTCTGCCCCAGGCTCTGCGCGGCAGCTCACCTTTCGTGACCATCTCACGCAGCCGCTCAAACAGGCGGTGCTCCTGGGGCAGCCGGAAGGGAGGGTGACGTTCGCCCATAGAAGGCTGTTAAGGGCAGGGAAGACAA
MSTRG.43629.32	1.23	0	2.45E-07	-8.61	down	TGGCAGTGTGCACACCCCCAGCCCCTAAGCACTCTGCAAGGACGCCTGGCTCCTAGGTCCGAAGGGAAGGCGAGAGCTGAGCTGAGGCACCAGTCAGGTCTCCCACACTCCCGCAGCCTGCTCCTTCCCGGGCGCGAACGCTAGAAGCTGGGCAGGGCTTGGGGAGGCTCGCCCAGCACAGAGCAGCCTCTCGGCCGGGGCTCTGTGCTATCGCGCCTGGGCGCAGAGCTGGAGCTCCGCCGGTGTGGAAGCTCACTCGGTCCATCCCAGTACAGGCTTGCAAGCACCCGCCGGCAGCCAGCCTCTCTCTCCCGCGTTCCGGGCATTGGGCTCCAAACCTGGGACCAAAGTACTGCCCGGAGCTCACTGATCA

MSTRG.234381.1	2.16	0	2.30E-06	-9.26	down	TTTTCAGTGAGTCAGCTCTTTGCATCAGGTGGCCAAAGTATTGGAGTTTCAGCTTCAGCATCAGTCCTTACCAATGAATATTCAGGACTGTAAAGAACAACCTTACTTAATCATTATCAGTATATGAAAGTTCTGGATATTGTCGCCAATGCTTAATATTGTCAGTGTTTAATTTTAGCCATCTGGCTGTTGTTCTGTAGTACCTTACTGTGGTTTTTATTTCCATTTTTTCTAATGAGTTGGAATCCTTTTTATATGTTTTTTGGTCATGTGGATGTTAGCTTTTGTAAGGAGCCTGTTCTAATCTCATACTTATTTTTTCTTTTTTATTGATTTGTATTTCTTTATTTTGTGTCTGGAAATGACCATTTTTACATGTTCATTATATGTATTAACATTTCAGGATATATATTTTATACAAGCCTAGAATTTGAAGGCCAGAGACCCGTAAAGAATATATCGTCCAGTCTCTTATTAATAGCTTAATGTGAAACTGAGACCTATAGAGATAGTCATTTGCTCAAACCCTCTTCTAGTTAGTAGAAAGATCAGAAATAAAACCAGAAATAGTCTCTGAATCCTGACAGTAATGACCTTCCCTATTAAAACATGGAGAGTCTTTAGTCCTTTCTAATATGGTAACTGAGACTCTGTCTGTCTGGGAAGGCCAAGCAATGGAATGCATGACATAGTTATTGTTAATAGCAAATACAGTGTGATATATGATTAATCTCATTGATTCTCTGGACAAAAATATCCTCAACTAATCAACTGTTTTATAGCATGATGATACAAAATTTCAACGTGTATTTTTCTTTTGGTCTTAGCTTTATTGTTTTTTCTTTTCCATAGCTTTATTGTTGCATTCAACTTTTCATATTTATTCCATTACTCCTATCTTCTCATTCAACATTTGGTTCAAATTCAAGATAATCCATTTTTTTCTATAGCAGATTAGTAAGAATGTTAACTTGTTACTATTGTCATGAAATAGAGCAAAGTTAGGTTACCAGACATCCTTGCTAATAGAATAACGATGCCTACTATATA
MSTRG.36282.7	0.19	0	5.34E-06	-9.32	down	GTAGCAGTAGCAGCTTTTTGCTTTAGTTAGCATCCCTAAAATCTGCACCTGGTAAAAGGATATTCTTTTAAATGGCTCCTTGTCGATTACATTTAAGCTAATTAAAATATTTCTTTCTTCCCCCTGTTTTTATTATTGCTTGTGTCAAGTGCCCTTCTTCTTAAAGCAAAACAATTTGTAAATTCTGGAAATGAGTTATATATCTTTGTTACATTTTCACTTAGATGCTCTTTGAAAGAACAGATGTGGCGGCAAGTTACTTTCTTCAGATTCTGTTACTGGTTCAAACATCATGGTTCTTAATTTAGAAAGTTGCATCTGATGATCAAAGAAAAGTCAGAAATCTTGTAGGTTTTTGATGGGGATTGTGTGTTTCTCATTGTCCTTATTTTAAAATTCTTGATCTTTATTTCTCAATTTCCTTGTATCTAACTGGGTTAATGTCTTTTAAATGATTGTAGAGAACAATCCTAGAGATAAGAGAAAGAAAAAGCTATTACTGAATGCCTCTGAGTGTGTGTGTGCTCAGTCATGTCTGATTCTTTGCGATCCCATGGACTGTAGTCCCCCAGGCTCCTCTGTCCATGGAATTTTCCAGGCGAGAATATTGGAGTGGGTTGCCATTTCCTTCTCTAGGGGATCTTTCCTACCCAGGGATGGAACCCGCATCTCTAACATCTCCTGCATTGGCAGGCAGATTCTTTACCACTAAGCCTCCTGGGAAGCCACTGGCTGCCTAGTTTTAGGCAGATTCTATGAAAACTCATACGGTTTCATTTAATTTGCCCTATGCTCCCTTTTTTAATGCACAAATAAAGAAAAGGAAGCCCAGAGAGGTTTAGTTTGTCTCCAGAGGTCCTGCAGCTGGTAATTTGCAGAGCTCATTTTGAAATCAAAGTTTATCTGACTATAAACCCAGATTACTTTATGAAGACAGCTCCTGTTTTTATGCTGTGGCTTTAAAATCTAAACCGTAGCTATCTAAAATTAACACAGTTTTTTTGAGCCTTGAATCCCAATAAGAATACAACTTTTATGAAATAATTTCTG

MSTRG.148408.2	0.33	0	4.58E-11	-11.8	down	GGTCGGACGCGCCCGCAGCGGGCGAAGAGAGCGAAGTGGACA<br>AGGCCGCCATGTTAGGGGTGCTGGAAAGATTCCGCGGAGCCC<br>GCTGAGGTTTTAGGGAACTCGGGAGCCGAGTGGTGGTGCAGA<br>GGGGACAGTGAGGAGGAGGATAAGTCGGTTGAATCTGTCTTC<br>ACTATGAAAAGATGAGAAATTTTCTTTCTTCCTGAGGGTGTC<br>GGAAGGTCTTAAAAGGCTGATATATTAGTGCTGTTTTTCTCC<br>TTTCCTTTTGCATATTATCCTGAAAACATAACATCACTTTGG<br>GTCAGTAGGGGTATGCACTTCACTTGTTAGGAGTTAAAATTA<br>TAGCTGATAAGTGGATTACTGTATCAAGGAAAGACTAGGCAG<br>AGTGATTATAAATGACAAAAACATGGTCAAAACATTTCTCAG<br>ATGTATGCAGATGATAATTACAGAAATAGTTTTATAAAGAAT<br>GATCTTACATATTGTCATGTTTCTAGGAACAAGTCAGATGAA<br>TGGCTTTCATTTTTAAACAGAGGTAGTATGTGTTGGTCTCAG<br>TTATGCATGGAGCTTATGGAATTATTTGTTACTCAGAAATAC<br>CTTCTTTACAAGTAATGTTGGTAAGTATAGGAAAAGGAACAA<br>AATGTGTACTGTTTTCTTTGTGATCTGTGTTTCTTCGTATAA<br>CTATAGACATGTTTTAACCTTAAGAGAGACAAAATAGACTGA<br>ACAGATTTACATTTAGGCTTTCTTTCAAACTCATACAAATTA<br>TTTTTAGGGGTTGGTGATTCATGGGTGTTGTTGTGTCTCATA<br>TGAACTTATCTTGTTCATTTTGTAAGAGACTCCTTTATATAA<br>GCAATAACCTCCTCAATTTGAGTTACTATAAAAAAAAAATTT<br>GTTGGGGGCGGGGGAACCCAGCAGATTTCTCAAAAACATTGT<br>ATGAGAGAAATGAGACCTGTATAATATAGTGATTTCTGTAAT<br>TTGCCATGTGTTTGGGTAGTTAGAAATTTTTTTCAGACTTTT<br>ATCTAATGGGCATCAAGCCATAAAGAAATGAAATCTAGTTAC
MSTRG.12156.2	0.97	0	6.81E-11	-11.8	down	TCCCCCCCGAAACCCCGGCTCCAGAAGAGCGCGCGGCGGAGG<br>GCACCGGCCGGTTACTTCCTCCGGCGGCGGCGCGGAGCGCGG<br>TGCAGCCACAGCCCGGATCGCCCCGATCGTCGCGAGCCGGCC<br>CGCCGTCCCCGGCCCGCCCAGGCCTCCCGGATCTCCCGGGGA<br>GCGCTCTGCGGCGCCCGCTTCTCTCCCGGGGAACCCAGGAAC<br>TGAGTTGGGACGCAGGTGGCGCCCCGGATCCCCAGGGCGCAG<br>CCGAGGTCCAGGCAGGGTGGCGACTATTCGTGGGATTCTGGG<br>GTCGCGCGTCCAGGATCAGTCCGCTTGGGGTTCTTATTCCCC<br>GTGTCGAGTGGCTTTGGGGGTAGGTGGGTGGGCGGCGAGCAG<br>GGTTCCGGGTGGGCCCGGCCGTGACGCGCGGAGCCGGCTCCG<br>AACTTGGGCCGCGGGGCCTGGGGTCGCCGAGGCGGCTGGGGC<br>GCGCAAGGCGCGCCGGCTCGTTTCACTTGCTTTTGTTTTAAA<br>AGGAAACGCAGGCGTGAAGGCGGGGGTGGCTCCTGCCGGTGG<br>GCGCCCGGCGAACCTGATTTCGCCTGGGGGCTTGGGGGACGC<br>GCGAGAAGGTGTCTGAGGGCCCCTGGCGCGCCCTGGTTTGCC<br>GGTGACTTACCGGAGGGCGCCGGGCCCGGCAAGATCCGGGGC<br>GCCTGAAACACCTGGGGAACTGCGGCTCCCACCCCCGCTCCC<br>CGTCACCTGCGCTGTCGCGTCCGGGGCCCGGCCAGGGCGGCA<br>GGCAACGGGGGCGCGGGGAGGATCCGGCGGCTCTACCGAGAC<br>CCCCGGGGCTCCCCAGGACCTCTGCATTCGGGCCCCTTGATG<br>CTGGGAGGTGAATGTGGGGACAGTGTCGGAAATGGGGTTATG<br>GGTGCGCAAGACTGCCTCTAATTTCGGGATCTTACAGCATTT<br>TGGACGAAGGGGTAACAGCGTTTGTGCGTATATGTTTTTTTT<br>TTTTTTCCAAGCACTCGAGACTTTTTTATTGGCCTGTATTCA<br>CTTGTACGGTATCCTAAAATAGTATTTGACCTGCAGTTTATT

MSTRG.16757.11	####	12.6	2.42E-07	-12.5	down	TTTTCATCGCCGGGCGCGGTGGCGCGCGCCTGTAGTCCCAGCTACTCGGGAGGCTGAGGCTGGAGGATCGCTTGAGCTCAGGAGTTCTGGGCTGCAGTGCGCTATGCCGATCGGGTGTCCGCACTAAGTTCGGCATCAATATGGTGACCTCCCGGGAGCGGGGGACCACCAGGTTGCCTAAGGAGGGGTGAACCGGCCCAGGTCGGAAACGGAGCAGGTCAAAACTCCCGTGCTGATCAGTAGTGGGATCGCGCCTGTGAATAGCCACTGTACTCCAGCCTGGGCAACATAGCGAGACCCCGTCTCTTTTGAGTT

Table S2. Significantly enriched GO terms.

#GO_classify1	GO_classify2	All gene	DE gene
#Total_gene		21276	157
cellular component	extracellular region	4233	23
cellular component	cell	17944	144
cellular component	nucleoid	41	1
cellular component	membrane	10248	62
cellular component	virion	12	0
cellular component	cell junction	1440	10
cellular component	membrane-enclosed lumen	2698	38
cellular component	macromolecular complex	5663	61
cellular component	organelle	13348	126
cellular component	other organism	6	0
cellular component	other organism part	6	0
cellular component	extracellular region part	3171	19
cellular component	organelle part	8111	81
cellular component	virion part	9	0
cellular component	membrane part	7250	37
cellular component	synapse part	383	2
cellular component	cell part	17944	144
cellular component	synapse	558	3
cellular component	supramolecular complex	760	8
molecular function	transcription factor activity, protein binding	414	4
molecular function	nucleic acid binding transcription factor activity	916	13
molecular function	catalytic activity	8106	56
molecular function	signal transducer activity	2564	8
molecular function	structural molecule activity	616	8
molecular function	transporter activity	1691	6
molecular function	binding	14457	128
molecular function	electron carrier activity	154	1
molecular function	morphogen activity	4	0
molecular function	antioxidant activity	71	1
molecular function	metallochaperone activity	5	0
molecular function	protein tag	3	0
molecular function	chemoattractant activity	42	0
molecular function	translation regulator activity	25	0
molecular function	chemorepellent activity	5	0
molecular function	molecular transducer activity	2504	5
molecular function	molecular function regulator	980	9
biological process	reproduction	1341	11
biological process	cell killing	48	0
biological process	immune system process	2261	25
biological process	behavior	572	5
biological process	metabolic process	11762	88
biological process	cellular process	17219	129
biological process	reproductive process	1275	11
biological process	biological adhesion	1570	5
biological process	signaling	6550	37
biological process	multicellular organismal process	10758	80
biological process	developmental process	8186	80
biological process	growth	392	8

biological process	locomotion	1382	10
biological process	single-organism process	15641	121
biological process	biological phase	34	0
biological process	rhythmic process	166	0
biological process	response to stimulus	10538	82
biological process	localization	6912	45
biological process	multi-organism process	1682	16
biological process	biological regulation	14451	109
biological process	cellular component organization or biogenesis	6170	56
biological process	cell aggregation	20	1
biological process	detoxification	84	1
biological process	presynaptic process involved in chemical synaptic tran	53	2

Table S3. Significantly enriched KEGG.

#KEGG_n		KEGG_N
Spliceosome	ko03040	4
SNARE interactions in vesicular transport	ko04130	2
Ribosome biogenesis in eukaryotes	ko03008	3
Ras signaling pathway	ko04014	4
RIG-I-like receptor signaling pathway	ko04622	3
Primary bile acid biosynthesis	ko00120	1
One carbon pool by folate	ko00670	1
Nucleotide excision repair	ko03420	2
Measles	ko05162	4
Longevity regulating pathway - multiple species	ko04213	2
Legionellosis	ko05134	3
Influenza A	ko05164	3
Hypertrophic cardiomyopathy (HCM)	ko05410	2
Epstein-Barr virus infection	ko05169	5
Endocytosis	ko04144	4
DNA replication	ko03030	2
Complement and coagulation cascades	ko04610	2
Arrhythmogenic right ventricular cardiomyopathy (ARVC)	ko05412	2
Antigen processing and presentation	ko04612	3
Aminoacyl-tRNA biosynthesis	ko00970	2

Table S4. LncRNA-miRNA co-expression network.

gene_name	gene_log2FC	gene_regulated	lncRNA_name	lncRNA_log2FC	lncRNA_regulated
IGF1R	0.481573148	up	MSTRG.128763.1	-0.782306327	down
			MSTRG.13578.1	-1.407501398	down
			MSTRG.137849.6	-1.176385269	down
			MSTRG.100266.1	-1.235290401	down
			MSTRG.103991.1	-1.089861162	down
			MSTRG.12482.1	-0.66492613	down
			MSTRG.120825.1	-0.334559953	down
			MSTRG.117221.1	-0.932416378	down
			MSTRG.108092.1	1.182532533	up
IRS1	-0.463087677	down	MSTRG.108579.2	-0.285494922	down
			MSTRG.133330.1	0.11465383	up
			MSTRG.137860.1	0.770134252	up
			MSTRG.106118.1	1.115603831	up
			MSTRG.113577.13	-0.498159706	down
			MSTRG.117494.6	0.926473667	up
			MSTRG.12205.2	0.375688612	up
			MSTRG.122434.1	0.328846168	up
			MSTRG.125445.3	-0.323133452	down
			MSTRG.133282.1	1.026545002	up
			MSTRG.133279.1	0.480801455	up
			MSTRG.131525.1	0.205516541	up
			MSTRG.128161.5	0.354207201	up
AKT1	0.130986577	up	MSTRG.102914.4	-0.083138298	down
			MSTRG.103314.1	-0.60974236	down
			MSTRG.104185.1	-0.00725758	down
			MSTRG.105346.1	1.048259926	up
			MSTRG.107702.19	-0.779822599	down
			MSTRG.111347.19	1.260568359	up
			MSTRG.113751.1	-0.429326947	down
			MSTRG.120618.45	-2.172799702	down
			MSTRG.12205.1	1.227420304	up
PIK3R1	0.14623156	up	MSTRG.117686.3	-0.811084015	down
			MSTRG.119102.2	-1.095434619	down
			MSTRG.134401.1	0.314609974	up
			MSTRG.103177.1	0.270976279	up
MTOR	0.370152329	up	MSTRG.120824.1	0.972039436	up
			MSTRG.123964.1	-1.016894197	down
			MSTRG.128131.1	1.514764023	up
			MSTRG.133381.13	3.398279559	up
			MSTRG.13401.1	-1.384289876	down
			MSTRG.10286.2	-2.296876105	down
			MSTRG.108811.1	-1.695849876	down
			MSTRG.116750.1	1.160655598	up
			MSTRG.117744.2	10.00030544	up
FOXO1	-0.892254044	down	MSTRG.108560.5	-2.136835727	down
			MSTRG.110124.7	2.928086144	up

			MSTRG.111545.1	-0.696918911	down
GSK3A	0.10592042	up	MSTRG.102914.1	0.695194772	up
			MSTRG.106618.1	-0.323118096	down
			MSTRG.111044.3	-1.423666078	down
			MSTRG.117892.3	-1.352206727	down
			MSTRG.133055.1	1.518355214	up
			MSTRG.133381.12	-1.581109074	down
			MSTRG.134631.1	-0.509101392	down
			MSTRG.136192.1	0.619506817	up
			MSTRG.136197.1	-1.308633625	down
GSK3B	-0.227301843	down	MSTRG.139409.1	0.02971043	up
			MSTRG.101877.1	0.079334886	up
			MSTRG.103771.1	-0.023973242	down
			MSTRG.111347.14	1.340697835	up
NF-kappa B	0.125113106	up	MSTRG.105299.1	0.764673497	up
			MSTRG.109472.1	0.588752099	up
			MSTRG.129991.1	1.060896867	up
			MSTRG.133086.11	-0.935614266	down

Table S5. Primers used for qPCR

primers name	sequence (5'-3')
GAPDH F	ACATACTCAGCACCAGCATCAC
GAPDH R	ATTCTGGCAAAGTGGACATCG
MSTRG.111347.2 F	GTGGGTGGGTGCAAGTGATA
MSTRG.111347.2 R	TTGTGCCTTCCAGTTCTCCC
MSTRG.119476.4 F	CAGAGGATGAGATGGTTGGATGGC
MSTRG.119476.4 R	TCAGTTCAGTCGCTCAGTTGTGTC
MSTRG.143209.3 F	CCTCTTCCGTGATCTCTTCTAGG
MSTRG.143209.3 R	GTAGGATCACACAAGTCACCGT
MSTRG.14510.5 F	TGCATATGAAAACCACCCTCCA
MSTRG.14510.5 R	TCCAGTCACCCGAGTAGCAT
MSTRG.129912.5 F	GTGTGGAACGCAGGCTCAGTAG
MSTRG.129912.5 R	GGTGGTCCAGTGGTTAAGAATCCG
MSTRG.133982.9 F	GTTTGGTGAGTGAGTCCTGGG
MSTRG.133982.9 R	GTGCTTGGCTCCGAATCCT
MSTRG.167273.1 F	TTCAGTTGCGCGTATCAGATCCAG
MSTRG.167273.1 R	TCCAGTGGCTAGAACTCTGTGCTC
MSTRG.205178.3 F	ATTCAGGCTGCCTCTTGTGTTGG
MSTRG.205178.3 R	CTGGAGAGGAACATTGGCTTCTGG
MSTRG.100266.1 F	TAATGTCAGGGTGGCTGGAAG
MSTRG.100266.1 R	CAGTCTCCTTCCTCTCTTGCC
MSTRG.103991.1 F	TTGGATAATGTGGGGCGAGG
MSTRG.103991.1 R	ACGTGACCCCAGCTAAACAC
MSTRG.119236.1 F	GAATACACAGTTCGAGACGGGA
MSTRG.119236.1 R	GGCTGTCACTTTCTCCCCAC
